# Supplementary material for: The network characteristics of classic red tourist attractions in Shaanxi province, China
Source: PLoS One. 2024 Mar 29;19(3):e0299286. doi: 10.1371/journal.pone.0299286 (PMC10980247; doi:10.1371/journal.pone.0299286)
Supplement: S1 Table — (DOCX) [file pone.0299286.s001.docx]

**S1 Table. Network attention of** **scenic spots**

| **Size sorting** | **Scenic spots** | **Network attention** |
| --- | --- | --- |
| **1** | Nanjiniwan Revolutionary Site | 0.902715 |
| **2** | Yan’an Revolutionary Memorial Hall | 0.831570 |
| **3** | Wangjiaping Revolutionary Site | 0.621483 |
| **4** | Xiahe Conference Site | 0.598532 |
| **5** | Zaoyuan Revolutionary Site | 0.523566 |
| **6** | Anwu Youth Training Class Revolution Site | 0.516565 |
| **7** | The site of the transportation liaison station in the revolutionary base area of Weibei | 0.516450 |
| **8** | Martyrs' Cemetery of Kangzhuang Battle | 0.509822 |
| **9** | Malan Revolutionary Site | 0.478405 |
| **10** | Memorial Site of the 120th Division of the Eighth Route Army in Commemoration of Anti-Japanese Oath | 0.470860 |
| **11** | ‘Xi'an Incident’ Memorial Hall | 0.462289 |
| **12** | Sichuan-Shaanxi Revolutionary Base Memorial Hall | 0.455298 |
| **13** | Fenghuangshan Revolutionary Site | 0.378926 |
| **14** | Qianspoling Battlefield Ruins | 0.372499 |
| **15** | The Former Site of the Shaanxi-Gansu-Ningxia Border Region Government | 0.349204 |
| **16** | The former site of the General Rear Hospital of the Fourth Red Army | 0.337614 |
| **17** | The Memorial Hall of the Eighth Route Army Xi'an Office | 0.332220 |
| **18** | Fuping County Youth Education Base | 0.331529 |
| **19** | Fumai Battle Memorial Hall | 0.327638 |
| **20** | Yangjialing Revolutionary Site | 0.319207 |
| **21** | Wuyaobao Conference Site | 0.303737 |
| **22** | Niutuiling Battle Site | 0.299995 |
| **23** | Former site of the Headquarters of the 25th Red Army | 0.286749 |
| **24** | Qingliangshan Revolutionary Site | 0.276998 |
| **25** | Xiaoshiya Revolutionary Site | 0.275489 |
| **26** | Martyrs' Cemetery of "48" | 0.225547 |
| **27** | The Memorial Hall of the Chinese People's Anti-Japanese Military and Political University | 0.197175 |
| **28** | The former site of the Shaanxi-Gansu-Ningxia Border Region Revolutionary Base in Zhaojin | 0.187324 |
| **29** | The Suide County Revolutionary History Museum | 0.185564 |
| **30** | Liangdang Uprising Memorial Site | 0.162800 |
| **31** | Revolutionary Site of the Northwest Bureau of the Communist Party of China Central Committee | 0.143908 |
| **32** | Qiaorugou Revolutionary Site | 0.140278 |
| **33** | Yangjiagou Revolutionary Site | 0.140184 |
| **34** | Baotashan Scenic Area | 0.125831 |
| **35** | Shenspringbao Revolutionary Memorial Hall | 0.117588 |
| **36** | The former site of the Headquarters of the 29th Red Army | 0.105982 |
| **37** | Lochuan Conference Memorial Hall | 0.103774 |
| **38** | Baoan Revolutionary Site | 0.101739 |
| **39** | Wuqi Town Revolutionary Site | 0.080962 |
| **40** | Wei Hua Uprising Memorial Hall | 0.012064 |
