# Supplementary material for: The network characteristics of classic red tourist attractions in Shaanxi province, China
Source: PLoS One. 2024 Mar 29;19(3):e0299286. doi: 10.1371/journal.pone.0299286 (PMC10980247; doi:10.1371/journal.pone.0299286)
Supplement: S2 Table — (DOCX) [file pone.0299286.s002.docx]

**S2 Table. Data Processing**

In order to enhance the aesthetic appeal of the network structure of tourist flows, this study has made modifications to the relevant attraction names. The specific modifications are as follows:

| Raw data | Data processing |
| --- | --- |
| \| Eighth route army's memorial hall \| \| --- \| | Memorial hall |
| Caiwenji memorial hall | Caiwenji |
| Xi'an incident memorial hall | Xi'an incident |
| Datang furong garden | Furong garden |
| \| Qin terracotta army \| \| --- \| | \| Terracotta army \| \| --- \| |
| \| Small wild goose pagoda \| \| --- \| | \| Small pagoda \| \| --- \| |
| \| Xi'an revolutionary park \| \| --- \| | \| Revolutionary park \| \| --- \| |
| \| Daming palace national park \| \| --- \| | \| Daming palace \| \| --- \| |
| \| Seg international shopping center \| \| --- \| | \| Seg \| \| --- \| |
| \| Wangjiaping revolution site \| \| --- \| | \| Wangjiaping \| \| --- \| |
| \| Shaanxi history museum \| \| --- \| | \| Shaanxi museum \| \| --- \| |
| \| Xicang flower market \| \| --- \| | \| Flower market \| \| --- \| |
| \| Great wild goose pagoda \| \| --- \| | \| Great pagoda \| \| --- \| |
| \| Xi'an jiaotong university \| \| --- \| | \| Jiaotong university \| \| --- \| |
| \| Yan'an revolution memorial hall \| \| --- \| | \| Yan'an memorial hall \| \| --- \| |
| \| Yangjialing revolution site \| \| --- \| | \| Yangjialing \| \| --- \| |
| \| Zaoyuan revolution site \| \| --- \| | \| Zaoyuan \| \| --- \| |
| \| Tang west market museum \| \| --- \| | \| West museum \| \| --- \| |
| Shaanxi radio and television tower | \| Television tower \| \| --- \| |
| \| Zhaojin revolution site \| \| --- \| | \| Zhaojin \| \| --- \| |
| Zhaojin danxia national geopark | \| Danxia \| \| --- \| |
| \| Anwuqing training revolution site \| \| --- \| | \| Anwuqing \| \| --- \| |
| \| Qiaoergou revolution site \| \| --- \| | \| Qiaoergou \| \| --- \| |
| \| Qingliang mountain revolution site \| \| --- \| | \| Qingliang mountain \| \| --- \| |
| \| Wangjiaping revolution site \| \| --- \| | \| Wangjiaping \| \| --- \| |
| \| Wayao fortress conference site \| \| --- \| | \| Wayao fortress \| \| --- \| |
| \| Phoenix mountain revolution site \| \| --- \| | \| Phoenix mountain \| \| --- \| |
| \| Xiahe conference site \| \| --- \| | \| Xiahe \| \| --- \| |
| Shenquan fort memorial hall | \| Shenquan fort \| \| --- \| |
| \| Yangjiagou revolution site \| \| --- \| | \| Yangjiagou \| \| --- \| |
| \| Memorial hall of the Chinese people's anti-Japanese military and political university \| \| --- \| | \| Military and political university \| \| --- \| |
| \| Revolution site of the northwest bureau \| \| --- \| | \| Northwest bureau \| \| --- \| |
| \| China red bookstore \| \| --- \| | Red bookstore |
| \| "48" martyrs' cemetery \| \| --- \| | \| "48" cemetery \| \| --- \| |
| \| Yan'an cultural and art center \| \| --- \| | \| Cultural and art center \| \| --- \| |
| \| Nanniwan revolution site \| \| --- \| | \| Nanniwan \| \| --- \| |
| \| Baoan revolution site \| \| --- \| | \| Baoan \| \| --- \| |
| \| Liuzhidan martyrs' cemetery \| \| --- \| | \| Liuzhidan cemetery \| \| --- \| |
| \| Shaanxi-Gansu-Ningxia region government site \| \| --- \| | \| Government site \| \| --- \| |
| Academic learning institutes | Learning institutes |
| \| Loess plateau observation deck \| \| --- \| | \| Observation deck \| \| --- \| |
| \| Beijing's youth residence \| \| --- \| | \| Youth residence \| \| --- \| |
| \| Cultural tourism industrial park \| \| --- \| | \| Cultural industrial park \| \| --- \| |
| \| Jiangcheng small restaurant \| \| --- \| | \| Jiangcheng \| \| --- \| |
| Sichuan-Shaanxi revolutionary base memorial hall | \| Base memorial hall \| \| --- \| |

Note: Attractions and mergers handling

1.Xi'an incident memorial hall：the site of the Xi'an incident；2.Eighth route army's memorial hall：the former site of the eighth route army office;3.Xi'an wall：Ancient city walls, city walls,4.Hukou waterfall：Yan'an Waterfall；5.Yuanjia village:Yuanjia village guanzhong lmpression experience store.
