# Supplementary material for: The network characteristics of classic red tourist attractions in Shaanxi province, China
Source: PLoS One. 2024 Mar 29;19(3):e0299286. doi: 10.1371/journal.pone.0299286 (PMC10980247; doi:10.1371/journal.pone.0299286)
Supplement: S1 File — (DOCX) [file pone.0299286.s003.docx]

| 景点 | 搜索数量 |
| --- | --- |
| 八路军西安办事处纪念馆 | 4,240,000 |
| “西安事变”纪念馆 | 5,350,000 |
|  | 2,080,000 |
| 延安革命纪念馆 | 20,900,000 |
| 枣园革命旧址 | 5,680,000 |
| 杨家岭革命旧址 | 5,880,000 |
| 王家坪革命旧址 | 3,640,000 |
| 凤凰山革命旧址 | 5,100,000 |
| 清凉山革命旧址 | 2,810,000 |
| “四八”烈士陵园 | 1,460,000 |
| 洛川县洛川会议纪念馆 | 901,000 |
| 子长县瓦窑堡会议旧址 | 618,000 |
| 宝塔山景区 | 6,030,000 |
| 桥儿沟革命旧址 | 534,000 |
| 南泥湾革命旧址 | 3,310,000 |
| 中共中央西北局革命旧址 | 1,110,000 |
| 陕甘宁边区政府旧址 | 33,000,000 |
| 志丹县保安革命旧址 | 509,000 |
| 吴起镇革命旧址 | 891,000 |
| 中国人民抗日军政大学纪念馆 | 3,290,000 |
|  | 490,000 |
|  | 2,150,000 |
|  | 369,000 |
| 米脂县杨家沟革命旧址 | 761,000 |
| 佳县神泉堡革命纪念馆 | 200,000 |
| 绥德县革命历史纪念馆 | 394,000 |
| 凤县两当起义纪念地 | 1,040,000 |
| 眉县扶眉战役纪念馆 | 350,000 |
| 汉中市洋县华阳红二十五军司令部旧址 | 128,000 |
| 西乡县红二十九军军部旧址 | 25,900 |
| 红四方面军总后医院旧址 | 43,400 |
| 安康市汉滨区牛蹄岭战役遗址 | 64,700 |
| 商洛市商南县前坡岭战斗遗址 | 55,000 |
|  | 323,000 |
|  | 27,600 |
|  | 293,000 |
| 富平县青少年教育基地 | 130000 |
| 八路军 120师抗日誓师纪念地 | 194,000 |
| 渭北革命根据地交通联络站故址 | 6,160 |
| 康庄战斗烈士陵园 | 453,000 |
