# Supplementary material for: The network characteristics of classic red tourist attractions in Shaanxi province, China
Source: PLoS One. 2024 Mar 29;19(3):e0299286. doi: 10.1371/journal.pone.0299286 (PMC10980247; doi:10.1371/journal.pone.0299286)
Supplement: S2 File — (DOCX) [file pone.0299286.s004.docx]

1-西安市红色旅游系列景区(八路军西安办事处纪念馆,“西安事变”纪念馆)

1、八路军西安办事处纪念馆：4,240,000

2、“西安事变”纪念馆：5,350,000

2.汉中市川陕革命根据地纪念馆

2,080,000

3.延安市延安革命纪念地系列景区(延安革命纪念馆,枣园革命旧址,杨家岭革命旧址,王家坪革命旧址,凤凰山革命旧址,清凉山革命旧址,“四八”烈士陵园,洛川县洛川会议纪念馆,子长县瓦窑堡会议旧址,宝塔山景区,桥儿沟革命旧址,南泥湾革命旧址,中共中央西北局革命旧址,陕甘宁边区政府旧址,志丹县保安革命旧址,吴起镇革命旧址,中国人民抗日军政大学纪念馆)

延安革命纪念馆：20,900,000

枣园革命旧址：5,680,000

杨家岭革命旧址：5,880,000

王家坪革命旧址：3,640,000

凤凰山革命旧址：5,100,000

清凉山革命旧址：2,810,000

“四八”烈士陵园：1,460,000

洛川县洛川会议纪念馆：901,000

子长县瓦窑堡会议旧址：618,000

宝塔山景区：6,030,000

桥儿沟革命旧址：534,000

南泥湾革命旧址：3,310,000

中共中央西北局革命旧址：1,110,000

陕甘宁边区政府旧址：33,000,000

志丹县保安革命旧址：509,000

吴起镇革命旧址：891,000

中国人民抗日军政大学纪念馆：3,290,000

4.咸阳市旬邑县马栏革命旧址

490,000

5.铜川市陕甘边照金革命根据地旧址

2,150,000

6.渭南市华县渭华起义纪念馆

369,000

7.榆林市红色旅游系列景区(米脂县杨家沟革命旧址,佳县神泉堡革命纪念馆,绥德县革命历史纪念馆)

米脂县杨家沟革命旧址：761,000

佳县神泉堡革命纪念馆：200,000

绥德县革命历史纪念馆：394,000

8.宝鸡市红色旅游系列景区(凤县两当起义纪念地,眉县扶眉战役纪念馆)

凤县两当起义纪念地：1,040,000

眉县扶眉战役纪念馆：350,000

9.陕南红军革命根据地系列景区(汉中市洋县华阳红二十五军司令部旧址,西乡县红二十九军军部旧址及红四方面军总后医院旧址;安康市汉滨区牛蹄岭战役遗址;商洛市商南县前坡岭战斗遗址)

汉中市洋县华阳红二十五军司令部旧：128,000

西乡县红二十九军军部旧址：25,900

红四方面军总后医院旧址：43,400

安康市汉滨区牛蹄岭战役遗址：64,700

商洛市商南县前坡岭战斗遗址：55,000

1. 咸阳市泾阳县安吴青训班革命旧址

323,000

1. 黄陵县陕甘边小石崖革命旧址

27,600

1. 靖边县小河会议旧址

293,000

1. 富平县红色旅游系列景区(富平县青少年教育基地、八路军 120师抗日誓师纪念地、渭北革命根据地交通联络站故址、康庄战斗烈士陵园)

富平县青少年教育基地：130,000

八路军 120师抗日誓师纪念地：194,000

渭北革命根据地交通联络站故址：6,160

康庄战斗烈士陵园：453,000
