# Supplementary material for: The network characteristics of classic red tourist attractions in Shaanxi province, China
Source: PLoS One. 2024 Mar 29;19(3):e0299286. doi: 10.1371/journal.pone.0299286 (PMC10980247; doi:10.1371/journal.pone.0299286)
Supplement: S3 File — (DOCX) [file pone.0299286.s005.docx]

1-西安市红色旅游系列景区(八路军西安办事处纪念馆,“西安事变”纪念馆)

八路军西安办事处纪念馆

1、余川、（评论：21 喜欢：3 观看： ）

2、anbaby、（出行时间：2018-3-8）、（点赞：18 评论：10 喜欢：10 观看：431）（回民街——华清池——八路军办事处旧址——钟鼓楼——小雁塔景区——城墙——大明宫遗址公园）

3、庐江周公瑾——2020-1-22、（点赞：10 评论：2 喜欢：3 观看：504）（蔡文姬纪念馆——西安事变纪念馆——八路军西安办事处纪念馆——杨虎城别墅（止园）——大唐西市博物馆——大雁塔——大唐不夜城——城墙）

4、老憨歌、（点赞：143 评论：12 ）

5、莉薇、、、（2016-8-24）、（点赞：35 评论：6 喜欢：26 观看：1902）（大唐芙蓉园——八路军西安办事处纪念馆）

6、枫树、、鸟、（点赞：61 评论：2 ）

7、与、、不同、（2012-3-3）（点赞：1700（1.7千） 喜欢：5 观看：观看：2314）、（钟鼓楼——城隍庙——回民街——湘子庙——西安孔庙——西安碑林博物馆——小雁塔——西安博物院——八路军西安办事处纪念馆）

8、萧、、邂、（点赞：7 喜欢：4 ）

9、知足常乐、（2017-12-24）、（点赞：8 喜欢：5 观看：924）

10、一叶菩提、（点赞：21 ）

11、小老汉、（点赞：31 评论：2 喜欢：1）

12、竖琴、、（2019-10-20）、（点赞：6喜欢：1观看：99）、（大雁塔——秦兵马俑——八路军西安办事处纪念馆——钟鼓楼——回民街）

13、那朵花儿、（2019-5-1）、（点赞：80 评论：2 喜欢：63 观看：6405）、（八路军西安办事处纪念馆——秦兵马俑——大雁塔）

14、大秦酒瓶、、（点赞：4 喜欢：1 观看：52）

15、龙吻你的地盘、（点赞：16 喜欢：1）

16、虎爪与花猫、（2014-9-12）、（点赞：4 评论：9 喜欢：3 观看：922）

17、笑天路、、（点赞：10 ）

18、SC、（2017-11-6）、（点赞：23 评论：14 喜欢：12 观看：1457）、（西安事变旧址——钟鼓楼——回民街——高家大院、八路军办事处——小雁塔——明城墙）

19、凤岐、（点赞：3）

20、jesscia、（2020-5-31）、（点赞：12 喜欢：9观看：2456）

21、大眼玉、（2020-4-30）、（点赞：63 喜欢：17 观看：871）、（西安革命公园——八路军西安办事处纪念馆——钟鼓楼——半坡博物馆）

22、jeffre、、（2016-7-1）、（点赞：16 评论：7 观看：714）、

23、汛水的渔（2019-4-23）、（点赞：1 ）

24、西洲小侯、（点赞：22 喜欢：4 ）

25、暖暖、（点赞：8 喜欢：2 观看：241）、

26、燕子、（点赞：1 喜欢：1 ）

27、东北农村穷游客、（点赞：5 ）

28、墅e族、、（2022-10-19）、（点赞：61 评论：3 喜欢：19 观看：164、）、（八路军西安办事处纪念馆——大明宫国家遗址公园——青龙寺）

29、狮子座流行、

30、阿宽（2020-11-9）、（点赞：65 喜欢：45 观看：1689）、（钟楼——鼓楼——碑林博物馆——古城墙——西安事变纪念馆、八路军西安办事处纪念馆——赛格国际购物中心——永兴坊——大雁塔）

31、waterloo、

32、云淡风轻、（点赞：2）

33、山高客远、（点赞：2

34 、诗意的栖居、(点赞 67 评论 :1 喜欢 :19 观看 :648（延安瀑布——王家坪革命旧址——黄帝陵（陵园）、八路军西安办事处纪念馆）

1. 久留米、（2018-10-31）、（点赞：8 喜欢:4 观看:216）、（八路军西安办事处纪念馆——小雁塔——西安博物院——大雁塔）
2. 琳琳爹、、（点赞：83 评论：29 观看：83）
3. 忘乎君、（点赞：20 ）
4. 马蜂窝用户、（点赞：1）
5. 大小狐狸（点赞：2）
6. 始终在这里（点赞：1）
7. 少年由于的烦、（点赞：26 ）
8. 佳爷1230、（点赞：1）
9. 晓晓、（点赞：1）
10. 年少春、、（点赞：5 评论：1）
11. 2022、（点赞：1）
12. 田鼠、（点赞：7）
13. Standfast、（点赞：10 ）
14. 山本无情、、（点赞：8）
15. 山高客远、（点赞：1）
16. Niaomamo、（点赞：3）
17. 仗剑走天涯、（点赞：2）
18. 金刀向、（点赞：1）
19. 为了旅游而奋斗、（点赞：1）
20. 嘎啦耍、（点赞：1）
21. 酒樽、（点赞：8）
22. 奇幻旅游、（点赞：24 ）
23. 高金卫、（点赞：1）
24. 回家啦、（点赞：6 评论：3）
25. 溪的美、风的、（点赞：6 评论：1）
26. 山本无情、、（点赞：4）
27. Y.B.L（点赞：3）
28. 慕圣堂、（点赞：3）
29. 啊不咋比、（点赞：4）
30. 陕西虎牙、（点赞：1）
31. Jesscia、（点赞：1）
32. Nina、（点赞：1）
33. 慕圣堂、（点赞：1）
34. 天才向哥、、（点赞：1）
35. 寇、（点赞：3）
36. Hay 、、（点赞：5）
37. 回家啦、（点赞：5 观看：1）
38. 方一、（点赞：1）
39. 天才向哥、、（点赞：1）
40. 林北、（点赞：1）
41. YOYO、
42. 苏子墨
43. 余柯南
44. 、睿、、（喜欢：1）
45. 爱转转、
46. Song
47. 彩虹姐姐（点赞：15 喜欢：1）
48. Song
49. HQH2014、（评论：1）
50. （SST LEE）、
51. 庐江周公瑾
52. 似水往昔
53. 波波爱马蜂窝
54. 姚远程
55. 第五元素
56. 笨蠢
57. G时代
58. ROOi
59. Feng
60. 至乐
61. 陈
62. 邢教练
63. 爱美丽
64. Jesscia

“西安事变”纪念馆

1. 大唐小狐狸、（点赞：61 评论：7 喜欢：6 ）
2. Hoojane、（2013春）（点赞：69 评论：44 喜欢：43 观看：9795）（西安事变纪念馆——碑林博物馆——大雁塔——大慈恩寺——大唐芙蓉园）
3. 尘里满（点赞：21 喜欢：3 ）
4. 东北农村、、（点赞3）
5. 梓桉、（点赞：54 评论：4 喜欢：1）
6. 京、（点赞：88 喜欢：14 ）、（陕西历史博物馆——西安事变纪念馆——高家大院——回民街）
7. 小啊葱、、（2014-11-12）、（点赞：37 评论：2 喜欢：31 观看：4078）（华清宫——西安事变纪念馆）
8. 万柒（三水）、（点赞：22 喜欢：4 ）
9. 徐来、（点赞：42 喜欢：1 ）
10. 大风乎乎、（2019-9-12）、（点赞：12 喜欢：6 观看：799）、
11. 自言自语、（点赞：23 评论：2 喜欢：2 ）
12. CHENJU、（2016-8-31）、（点赞：146 评论：30 喜欢：25 观看：2301）、（西安事变纪念馆——天主教堂）
13. 三箱、、（点赞：21 评论：1 喜欢：1 ）
14. Jesscai （2020-5-16）、（点赞：16 喜欢：11观看：1176）
15. 小老汉|（点赞：15 评论：2）
16. 枫树、、（点赞：42 评论：1）
17. Wilfred 、（点赞：1）
18. 微微、、（2019-3-28）、（点赞：10 喜欢：6 观看：191）、（西安事变纪念馆——永兴坊——钟鼓楼）
19. 保留曲目、（点赞：1）
20. 乔、（2014年初秋）、（点赞：8 评论：8 喜欢：6 观看：851）
21. Angel 、、（2018-5-25）、（点赞：29 评论：3 喜欢19 gk ；999）、（西安博物院——小雁塔——袁家村关中印象体验店——西安事变纪念馆）
22. Love（点赞：30）
23. 是云云、、（2016-1-1）、（点赞：56 评论：4 喜欢：17 观看2695）、（西安事变纪念馆）
24. 珞瑜、（点赞：1）
25. 龙行天下、（喜欢：9）
26. 萨菲娜、（2018-5-23）、（点赞83 评论：10 喜欢：21 观看：3672）、（西安事变纪念馆——西安博物馆——青龙寺——回民街——西安城墙——华山）
27. 格日勒、、（2019-1-8）、（点赞：101 评论：11 喜欢：58 观看：2715）、（广仁寺——西仓花卉市场——西安事变纪念馆——平仄咖啡馆）
28. 。
29. 我爱、、（点赞：4）
30. 西北麻狼、（点赞：26 评论：2）
31. Falazhang、（2017-8-20）、（点赞：18 评论：9 喜欢：5 观看：524）、（半坡博物馆——秦兵马俑——大雁塔——西安事变纪念馆——西安交通大学）、
32. 超长发挥|
33. 云知道、（点赞：17 评论：3 喜欢：2 ）
34. 雪泡儿、（点赞：6 ）
35. 阿兰、（2019-6-3）、（点赞：7 喜欢：2 观看：82）、
36. 、、GAE、（点赞：4 ）
37. 完全燃烧|（点赞：14）
38. 飘逸的张、、（2016-12-11）、（点赞：40 评论：13 喜欢：24 观看：1040）、（城墙——西安事变纪念馆——书院门）
39. 王冕、（点赞：13）
40. 机智过人的、、（2019-10-1）、（点赞：79 评论：5 喜欢：36 观看：1647）
41. 波妞爸爸、（点赞：57 评论：1 喜欢：15 ）
42. 江州客、（点赞：2）
43. 收获季、（）
44. 朝行转换、（2017-9-13）、（点赞：51 评论：3 喜欢：19 观看：2797）、（陕西历史博物馆——大雁塔——西安事变纪念馆）
45. ——、（点赞：3）
46. 胡子哥哥、（点赞：2 喜欢：1）
47. 仙剑小鱼、（2018-12-13）、（点赞157 评论：5喜欢：18 观看：2446）、（古城墙——书院门——碑林博物馆——西安事变纪念馆——回民街——高家大院）
48. 机智过人的、、（点赞：4 欧伦：1 喜欢：2）
49. 红桃小六哥、（）
50. 九度、、（点赞：3）
51. 徐八九、（点赞：1）
52. Tortoise 、
53. Happy、、（2018-1-25）、（点赞：17 喜欢：9观看：806）、（西安事变纪念馆——西安古城墙）
54. 曹三马、
55. 荔枝、（点赞：4）
56. 国良、（点赞：5）
57. 西北麻狼|（点赞：22 评论：2）
58. 不起长名字、、（2017-11-17）、（点赞：11 评论：2 喜欢：6 观看：511）、（陕西历史博物馆——大雁塔——西安事变纪念馆——钟楼——永兴坊——古城墙、宝塔山——延安革命纪念馆——杨家岭革命旧址——枣园——延安1938）
59. 圆脸怪、（点赞：4 喜欢：1）
60. 毛毛的记忆、（2016-10-15）、（点赞;20 评论：4 喜欢：10观看：1302）
61. Jason （点赞4）
62. 猕猴桃、（点赞1）
63. 凤凰舞（）
64. DF随心所欲、（点赞：6 评论：4喜欢1观看：1385）（西安事变纪念馆——碑林博物馆——西安城墙——钟鼓楼——回民街）
65. Kerl （点赞：3）
66. 阿呆手机、（点赞：3）
67. Andreea （）
68. 祭、（2018-4-5）、（点赞：14 评论：4喜欢：5 观看：374）、（碑林博物馆——西安事变纪念馆——大唐西市博物馆）
69. 至真、（点赞：1）
70. 二班小田、（点赞：2）
71. 阿呆手、（点赞：2）
72. Geoyeo （点赞：2）
73. 孤独患者（点赞：13）
74. 知足常乐、（2018-8-31）、（点赞26 喜欢：17 观看：3451）、
75. 东仔（点赞：9）
76. JESSCIA （）
77. Gto-1314、
78. 世间
79. 马蜂窝用户
80. 奇尼的（点赞：12 喜欢：3观看：278）、（西安事变纪念馆——陕西历史博物馆——大雁塔——钟鼓楼）
81. 娱乐小圆圈
82. 希格的欧尼、
83. 茗、（点赞：14 评论：4）
84. 森木、（2019-6-25）、（点赞：19 喜欢：5 观看：327）、
85. 骄阳、（点赞：4）
86. 农夫、（点赞：12 观看：1072）
87. NIC（点赞：4 喜欢：1）
88. Leo、（2016-2-26）、（点赞：7 评论：5 希：3观看：294）、
89. 殊途、（点赞：3）
90. 大头、
91. 、、1号（点赞：2）
92. 李新（点赞：2）
93. 北方辣妈（点赞：1）
94. 小悟（点赞：1）
95. 小平（2022-2-11）、（点赞13 喜欢：5 观看325）、
96. 2022（点赞：1）
97. 新干线
98. 开越野、、（点赞：7 喜欢：3 观看;5）
99. 知足常乐|（2018-9-6）、（点赞：21 评论：2 喜欢;3 gk；1378）、、
100. 成粥（点赞：9）
101.
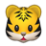
、（2018-9-22）、（点赞：4 喜欢：3 观看：299）
102. 啊呆手机、（点赞：7）
103. 丁沟、（点赞：8）
104. 翰墨、（观看：92）
105. Betty、、（点赞：23）
106. 姜老师
107. 豆豆的爸爸
108. 花椒、、（（2017-12-30）、（点赞：9 喜欢：3 观看：408）
109. Song
110. 不会拐弯、（2016-6-3）、（点赞：6 评论：1 喜欢：3 观看：551）
111. 龙酱、
112. 以为爱旅行的（2020-6-28）、（点赞：1 喜欢：1观看：155）、
113. 啸沙（点赞：8）
114. 知道不知道、（2020-7-18）、（观看：13）、（西安事变纪念馆——西安博物院——小雁塔）
115. Zhzhzhuang、
116. 啊呆手机、（点赞：2）
117. 仗剑走天涯（点赞：1）
118. 蓝色骨头（点赞：2）
119. 我叫宝宝（2019-2-5）、（点赞10 喜欢：5 观看：337）、（大华1935——西安事变纪念馆——言又几）、
120. 心想事成（点赞：2
121. 骑驴、、（点赞：1）
122. Doudou（点赞：2）
123. 起个名字容易、（2018-2-17）、（点赞15 评论：1 喜欢：5观看：504）
124. Doudou（点赞：2）
125. 我就是九、（点赞：19 评论：2 喜欢：8 观看：250）、（鼓楼——西安事变纪念馆——城墙）
126. 李小酷（点赞：2）
127. 桃夭、（点赞：301 评论：35 喜欢：11 观看：4319）、（大雁塔——西安事变纪念馆——回民街
128. 黑白、、（点赞：2）
129. 二班小田、（2016-7-31、（点赞：36 评论：4 喜欢：10 观看：476）、（西安事变纪念馆——陕西广播电视塔）
130. Fade 、（）
131. 小豆粥、（点赞：2）
132. 沈北（点赞：1）
133. 小豆点（点赞：3
134. 笨笨（点赞：1 评论：1）
135. Svan （评论：3）
136. 开心就好（点赞：4）
137. 现在，去年你）
138. 宅宅、（点赞：10 喜欢：5观看;239）、
139. 小聚哈
140. 汉中市川陕革命根据地纪念馆
141. 钱爷爱溜达9dz :3 xih :1
142. 汉中文旅、（点赞：16 ）
143. 行走的motto、（点赞：2）
144. Xiaozhongdong
145. Guoguo （点赞：18 喜欢;1）

4.咸阳市旬邑县马栏革命旧址

1、山峰来客、（点赞：29）

2、城北可乐、（2018-9-15）、（点赞：17 评论：2 喜欢：11观看：1749）

3、放蜂人、（2019-5-1）、（点赞：13 喜欢：7 观看：1785）

4、maxin、（喜欢：1）

5、maggie 、（点赞：1）

6、龟先森、

7、老狮子、（喜欢：1）

8、放蜂人、（喜欢：2）

9、樱木、（点赞：56 喜欢：1）

10、莉儿、（点赞：3）

11、放蜂人、

12、乡间小道、（点赞：9 喜欢：1）

13、放蜂人、

14、馒头、（点赞：5）

15、白发飘飘、（点赞：4）

16、就是那个、（点赞：3）

17、依然自乐、（点赞：2）

18、独立松（点赞：2）

19、我又赢了、

20、丸子

21、三个苹果、（2017-4-30）、（点赞：23 喜欢：6观看：398）、

5.铜川市陕甘边照金革命根据地旧址

1、西部、（2017-5-18）、（点赞：66 评论：6 喜欢：37 观看：4978）、

2、晴儿、（2017-8-12）、（点赞：3400、评论：13 喜欢82 观看：3547）、

3、闲人、（2021-5-26）、（点赞：11 评论：3喜欢：4 观看：811）、

4、温驯的狮子、（2021-5-23）、（点赞：17 喜欢：10 观看：1129）、（照金滑雪场——照金牧场——照金红色小镇——陕甘边照金革命根据地旧址——照金丹霞国家地质公园）

5、傲雪、（2021-4-12）、（点赞：6 喜欢：3 观看：225）（陕甘边照金革命根据地旧址——壶口瀑布）

6、祈福、（点赞：13 喜欢：6 观看：349）、（陕甘边照金革命根据地旧址——照金丹霞国家地质公园）

7、木+、、（点赞：16 评论：12 喜欢：3 观看：2913）、

8、中红旅、（点赞：11 喜欢：6 观看：737）

9、林荫大道、（2020-7-17）、（点赞：13 评论：1喜欢：5 观看：121）

10、阿乱、（2021-7-3）、（随着：7 喜欢：5 观看：469）、（马咀欧洲风情小镇——薛家寨——陕甘边照金革命根据地旧址——照金牧场——溪山胜境）

11、陕西旅行、（观看：9）

12、瑶看世界（观看：36）

6.渭南市华县渭华起义纪念馆

1、白小西西、（点赞：35 评论：4 喜欢：22 观看：1350）

2、墨泪、、（2020-1--9）、（点赞：12 评论：1喜欢：7 观看：352）

3、w1z2m3 （点赞：6 喜欢：3 观看：590）

10.咸阳市泾阳县安吴青训班革命旧址

1、wsl203、（2017-9-24）、（点赞104 评论：5 喜欢：58 观看：1.1万）、

2、浏阳河、（点赞：159 喜欢：12 观看：1858）、（咸阳市泾阳县安吴青训班革命旧址——三元城隍庙）

3、农夫、（2020-9-9）、（点赞：2 喜欢：1 观看：238）

11.黄陵县陕甘边小石崖革命旧址

1、哥行天下、（2019-9-24）、（点赞：64 评论：2喜欢：25 观看：1442）、

2、w1z2m3 （2019-7-27）、（点赞：16 评论：2 喜欢：8 观看：1591）、

3、旅游.头条、（点赞：8 喜欢：1、观看：8）

4、意识散、（2020-5-1）、（点赞：342 评论：11 喜欢：102 观看：3624）、（枣园——杨家岭——桥儿沟鲁艺——清凉山——一二街）（王家坪——钟山石窟——瓦窑堡会议旧址——凤凰山革命旧址——西安）

5、乐逍遥、（2018-9-20）、（点赞：28 喜欢：9观看：602）、（延安革命纪念馆——王家坪革命旧址）

6、错错|（点赞：16 评论：2 喜欢:7 观看：705）、（枣园——杨家岭——延安革命纪念馆——宝塔山）

7、伤情书生、（2021-7-20）、（点赞：101 点赞：4 喜欢：8观看：447）、（黄陵县——黄帝陵——杨家岭——宝塔山——陕西壶口瀑布）

8、空军叔叔|（2018-5-30）、（点赞：11 喜欢：3 观看：890）、（雨岔大峡谷——延安革命纪念馆——清凉山中共新华社——枣园——波浪谷景区）

12.靖边县小河会议旧址

1、旅游之声（2017-10-7）、（点赞：5 喜欢：3 观看：1024）、

2、小沈哥（点赞：16）、

3、小沈哥（点赞：15）

4、云淡风轻（2020-10-5-6）、（点赞：4）（小河会议旧址、王家坪、枣园、延安革命纪念馆）

5、陕北波浪谷、（点赞：6）

8.宝鸡市红色旅游系列景区(凤县两当起义纪念地,眉县扶眉战役纪念馆)

凤县两当起义纪念地

1. 老狮子、（2022-7-1）、（点赞：6 喜欢：3 观看：326）、
2. 沙sir、（点赞：1）
3. 宝鸡文旅（喜欢：1+5、观看：5542 +5115）
4. 未童、（喜欢：1）
5. 、、故人
6. Lucifer（点赞：1+1）
7. 宝鸡文旅（点赞：2 喜欢：2 观看：4922）
8. EGG
9. Panpans
10. 唐
11. 猴主任、、（喜欢：1）
12. 苍蝇
13. 用户
14. 东方
15. 刘
16. 柿饼（点赞：16）
17. 星辰
18. 用户（点赞：3）

7.榆林市红色旅游系列景区(米脂县杨家沟革命旧址,佳县神泉堡革命纪念馆,绥德县革命历史纪念馆)

米脂县杨家沟革命旧址

1. allyear
2. 雪菲、（点赞：26 评论：2 喜欢：1）
3. Huin （点赞：9 评论：1）
4. 福悠
5. 小卷卷（点赞：4）
6. Huin （点赞：1 ）
7. 旅行者
8. 陕北旅行、、
9. 舍予、（点赞：10 喜欢：4观看：519）
10. 川、大叔、
11. 其其、（点赞：8 喜欢：2观看：178）
12. Zsp、（点赞：8 ）
13. 秦人、、（2021-10-18）、（点赞：31 喜欢：10 观看;94）
14. 快乐旅程、（点赞：114 评论：21 喜欢：1 观看：114）
15. 静体自然(（2020-9-18）、（点赞：9 喜欢：1 观看：185）、（香炉寺——神泉堡革命纪念馆——杨家沟革命纪念馆——万佛洞）
16. 雪菲、（点赞：10）
17. 冬凌草、（2019-5-1）、、（点赞：183 评论175 喜欢8 观看927）、（杨家沟——姜氏庄园——李自成行宫）
18. 安徽塑料（点赞：62 评论：1 喜欢：4 观看：62）、
19. 雪菲、（点赞：26 评论：2 喜欢：1 观看：26）

佳县神泉堡革命纪念馆

1. 行者无疆|（点赞：59 评论：1 喜欢：2 ）
2. 其其（点赞：8 喜欢：3观看：170）
3. 福悠（点赞：1）

绥德县革命历史纪念馆

1. 朱利安、
2. 丁香、、（2021-1-1）、（点赞：28 评论：9 喜欢：1）、

13.富平县红色旅游系列景区(富平县青少年教育基地、八路军 120师抗日誓师纪念地、渭北革命根据地交通联络站故址、康庄战斗烈士陵园)

富平县青少年教育基地

1. rudy 钟（点赞：1）

八路军 120师抗日誓师纪念地

1、

渭北革命根据地交通联络站故址

1、

康庄战斗烈士陵园

1、

9.陕南红军革命根据地系列景区(汉中市洋县华阳红二十五军司令部旧址,西乡县红二十九军军部旧址及红四方面军总后医院旧址;安康市汉滨区牛蹄岭战役遗址;商洛市商南县前坡岭战斗遗址)

汉中市洋县华阳红二十五军司令部旧址

1. 老郭、（2018-8-12）、（点赞：18 评论：1 喜欢：7 观看;6167）

西乡县红二十九军军部旧址及红四方面军总后医院旧址

1. 西文图、（点赞：5 评论：1 喜欢：1）、
2. 蓑笠翁、

安康市汉滨区牛蹄岭战役遗址

1、

商洛市商南县前坡岭战斗遗址

3.延安市延安革命纪念地系列景区(延安革命纪念馆,枣园革命旧址,杨家岭革命旧址,王家坪革命旧址,凤凰山革命旧址,清凉山革命旧址,“四八”烈士陵园,洛川县洛川会议纪念馆,子长县瓦窑堡会议旧址,宝塔山景区,桥儿沟革命旧址,南泥湾革命旧址,中共中央西北局革命旧址,陕甘宁边区政府旧址,志丹县保安革命旧址,吴起镇革命旧址,中国人民抗日军政大学纪念馆)

中国人民抗日军政大学纪念馆

1. 庄公、（点赞：43）
2. 马蜂窝小可、（2018-7-20）、（点赞;69 pl :5 喜欢：28观看:1923）、（宝塔山、（杨家岭革命旧址——枣园革命旧址——延安1938主题街区）、（延安革命纪念馆——王家坪革命旧址——清凉山——延安新闻纪念馆——宝塔山）、（凤凰山麓革命旧址——中国抗日军政大学旧址——西北局旧址——中国红色书店）
3. 庐江周公瑾、（2019-6-15）、（点赞：76 评论：22 喜欢：50观看：1484）、（宝塔山——凤凰山——延安新闻纪念馆——清凉山）、（杨家岭——四八烈士陵园——枣园——延安革命纪念馆——王家坪——西北局）
4. 小有与大有、（点赞：15）
5. T-BIN（点赞：2）
6. 慵懒的大猫、（点赞：1）
7. 大有与小有（点赞：36 评论：6 喜欢：3 观看：867）、
8. 珞瑜、（点赞：1）
9. Jyun2001、（dz :30 xih :10 观看：2356）、（凤凰山——凤凰山周边——抗日军政大学旧址——西北局——高等法院）、（清凉山革命旧址——延安新闻纪念馆——太和山——延安革命纪念馆——王家坪——延安文艺中心（点赞：27 评论：1 喜欢：12 ））、（宝塔山——杨家岭——路遥墓——鲁艺旧址——鲁艺文化园区（点赞：31 评论：1 喜欢：13））、（枣园——四八烈士陵园——中央军委三局——北京知青博物馆——延安科技馆）
10. （三个金）、（点赞：2）
11. 任侠生、（点赞：1）
12. 新天之骄子、（2020-10-4）、（点赞：4 喜欢：1 观看：170）、（王家坪革命旧址——延安革命纪念馆——杨家岭革命旧址——枣园——抗大——凤凰山）、（南泥湾——杜公祠——西北局——宝塔山）
13. 跟着隆哥、、（2016-5-20）、（点赞：149 喜欢：29 观看：199）、（宝塔山——枣园——延安革命纪念馆——延安红军纪念广场）（杨家岭——王家坪——抗大——杜公祠——南泥湾革命旧址）
14. 小有与大有、（点赞：19你评论：1）
15. 大笨（点赞：3）
16. 飞扬（点赞：5）
17. 水田里的稻米、（点赞：1）
18. 乡下人、（点赞：1）
19. 庄公（点赞：13）
20. 客旅的

吴起镇革命旧址

1. zhizhimiao（点赞：103 评论：15喜欢：4
2. 仗剑走天涯、（2018-8-17）、（点赞：7 喜欢：4 观看：357）、
3. 鹤鸣九（点赞：9）
4. 周秦汉唐、（）
5. 天马行空（点赞：1）
6. 闲庭信步
7. 笑苍天（点赞：16）
8. 大卫老弟（点赞：6）

志丹县保安革命旧址

1. 佟湘玉、（2018-11-26）（点赞：44 评论：5喜欢：9 ）（永乐寨——保安革命旧址）
2. Jyun2001、（2019-1--5）、（点赞：6 评论：1 喜欢：1 观看：481）、（保安革命旧址——刘志丹烈士陵园——抗大）
3. 仗剑走天涯——（2018-8-18）、（点赞：5 喜欢：3 观看：141）、（刘志丹烈士陵园——保安革命旧址）
4. 三秦小子、（点赞：39 评论：2）
5. 小鱼儿、（点赞：1）
6. 马蜂窝用户（点赞：3）

陕甘宁边区政府旧址

1、0

中共中央西北局革命旧址,

1. 还是没人理、（2019-10-19）（点赞：22 评论：25 喜欢：5 观看：531）
2. Zhouyujin
3. 小有与大有、（点赞：40 评论：6 喜欢：3 观看：462）、
4. Sweet、（评论12 点赞：48 喜欢：274 观看：4.2万）、（延安革命纪念馆——王家坪革命旧址——枣园）
5. 小白、（2021-9-14）、（点赞：2）
6. KEVIN、（点赞：10 评论：10）
7. 多忘（点赞：11评论：10）
8. Jia yu，（点赞：313 评论：3 喜欢：8 观看：1250）、（南泥湾——宝塔山——王家坪——枣园——）
9. 陕西文旅（官方）、（点赞：47 评论：4 喜欢：267 观看：1.9万）
10. 南山八院（点赞：15 喜欢：11观看;1032）、
11. 秀山村民、（2018-10-11）、（点赞427 pl :2喜欢：51 观看：6.2万）、（延安革命纪念馆——王家坪）、（陕甘宁边区政府——西北局）、（延安新区学习书院——杨家岭——四八烈士）
12. 王、、吉（2017-4-3）、（点赞：20 评论：2 喜欢：14 观看：781）、（杨家岭——枣园——王家坪——延安革命纪念馆——壶口瀑布——宝塔山）

南泥湾革命旧址,

1. 半日闲、（2021-5-25）、（点赞：19）
2. 陕西中青旅（攻略）、（点赞：179 评论：13 喜欢：1887观看：531520）
3. 阿诺（点赞：42 评论：2）
4. 小蜂（攻略）、（点赞：26 喜欢：412 喜欢：3420 观看：178904）
5. 陕西文旅（评论：2 点赞：12 喜欢：102 观看：18150）
6. 马建军律师（点赞：13）
7. 诗远（点赞：60 评论：3）
8. 闲云野鹤（点赞：9）
9. 诗远（点赞：59 评论：2）
10. Herryon
11. 落英缤纷（点赞：21 评论：2 喜欢：1）
12. 诗远（点赞：53 评论：3）
13. 13704039、、（点赞：1）
14. ~悠云~、（2017-1-13）、（点赞：126 评论：5 喜欢：94 观看：3529）
15. 、、雨、（点赞：3）
16. 快乐老顽童（2021-5-13）、（点赞：68 评论：3 喜欢：13 观看：376）、（黄河湖楼瀑布——南泥湾）、（延安革命纪念馆——枣园——杨家岭）、（宝塔山——清凉山）
17. TRY、（点赞：4）
18. 啦啦
19. Lijian（2020-10-13）、（点赞：12 喜欢：6观看：451）、（二道街——枣园——延安1938主题街区——杨家岭）、（南泥湾——壶口瀑布）
20. 德小德（点赞：4）
21. 鱼蛋粗面——（2016-6-7）、（点赞：44 评论：2 喜欢：6 观看：1130）、（壶口瀑布——南泥湾）
22. 双维（点赞：3）
23. 心情玫瑰（点赞：3）
24. 小炳88（2016-5-30）、（点赞：74 评论：4 喜欢：18 观看：1043）、（枣园——延安大学——杨家岭——延安革命公园——延安新闻纪念馆——清凉山）、（南泥湾——壶口瀑布）、（兴庆宫公园——八路军办事处——城墙）、
25. 玉树临风（点赞：1 评论：1）
26. 一意孤行（2016-10-10）、（点赞：82 评论：27 喜欢：20观看：2003）、（壶口瀑布——南泥湾——宝塔山）、（枣园——乾坤湾）、（杨家岭——宝塔山——清凉山——太和山）
27. 燕miss（2019-4-20）、（点赞：9 喜欢：3 观看;244）、（南泥湾——王家坪——枣园——宝塔山——回民街）、（大兴善寺——西安事变纪念馆——大唐芙蓉园——慈恩寺遗址公园——大慈恩——大雁塔——不夜城）
28. 钱爷爱溜达（点赞;5 xih :1）
29. 梅苏特（点赞：16）
30. Gavin（点赞：3）
31. 龙哥杰森（点赞：5
32. 愚翁（点赞：5 评论：1 喜欢：1）
33. 伯文（点赞：13 ）
34. 秋秋、、（2017-5-26）、（点赞：121 评论：30 喜欢：2 观看;597）
35. 马蜂窝星探（点赞：19 喜欢：2 ）
36. O 0、、00、（点赞：2）
37. CoCo、（点赞：7 评论：2 喜欢：4 观看：881）
38. 德小德（点赞：5）
39. 紫涵（点赞：10 评论：3 喜欢：4 ）
40. 吱吱姑、（点赞：17）
41. 翁宁（2019-5-10）、（点赞33 评论：8 喜欢：19 观看：1313）
42. 沙尘（点赞：1）
43. 微波
44. 薇123
45. 慕师兄（点赞：2）
46. 2020
47. 柠檬、、（2016-8-1）、（点赞：20 评论：6 喜欢：6 观看：354）、（壶口瀑布——南泥湾）、（延安革命纪念馆——王家坪——杨家岭）
48. 城市小飞（点赞：6）
49. Derek（点赞：10）
50. 开心每一天（点赞：1）
51. 行走江湖的（点赞：8）
52. 凯婷（点赞：4）
53. 宇徒（2017-8-18）、（点赞：151 观看：961）
54. 乔木在、、（2017-11-10）、（点赞：25 喜欢：10 观看;1059）、（延安革命纪念馆——杨家岭——枣园）、（宝塔山——中山街——凤凰广场）、（南泥湾——黄土高坡观景台——壶口瀑布）
55. 娜姐、、（点赞：16）
56. 雨蓬（点赞：3）
57. 过眼明、（点赞：12 评论：2 喜欢：7 观看：2223）、（南泥湾——王家坪——枣园——北京知青故居）
58. 7秒（点赞：3）
59. Dingdayiyi（2019-7-9）、（点赞：53 评论4 喜欢：8 观看：1276）、（壶口瀑布——北京知青故居）、（南泥湾——宝塔山——王家坪——枣园
60. Nic （点赞4 喜欢;1）
61. 泡泡（点赞：3）
62. 平静如水（点赞：5 评论：1 喜欢：1）
63. 李大喵（点赞：1）
64. 鱼大花（点赞：4）
65. Lvye
66. 闰土（点赞：1）
67. Anjane（点赞：4）
68. 转角遇见海（点赞：1）
69. 孙小姐（点赞：2）
70. 国际、（点赞：1）
71. 糖（点赞：1）
72. 爱小娃
73. Mera
74. Mera
75. 云在海边（点赞：6 喜欢：1 观看：77）、（知青——南泥湾——王家坪——延安革命纪念馆——杨家岭）、（枣园——靖边县波浪谷——牛角寨）
76. Mera
77. Mera
78. 加班、、
79. 胆小鬼
80. 二江
81. 星际（2019-8-5）、（点赞：228 评论：1 喜欢：6 观看：753）、（知青——南泥湾——宝塔山——王家岭——枣园
82. 一路上有你（点赞：1）
83. 方一（点赞：2）
84. Jadek
85. 火锅、
86. 榔头
87. 极爱自驾游
88. 福气他爹
89. 马蜂窝（点赞：4）
90. 马蜂窝（点赞：3）
91. Miki (dz :5
92. One0099（点赞：4）
93. 平平（点赞：1）
94. 清和
95. 麦兜、（点赞：1）
96. 浮生若梦（点赞：1）
97. Ynau（点赞：2）
98. 马蜂窝（点赞：2）
99. 若冰（点赞：13）
100. 小汤圆
101. 海阔
102. 走路
103. Zhm-jinan(2012-10-1）（点赞：4000 评论：483 喜欢：55 观看;5278）、（王家坪——延安革命纪念馆）、（枣园——南泥湾——壶口瀑布）
104. 桑尼（2022-7-24）、（点赞：4 观看：51）、（知青——南泥湾——壶口瀑布）、（枣园——杨家岭）、（宝塔山——延安革命纪念馆）
105. Mera
106. 自由飞翔
107. Mera
108. 砖、、（2018-1-1）、（点赞：18 评论：2 喜欢：5 观看：2486）、
109. Pass、、、（2019-10-7）、（点赞：10 喜欢：5 观看：49）、
110. 糊涂
111. 孤独的守望（2021-5-1）、（点赞：45 评论：29 喜欢：19 观看：644）、（枣园——宝塔山——南泥湾——壶口瀑布）
112. 再来一（点赞：1马蜂窝（点赞：2）
113. 欣欣（点赞：1）
114. 行动者（点赞：1）
115. Babe（2019-3-16）、（点赞：11 喜欢：4 观看：1853）、（延安革命纪念馆——杨家岭——宝塔山）、（黄土高原观景台——壶口瀑布——知青——南泥湾）
116. 旅行。与天（点赞1）
117. 小逃爱（点赞3）
118. 猿、（2017-10-28）、（点赞：48 评论：1 喜欢：31 观看：2860）
119. 祥子（点赞：3）
120. 珙桐、（2017-5-27）、（点赞：63 评论：3 喜欢：44观看：6281）、（壶口瀑布——南泥湾——宝塔山——枣园）
121. 敏夫人（点赞：3）
122. Xiao （点赞：3）
123. 、二哥围脖（2015-10-2）、（点赞：24 评论：4 喜欢：15 观看：3393）、（中心街——延安革命纪念馆——王家岭——枣园）、（壶口瀑布——南泥湾）、
124. 小妖（2021-6-1--）、（点赞：815 评论：1 喜欢：36 观看;1872）、（枣园——杨家岭——金延安）、（延安革命纪念馆——王家坪——宝塔山——红街道）、（南泥湾——壶口瀑布——知青）
125. 温血（）
126. 南瓜团团（2015-7-13）、（点赞：56 评论：5 词汇：32 观看：2130）、（南泥湾——王家坪——延安保卫战——枣园——西安）
127. 米唐（点赞：2）
128. 有故事的人
129. 、、快快、（2018-10-28）、（点赞：48 评论：4 喜欢：25 观看：1557）、（壶口瀑布——南泥湾）、（延安大学——杨家岭——靖边波浪谷）
130. Leilei （点赞：1）
131. Moonlight （2015-8-28）、（点赞：2000 评论：156 喜欢：1100 观看：57703）、（清凉山——宝塔山——枣园——延安革命纪念馆）、（杨家岭——王家坪）
132. 黑夜中de 蜗牛（（2019-3-24）、（点赞：36 评论：2 喜欢：25 观看：1610）、

桥儿沟革命旧址,

1. 五洋（点赞：8）
2. 小有大有（点赞：20 评论：3 喜欢：5观看：1254）、
3. CAI（点赞：1）
4. 仁者、、（2015-9-11）（点赞：15 评论：2 喜欢：2 观看：1449）、（宝塔山——王家坪——杨家岭——枣园——壶口瀑布）、
5. 斯坦尼、、（点赞：11）
6. 绵绵（点赞：2）
7. （点赞：5）
8. 、、宋导（点赞：10）

子长县瓦窑堡会议旧址,

1. 袋鼠、（2021-10-16）、（点赞：8 喜欢：1 观看：102）、
2. 无限接近、、（点赞：7）
3. 黑陶、（）
4. 五洋、（点赞：4）
5. 陕西文旅（官方）、（点赞：36 评论：2 喜欢：187观看17000）

洛川县洛川会议纪念馆

1. 、水的渔（点赞：5）
2. 月半子、（2021-7-29）、（点赞：22 喜欢：9 观看：618）、（洛川会议纪念）
3. 融化（2019-8-15）、（点赞：85 喜欢：13 观看：17147）、（延安革命纪念馆——靖边波浪谷）
4. 梅苏特（点赞：17）
5. 闲庭信步
6. 梁状（2020-10-10）、（点赞：6 喜欢：2 观看;295）
7. 超妖、、
8. 兰采和（2020-10-21）、（点赞：89 评论：1 喜欢：8 观看：588）、
9. 超妖、、
10. Rabbit
11. 吕云、、（点赞：4）
12. 秦岭、、
13. 生命里的（点赞：2）
14. 骑在路上（点赞：3）

“四八”烈士陵园,

1. 罗布泊、（点赞：10）
2. 小有与大有

宝塔山景区

1. 侍其孤独行（点赞：50 评论：14 喜欢：2 ）、
2. 炜哥、（2017-5-11）、（点赞：9 喜欢：3 评论：680）、（杨家岭——枣园——王家坪——延安革命纪念馆——宝塔山景区）
3. W1z2m3 （2019-7-27）（点赞：11 喜欢：5 观看：637）
4. 王九爷殿下（点赞：85 评论：19 喜欢：1）
5. 蚂蜂行者（2019-8-31）、（点赞：17 评论：3 喜欢：4 观看：189）
6. 苏鲁豫、、（点赞：53 评论：3 喜欢： 3 ）
7. 三旬奶爸、（点赞：15 喜欢：3 ）
8. 镜头下的旅（点赞：28 喜欢：12）
9. 神奇在路上（点赞：68 喜欢：4 评论：7）
10. 凝杉（点赞：29）
11. 初心依然、（点赞：9）
12. 马爷的马蜂窝（点赞：9）
13. 红樱桃（点赞：24 评论：1 喜欢1）
14. 天驴、（点赞3）
15. 马蜂窝用户（点赞：7）
16. 叮当的大熊（点赞：2 评论：4）
17. Tasi
18. 关炜
19. 洛雯（点赞：13 评论：1 喜欢2）
20. MY网 （点赞：1 评论：2）
21. DONKEY（点赞：8）
22. 曼陀林（）
23. 随遇而安
24. 延安“镜哥”、（点赞：6）
25. 林文（点赞：1）
26. 任强（点赞：1）
27. 沈良（点赞：14）
28. 炜哥
29. 超妖书生
30. Chengxhing、（2022-9-27）、（点赞：1）
31. Zhouyulin
32. 勇者
33. 雪岩
34. 人生就是一场（点赞：1）
35. 哥行天下（点赞：16 评论：1）
36. Jonnney
37. 文、、（点赞4
38. 宫嘉悦
39. 梦游字符串（点赞：13）
40. 小白（点赞：1）
41. 追逐
42. 行走的鱼
43. 木、、农夫 （）
44. 自由的飞翔
45. 、落、长安（）
46. Amway
47. 小李是个、、
48. 哦呼（点赞：16）
49. 、、非、、
50. 余拾柒、（点赞：1）
51. 冰封的旋律
52. 南飞雁（点赞：3 评论：1）
53. 伯牛
54. Bail（点赞：2）
55. Bail（点赞：2）
56. 橡树的承诺（点赞：1）
57. If（点赞：8）
58. 1360、、81（点赞：10）
59. 渝哥
60. 朱朱（点赞：3 评论：1）
61. 走起来不停、、
62. 和通、、
63. 幸福汉堡、、（点赞：1）
64. 、、仁（点赞：13）
65. 、、长啸（点赞：9 评论：1）
66. 我好爱、、（点赞：11）
67. T-BIN
68. 猫耳朵（点赞：3）
69. 心随景动（点赞：2）
70. 延安“镜哥”
71. 马蜂窝、、
72. 柳、、
73. 粉、、
74. 一意孤行（点赞：5 评论：2）
75. 一凡（点赞：3）
76. 玫瑰千层（点赞：4）
77. 丁香、、（点赞：5）
78. 金星、、（点赞：3）
79. 不会照相的（点赞：4）
80. 挖、、（点赞：3 评论：1 喜欢：1
81. 冰封的旋律
82. 欣、、（点赞：1）
83. 金虎（点赞：2）
84. 流~云（点赞2）
85. 幸福的汉堡（点赞：1）
86. 玉树临风（点赞：1）
87. 可乐ACE（点赞：1）
88. 不简单的、、（点赞：1 喜欢：1）
89. 缘
90. 策马奔腾
91. 马蜂窝
92. 、、非、、
93. Sean kang
94. Diudiu
95. 曼陀罗
96. 君子蓝
97. 百里溪（点赞：22 喜欢：1）

清凉山革命旧址

1. 炎晴（2020-10-2）、（点赞：21 欧伦：3 喜欢：9 观看：990）、（南泥湾——壶口瀑布——北京知青故居）、（清凉山——延安新闻纪念馆——宝塔山）、（延安科技馆——枣园）
2. Ban （点赞：17 评论：5 喜欢：17 观看：1851）
3. Al（点赞：39）
4. 错错、（点赞：16 评论：2 喜欢：7 观看：708）、（清凉山——枣园——杨家岭——延安革命纪念馆——宝塔山）
5. 喵大仙（点赞：122 评论：7 喜欢：205）
6. 顺时针、（点赞“69 喜欢：5”
7. 生于浮、（点赞17 评论：2 喜欢：8 观看：1557）、
8. 快乐旅程（点赞：81 评论：50 喜欢：2 ）
9. 咚咚 （点赞：39喜欢：1）
10. 革命老知青、（2020-10-19）、（点赞：33 评论：38 喜欢：6 观看：505）、
11. 程红（点赞：30 评论：3）
12. 瑞、（**攻略**）（点赞：118 评论：6 喜欢：72 ）
13. 随风、（点赞：28 评论：1 喜欢：5）
14. 、、残柳（点赞：16 评论：4）
15. 浪漫骑士、（2020-7-31）、（点赞：12 喜欢：4 观看：489）、
16. 千帆、、（点赞：44 评论：14）
17. 半日闲（点赞：17）
18. 浪尽天涯、（点赞：6）
19. 还是没人理|（2019-10-19）、（点赞;26 pl :37 喜欢：5 观看：772）、（延安新闻纪念馆——清凉山）
20. 彪、、（点赞：40 评论：14）
21. LIUGE（点赞：14 评论：13 喜欢：2观看3217）、
22. 不、、舟（点赞：7）
23. 一叶菩提、（点赞：11 评论：2 喜欢：1）
24. JJS（点赞：26 评论：1 喜欢：12 观看：747）、（清凉山——延安革命纪念馆——枣园）
25. 黑娃（点赞：59）
26. 大手牵小手（点赞1
27. 吉祥岁月（点赞：2 评论：1）
28. 橘子味的猫、（2018-7-27）、（点赞：22 评论：5 喜欢：8 观看：589）
29. 马蜂窝、、（2019-12-6）、（点赞：18 评论：2 喜欢；13、观看：700）、（宝塔山——凤凰山——二道街）
30. Phelps （点赞：2）
31. 哥行天下（点赞：9 评论：6 喜欢：2）
32. Kun（2018-4-23）、（点赞：8 喜欢：4 观看：250）、
33. 嗅、
34. 张大、、（点赞：10 喜欢：5 观看：354）、（（宝塔山——西北局——清凉山——延安革命纪念馆——王家坪）、（枣园——杨家岭——延安大学）
35. 爱逛吃、、（点赞：1 喜欢：1）
36. 黑石（点赞：1）
37. Dr
38. 小川的
39. 、、残柳（点赞：24 评论：12 喜欢：1）
40. 看看世界、（点赞：16）
41. 大饼油条
42. Kuai （点赞：1）
43. 春天彩虹（点赞：4）
44. Zhouyujin
45. Tsai
46. 周、、（点赞：8）
47. Ediver（点赞：17）
48. 大手牵小手（点赞：16）
49. 刘雨吖（点赞：1）
50. 凡
51. 流浪星
52. Ray
53. 被动的鱼（点赞：10）
54. 依心、、（点赞：8 评论：1）
55. Phoen（点赞：1）
56. 阿狸爱喝
57. 素面朝天（点赞：4 喜欢：1）
58. 大手牵小手、（点赞：15）
59. 依心、、（点赞：3 评论：1）
60. 鸭子（点赞：1 评论：1）
61. 依心、、（点赞：3 ）
62. Ameliaon （点赞：3）
63. 尕、、（点赞：4）
64. 皓月、、公子（
65. 诚实可靠（点赞：2）
66. Winni（点赞：2）
67. 念泥塘（点赞：1）
68. 麋鹿（点赞：7）
69. 方一（dz）
70. 不后悔
71. Winni（点赞：3）
72. 一意孤行（点赞：5 评论：1）
73. 623to 、、（点赞：2）
74. Ynau （点赞：1）
75. Winni（点赞：1）
76. 瑶妈（点赞：1）
77. 我心中尚未、（点赞：1）
78. 、、蓝调（点赞：13 评论：6 喜欢：1）
79. JJS（点赞：6）
80. 623to 、、（点赞：2）
81. Xiake (（点赞：3）
82. 陕北波浪谷小王（点赞：4）
83. 星际旅行者（2019-8-9）、（点赞：241 评论：3 喜欢：19 观看：1434）
84. 右手、（点赞：2 评论：1）
85. 丫丫 （点赞：3）
86. 大手牵小手（点赞：1）
87. 晴天娃娃（2012-10-4）、（点赞：2 观看：2915）、（枣园——杨家岭——延安革命纪念馆）、（清凉山——宝塔山）
88. 西安、、代驾（点赞：1）
89. 李大喵（点赞：1）
90. 坐在夕（2011-7-1）、（点赞：1观看：1181）
91. 碧玉（点赞：1）
92. 、煜（点赞：1）
93. Mr （点赞：1）
94. 贾（下面+）早、、（点赞：1）
95. 浅浅（点赞：1）
96. 东东（点赞：1）
97. 万水千山（点赞：2）
98. 老饕（点赞：1）
99. Luc、、（点赞：2）
100. 黄河大侠（点赞：2）
101. 、、瑞、、（点赞：1）
102. 行者terry (（）
103. 风雨侠
104. 飞向远方
105. 67L
106. 小师太走江湖
107. 只要简单爱
108. 张小游
109. Memory
110. Mr

凤凰山革命旧址,

1. 归雁
2. 演刚（2019-2-4）、（点赞：34 评论：2 喜欢：5 观看;686）
3. 一阵眩晕（点赞：6 喜欢：1）
4. 背景老刘的足迹（2019-6-23）、（点赞：5 评论：7 喜欢：2 观看：462）、
5. Shirley （点赞;19 喜欢 :1）
6. 、、爱甜点（2018-9-21）、（点赞：22 评论：2 喜欢：12观看：1190）、（南泥湾——壶口瀑布——会峰寨——乾坤湾）
7. 客旅的
8. 334969、、（当中;2）
9. 猪爸（2020-9-22）、（点赞：13 评论：1 ）、（凤凰山——宝塔山——梁家河村知青旧址）
10. 小有与大（点赞：38 评论：6 喜欢：5 观看：596）、
11. 黑陶、、
12. Drizzle 、、（**攻略**）（2020-4-23）、（点赞：137 评论：45 喜欢：27 观看：5785）、
13. 庐江周公瑾
14. 半日闲（点赞：15）
15. 乐桃桃（点赞：12）
16. 笑笑
17. 天涯到处游（点赞：1）
18. 马蜂窝、、（点赞：13）
19. 鱼骨（点赞：2）
20. 苹果（实物）
21. 伯文（点赞：10）
22. 小小、、（点赞：10）
23. 飞扬（点赞：4）
24. 乡下人（点赞；3）
25. 飞扬（点赞：2）
26. 强子（点赞;10）
27. Waiker
28. 悠然在线
29. 乘风破浪的姐姐（点赞：15）
30. 竹影（点赞：3）
31. 觉悟大叔（点赞：1）
32. 小有与大有（点赞：1）
33. 、瑞、、（
34. 陕北波浪谷（点赞2）
35. 尸体的眼泪（）
36. 飞、、1025

王家坪革命旧址

1. 侍其孤独行（点赞：40 评论：10 喜欢：1 ）
2. 陕西中青旅、（点赞：13 评论；179 喜欢：1887观看531528）、（**攻略**）
3. Gimoon （2018-9-29）、（点赞：370 评论：75 喜欢：118 观看：4432）、
4. 晨光苏醒（点赞：46 评论：1 喜欢：2）
5. 马蜂窝**攻略**组|（观看：7840）
6. 、（向下的符号）、（点赞：9 喜欢：4观看：87）、
7. 飘摇|（点赞：36）
8. 马蜂窝**攻略**组|（观看：8899）
9. 大鲁的爸爸（2021-7-22）、（点赞：16）
10. 大鲁的爸爸（2021-7-22）、（点赞：17 评论：2）
11. 峡谷小庄（点赞：48 欧伦：2）
12. 炎冰（2021-7-21）、（点赞：166 评论：2 喜欢：16 观看;1334）、（延安革命纪念馆——王家坪——杨家岭——枣园——梁家河村）
13. 幽岩灰蹄、（点赞：27 评论：9 喜欢：2 ）
14. 倩先森（2018-7-22）、（点赞：84 评论：2 喜欢：55 观看;2529）、（南泥湾——清凉山——王家坪——枣园——1938文化美食节——延安剧院）
15. 小老兵（点赞：16）
16. 知秋一叶（2018-9-2）、（点赞：34 评论：1 喜欢：19 观看：1263）、（延安新闻纪念馆——枣园——延安1938文化街区——延安知青博物馆——杨家岭）、（延安革命纪念馆——王家坪——鲁迅文艺文学院旧址）
17. 紫涵凝月、（点赞：11 评论：2 观看：4）
18. 金哥铁马（点赞：6 评论：1 喜欢：2）
19. 多多益善（点赞：16 评论：5 喜欢：1）
20. 一玮（2016-8-11）、（点赞：24 评论：1 喜欢：15 观看：897）、（延安革命纪念馆——王家坪——延安.1938——枣园）
21. 雪菲（点赞：10 评论：4）
22. 麻糖（斗地主：15）
23. Tommylion88（点赞：5 评论：2）
24. Sweet-小不点（2016-9-14）、（点赞：761 评论：77 喜欢：201 观看11924）、（延安革命纪念馆——王家坪——1938——枣园）
25. 凌子姐姐（点赞：5）
26. Moonlight （2015-8-28）、（点赞：2000 评论：156 喜欢：1100观看：57705）、（清凉山——宝塔山——枣园——延安革命纪念馆）、（杨家岭——王家坪）
27. 墨迹小阿龟（点赞;1）
28. 林可爱、（2017-10-28）、（点赞：67 评论：4 喜欢：9 观看;1324）、（王家坪——枣园——圣地大剧院——钟楼）
29. 双玮、（点赞）
30. 阿牛（点赞：5）
31. 智者游天下（点赞：1）
32. 起司兔（2019-11-30）、（点赞：22 喜欢：6 观看：435）、（枣园——延安大学——杨家岭革命旧址——延安革命纪念馆——王家坪——宝塔山）
33. 疆域拓土、（2018-11-3）、
34. 硕果（2017-7-1）、（点赞：12 喜欢：6 观看：850）
35. 英子、（点赞：5）
36. Eric zhp （点赞：1）
37. 胡迪（2018-10-25）、（点赞：729 评论：49 喜欢：317观看：8505）、（甘泉大峡谷——枣园——杨家岭——王家坪）
38. 岩松（点赞：1）
39. 最讨厌昵称、、（点赞：1 评论：5 观看：1742）、（宝塔山——王家坪——枣园）
40. 大魔鬼）点赞;2
41. 唐媛（点赞：1）
42. 跟着老公去旅行（）
43. 享旅（点赞：1）
44. 菜中菜（2015-5-9）、（点赞：17 喜欢：3 观看：310）、（宝塔山——延安新闻纪念馆——延安革命纪念馆——杨家岭——枣园——靖边龙洲丹霞）
45. 猕猴桃
46. 游游、（2017-1-19）、（点赞;145 评论：4 喜欢：5 观看：2028）、（回民街——鼓楼——大雁塔——黄帝陵——壶口瀑布——南泥湾——八宝山——王家坪——枣园——钟楼）
47.
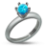
（点赞：17）
48. 玮哥
49. 柠檬色的琴声（2012-10-2）、（点赞：25 评论：10 喜欢：13 观看：2617）
50. 米多多
51. 透明雨（2019-11-15）、（点赞：147 评论：1 喜欢：5 观看：780）、（南泥湾——王家坪——枣园）
52. 小有与大有（点赞：26 评论：18 喜欢：5 观看：1345）
53. 老马的家常（点赞：3）
54. Id80
55. 小有与大有（点赞：34 评论：1 喜欢：3 观看：318）
56. Chengxhinghoo （点赞：1）
57. 爱深沉、（2017-1-8）、（点赞：25 评论：3 喜欢：4）
58. Zhouyujin
59. Ripchan（2016-10-1）、（点赞：117 评论：20 喜欢：17 观看：1924）
60. Gimoon （点赞;4）
61. 天崖为客（点赞：1）
62. 礼天下（2015-9-10）、（点赞：10 评论：13 喜欢：3 观看：1485）
63. 光之翼（点赞：1）
64. 、rebecca、（点赞：6 评论：2）
65. Chenxi3676 （点赞：6）
66. 潘涛先生（点赞：77 评论：2 喜欢：17 观看：138）、（延安革命纪念馆——鲁迅艺术学院旧址）
67. 菜中菜（点赞：1）
68. 清和
69. 阿牛是老虎（点赞：3）
70. Gemini-jasmine （2017-12-23）、（点赞：5 喜欢：2 观看：194）
71. 超
72. 李品忠（2020-10-26）、（点赞：7 喜欢：2 观看：164）、（雨岔大峡谷——枣园——杨家岭——王家坪）
73. 南飞雁、（点赞;2）
74. 经纬线（2020-11-19）、（点赞;32 喜欢：4 观看：601）
75. Zhzhzhuang（点赞：1）
76. 13704039615、
77. 坚冰
78. 衷寒、
79. 亮剑
80. 还是没人理（2019-10-15）、（点赞：28 评论：30 喜欢：6 观看：515）
81. 慕师兄（点赞：1）
82. 不断前行的蜗牛（2019-1-28）、（点赞：26 评论：2 喜欢：8 观看：633）、（杨家岭——延安革命纪念馆——王家坪——宝塔山）
83. Lilun（点赞：2 评论：1）
84. 想看世界的穷小子（2019-3-17）、（点赞：147 评论：1 喜欢：114 观看;5362）、（王家坪——枣园——延安大剧院）
85. Nic-law （点赞：5 喜欢：1）
86. 三秦小子（点赞：4）
87. 春行夏随、（2020-9-30）、（点赞:12 pl :1 喜欢：7 观看：177）、（小河会议旧址——杨家岭——枣园——宝塔山）、（王家坪——延安革命纪念馆——甘泉大峡谷）
88. 马蜂窝用户
89. Mr.clown （点赞：2）
90. Mm (dz :1
91. 猫耳朵（点赞：1）
92. Mr.clown (（点赞：12）
93. Mr.clown (（点赞：12）
94. 啫喱jelly （2017-7-12）、（点赞：113 评论：7 喜欢：39 观看：4449）、
95. 秦1统（）
96. fs阿简（2021-5-15）、（点赞：8 喜欢：3 观看：113）、
97. 黑灯
98. HTY
99. 姝姝（评论：1）
100. 老张投（点赞：3）
101. 延安西安顺丰（点赞：1）
102. Ameliaon 、、（点赞：1）
103. 飞扬（点赞：2）
104. Brolin（点赞：1）
105. 珂子（2017-8-4）、（点赞：46 评论：10 喜欢：12 观看：2596）、（宝塔山——延安革命纪念馆——王家坪——桃林公园——枣园）
106. 碟妖梦儿（点赞：1）
107. 二宝他妈、（点赞：12）
108. 京城蓝调（点赞：12 评论：2）
109. 鱼儿水中游（）
110. 行吗（2017-9-3）、（点赞：39 评论：2 喜欢：10 观看;678）、（王家坪——枣园）
111. 山竹
112. 雨里蒹葭（2019-8-25）、（点赞：53 评论：4 喜欢：16 观看：4471）、（波浪谷——延安革命纪念馆——王家坪——清凉山——杨家岭）
113. 菜菜的后花园、（点赞：51 喜欢：23 ）
114. Hpopesky （2013-6-10）、（点赞：17 评论;7 观看：3371）、（延安革命纪念馆——王家坪）
115. 马蜂窝用户苍蝇（点赞：4）
116. 砖儿姐、、（2018-9-30）、（点赞：10 评论：4 喜欢：5 观看：634）、（延安革命纪念馆——王家坪——南泥湾）
117. 三秦小子（点赞：4）
118. Yanzi （点赞：3）
119. 小丽丽、小胡胡（2018-2-23）、（点赞：9 喜欢：4 观看：298）、
120. 马蜂窝（点赞：3）
121. 猪猪侠（点赞：4）
122. 泡泡、（2017-5-23）、（点赞：56 评论：40 喜欢：13 观看：1505）、（宝塔山——枣园——杨家岭——王家坪）
123. 三秦小子（点赞：3）、
124. 仁者乐山（2019-5-1）、（点赞：20 喜欢：5 观看：521）
125. 春天哥歌（点赞：2）
126. 三秦小子（点赞：2）
127. 慕容彧、（2015-9-26）、（点赞：222 评论：6 喜欢：19 观看：5398）、（枣园——杨家岭——延安革命纪念馆——王家坪）
128. 老船长（点赞：1）
129. 三秦小子（点赞：2）
130. 远方、（2015-7-18）、（点赞;802 xih :2 gk :64）、
131. 旅行.与天地语（点赞：1）
132. 鱼大花（点赞：1）
133. 炎冰（点赞：1）
134. 小有与大有（2018-6-5）、（点赞：59 评论：9 喜欢：8 观看：641）、（延安革命纪念馆——王家坪）、（杨家岭——枣园）、（抗大——凤凰山）、（西北局——宝塔山）
135. 程序人生
136. 、雯奕
137. Rena
138. 旅行.与天地语
139. 、雯奕
140. 糊涂峰
141. 冰雪
142. 花、、鼠
143. Zyw0421
144. 沈苏言（2019-10-2）
145. 摄之旅（2020-4-7）（点赞：53 评论：18 喜欢：6 观看：1075）（王家坪——杨家岭——延安革命纪念馆——枣园——延安新闻纪念馆——清凉山——宝塔山）

杨家岭革命旧址,

1. abcd5059、（点赞：59 喜欢：2）
2. 陕西文旅（攻略）、（点赞：12 评论：2 喜欢：102 观看：18156）
3. 木兰、（点赞：66 评论：13 喜欢：4 ）
4. 马蜂窝攻略组、（观看：8905）
5. 马蜂窝攻略组、（观看：7848）
6. 月亮湾（点赞：25 喜欢：5）
7. Marry （点赞：11 评论：3）
8. 陕西文旅（官方）、（点赞：141 评论：21 喜欢：581）
9. 雪菲（点赞：28 评论：2 喜欢：1）
10. 陕北旅游私人订制（攻略）、（点赞：195 评论：2 喜欢：156 观看：12386）
11. 小有与大有（点赞：43 评论：1 喜欢：7 观看：1472）
12. 拜山之家、（点赞：47）
13. 哄哄、（2018-10-9）、（点赞：54 评论：1 喜欢：2）
14. Xiaoshao520（点赞：19 喜欢：1）
15. 知秋一叶、（2018-9-2）、（点赞：34 评论：1 喜欢：19）、（新闻纪念馆——枣园——1938——延安知青博物馆——杨家岭）、（延安革命纪念馆——王家坪——鲁迅文艺文学院旧址）
16. 玮哥（2017-5-11）、（点赞：9 喜欢：3 观看：683）、（杨家岭——枣园——王家坪——延安革命纪念馆——宝塔山）
17. 金色海洋（点赞：28 评论：8）
18. 蜜豆去旅行（2019-4-30）、（点赞：5 喜欢：2 观看：123）、（壶口瀑布——延安革命纪念馆 ——杨家岭）
19. 全顺、（2021-10-16）（点赞：40）
20. 燕子、（点赞：14）
21. 多忘（点赞：10 评论：10）
22. 月浪月花（2020-8-4）、（观看：80）
23. 新三十六技（点赞：10）
24. 北风糍粑（2017-2-17）、（点赞：39 评论：4 喜欢：29）、（延安革命纪念馆——杨家岭——）
25. 不断前行的蜗牛（点赞：12 喜欢1）
26. 大大大大骏（2017-9--22）、（点赞：25 喜欢：4 观看：782）、（杨家岭——枣园——南泥湾——壶口瀑布）
27. 碟儿、（点赞：6）
28. 禹禹独行（）
29. 桃桃（2017-11-26）、（点赞：26 评论：4 喜欢：11观看：560）、（延安革命纪念馆——杨家岭——保育院演出——枣园）
30. Scyhappy（点赞：1）
31. 阿皮大叔、（点赞3）
32. 周浩
33. 雨化田（点赞：5 评论：3）
34. 老友记（）
35. 咣当小肉
36. JACK Lee
37. 炜哥
38. 月轩越影（2020-9-5）、（点赞：46 喜欢：18 观看：510）、（壶口瀑布——杨家岭——枣园——宝塔山1938）
39. Miss金
40. 还是没人理（2019-10-16）、（点赞：27 评论：31 喜欢：6 观看：570）
41. 、、宇（点赞：1）
42. 偶尔而来（点赞：2）
43. 斗斗（2016—10-7）、（点赞：7 评论：2 喜欢：2 观看：1110）、
44. 马蜂窝用户（点赞：5）
45. 美丽的梦（2019-6-7）、（点赞：7 喜欢：5 观看：520）
46. 来了（）
47. 奕孜（）
48. 侍其孤独行（点赞 ：47 评论：24 喜欢：1）
49. Depthdiving
50. DONKEY （点赞：36 评论：8喜欢：1）
51. 王烨
52. Id80
53. Dora-p （点赞：3 评论2 xih :1 ）
54. 小有与大有 （点赞：22 评论：3 喜欢：4 观看：539）、
55. 某某某
56. 皓月肆蹄子公子
57. Zhouyu;in）
58. Chengxhiinghoo （点赞：1）
59. 小狼蹄子
60. Dudu (2011-6-1）、（点赞9 评论：12 喜欢：5 观看：2502）、
61. 小白、（2021-9-14）
62. 潮尚旅行（2020-7-11）、（点赞：19 喜欢：4观看;218）、（杨家岭——龙洲丹霞波浪谷）
63. 请给我一只板鸭（点赞：1）
64. 王小米粒子（点赞：2）
65. 游来游去的、
66. 城市小飞（点赞：6）
67. 勇者
68. 小有与大有（点赞：6）
69. 睡觉的企鹅（点赞：1）
70. Niukou2000*（点赞：1）
71. King d l (（点赞：13）
72. Zyw0421
73. Xiaoshao520（点赞：2）
74. 大手牵小手
75. 唐媛（点赞：1）
76. 阿牛（点赞：2）
77. 逐梦者小鹏（点赞：1）
78. 智者游天下（点赞：1）
79. 唐小杰
80. Zhzhzhuang （点赞：1）
81. 小白（2021-9-14）、和蔼可亲亲（点赞：5）
82. 光之翼（点赞：1）
83. 兔子罗恩（点赞：1）
84. 说走就走的旅行（点赞：1 评论：1 喜欢;1）
85. 老duck 看世界（点赞：15）
86. 东方（点赞：12 喜欢：1）
87. 丁浏宾（点赞2）
88. 菜中菜
89. Hdwhdong
90. Zoey
91. 大鲁的爸爸（点赞：3）
92. 菜菜的后花园（点赞：433 评论：4 喜欢：6）
93. 燕子（点赞：3）
94. 荒野（点赞：4 评论：1）
95. Berylzlf （点赞：3）
96. 马蜂窝用户（点赞：3
97. 炎冰（点赞：3）
98. 乘风破浪的姐姐（）
99. 鱼儿水中游
100. 方一
101. 奈叶
102. Zjs
103. 听泉品茶（点赞：3 喜欢：1）
104. 三位真火（点赞：3）
105. 高霞客（（2012-4）点赞15）
106. 亦行（点赞：4）
107. 随心而行（点赞：2）
108. 亦行（点赞：2）
109. 旅行.与天地语（点赞：1）
110. 宛如清风（点赞：1）
111. 、、宇（点赞：2）
112. 知足常乐
113. 出门旅游啦（收藏：1）
114. 阿、梅
115. 套不住的马
116. 老猫
117. 胆小鬼3371

枣园革命旧址

1. 海洋（点赞：43）
2. 符筱笃（点赞：19 喜欢：3）
3. 陕西中青旅（评论：13 点赞179 喜欢：1887观看：5315537）、
4. 小蜂在路上（攻略）、（点赞：412 评论：26 喜欢：3421、观看：178923）
5. 晨光苏醒（点赞：51 篇：6 喜欢：1）
6. Hulin （点赞：44 评论：4 喜欢：1）
7. 平淡生活（点赞：14）
8. 马蜂窝用户（点赞：6）
9. 恋恋四季（点赞：8 喜欢：3）
10. Xioashao520（点赞：3）
11. 双维（点赞：4）
12. 燕子
13. 唐媛（点赞：2 评论：1）
14. Xiaoshao520（点赞：2）
15. Xiaoshao520（点赞：1）
16. 混世大魔王（点赞：4 评论：2）
17. 不简单的小情歌（点赞：1）
18. 风筝与线
19. Tsai
20. 唐小杰（点赞：1）
21. 智者游天下（点赞：1）
22. Miss 金
23. 炜哥
24. 慵懒的大猫（点赞：2）
25. JACK Lee
26. 冬日里的（点赞：42）
27. 小有与大有（点赞：39 评论：1 喜欢：4 观看；1857）
28. 新三十六技（点赞：5）
29. 菜中菜（2015-5-9）、（点赞;1 ）
30. 麻糖
31. 王烨
32. 一个小朋友（点赞：2）
33. Zhouyujin
34. 也想玩玩（点赞：1）
35. Zhzhzhuang （点赞;1）
36. 关关
37. 三味真火（点赞;16 xih :2）
38. 花脸（点赞：54 评论：22 喜欢：14）
39. 睡觉的企鹅（点赞：1）
40. 迟小胖（点赞：1）
41. 137040439、、9dz :1
42. 小白（2021-9-14）、（点赞;2 pl :1）
43. 还是没人理（2019-10-16）、（点赞：29 评论：28 喜欢：7观看：603）
44. 小白（2021-9-14）、（点赞：2）
45. 小白（2021-9-14）、（点赞：1）
46. 老公、、
47. 光之翼（点赞：1）
48. 喜多（点赞：1）
49. 张飞
50. 029云
51. 小熊旺旺（2019-8-10）、（点赞：12 喜欢：6 观看：750）、（雨岔大峡谷——枣园）
52. 北
53. 温哥（点赞：4）
54. 大帅（点赞;1）
55. 一见腊梅（）
56. 阿牛是老虎（点赞：3）
57. 太阳花（点赞：17）
58. 马蜂窝用户（点赞：15）
59. Map 、、（当中：1）
60. 华仔（点赞：2）
61. Season （点赞：1）
62. 甜甜（）
63. 上帝之城（点赞：12）
64. 陕北波浪谷小王（点赞：3）
65. 在路上（点赞：5）
66. 赛旅（点赞：1）
67. 行走的一股清流（点赞：1）
68. 邯郸学步（点赞：2）
69. 九磅十五便是（点赞：2）
70. 菜菜的后花园（点赞：27 评论：1）
71. 柳絮儿飞（点赞：6）
72. 京城蓝调（点赞：9 评论：8）
73. Joe（点赞：3）
74. 高霞客（2012-4）、（点赞：16）
75. Sunsss（点赞：4 评论：5 喜欢：2）
76. 浙、、小乌龟（点赞：4）
77. 晨光苏醒（点赞：4）
78. 京华色影（点赞：1 喜欢：1）
79. 五洋（点赞：3）
80. 亦行（点赞：4）
81. 旅行.与天地、（点赞：4）
82. 大手牵小手（点赞：1）
83. My way （2019-8-17）（点赞：64 喜欢：2 评论：11）
84. Ouyili（2019-2-9）、（点赞：22 喜欢：9 观看：500）、
85. My way （2019-8-17）（点赞：38喜欢：1 评论：7）
86. 天予自在（点赞：1）
87. 天予自在（点赞：2）
88. 旅行.与天地（点赞：2）
89. 马蜂窝用户（点赞：1）
90. 甘泉雨岔峡谷、、（点赞：1）
91. 炎晴（2020-10-2）、（点赞：21 评论：3 喜欢：9 观看：992）、（南泥湾——壶口瀑布——知青）、（清凉山——延安新闻纪念馆——宝塔山）、（延安科技馆——枣园）
92. 老duck 看世界（点赞：14 喜欢：1）
93. 百里奚（2016-7-24）、（点赞：）
94. 、zhilavie
95. 简单-
96. 沐溪浅浅
97. 泡泡
98. 瑜见、、
99. Merafour
100. 、、雯奕
101. 、雯奕
102. 加班喵要干活
103. 马蜂窝洪湖
104. Bounty

延安革命纪念馆

1. 海洋（点赞:60）
2. 陕西中青旅（攻略）（点赞：179 评论：13 喜欢：1887 观看：531541）
3. Charles （点赞：18）
4. 小峰在路上、（点赞：412 评论：26 喜欢：3420 观看：178926）、
5. lawyer qi（2019-12-18）、（点赞：11 喜欢：7 观看：2506）
6. 侍其孤独行（点赞：63 评论：31 喜欢：1）
7. 锄头（点赞：16 评论：1 喜欢：5）
8. 智者游天下（点赞：1）
9. 击浪飞歌（点赞：32 评论：18）
10. 喵大仙（2019-4-30）、（点赞：375 评论：1 喜欢：19 观看：1657）、（宝塔山——清凉山——延安革命纪念馆——杨家岭）
11. 东方（点赞：13 评论：2）
12. 行者terry（点赞：22 喜欢：2）
13. 、三箱、、（点赞：12 评论：1）
14. Tommylion888 （点赞：7 评论：1）
15. 金戈铁马（点赞：6）
16. ASSEN（2018-10-1）、（点赞：25 评论4 xih :4 gk :425）
17. 骑士（点赞：17）
18. 农夫（点赞：8 观看：494）、
19. Shenhuan（点赞：8）
20. 笑看风云（2020-7-19）、（点赞：13 喜欢：4 观看：695）
21. 刘欢欢（点赞：10 喜欢：6 ）
22. 农夫、（点赞：16 喜欢：2 观看：198）
23. 、、园（点赞：7）
24. Yolo （点赞：12 评论1xih :5 gk :500）\
25. 事在人为（点赞：8）
26. 小有与大有（点赞：50 评论：4 喜欢：14 观看：2064）
27. 风从东方来（点赞：8）
28. Yuanr （点赞：21 喜欢：2）
29. 丑哥（2019-4-22）、（点赞：19 喜欢：8 观看：2054）、
30. 符号号（点赞：4 喜欢：3）
31. 燕子（）
32. 绝对疯了（点赞2 pl :10 xih :1gk :1407）
33. 何俊辰（点赞4）
34. Ripchan （2016-10-1）、（点赞：117 评论：20 喜欢：17 观看：1926）、
35. 兔子—fiona （点赞：2）
36. 天与地（点赞：5）
37. 嘎啦耍
38. 周州（点赞：3）
39. 还是没人理（点赞：29 评论：35 喜欢：12 观看：992）、
40. 炜哥
41. 也想玩玩（点赞：1）
42. 、天涯
43. 小、、（2021-7-26）、（点赞：8 喜欢：1 观看：1409）
44. 新三十六技（点赞：4）
45. 农夫（点赞：7 喜欢：2观看：2193）
46. 菜中菜（2015-5-9）（点赞：1）
47. 延安旅游包车（点赞：15 喜欢：2）
48. 、、谢泡泡、（点赞：3）
49. 大梅明雪（点赞：2）
50. 雅伦的旅行（点赞：14 喜欢：9 观看：1102）、
51. 无昵称（点赞：1）
52. 行者无疆（2018-9-17）、（点赞17 xih :7 gk :675）
53. 崔（三金）、、（2018-6-14）、（点赞：17）
54. Zhouyujin
55. 天与地（2019-10-6）、（点赞：10 评论：1喜欢：3 观看：141）、
56. 岁月是把杀猪刀
57. Feng
58. Ashlyn
59. 农夫（点赞：7 喜欢：2 观看：590）
60. 王剑招（点赞：3）
61. 神奇在路上（点赞：61 喜欢：5 评论：2）
62. 农夫（点赞：8 观看：2557）
63. 春天的菠菜（点赞：14 评论：1）
64. 小胡（点赞：3）
65. 强子（）
66. 泉仔（2020-8-4）、（观看：23）、
67. 小北（点赞：1）
68. 珞瑜
69. 缘来（点赞：2 评论：1）
70. 凤舞香罗（点赞：16 喜欢：9观看：1000）、（路遥墓——宝塔山——王家坪——延安革命纪念馆——杨家岭）
71. 我曾经就在这里（点赞：）
72. 博博走天下（点赞：11 评论：2 喜欢：7 观看：444）、
73. 小浣（点赞：）
74. 上善若水（点赞：1）
75. Zhzhzhuang
76. 小白（2019-9-15）、（点赞：2评论：1）
77. 小白（2019-9-15）、（点赞：1）
78. 其其（点赞：6 喜欢：3 观看：13）
79. 小白（2019-9-15）、（点赞：1）
80. 小白（2019-9-15）、（点赞：2）
81. 小白（2019-9-15）、（点赞：1）
82. 小白（2019-9-15）、（点赞：1）
83. 小白（2019-9-15）、（点赞：1）
84. 小白（2019-9-15）、（点赞：1）
85. 小白（2019-9-15）、（点赞：1）
86. 小白（2019-9-15）、（点赞：2）
87. 小白（2019-9-15）、（点赞：2）
88. 小白（2019-9-15）、（点赞：1）
89. 小白（2019-9-15）、（点赞：3）
90. 小白（2019-9-15）、
91. Chengxyang
92. 关关
93. 智慧
94. 捏泥巴的二毛
95. 瞌睡虫（点赞：5）
96. 甜点（点赞：4）
97. 君梓平（点赞：4）
98. 静（点赞：1）
99. 睡觉的企鹅（点赞：1）
100. 竹雨冰心（点赞：2）
101. L.
102. Chengxihighoo（点赞：1）
103. Coco
104. 大纯子
105. 吴小特（点赞：1）
106. 竹雨冰心（点赞：1）
107. 晴天娃娃（2012-10-4）、（点赞：2 观看：2919）、（枣园——杨家岭——延安革命纪念馆）（清凉山——宝塔山）
108. 寒星魂（点赞：1）
109. 套不住的马（点赞：1）
110. 向往极光（点赞：2）
111. Lady q （）
112. 行者terry
113. 爱旅行的biil（点赞：5）
114. 丁香、（点赞：3）
115. 大鲁的爸爸（点赞：3）
116. 大鲁的爸爸（点赞：1）
117. 乐逍遥（点赞：1）
118. 行动学习者（点赞：1）
119. 东方（）
120. 大手牵小手
121. Treating
122. 雪菲（点赞;25 pl :2xih :2）
123. 云朵的旅行日记（点赞：60评论：3 喜欢：26）
124. 高霞客（点赞：17）
125. 百里奚
