# Supplementary material for: The network characteristics of classic red tourist attractions in Shaanxi province, China
Source: PLoS One. 2024 Mar 29;19(3):e0299286. doi: 10.1371/journal.pone.0299286 (PMC10980247; doi:10.1371/journal.pone.0299286)
Supplement: S4 File — (DOCX) [file pone.0299286.s006.docx]

“西安事变”纪念馆

1. 小涂学长、（点赞：184）
2. here查无此人（点赞：13）
3. 这里是长安（点赞：23）
4. 这里是长安（点赞：26）
5. DD是个好孩子（攻略）（点赞：1108 喜欢：1809）
6. 奔跑的大粗腿（攻略）（点赞：155 喜欢68 评论：6）
7. 西安旅游规划师——小周、（攻略）（点赞：127喜欢：40 评论：2）
8. 西安旅游规划师——小周、（攻略）（点赞：35喜欢：16 评论：1）
9. 追风的耿耿（攻略）（点赞：210 喜欢：8 ）
10. Hthr2551（攻略）、（点赞：160 喜欢：6 评论：12）
11. 影客儿（点赞：148）
12. 风行十载（点赞：2 喜欢：2）（回民街——古城墙——西安事变纪念馆——珍妮花园咖啡馆——赛格国际购物中心）
13. Magic-ace （攻略）（点赞：12）
14. 蓝若辰（攻略）、（点赞：12）
15. 戴帽子的鱼儿（攻略）、（点赞：3 评论：3）
16. 饭桶大哥（攻略）（点赞：17）
17. Doris.c（攻略）（点赞：10）
18. 悲剧的一切（西安事变纪念馆——城隍庙）、（点赞：16 评论：2）
19. 郭佳林（攻略）、（点赞：12 评论：2）
20. 羊羊撒撒（攻略）、（点赞：20）
21. 司洁洁（攻略）、（点赞：15）
22. 张宇鹏（陕西历史博物馆——大唐芙蓉园——西安事变纪念馆——江城小馆——回民街）
23. Fendi （2021-4-28）（点赞：17 评论：1）、（大明宫国家遗址公园——西安事变纪念馆——大兴善寺——曲江池）
24. 恩和、、（攻略）（点赞：125 喜欢：2 评论：6）
25. 猫头鹰（攻略）、（点赞;144 喜欢：5）
26. Bnb-1000667044（攻略）、（点赞：51 喜欢：47）
27. 爱伦（点赞：20 喜欢：2 评论：15）（）
28. DD是个好孩子（攻略）（点赞：166 喜欢：180评论：9）
29. 恩和、、（攻略）（点赞：12）
30. 周天旭（攻略）、（点赞：12）
31. 小爽（攻略）、（点赞：11）
32. 程小柚（攻略）、（评论：2）
33. 有你亦安然（攻略）、（点赞：11）
34. 栗子家的二锅头（攻略）、（点赞：10喜欢：1）
35. Teatea（点赞：13 ）、（书院门——西安事变纪念馆——鼓楼——回民街——洒金桥）
36. 这里是长安（点赞：21 喜欢：3 评论：5）
37. 陆佃（攻略）、（点赞：16 评论：1）
38. 这里是长安（点赞：22 喜欢：1 评论：4）
39. 这里是长安（点赞：34 评论：6）

2.汉中市川陕革命根据地纪念馆

1、ashley（点赞：14 喜欢：4）、（古汉台——川陕革命根据地纪念馆）

2、破损的战衣（点赞：17 喜欢：7 评论：3）、（）

咸阳市泾阳县安吴青训班革命旧址

1. 夜晨life（点赞：1）
2. 皮县豆瓣酱（点赞：12）

汉中市洋县华阳红二十五军司令部旧址

1. 追风的耿耿（攻略）（点赞：126 喜欢：6 评论：10）

南泥湾革命旧址

1. 慕君威（攻略）、（点赞：16）
2. 倾听雨落（点赞：19）
3. Kiritoeugeo （点赞：13）
4. 感知、（（点赞：21 喜欢：1）
5. 埃塞克斯（点赞：21 喜欢：1 评论：5）
6. 蓝色玻璃瓶（）
7. 想成为大触（点赞：18 ）
8. Clean （点赞：18）
9. 徐晓（点赞：21 喜欢：1）、（猫巷大峡谷——金延安——南泥湾）

桥儿沟革命旧址,

1、

宝塔山景区,

1. 青岛（点赞：1 喜欢：2 评论：3）
2. 想成为大触（点赞：25 喜欢：2）、
3. Trtata（点赞：16 喜欢：1）
4. 苏风-v（点赞：13 喜欢：1）、
5. 老鬼（点赞：13）
6. 孙米-（点赞：12 喜欢：1）
7. 尤武图斯（2012-4）（点赞：30 喜欢：1评论：2）
8. 夜晨life（点赞：18）
9. 玩世不恭（点赞：19 喜欢：1）、
10. 周扬fly （点赞：16）
11. 黑谷量子（点赞：13 喜欢：2）
12. 电量未满（点赞：14 喜欢：1）
13. 神奇在路上（攻略）（点赞：23 喜欢：9）
14. 健康就好（2019-10-13）（点赞：22）
15. 北极极光（点赞：17）
16. 我的个凡呐2333
17. 魉、、（点赞：20）
18. 墨茄（点赞：16）
19. 阿赖、、智慧（点赞：12 喜欢：1）
20. 850988（2016-4）、（点赞：17）
21. 阿榕君（点赞：18）
22. 咯啬（点赞：32）
23. 倔驴（点赞：22 喜欢：5）
24. 铂睿、、投（点赞：18 喜欢;1）
25. 云游中国（点赞：34 评论：5）、
26. 穿短裤的008（点赞：16）
27. 在清风李（点赞：22 喜欢：1）
28. 去哪儿用户（攻略）、（点赞：288 喜欢：7 评论：10）
29. 大米（攻略）、（点赞：27 喜欢：6）
30. 叠一叠浪一浪（点赞：45 喜欢：38 评论：12）
31. 暖阳（攻略）、（点赞：119 喜欢：2 评论：8）
32. 壳壳（点赞：131 喜欢：5 评论：5）
33. 北山（攻略）、（点赞：24 喜欢：40 评论：7）
34. 陕北旅游订制（攻略）、（点赞：122 喜欢：2 评论：6）
35. 之乎者也（点赞：18 喜欢：1）、（）
36. 烂烂的布头（点赞：33 喜欢：2 评论：3）
37. 卖梦旅人攻略（攻略）、（点赞：171 喜欢：22）
38. Ihzv20143 (（点赞：144 喜欢：1 评论：6）
39. 佛系青年DRAGON、（点赞：21 喜欢：2）
40. 卓卓（点赞：17）
41. 周12345、（攻略）、（点赞：44 喜欢：2 评论：4）
42. 玉置浩二、（攻略）、（点赞：1）
43. 游记、（攻略）、（点赞：245 评论：12）
44. 陕北旅游订制（攻略）、（点赞：16 喜欢：2 评论：4）
45. 通辽太史、、（攻略）、（点赞：16 喜欢：4 评论：2）
46. 瞳宝妈、（点赞：120 评论：6）（枣园——延安科技馆——杨家岭）、（杜公祠——西北局）
47. 言声（点赞：19）、（姜氏庄园——延安革命纪念馆——宝塔山）
48. 浮生几何（点赞：12）、（清凉山——宝塔山）
49. 唯手熟尔（攻略）、（点赞：13 评论;13）
50. 唯手熟尔（攻略）、（点赞：19）
51. 港岛妹夫（攻略）、（点赞：12）
52. 绝灵（点赞：20）
53. m黑桃皇牌m
54. 、、方方（点赞：24 评论：1）
55. 忽悠（攻略）、(（喜欢：2 评论：1）
56. 佛系青年、、（点赞：48 评论：6）
57. 茶如女人（点赞：11）
58. Lindainuk （点赞：14

清凉山革命旧址,

1. 独自随风（点赞：19）
2. 思久为远见（点赞：40 喜欢：3 评论：5）

凤凰山革命旧址

1. 昵称一定要酷（点赞：24 评论：1

王家坪革命旧址,

1. 卖梦旅人**攻略**、（点赞：171 喜欢：22）
2. 一条哈士奇（点赞：3）、（攻略）
3. 小红不想吃鱼（攻略）、（在：24）

杨家岭革命旧址,

1. fenton (（点赞：14）
2. 李违抗（点赞：20）
3. 123321（点赞：18 评论：1）
4. 甘泉大峡谷驿站、、小张（攻略）、（点赞：113 喜欢：4 评论：16）
5. 龙门鱼（攻略）、（点赞：18）
6. 佛系玩家（攻略）、（点赞：10）

枣园革命旧址

1. 周12345（攻略）、（点赞：44 喜欢：2 评论：4）
2. 看我介绍（）（点赞：16 喜欢：16）

延安革命纪念馆

1. 神奇在路上（点赞：35 喜欢：10）
2. Nje8848（点赞：1 评论：1）
3. 栗子在路上（点赞：19）
4. 山西陕西之旅、（点赞：64）、（宝塔山——延安革命纪念馆——枣园
5. 周某、（点赞：15）
6. 文艺蜀黍看世界（点赞：150 喜欢;1）
7. 文艺蜀黍看世界（点赞：285 喜欢：2）
