# Supplementary material for: The network characteristics of classic red tourist attractions in Shaanxi province, China
Source: PLoS One. 2024 Mar 29;19(3):e0299286. doi: 10.1371/journal.pone.0299286 (PMC10980247; doi:10.1371/journal.pone.0299286)
Supplement: S5 File — (DOCX) [file pone.0299286.s007.docx]

1-西安市红色旅游系列景区(八路军西安办事处纪念馆,“西安事变”纪念馆)

八路军西安办事处纪念馆

1. 紧急通知、（阅读量：4980）
2. 税务青年学党史 坚定信念跟党走——走进八路军西安办事处（2882）
3. 公告（2107）
4. 走进八路军西安办事处纪念馆，感悟初心与使命（1796）
5. 八路军西安办事处纪念馆关于暂停开放的公告（ 1784）
6. 庆祝建党百年主题党日 | 参观八路军驻西安办事处（1547）
7. 【难忘中国之声—抗战记忆】八路军西安办事处（1304）
8. 忆往昔,重温“红色记忆”:走进八路军西安办事处（ 1221）
9. 伟大的女性——海伦•福斯特•斯诺（一），敢于冒险的美国青年（1113）
10. 百年恰是风华正茂 创业园企业这样庆祝建党百年华诞（ 1069）
11. “相会如有期，同向光明路”（981）
12. 八路军西安办事处纪念馆临时闭馆公告（976）
13. 夏休不停学丨八路军西安办事处纪念馆参观见学、（931）
14. 党建铸魂 | 我所组织参观八路军西安办事处纪念馆（904）
15. 开馆公告（848）
16. 周恩来与七贤庄二三事（837）
17. 大赛预告|全市大学生红色经典诵读比赛开始报名啦（827）
18. 党史学习教育｜追寻红色印记 讲好党史故事④走进八路军西安办事处纪念馆（812）
19. 告全市党组织和党员倡议书（ 793）
20. 总书记又参观了一个重要展览！（762）
21. 八路军西安办事处纪念馆举办纪念海伦·斯诺诞辰111周年活动（ 753）
22. 打卡家乡红色景点 | 第五站，八路军西安办事处纪念馆（698）
23. 决议全文来了！（632）
24. 别慌！收到“时空伴随者”提示短信这样做（ 631）
25. 八路军西安办事处纪念馆参观须知（ 619）
26. 八路军西安办事处纪念馆向社会征集历史类图书的公告（ 615）
27. 探密古城长安，领略秦汉雄风 树仁作文研学夏令营（ 604）
28. 学生活动丨我院组织开展参观八路军西安办事处纪念馆活动（ 579）
29. 学校举办参观八路军西安办事处纪念馆活动（ 578）
30. 企业占11个，研学基地有3个！2022年青少年教育基地名单公布！（566）
31. 参观八路军办事处西安纪念馆（ 563）
32. 八路军西安办事处纪念馆恢复开放公告（543）
33. 全文来了！（ 539）
34. 为什么要学党史，习近平总书记这样讲（ 531）
35. 习仲勋的家风（ 520）
36. 线上微课——“跟着文物学党史”（508）
37. 八路军西安办事处纪念馆国庆节参观须知（503）
38. Spring Trip | 不负春光，SPAS同学带你行（ 452）
39. 西安地铁指挥部组织党员参观八路军西安办事处纪念馆（452）
40. 一图速览二十大报告（441）
41. 探访红色基地，弘扬革命精神——市市场监管局团委组织团员干部参观八路军西安办事处纪念馆（ 437）
42. 新城区委组织部到八路军西安办事处纪念馆开展党史教育活动（425）
43. 长龙航空西北党小组前往八路军西安办事处纪念馆参观学习（410）
44. 党日活动 | 现场运行中心党委开展参观八路军西安办事处纪念馆党日活动（409）
45. 喜迎二十大｜逐梦新征程 八办在行动（401）
46. 【最新追踪报道】西安八路军办事处纪念馆璀璨夜——浓情中秋赏月活动（400）
47. 勿忘！勿忘！勿忘！（385）
48. 省公安厅交管局机关党委组织党员领导干部赴西安八路军办事处纪念馆开展主题党日活动（376）
49. 盛世中国 喜迎华诞（ 368）
50. 深秋时节，我们一起来八路军西安办事处纪念馆打卡（356）
51. 重温峥嵘岁月 共走新的长征——走进“八路军西安办事处纪念馆”（356）
52. 八路军西安办事处纪念馆举办纪念中国工合成立80周年暨乔治·何克诞辰103周年座谈会（ 347）
53. 八路军西安办事处纪念馆党支部开展“铭记党史 不忘初心”专题党课（347）
54. 八路军西安办事处纪念馆获赠一挺日式轻机枪（345）
55. 专栏｜致敬最可爱的人——抗美援朝老兵口述历史（一）（334）
56. 我院青马班参观八路军西安办事处纪念馆活动顺利举行（ 319）
57. 喜迎建党百年系列活动|电气工程学院学生赴八路军西安办事处纪念馆开展主题党日活动（ 316）
58. “八办”纪念馆2019年“5.18国际博物馆日”系列活动具体安排（306）
59. 【红色之旅】八路军西安办事处纪念馆（295）
60. 群豪党建丨参观八路军西安办事处纪念馆，开展党史学习教育（292）
61. 新展资讯 | 展览先导片＆亮点解读＆布展花絮（287）
62. 新闻联播丨习近平带领中共中央政治局常委瞻仰延安革命纪念地（278）
63. 【献礼百年】绿京华热烈庆祝中国共产党成立100周年（ 275）
64. 我院团学干部参观八路军西安办事处纪念馆（276）
65. 百年党史青年说|走进“八路军西安办事处纪念馆”（273）
66. 八路军西安办事处纪念馆中秋节参观须知（263
67. 陕西出台推进博物馆改革发展实施方案（263）
68. 跟着总书记长见识 | 三苏祠:传承诗文里的传统文化（262）
69. 八路军西安办事处纪念馆清明假期参观须知（259）
70. 二十大报告指出：传承中华优秀传统文化，激发全民族文化创新创造活力（255）
71. 不忘初心，砥砺前行|参观八路军西安办事处纪念馆（ 251）
72. 展讯 | “为和平而来—公谊救护队援华史实展”开幕！（ 248）
73. 七一，张溪镇各村（居）这样庆祝建党百年！（247）
74. 同心抗“疫” 八路军西安办事处纪念馆在行动（247）
75. 廉政文化大片——八路军西安办事处纪念馆廉政教育基地（ 242）
76. 铭记历史·勿忘国耻 “九·一八”91周年主题纪念活动（238）
77. 二十大报告这些话，振奋人心！（238）
78. 图片新闻丨我队青年职工参观八路军西安办事处纪念馆（ 232）
79. 用“心”读懂党的二十大报告（ 230）
80. 八路军西安办事处纪念馆国庆假期参观须知（229）
81. 相约八办，充实您的这个夏天（ 219）
82. Day4调研心得|参观八路军西安办事处纪念馆（ 218）
83. “万人同书中国梦”二十大主题全国书画大赛展（3）（ 219）
84. 【不忘初心 牢记使命】我处党委组织赴八路军西安办事处纪念馆开展“不忘初心、牢记使命”革命传统教育（214）
85. 参观八路军西安办事处纪念馆有感（ 213）
86. 电控学子三下乡||探访八路军西安办事处纪念馆（212）
87. 八路军西安办事处纪念馆全体党员集中学习西迁精神（211）
88. 八路军西安办事处纪念馆喜迎“雪弗莱”老爷车回家（ 210）
89. 八路军西安办事处纪念馆五一假期参观须知（209）
90. 八路军西安办事处的“红色体育精神”（206）
91. 八路军西安办事处纪念馆端午节参观须知（ 201）
92. 八路军西安办事处纪念馆国庆节假期正常开馆公告（201）
93. 八路军西安办事处纪念馆八一建军节正常开放公告（200）
94. 【身临其境】革命遗迹——八路军西安办事处纪念馆（199）
95. 大学生红色经典诵读比赛决赛在八路军西安办事处纪念馆举办（195）
96. 省委外办组织党员干部赴八路军西安办事处纪念馆进行爱国主义教育（189）
97. 打卡红色教育基地 学四史行走课堂 | 八路军西安办事处纪念馆（188）
98. 八路军西安办事处纪念馆召开秦岭北麓违建专项整治纪律整改讲评大会（185）
99. 八路军西安办事处纪念馆接受林伯渠孙女林友群捐赠文物（181）
100. 触摸“红色驿站”：走进八路军西安办事处纪念馆（179）
101. 烈士纪念日丨缅怀！铭记！致敬！（177）
102. 八路军西安办事处纪念馆 “十一”期间参观指南（176）
103. 民族团结进步月 | 学校举办参观八路军西安办事处纪念馆活动（169
104. 校园vlog｜三分钟带你走进八路军西安办事处纪念馆（ 166）
105. 我用初心守护你（166）
106. 羲和国学社参观八路军西安办事处纪念馆 | 奕帮推（158）
107. 八路军西安办事处纪念馆 开展“我们的节日——七夕”主题社教活动（156）
108. 学习 | 走进八路军西安办事处纪念馆（156）
109. 高文党建 | “七一”红色党史学习活动--参观八路军西安办事处纪念馆（155）
110. 八路军西安办事处纪念馆党支部荣获西安市“党员先锋岗”荣誉称号（153）
111. 八路军西安办事处纪念馆入选全国首批“大思政课”实践教学基地（153）
112. 八路军西安办事处纪念馆馆藏稀见西安地区革命文献举隅（152）
113. 八路军西安办事处纪念馆端午假期参观须知（ 150）
114. 八路军西安办事处纪念馆党支部开展“巾帼心向党 奋进新征程”庆祝“三八”国际妇女节系列活动（149
115. 红色景区万里行——八路军西安办事处纪念馆（下）（144）
116. 红色景区万里行——八路军西安办事处纪念馆（上）（138）
117. 忆峥嵘岁月·度传统佳节 “我们的节日——中秋”（134）
118. 八路军西安办事处纪念馆元旦参观指南（133）
119. “八办”纪念馆党支部赴西安碑林博物馆开展固定党日活动（131）
120. 陕西省红色旅游指南——八路军西安办事处纪念馆（ 130）
121. “八办”纪念馆党支部 七一慰问困难党员 传递关怀忆初心（129）
122. 用红色文化激扬城市精神 徐明非副市长调研八路军西安办事处纪念馆（126）
123. 八路军西安办事处纪念馆开展突发事件培训演练（126）
124. 安康铁路运输检察院党总支组织党员干警参观八路军西安办事处纪念馆（ 125）
125. 峥嵘岁月忆先烈·不忘初心跟党走（122）
126. 八路军西安办事处纪念馆应邀参加陕西省延安精神研究会宣讲活动（121）
127. 八路军西安办事处纪念馆9月3日正常开馆公告（119）
128. 关于文物保护和传承，总书记这样说（115）
129. 八路军西安办事处纪念馆中秋节开放公告（116）
130. 【走进“西办”】记八路军西安办事处纪念馆（113）
131. 线下实践 | 参观八路军西安办事处纪念馆（111）
132. 八路军西安办事处纪念馆联合西五路派出所开展“中国旅游日”宣传活动（106）
133. 新城区设立八路军西安办事处纪念馆党性教育基地（105）
134. “数”读二十大报告（104）
135. 团队动态 | 纳米光电化学与应用研究团队组织参观八路军西安办事处纪念馆（104）
136. 探寻红色 ——八路军西安办事处纪念馆（99）
137. 红色景区万里行——八路军西安办事处纪念馆（99）
138. 值得收藏 | 党的二十大报告学习笔记（99）
139. 八路军西安办事处纪念馆开展国庆节期间工作部署会（96）
140. 西安红色地标】八路军西安办事处纪念馆遗址群（94）
141. “八办”纪念馆党支部召开“讲政治、敢担当、改作风”组织生活会（90）
142. 八路军西安办事处纪念馆开展干部作风排查整改工作动员会（90）
143. 【微历史】走进八路军西安办事处（86）
144. 团日活动|游八路军西安办事处纪念馆（87）
145. 参观八路军西安办事处纪念馆（86）
146. 《五四纪念歌》的词作者冯文彬（85）
147. 【智慧旅游】八路军西安办事处纪念馆（79）
148. 文化和自然遗产日｜文物保护：时代共进 人民共享（79）
149. 八路军西安办事处纪念馆举办“勿忘国耻 圆梦中华”主题纪念活动（78）
150. 学习二十大 奋进新征程（十）（78）
151. 市统计局机关支部参观八路军西安办事处纪念馆（77）
152. 百花盛开，桃红柳绿，八路军西安办事处纪念馆开馆啦！（77）
153. 清风千年 | 立心立命的张载（76）
154. 八路军西安办事处纪念馆“优秀历史文化进校园”主题活动走进陕西学前师范学院（75）
155. 八路军西安办事处纪念馆参观讲解（75）
156. 八路军西安办事处纪念馆应邀参加“两个斯诺在中国”国际研讨会（73）
157. ★2月25日走进西安八路军办事处纪念馆活动精彩回顾（71）
158. 八路军西安办事处纪念馆受邀参加中国“工合”运动国际研讨会（69）
159. 红色印记 | 八路军西安办事处纪念馆 （第58期）（69）
160. 百个红色景点“云”参观㉘ | 八路军西安办事处纪念馆（69）
161. 八路军西安办事处纪念馆召开社教工作专题会（69）
162. 八路军西安办事处纪念馆 开展“送温暖、献爱心”活动（67）
163. 专栏｜致敬最可爱的人——抗美援朝老兵口述历史（二）（ 68）
164. 八路军办事处驻西安纪念馆（67）
165. 市残联组织参观八路军西安办事处纪念馆（66）
166. 人民日报 | 让更多文物和文化遗产活起来（64）
167. 陕西革命旧址云上展（十四）| 八路军西安办事处纪念馆（64）
168. 感悟峥嵘岁月 传承红色基因——基层人防组织建设培训班学员参观八路军西安办事处纪念馆（64）
169. 【社科基地巡礼】八路军西安办事处纪念馆（62）
170. 学习二十大 奋进新征程（八）（61）
171. 习近平的文化情怀（61）
172. 学习二十大 奋进新征程（九）（56）
173. 2022网安周 | 网络安全为人民 网络安全靠人民（55）
174. “实践学习四史，喜迎建党百年”第4期|八路军西安办事处纪念馆（53）
175. 红色景点丨七贤庄八路军西安办事处纪念馆（53）
176. 文物事业十年成就 | 赓续文明根脉 筑牢自信根基（52）
177. 八路军西安办事处纪念馆端午节特别活动（50）
178. 关注 | 土门街道班子成员到八路军西安办事处纪念馆参观学习（50）
179. 旧事视频：走进八路军西安办事处纪念馆（49）
180. 八路军西安办事处纪念馆举办 社会教育工作“微讲座”（49）
181. 保密公益宣传片《保密防线》上线（49）
182. 八路军西安办事处纪念馆 ——不熄的火种（49）
183. 学习二十大 奋进新征程（十一）（52）
184. 八路军西安办事处纪念馆开馆公告（48）
185. 读书，不只在今天……（46）
186. 红色基地：八路军西安办事处纪念馆（46）
187. 八路军西安办事处纪念馆中秋节假期正常开馆公告（45）
188. 八路军西安办事处纪念馆（上）——寻走历史的踪影（三十一）（41）
189. 【纪念馆巡礼】八路军西安办事处纪念馆（40）
190. 国家网络安全宣传周｜网络安全小课堂（39）
191. 社会实践|八路军西安办事处纪念馆（39）
192. 八路军西安办事处纪念馆进校园，与学生一起纪念“九·一八”（38）
193. 陕西红色地标（三）——八路军西安办事处纪念馆（37）
194. 八路军西安办事处纪念馆——西安免费景点（34）
195. 【美文星空】爷爷是个老红军（30）
196. 八路军西安办事处纪念馆举行小讲解员汇报讲解活动（30）
197. 【小知识】八路军西安办事处纪念馆（30）
198. 【教育基地】八路军西安办事处纪念馆（29）
199. “9.18"事变纪念日市民参观八路军西安办事处纪念馆（26）
200. 建党百年，100个红色旅游地推荐，沿着党的足迹重温红色记忆（25）
201. 【2022三下乡实践】八路军西安办事处纪念馆（24）
202. 红色故旅 | 八路军西安办事处纪念馆（23）
203. 每日名胜 | 陕西·八路军西安办事处纪念馆（23）
204. “云游”红色展厅-走进八路军西安办事处纪念馆（22）
205. 团日丨游八路军西安办事处纪念馆（19）
206. 游遍陕西——西安市（31）：八路军西安办事处纪念馆（19）
207. 【跟党走的一百年·团史故事100讲】 第七十九期:维护网络安全，共忆抗战70周年（17）
208. 【2022西安冬季】探秘古都历史，领略秦风唐韵，体验关中文化夏令营（5日）（2）
209. 红色展馆推介|八路军西安办事处纪念馆（14）
210. 八路军西安办事处纪念馆（10）
211. 西安八路军办事处纪念馆开放信息（10）
212. 八办“视”界 | 历史上的今天 —— 11月13日（9）
213. 区市容园林局组织党员干部参观八路军西安办事处纪念馆（9）
214. ￥3399/人『西安5日｜西安兵马俑、陕西历史博物馆、华山、明城墙、大唐芙蓉园、钟鼓楼、高家大院、回民街』（10）
215. 第七期 八路军西安办事处纪念馆（9）
216. 走进八路军西安办事处纪念馆（8）
217. 八办“视”界 | 历史上的今天 —— 11月14日（7）
218. 八办“视”界 | 历史上的今天 —— 11月15日（6）
219. 八办“视”界 | 历史上的今天 —— 11月16日（6）
220. 【2022西安冬季】探秘古都历史，领略秦风唐韵，体验关中文化夏令营（5日（2）
221. 市妇联党员干部参观八路军西安办事处纪念馆（85）
222. 周末，不妨去八路军西安办事处纪念馆转转（59）
223. 八路军西安办事处纪念馆讲解员招聘公告（24）
224. 八路军西安办事处纪念馆应邀参加“博物馆校园大讲堂公益巡讲”（14）
225. 游学八路军西安办事处纪念馆（24）
226. 西安-八路军西安办事处纪念馆（84）
227. **视频**：12

“西安事变”纪念馆

1. 【汉中蜀道石刻艺术博物馆】党史知识:西安事变旧址张学良公馆！（16）
2. 最新提示！西安这些景区需要核酸证明（594）
3. 铭记历史，缅怀先烈——西安事变纪念馆参观学习（245）
4. 红色展馆推介|西安事变纪念馆（34）
5. 招募|西安事变纪念馆社会志愿者招募啦~（513）
6. “寻找红色足迹·探寻爱国教育基地”党史学习实践活动系列之八路军西安事变办事处纪念馆（39）
7. 追忆 | 西安事变纪念馆（39）
8. 西安事变纪念馆“五一”假期参观指南（893）
9. 西安事变纪念馆，见证民族危难的关键转折（135）
10. 红色寻访｜西安事变纪念馆（87）
11. 隔壁的“西安事变纪念馆”（23）
12. 西安事变纪念馆恢复开放（1047）
13. 西安事变纪念馆暂停开放公告（1081）
14. 西安事变纪念馆的前生今事（136）
15. 西安事变纪念馆众志成城共同抗疫（298）
16. 西安事变纪念馆开展纪念西安事变85周年系列活动（87）
17. 点燃爱国情，国庆假期西安“红色游”受热捧（5528）
18. 国庆假期红色旅游持续火热！这些红色“打卡地”你都去过吗？（1399）
19. “红色之旅”上线啦！一起看看西安有哪些打卡地（5292）
20. 【海气|学党史】“寻访红色地标专题行动”第二十六站：西安事变纪念馆（52）
21. 西安事变纪念馆 回望峥嵘 铭记历史（47）
22. 【红色堡垒·百年党史】红色地图 | 西安事变纪念馆（45）
23. 特辑:西安事变纪念馆（30）
24. “回家乡，学党史”暑期社会实践|第四站“西安”西安事变旧址+八路军西安办事处纪念馆（261）
25. 精选陕西这几条红色旅游线路，带娃踏上红色之旅吧！（1697）
26. 西安事变纪念馆——西安免费景点（35）
27. 游陕西 | 陕西这些红色旅游目的地你都知道吗？（1215）
28. 集团水电公司总部党支部组织参观“西安事变”纪念馆（255）
29. “西安事变纪念馆”邮资机宣传戳片（124）
30. 学党史 坚定信念跟党走——走进西安事变纪念馆（141）
31. 党史学习 | 西安事变纪念馆：历史在这里转折（23）
32. 一起学党史 | 西安事变纪念馆：历史在这里转折（2679）
33. 参观西安事变纪念馆学党史铭党(（34）
34. 赴“西安事变纪念馆”开展主题党日活动（137）
35. 【党史学习】微展播——西安事变纪念馆（105）
36. 研究生 | 西安事变纪念馆红色景区党史教育实践活动（109）
37. 西安事变纪念馆讲解（155）
38. 网上红色展馆||西安事变纪念馆（40）
39. 百年党史｜西安事变纪念馆（578）
40. 主题党日 | 学党史、悟思想，走进西安事变纪念馆（178）
41. 心怀国之大者--硕9054党支部参观西安事变纪念馆（115）
42. 财经系 |【云游红色纪念馆（六）】了解西安事变——走进西安事变纪念馆（30）
43. 时代转换的枢纽——参观西安事变纪念馆（92）
44. 学四史 守初心 迎百年|红色文化研学·西安事变纪念馆（697）
45. 铭记|国家级抗战纪念设施、遗址名录-西安事变纪念馆（65）
46. 寒假生活】西安事变纪念馆观后感（135）
47. 西安事变纪念馆开展冬季旧址巡查工作（527）
48. 打卡红色教育基地，学四史行走课堂 | 西安事变纪念馆（278）
49. 西安事变纪念馆举办纪念西安事变84周年系列活动（14）
50. 追寻红色足迹|我院研究生参观“西安事变”纪念馆（565）
51. (一) 西安事变纪念馆（33）
52. This is Shaanxi: Xi'an Incident Memorial【这里是陕西】西安事变纪念馆（248）
53. 西安事变纪念馆昨起恢复正常开放（258）
54. 西安事变纪念馆恢复开放(预约免费游览)（237）
55. 又见长安（3）-----参观西安事变纪念馆（92）
56. 西安事变纪念馆关于闭馆的公告（846）
57. 西安之旅—西安事变纪念馆（56）
58. “不忘初心，牢记使命”丨丨参观西安事变纪念馆（378）
59. 参观西安事变历史纪念馆（128）
60. 走进西安事变纪念馆（184）
61. “博物馆月”第三弹:勿忘国耻，牢记历史——走进西安事变纪念馆（687）
62. 博物馆课｜西安事变纪念馆，止园《西安事变》（75）
63. “红色博物馆之城”之西安事变纪念馆（247）
64. 我和我的祖国|记参观西安事变纪念馆（208）
65. 践行践学||西安事变纪念馆——莫让历史成为历史（66）
66. 主动作为 自觉担当好革命旧址保护使命 ——王庆华赴西安事变纪念馆调研（483）
67. 西安事变纪念馆学习有感!（185）
68. 你去西安事变纪念馆了吗（91）
69. 参观“西安事变”纪念馆（90）
70. 西安事变纪念馆举办系列活动纪念西安事变82周年（28）
71. “西安事变”纪念日 那些历史转折发生地今何在？（154）
72. 探访西安事变纪念馆，了解近代历史性转折的那一刻（123）
73. 这里是西安事变纪念馆（276）
74. 碑林故事|高桂滋公馆——“西安事变旧址”（112）
75. 海峡两岸同祖同根一家亲 两岸五地 张学良纪念馆联盟年会在西安事变纪念馆召开（86）
76. 西安事变纪念馆（23）
77. 记录中国丨西安事变纪念馆（33）
78. 走进西安事变纪念馆，缅怀革命先烈（75）
79. 会展公司党支部赴“西安事变”纪念馆开展主题党日活动（68）
80. 西安事变纪念馆——寻走历史的踪影（十四）（91）
81. 【特色践学】红色之旅——三（1）班参观“西安事变纪念馆”（197）
82. 建筑学院研究生团总支组织庆“五四”青年节参观西安事变纪念馆活动（250）
83. 飞盘俱乐部红色游学系列活动之---西安事变纪念馆（263）
84. 西安事变纪念馆观后感（155）
85. 西安事变纪念馆举办活动纪念西安事变81周年（51）
86. 2013年古都之旅12——西安事变纪念馆（8）
87. 【陕西名胜】西安事变纪念馆（7）
88. [党校微课堂]现场教学篇——西安事变纪念馆（1274）
89. 学生党支部参观“西安事变”纪念馆（111）
90. 西安事变纪念馆（张学良公馆）游记（941）
91. 馆长访谈‖陈斌：西安事变纪念馆及周边将打造民国风情一条街（151）
92. 西安事变纪念馆免费讲解公告（321）
93. 【纪念馆巡礼】西安事变纪念馆（79）
94. “八办”纪念馆参加纪念长征胜利80周年、西安事变80周年座谈会（49）
95. 我馆举办系列活动纪念西安事变80周年暨纪念馆对外开放30周年（128）
96. 西科学子西安事变纪念馆上演话剧，引游客拍手叫好！（2028）
97. 我系学生干部培训系列活动——"西安事变纪念馆"参观学习（249）
98. [法学院暑期社会实践进行时]实践简报之"观西安事变遗址，得热血抗日精神"第一期（52）
99. 解放军94070部队与西安事变纪念馆共建爱国主义教育基地（198）
100. 双12涨姿势，一起看看西安事变纪念馆（841）
101. **视频：129**

汉中市川陕革命根据地纪念馆（156）

1、网上看展｜又见杜鹃红—川陕革命根据地妇女武装图片展(完整版)（179）

2、志愿红｜川陕博物馆“疫”线集结，守护巴城！（330）

3、喜报！我县这个地方被命名为省级青少年教育基地（140）

4、喜报！我市四家单位被命名为陕西省青少年教育基地！（1752）

5、@平昌人！“纪念川陕革命根据地创建90周年学术研讨会”征文开始啦（125）

6、等您投稿！“纪念川陕革命根据地创建90周年学术研讨会”征文开始啦（1721）

7、再添“历史记忆“，红军后代向川陕革命根据地博物馆捐赠文物（283）

8、纪念川陕革命根据地创建90周年 | “红军入川第一镇”的新生之路（1594）

9、【强国复兴有我】打卡红色教育基地 （二）川陕革命根据地纪念馆（70）

10、汉中这么多博物馆，忍不住想要分享给你！一起来“云”打卡吧（1036）

11、缅怀革命先烈，牢记革命初心 丨我公司组织全体员工参观汉中市川陕革命根据地纪念馆（50）

12、重温党领导下的中国青年运动史|政法学子走进陕西省汉中市川陕革命根据地纪念馆（122）

13、“云游”红色展厅-走进川陕革命根据地纪念馆（34）

14、红色展馆推介|川陕革命根据地纪念馆（22）

15、国际博物馆日，汉中这些好地方让您大饱眼福（654）

16、直播预告：川陕革命根据地博物馆邀您云上看展（713）

17、川陕革命根据地创建九十周年① | 川陕革命根据地博物馆（376）

18、汉中川陕革命根据地纪念馆最新招聘讲解员！（1923）

19、周末遛娃去哪儿？汉中这些宝藏地，真香！（1091）

20、探索红色博物馆、纪念馆高质量发展思考——以川陕革命根据地博物馆为例（下）（487）

21、探索红色博物馆、纪念馆高质量发展思考——以川陕革命根据地博物馆为例（上）（614）

22、西乡农商银行组织参观川陕革命根据地纪念馆（191）

23、学党史，颂党恩，跟党走（三十二）|川陕革命根据地纪念馆（30）

24、打卡廉政教育基地 | 川陕革命根据地纪念馆（2815）

25、【网上展馆】第十五站·川陕革命根据地纪念馆（1889）

26、川陕革命根据地纪念馆：建证“陕南红了半边天”丨红色建筑镌党史（4250）

27、下雨天不要慌，汉中这些好地方让你假期不“泡汤”（2208）

28、汉中“红色之旅”来了，去打卡吧！（5466）

29、"不忘烈士抛忠骨，民族复兴中国梦"一一记参观川陕革命根据地博物馆（255）

30、关注 | 川陕革命根据地红军烈士纪念馆：3年为30多位烈士找到亲人（1685）

31、云游红色纪念馆——【陕西篇】川陕革命根据地纪念馆（52）

32、川陕革命根据地纪念馆 | 不忘来路（78）

33、【学党史 开新局·云走红色圣地】川陕革命根据地纪念馆（51）

34、【HVAC】红色文化建筑（17）：川陕革命根据地纪念馆暖通空调设计（342）

35、注意 | 每个批次游客量不超过20人！川陕博物馆调整开馆时间和参观流程（1120）

36、工行汉中分行主题党日川陕革命根据地纪行（148）

37、青春汉中行·“三下乡”社会实践活动纪实报道（3） | 川陕革命根据地纪念馆篇（287）

38、“回家乡，学党史”暑期社会实践 | 第八站“汉中”川陕革命根据地纪念馆（232）

39、重走汉中红色足迹，助力红色旅游发展小队——川陕革命根据地纪念馆（234）

40、新进展！川陕苏区将帅碑林纪念馆新馆即将完工（3022）

41、【视频】川陕革命根据地纪念馆（32）

42、汉中红色旅游精品景点推荐（1819）

43、“陕西信合汉中党性教育基地” 在川陕革命根据地纪念馆挂牌成立（220

44、关注 | 点赞！川陕革命根据地红军烈士陵园被命名为全国妇女爱国主义教育基地（2446）

45、汉中市川陕革命根据地纪念馆关于规范参观团队管理相关事宜的公告（260

46、春到长安 | “永远跟党走”红色旅游精品线路（676）

47、传承红色基因 弘扬英模精神——汉中公安组织青年民警赴川陕革命根据地纪念馆开展红色教育（1222）

48、传承红色精神 筑牢红色阵地—县妇计中心参观汉中市川陕革命根据地纪念馆（193）

49、缅怀革命先烈，学习革命精神--县妇计中心参观汉中市川陕革命根据地纪念馆（830）

50、【工作动态】庆“三八妇女节” 汉中市赤土岭文化交流协会赴川陕革命根据地纪念馆参观学习（308）

51、汉中市川陕革命根据地纪念馆急聘讲解员！（2506）

52、汉中市川陕革命根据地纪念馆招聘（讲解员）（21）

53、汉中市川陕革命根据地纪念馆招募讲解员！（556）

54、汉中市川陕革命根据地纪念馆招聘公告（167）

55、2021年汉中市川陕纪念馆春节期间疫情受控开馆公告（103）

1. 川陕革命根据地博物馆景区提升工作日前完成（49）
2. 转发｜ 川陕革命根据地博物馆创新模式讲好红色故事（561）
3. “我为汉中代言”优秀奖作品：《川陕革命根据地》（71）
4. 赞！汉中连续两天上央视，因为......（1万）
5. “追寻历史足迹，点燃红色初心”汉中市川陕革命根据地纪念馆2020年“优秀历史文化进校园”活动（67）
6. 【红色印记】川陕革命根据地纪念馆（405）
7. 川陕革命根据地博物馆 组织开展“119”消防安全日宣传教育活动（91）
8. 点赞！川陕革命根据地博物馆2件文创产品获全省铜奖（2253）
9. 人社部工作组参观川陕革命根据地博物馆（90）
10. 川陕革命根据地博物馆党支部与市委办机关第四党支部联合开展主题党日活动（）474
11. 川陕革命根据地博物馆 川陕苏区将帅碑林纪念馆 开展“推广普通话宣传”进校园活动（178）
12. 川陕革命根据地博物馆 开展安全风险大排查大整治（114）
13. 全省唯一！川陕博物馆获全国“优秀活动单位”称号（3201）
14. 安全生产月|巴中消防深入川陕革命根据地博物馆开展消防知识培训（111）
15. 留存记“疫” 川陕革命根据地博物馆收到一批抗疫见证物品((（538）
16. 汉中市川陕纪念馆开展“5.18世界博物馆日”活动（51）
17. 汉中市川陕革命根据地纪念馆关于新型冠状病毒感染的肺炎疫情受控期恢复开馆的公告（48）
18. 汉中市川陕革命根据地纪念馆闭馆通知（365）
19. 汉中市川陕革命根据地纪念馆招募精英（4485）
20. 传承红色基因 铭记初心使命——汉中市公安局组织领导干部参观川陕革命根据地纪念馆（1080）
21. 汉中经开区组织党员赴川陕革命根据地纪念馆接受红色革命教育（111）
22. 川陕革命根据地博物馆2019年暑假“小小讲解员”培训开始了（426）
23. 汉中市科技职业中专党委赴川陕革命根据地纪念馆及何挺颖烈士故居开展学习教育活动（316）
24. 【纪念建党98周年】川陕革命根据地博物馆再掀参观热潮（339）
25. 汉中川陕革命根据地纪念馆关于规范研学实践教育秩序的公告（299）
26. 汉中市川陕革命根据地纪念馆关于规范研学实践教育秩序的公告（804）
27. “诵读红色经典 筑牢文化根基”汉中市川陕革命根据地纪念馆2018下半年“优秀历史文化进校园”活动（22）
28. 党史故事|秦巴山水间的红色往事——走进川陕革命根据地纪念馆（18）
29. 秦巴山水间的红色往事——走进汉中南郑川陕革命根据地纪念馆（279）
30. 秦巴山水间的红色往事——走进川陕革命根据地纪念馆（1454）
31. 汉中市川陕纪念馆开展“中国文化遗产日”宣传活动（5）
32. 汉中市川陕革命根据地纪念馆开展“优秀历史文化进校园”活动（101）
33. 揭秘！川陕革命根据地廉政教育馆“长”啥样？（925）
34. 汉中市（党员）干部党性教育基地——川陕革命根据地纪念馆命名授牌仪式（242）
35. 汉中中学组织党员参观川陕革命根据地纪念馆（1444）
36. 川陕革命根据地纪念馆被命名为全国爱国主义教育示范基地（393）
37. 【最新】陕南首个全国爱国主义教育示范基地在汉中举行揭牌仪式 看看在哪里（774）
38. 汉中南郑红寺湖，美得令人心醉！（1543）
39. 汉中市川陕革命根据地纪念馆讲解员招聘公告（722）
40. 探访川陕革命根据地红军烈士陵园：2万多名红军将士在此长眠（688）
41. 震撼！航拍全国最大红军烈士陵园，2.5万余名烈士安眠（4043）
42. 最大红军烈士陵园，25000名红军烈士在此安息！（711）
43. 汉中市川陕革命根据地纪念馆启动庆祝建国70周年专题展览活动（361）
44. 关注 | 全市新任组织委员参观川陕革命根据地纪念馆（58）
45. 汉中市川陕革命根据地纪念馆欢迎您（101）
46. 欢迎大家来南郑川陕革命根据地纪念馆（201）
47. 汉中市川陕革命根据地纪念馆开展业务学习（71）
48. 汉中市9.30烈士公祭活动 暨《汉中市川陕革命根据地纪念馆》 个性化邮票首发式（390）
49. 汉中市川陕革命纪念馆举办 抗日战争胜利70周年展览活动（386）
50. 汉中市川陕革命根据地纪念馆开展“博物馆进校园”活动（60）
51. 何挺颖烈士纪念碑广场和汉中市川陕革命根据地纪念馆简介
52. **视频：194**

**咸阳市旬邑县马栏革命旧址 （54）**

1. 红色会址推荐|第九期：马栏革命旧址（13）
2. 祝贺！旬邑县马栏革命纪念馆成功入选省级青少年教育基地（161）
3. 陕西革命旧址云上展（四十一）| 马栏红二十六军成立地旧址（22）
4. 公开招聘！马栏革命旧址公开招聘劳务派遣制讲解员（1880）
5. 【红色旅游】旬邑县 马栏革命旧址（36）
6. 马栏革命旧址管理处7月重要工作记事（75）
7. 红色印迹 | 走进马栏革命旧址，追寻革命初心（25）
8. 民革凤翔区总支部赴马栏革命旧址开展政治交接主题教育活动（30）
9. 三下乡 | “重温革命历史 讲述红色精神”党史实践队 | 马栏革命旧址（105）
10. 引镇街道赴咸阳市旬邑县马栏革命纪念馆开展“喜迎二十大·奋进新征程”主题党日活动（502）
11. 【动态】省市侨联赴马栏革命旧址开展主题党日活动（36）
12. 中铁一局铁建公司到咸阳市旬邑县马栏革命旧址开展“开路先锋、星火传承、一路争先、筑梦前行，奋进喜迎二十大”主题党日实地践学活动（168）
13. 高新区庙北村党总支赴马栏革命纪念馆开展主题党日活动（380）
14. 【聆听历史回响，阔步新的征程】咸阳市一院党员走进马栏革命旧址~（1533）
15. 主题党日活动|追寻红色印记——走进马栏革命旧址（247）
16. 既有历史名迹，又有自然奇景——咸阳的这些绝美风光真的值得去看看（392）
17. 【要闻】​旬邑县县级党员领导干部赴马栏革命旧址开展“主题党日”活动（992）
18. 咸阳旬邑县马栏镇：“四红”工程为乡村振兴蓄势赋能（25）
19. 老区新貌 | 马栏镇：革命老区展新颜 美丽乡村入画来（237）
20. 陕西革命旧址云上展（28）| 马栏革命旧址（29）
21. 【乡村振兴】咸阳市：陕甘宁革命老区咸阳市旬邑县马栏镇——红色沃土盛开振兴之花（6）
22. 旬邑县召开红色马栏建设专题会议（1344）
23. 旬邑：赓续传承马栏红色基因 谱写高质量发展新篇章（550）
24. 咸阳市委常委班子到旬邑县瞻仰马栏革命旧址（38）
25. 缅怀先辈以史为鉴 坚守初心开创未来 市委常委班子到旬邑县瞻仰马栏革命旧址（168）
26. 市委常委班子到旬邑县瞻仰马栏革命旧址（1.1万）
27. 雪漫马栏革命旧址（375）
28. 打卡廉政教育基地 | 马栏革命纪念馆（2903）
29. 【革命旧址巡礼】马栏革命旧址（156）
30. 【要闻】旬邑县召开马栏革命纪念馆园区整体保护提升方案评审会议（777）
31. 咸阳“红色之旅”来啦！一起打卡吧！（3729）
32. 市法援中心党总支联合市司法局公共法律服务处党支部参观马栏革命旧址开展主题党日活动（94）
33. 诗意旬邑|《西行漫记》之马栏革命纪念馆（329）
34. 【要闻】旬邑县召开马栏革命纪念馆园区整体保护提升方案征求意见座谈会（508）
35. 寻访革命足迹 传承红色基因——蓝田县城关中学组织党员及教师赴马栏革命纪念馆参观学习（627）
36. 党史故事我来讲丨第10站：走进马栏革命旧址（121）
37. 【项目发布】旬邑县马栏革命老区综合旅游开发项目（23）
38. 咸阳红色记忆（三）:旬邑马栏革命旧址和马家堡关中特区旧址（86）
39. 七一前夕勺勺客党工代表参观马栏革命旧址（86）
40. 建党100周年丨西安公安交警走进马栏革命旧址开展迎“七一”颂党恩主题党日活动（1130）
41. 合阳法院赴马栏革命旧址、扶眉战役纪念馆接受革命传统教育（组图（39）
42. 合阳法院赴马栏革命旧址、扶眉战役纪念馆接受革命传统教育（24）
43. 打卡马栏革命旧址| 追寻革命足迹，感悟初心使命（1441）
44. “忠诚号”列车第四站打卡| VR全景！走进马栏革命旧址（1723）
45. 马栏革命旧址（50）
46. 奋斗百年 一路向阳 | 走进马栏革命旧址 追寻红色记忆（87）
47. 学史力行之旅---“学习党的历史，重走红色道路”主题活动启动仪式在旬邑县马栏革命纪念馆隆重举行（871）
48. 【旬邑党史故事】马栏革命峥嵘岁月（300）
49. 【县区动态】旬邑县城东离退休干部党支部赴马栏革命旧址开展主题党日活动（14）
50. 党史学习教育｜追寻红色印记 讲好党史故事③走进马栏革命旧址（420）
51. 三秦大地上的红色印记丨马栏革命旧址所在地旬邑县（225）
52. 红色教学点·马栏革命纪念馆（262）
53. 今年花开逛咸阳丨“忆百年·红色游”来咸阳旬邑，感受马栏革命精神，赏祖国大好河山~（440）
54. 百个红色景点"云"参观⑱ | 咸阳市旬邑县马栏革命旧址（99）
55. “重温革命历史，传承红色基因”—秦直道律所参观学习马栏革命旧址（324）
56. 【奋斗百年路 启航新征程 】旬邑马栏：讲述党史故事 传承红色基因（159）
57. 百篇陕西红色经典|第十篇　马栏革命旧址（415
58. 【学党史 · 忆足迹】红色记忆----马栏革命旧址（742）
59. 旬邑县博物馆、马栏革命纪念馆邀您来投票啦！（216）
60. 马栏革命纪念馆被确定为全国关心下一代党史国史教育基地（93）
61. 【红色教育】旬邑县马栏革命纪念馆被授予“全国关心下一代党史国史教育基地”（34）
62. 【感悟历史】参观旬邑马栏革命旧址随想（290）
63. 咸阳市级领导班子在马栏革命旧址开展主题教育（4006）
64. 咸阳市级领导班子成员集体在马栏革命旧址重温入党誓词（2759）
65. 走进旬邑马栏革命纪念馆追寻革命传统教育（292）
66. 【壮丽70年 奋斗新时代】走进马栏革命旧址：军民同心大生产，为边区筑起红色堡垒（86）
67. 新筑社区卫生服务中心党支部赴马栏革命旧址开展主题党日活动（62）
68. 记者再走长征路 | 走进红色马栏，传承红色基因（71）
69. 壮丽70年 奋斗新时代——记者再走长征路|走进马栏革命旧址：军民同心大生产 为边区筑起红色堡垒（286）
70. 走进马栏革命纪念馆：铭记苦难 镌刻辉煌（431）
71. “七一”献礼，走近红色热土——咸阳旬邑！（478）
72. 【“七一”活动】区人大办党支部在旬邑县马栏革命旧址开展庆“七一”主题党日活动（96）
73. 2019咸阳踏青赏花旅游季丨相约马栏，踏上红色之旅~（261）
74. 东盟学子赴马栏革命纪念馆学习（104）
75. 机械工程学院学生第一党支部参观马栏革命旧址（113）
76. 参观马栏革命旧址 接受革命传统教育（842）
77. 旬邑测区组织参观学习马栏革命旧址（73）
78. 推荐丨“七一”献礼，邀您走近马栏革命旧址（53）
79. “绿色中国行”走进革命老区陕西旬邑（295）
80. [参观学习]市机关事务管理局组织离退休党员赴旬邑马栏革命旧址参观学习（39）
81. 光辉岁月|人文学院组织师生党员赴马栏革命旧址学习（63）
82. 2018咸阳踏青赏花旅游季丨相约马栏，踏上红色之旅~（103）
83. 【红色印记】马栏革命旧址（333）
84. 社市委秦川支社走进马栏革命旧址开展‘’九三秦川 红色马栏‘’书画主题创作筆会（52）
85. 到咸阳旬邑红色马栏重温峥嵘岁月！（333）
86. “七一”献礼，邀您走进马栏革命旧址（812）
87. “七一”献礼，邀您走近马栏革命旧址（1642）
88. 清明去哪儿丨到旬邑马栏革命旧址缅怀先烈，寄托哀思……（224）
89. “马栏革命旧址”被命名为全国爱国主义教育示范基地（1910）
90. 主题党日活动】咸阳机场绿化保洁公司组织党员赴马栏革命旧址开展主题党日活动（217）
91. 十一游咸阳，咸阳交警为您护航平安（3011）
92. 避暑何必远飞，旬邑就可消夏（864）
93. 咸旬高速沿线旅游采风掠影 旬邑显风姿（1023）
94. 网易旅游：周末去旬邑游马栏革命旧址 探秘赵家洞崖居（262）
95. 【西邮快讯】大学生记者团赴旬邑马栏革命旧址参观学习（222）
96. 【足于心 行于迹】51走进旬邑之马栏革命旧址游记（298）
97. 微咸阳|红色马栏，大美旬邑!（227）
98. 我县马栏革命旧址被评为为国家AAAA级旅游景区（36）
99. 咸阳马栏革命旧址成功创建国家4A级旅游景区（21）

**铜川市陕甘边照金革命根据地旧址（24）**

1. 陕甘边革命根据地照金纪念馆与孙思邈纪念馆开展馆际交流活动（42）
2. 【红色档案故事展播】以照金为中心的陕甘边革命根据地的创建（66）
3. 陕甘边革命根据地照金纪念馆与铜川博物馆开展馆际交流（219）
4. 层林尽染 美如画卷 照金景区为你奉上秋色盛宴！（187）
5. 红色血脉 | 走近陕西的革命纪念馆（776）
6. 党史天天学（二百四十）——陕甘边革命根据地照金纪念馆（10）
7. 来照金，追寻当年的红色记忆（76）
8. 忆往昔峥嵘岁月 望中华强国复兴④——陕甘边革命根据地照金纪念馆（163）
9. 照金纪念馆公开招聘合同制讲解员6名！（3700）
10. 照金精神，绽放时代光芒（205）
11. 全国首批！陕甘边革命根据地照金纪念馆入选“大思政课”教学基地（250）
12. 陕甘边革命根据地照金纪念馆入选“大思政课”教学基地（351）
13. 耀州窑博物馆同陕甘边革命根据地照金纪念馆开展学习交流活动（180）
14. 实践篇 | 第五弹—陕甘边革命根据地照金纪念馆（144）
15. 三下乡 | 追寻红色足迹（三）——铜川市陕甘边革命根据地照金纪念馆（145）
16. 【这里是铜川】全国百家红色旅游经典景区陕甘边照金革命根据地（233）
17. 红色展馆推介|陕甘边革命根据地照金纪念馆（37）
18. 铜· 影视丨【陕西电影】照金薛家寨革命旧址影视场景地！（12）
19. 资源推介｜照金薛家寨革命旧址影视场景地（1447）
20. 追忆｜陕甘边革命根据地·英雄照金（34）
21. 铜川市陕甘边根据地照金革命旧址保护条例（254）
22. 【海气|学党史】 “寻访红色地标专题行动” 第四十四站：照金纪念馆（160）
23. 中国精神丨传承红色基因，感悟兴国之魂——照金精神（1322）
24. 陕甘边革命根据地照金纪念馆：红色“照金”光辉永存丨红色建筑镌党史（1128）
25. 陕甘边根据地照金革命旧址保护条例颁布（64）
26. 红色照金（706）
27. 《铜川市陕甘边根据地照金革命旧址保护条例》颁布（74）
28. 铜川市举办纪念陕甘边革命根据地创建88周年活动（34）
29. 【红色照金】纪念陕甘边革命根据地创建88周年“弘扬照金精神·赓续红色血脉”专题展览（214）
30. 【照金纪念馆特辑】铜川市举办纪念陕甘边革命根据地创建88周年活动（2502）
31. 照金革命旧址保护公益诉讼工作纳入《铜川市陕甘边照金革命旧址保护条例》（104）
32. 走进陕西革命圣地，让红色精神薪火相传（163）
33. 陕西红色地标（五）——陕甘边革命根据地照金纪念馆（32）
34. 印记 | 红色政权的摇篮——照金（391）
35. 基地-陕甘边革命根据地照金纪念馆（83）
36. 【党史学习教育】照金：熠熠生辉的革命创业精神（309）
37. 【红色基地】红军落脚•抗日出发——陕甘边革命根据地照金纪念馆（341）
38. 追寻“红色印迹”】陕甘边革命根据地照金纪念馆（8）
39. 【百年党史】陕甘边革命根据地在中国革命史上写下了光辉的一页（23）
40. 永远跟党走 | 红色旅游精品景区推荐之铜川篇（1577）
41. 红色印记乡村行丨照金村：革命“火种”照亮新生（220）
42. 百年路·新征程⑦ | 如果老一辈革命家看到今天的照金镇……（4092）
43. 党史学习·足迹 | 打卡红色地标之陕甘边革命根据地照金纪念馆（50）
44. 百年历程 | 照金陕甘边根据地（26）
45. 【百年路•同心筑】这块革命根据地能“硕果仅存” 离不开灵活务实的统战政策（1277）
46. 博物馆（十九）陕甘边革命根据地照金纪念馆（106）
47. 陕西革命旧址云上展（十三）| 陕甘边革命根据地照金纪念馆​（154）
48. 党史学习教育｜追寻红色印记 讲好党史故事⑥走进陕甘边照金革命根据地（294）
49. 巍巍宝塔 初心永照——习近平总书记到过的红色圣地之陕西篇（4028）
50. 报名参加活动啦 | 走进陕甘边革命根据地照金纪念（1698）
51. 开展党团活动，感受照金精神(二)参观陕甘边革命根据地照金纪念馆（317）
52. 百篇陕西红色经典|第四十五篇 陕甘边革命根据地照金纪念馆（157）
53. 【红色照金】三秦大地上的红色印记——陕甘边革命根据地照金纪念馆（174）
54. 三秦大地上的红色印记丨陕甘边革命根据地照金纪念馆（1449）
55. 百年历程 红色记忆 | 照金陕甘边根据地（1208）
56. 红旗漫卷——陕西革命旧址云上展之十三：陕甘边革命根据地照金纪念馆（13）
57. 学党史｜陕甘边革命根据地——照金纪念馆（802）
58. 【照金纪念馆特辑】纪念陕甘边区革命委员会成立88周年（331）
59. 【红色铜川党史】陕甘边革命根据地照金纪念馆（327）
60. 【夜读】陕甘燃星火 红日映照金——走进陕甘边革命根据地照金纪念馆（1971）
61. 走进陕甘边革命根据地照金纪念馆，感受中国革命的星星之火（2257）
62. 三秦大地上的红色印记｜以照金为中心的陕甘边革命根据地（32）
63. 党史教育| 陕甘边革命根据地中心为何选在照金（18）
64. 【红色照金 滋养初心】探访陕甘边革命根据地照金纪念馆（172）
65. 民法典 与你同行 铜川市开展“民法典进单位”走进陕甘边 革命根据地照金纪念馆活动（127）
66. 全国普法办确定第二批全国法治宣传教育基地陕甘边革命根据地照金纪念馆入选（64）
67. 基地简介 ▏陕甘边革命根据地照金纪念馆（1501）
68. 市政协对口协商陕甘边照金革命旧址保护利用工作（422）
69. 陕甘边革命根据地照金打造“红色书香小镇”（252）
70. 全省税务系统党员干部学习教育基地在陕甘边革命根据地照金纪念馆挂牌成立（4718）
71. 【照金故事】陕甘边革命根据地的创立与发展（85）
72. 宜川县行知幼儿园全体教职工游学篇 之陕甘边照金革命根据地（193）
73. 读回信、学精神、传承红色基因——陕甘边照金革命旧址图话展走进阳光中学（85）
74. 【就业招聘】2018年照金纪念馆讲解员招聘简章（5598）
75. 感受红色文化，走进照金革命根据地（409）
76. 旅游｜全省文化旅游名镇（二）——耀州区照金镇（1.1万）
77. 陕西铜川.陕甘边革命根据地照金纪念馆（20）
78. 陕甘边革命根据地照金纪念馆“中国华侨国际文化交流基地”揭牌仪式举行 郭大为程勉贵等出席（171）
79. 红色革命圣地，陕甘边革命根据地——铜川照金纪念馆（149）
80. 陕西旅游登陆纽约时代广场　铜川照金在列（1959）
81. 走进陕甘边革命根据地照金纪念馆（255）
82. 公告丨陕甘边革命根据地照金纪念馆关于每周一闭馆的通知（39）
83. 陕甘边革命根据地照金纪念馆关于每周一闭馆的通知（827）
84. 照金革命旧址之兔儿梁（387）
85. 西北地区创立的第一个山区革命根据地——陕甘边照金（60）
86. 陕甘边照金革命根据地旧址参选全国“我最向往的党史纪念地”评选活动（672）
87. 陕甘边革命根据地照金纪念馆：留存红色记忆 传承革命精神（538）
88. 陕西铜川的陕甘边照金革命根据地纪念馆（组图）（142）
89. 【招聘】陕甘边革命根据地照金纪念馆2016年讲解员招聘简章（18）
90. 【就业招聘】陕甘边革命根据地照金纪念馆2016年讲解员招聘简章（1306）
91. 铜川市陕甘边照金革命根据地旧址（38）
92. 【旅游动态】陕甘边照金革命根据地旧址生态旅游基础设施（二期）项目初步设计评审会今召开（153）
93. #青春有梦话照金#【第一期】陕甘边革命根据地照金苏区的历史地位（26）
94. 【耀州画卷】红色旅游（二）陕甘边革命根据地照金纪念馆（475）
95. 历史耀州【陕甘边照金革命根据地的创建与发展（三）】（12）
96. 历史耀州【全国重点文物保护单位—陕甘边照金革命根据地旧址】（22）
97. 【公告栏】陕甘边革命根据地照金纪念馆 兑换免费参观券公告（10）
98. 红色记忆——原陕甘边照金革命根据地（14）

**渭南市华县渭华起义纪念馆（50）**

1. 追寻红色足迹 汲取奋进力量 | 记渭华起义纪念馆学习纪实（545）
2. 感悟先辈精神 强固信仰信念（1014）
3. “云游”红色展馆-走进渭华起义纪念馆（27）
4. 走进渭南渭华起义纪念馆 重温红色记忆（348）
5. 【跟着典故游渭南】渭华起义（45）
6. 陕西革命旧址云上展（二十七）| 渭华起义旧址（12）
7. 【红色故事】渭华起义纪念碑（13）
8. 展览预热 | 疫情未了，不想出门？这个展为您来解解压！（第一辑）（1487）
9. “青年读党史”第24期 | 渭华起义（583）
10. 【红色基地云参观】渭华起义纪念馆（78）
11. 渭华起义纪念馆保存的两件文物（321）
12. 故乡华州高塘渭华起义纪念馆与江南印象竹溪里摄影（329）
13. 周二：思党史事件 | 渭华起义（216）
14. 浪走 → 看皮影、逛公园、吃花馍...（932）
15. 【党史学习时】渭华起义的革命精神永放光芒（196）
16. 陕西红色地标（九）——渭华起义纪念馆（27）
17. 渭华起义纪念馆：渭南地区首个！（1305）
18. 事关渭华起义纪念馆，渭南地区首个！（1958）
19. 【回顾】站在渭华起义纪念馆旁，突然，我想到了这些事...（857）
20. 循红色足迹 看交通新貌|高塘镇： 好路带来好生活（76）
21. 渭南市华州区图书馆、文化馆、渭华起义纪念馆暂时闭关（74）
22. 渭南市华州区渭华起义景区8月8日暂停对外开放公告（62）
23. 区政协机关赴渭华起义纪念馆开展党史学习教育主题党日活动（579）
24. 渭南市华州区：参观渭华起义陈列大厅（307）
25. 渭南市华州区：参观渭华起义指挥部旧址（631）
26. 知史爱党 兴企力行 | 第22站：党史故事大家讲（347）
27. “重温百年党史 坚定初心使命”西安石油大学党史教育培训邀请函（71）
28. 华阴市红十字会参观渭华起义纪念馆（27）
29. 庆建党百年 | 渭南推出6条红色精品旅游线路（一）（2128）
30. 党史故事 | 渭华起义：革命火种从这里撒播（6）
31. 参观渭华起义纪念馆（38）
32. 我所组织参观学习渭华起义纪念馆（252）
33. 奋进100年 红色建筑多 | 渭华起义响春雷 红色塔馆传基因（288）
34. 【党史学习教育】渭南革命遗存 — 渭华起义纪念馆（77）
35. 【三贤文苑】建党百年特刊 · 詹俊峰 | 红歌唱响颂党恩 渭华精神永传承（178）
36. 走进渭华起义纪念馆 感受红色文化魅力----华阴市委统战部组织参观渭华起义纪念馆（28）
37. 报名参加活动啦 | 走进渭华起义纪念馆（554）
38. 华阴市委统战部组织参观渭华起义纪念馆（442）
39. 打卡“红色记忆”，一起“陕”闪放光彩！娘家人又有好活动等你来！（1959）
40. “学党史・助全运” | 高新区民政局庆助残日组织打卡渭华起义纪念馆，督导体验辖区道路无障碍设施（81）
41. 陕西总队党委组织参观渭华起义纪念馆（219）
42. 党史学习｜渭华起义：革命火种从这里撒播（15）
43. 【秦东风云录】渭华起义：革命火种从这里撒播（95）
44. 巾帼大学习| 渭华起义：革命火种从这里撒播（8）
45. 民建渭南市直师院支部赴渭华起义纪念馆开展主题教育活动（185）
46. 【天天学党史】第18期云参观：渭华起义纪念馆（4078）
47. “宝塔山下话党史”┃第九集 渭华起义——播撒西北地区革命的火种（1973）
48. 为期四天！渭华起义纪念馆活动今日启动！（442）
49. 秦女子党史课堂 | 渭华起义：革命火种从这里撒播（866）
50. 红色起点·渭华起义纪念馆（235）
51. 【党史学习教育】渭华起义：革命火种从这里撒播（34）
52. 党史回眸 | 渭华起义：革命火种从这里撒播（85）
53. 华西镇赴渭华起义纪念馆参观学习（511）
54. 渭华起义：革命火种从这里撒播（1.5万）
55. 百篇陕西红色经典|第三十三篇 渭华起义纪念馆（182）
56. 渭南，不甘心！（6.8万）
57. 大明杜湾：渭华起义纪念馆缅怀革命先烈//王立秋（1320）
58. 一组来自渭华起义纪念馆的照片​（1210）
59. 弘扬红色革命精神 锤炼忠诚担当品质——华阴市委政法委参观渭华起义纪念馆（330）
60. “四史”学习——追寻历史足迹，传承红色基因（149）
61. 【注意】渭华起义纪念馆，春节暂不开放！（928）
62. 渭华起义：中国革命走向复兴的重要历史路标（152）
63. 西安蓝田公安赴渭华起义纪念馆开展革命传统教育！（236）
64. 蓝田公安赴渭华起义纪念馆开展革命传统教育！（2028）
65. (二) 渭华起义纪念馆（34）
66. 渭南市博物馆党支部组织退役军人、党员及入党积极分子赴渭华起义纪念馆开展八一建军节纪念活动（1011）
67. 记录发现|陕西渭南渭华起义纪念馆（110）
68. 铜川市市场监管局非公企业党委组织直属支部书记赴渭华起义纪念馆和杨震廉政教育基地开展教育活动（113）
69. 每日一学||【爱国主义教育基地】渭南华州：渭华起义纪念馆（40）
70. 华阴市环保局党支部组织参观渭华起义纪念馆（20）
71. 渭华起义纪念馆：体验红色旅游 传承红色精神（613）
72. 【陕西党史】陕西省召开渭华起义纪念馆和烈士陵园筹建工作会议（4904）
73. 省发改委青年干部赴渭华起义纪念馆接受爱国主义教育（270）
74. 【视频】渭华起义纪念馆春节免门票+戏曲演出，及渭南周边景区民俗活动时间出炉！（1076）
75. 【三贤文苑】采风文//张会养//走进渭华起义纪念馆（180）
76. 【三贤文苑】采风文//陈 茹 | 参观渭华起义纪念馆（146）
77. 【学校要闻】渭华起义纪念馆（190）
78. 渭华起义纪念馆改扩建项目基本完工（689）
79. 离退休党支部组织离退休党员参观渭华革命纪念馆（63）
80. 华州区启动渭华起义纪念馆4A级景区申报工作（37）
81. 我院进行纪律教育学习宣传主题系列活动——参观渭华革命起义纪念馆（368）
82. 蒲城交警大队组织全体党员赴渭华起义纪念馆参观学习（501）
83. 渭南这个地方也要建渭华起义纪念馆，占地420亩！（5427）
84. 华阴市工商联组织参观渭华起义纪念馆（260）
85. 谒渭华起义纪念碑（200）
86. 祝红利 || 戊戌年清明诗三首（409）
87. 带你了解渭华起义烈士纪念馆（12）
88. 【渭南旅游】带你了解渭华起义烈士纪念馆（97）
89. 百姓问卷：票选你心中最美的渭南景点！（3860）
90. 缅怀革命先烈，坚定理想信念——中共五环中学党支部参观渭华起义纪念馆记（669）
91. 红5月，去渭南渭华起义纪念馆看看！（96）
92. 渭南纪检监察邀您游廉政教育基地（647）
93. 华山中学组织学生参观渭南博物馆和渭华起义纪念馆（346）
94. 【渭南景区】走，去华州区渭华起义纪念馆参观一番！（375）
95. 缅怀先烈牢记使命—市科技局组织党员赴渭华起义纪念馆学习参观（346）
96. 【渭南资讯】华县渭华起义旧址（105）
97. 习大大故乡如此美（9006）
98. 【渭南资讯】华县县政府深入渭华起义纪念馆检查烈士公祭日准备工作（24）
99. 【渭南资讯】上好入伍第一课 ——华县新兵参观渭华起义纪念馆（62）
100. 渭南华县150名新兵赴渭华起义旧址宣誓83

**榆林市红色旅游系列景区(米脂县杨家沟革命旧址（78）,佳县神泉堡革命纪念馆（50）,绥德县革命历史纪念馆（19）)**

**米脂县杨家沟革命旧址**

1. 西安之外，藏着一个“小北京”，冷门又好吃（2.7万）
2. 米脂县杨家沟革命纪念馆召开学习贯彻党的二十大精神专题学习会（288）
3. 党史故事 | 陕北小村庄见证历史大转折（6）
4. “不断把党建设得更加坚强有力”——习近平推动新时代党的建设的故事（332）
5. 《这里是榆林》之红色榆林（111）
6. 红色血脉 | 走近陕西的革命纪念馆（776）
7. 关注 | 区纪博中心深入学习贯彻党的二十大精神（184）
8. 米脂杨家沟革命旧址：赓续红色血脉 续写老区荣光（23）
9. 米脂县创建全域旅游示范县暨杨家沟革命旧址创建国家AAAA级旅游景区动员大会召开（1005）
10. 米脂县举行创建全域旅游示范县暨杨家沟革命旧址创建国家AAAA级旅游景区动员大会（183）
11. “苏&陕红色云旅游”暑期社会实践|第三小分队第三辑： 榆林米脂杨家沟革命旧址（36）
12. 米脂杨家沟村、高西沟村——风展红旗如画 绿映时代华章（226）
13. 把战略的坚定性和策略的灵活性结合起来（2.4万）
14. 喜迎二十大 奋进新征程——米脂县杨家沟革命纪念馆“七一”期间迎来客流量高峰（1482）
15. 【党务动态】粒状硅生产部党支部赴米脂县杨家沟革命纪念馆开展主题党日活动（356）
16. 米脂县杨家沟革命旧址：红色血脉代代传（15）
17. 榆林日报 || 杨家沟革命旧址：红色血脉代代传（455）
18. 足迹·温暖的回响 || 米脂县杨家沟革命旧址：走好赓续红色血脉之路（10）
19. 安全规范，平稳有序——米脂县杨家沟革命纪念馆“五一”假期运行情况（210）
20. 共青团榆林市委在杨家沟革命旧址举办“喜迎二十大、永远跟党走、奋进新征程”主题团日示范活动（950）
21. 【红色米脂】杨家沟革命旧址（123）
22. 杨家沟村—— 黄土地上的红色记忆（30）
23. 日出东方：新年曙光中的杨家沟（954）
24. 2022年新年献词——米脂县杨家沟革命纪念馆（649）
25. 百年初心成大道 万里征程作雄行（4.4万）
26. 以习近平同志为核心的党中央领导开展党史学习教育纪实（2万）
27. 米脂县杨家沟革命纪念馆关于暂停开放的通知（434）
28. 垄耘：米脂杨家沟（8295）
29. 不可逆转的历史进程（8.1万）
30. 【重磅】不可逆转的历史进程（3.7万）
31. 米脂县杨家沟革命纪念馆（10）
32. 学习习近平总书记来陕考察重要讲话重要指示精神——考察米脂县杨家沟革命旧址篇（129）
33. 金秋九月总书记来过——米脂杨家沟革命旧址（563）
34. 来，看直播！（3.8万）
35. 跟随总书记学党史|中共中央的杨家沟岁月（3368）
36. 杨家沟革命旧址：追忆革命历史 赓续红色血脉（1599）
37. 【追寻“红色印迹”】陕西榆林：杨家沟革命旧址（15）
38. 习近平考察的这个地方，背后故事多（3.3万）
39. 红色百宝 奋斗百年|陕西榆林：杨家沟革命旧址（37）
40. 总书记的期望，让杨家沟革命纪念馆工作人员信心满满——从红色资源中汲取前行的智慧和力量（822）
41. 新华全媒+丨榆林是个好地方（3833）
42. 习近平来陕考察调研，走进榆林米脂杨家沟革命旧址！（216）
43. 习近平陕西行丨走进米脂县高西沟村、杨家沟革命旧址（787）
44. 【建党百年】榆林红色景点：杨家沟革命纪念馆（562）
45. 三下乡 | 米脂杨家沟革命旧址宣讲（685）
46. 米脂县杨家沟革命纪念馆召开干部作风集中整顿工作动员会（463）
47. 米脂县杨家沟革命纪念馆对新冠疫情防控工作再安排再落实！（90）
48. 参观米脂县杨家沟革命纪念馆防疫须知！（614）
49. 毛泽东住过的米脂县杨家沟：三产联动焕发新活力（1237）
50. 【项目发布】米脂杨家沟红色旅游（一期）项目（18）
51. 庆祝建党100周年 | 走进红色纪念馆丛书之《中国革命从这里走向胜利——米脂县杨家沟革命纪念馆》出版（47
52. 榆林职院机电系赴佳县神泉堡、米脂县杨家沟革命纪念馆开展党史学习教育（222）
53. 党史学习教育￨赴米脂县杨家沟革命纪念馆（117）
54. 红色遗迹寻访 | 集团九公司党支部走进米脂杨家沟革命旧址，汲取奋进力量（391）
55. 米脂县杨家沟革命纪念馆向交警大队桥河岔中队赠送锦旗（605）
56. 米脂县杨家沟革命纪念馆召开党史学习教育动员会（479）
57. “印记”微党史摄制组走进 米脂县杨家沟革命纪念馆||花絮（48）
58. 【红色教育】米脂县杨家沟革命纪念馆被授予“全国关心下一代党史国史教育基地”（109）
59. 米脂县杨家沟革命纪念馆被授予“全国关心下一代党史国史教育基地”（596）
60. 白庚胜考察米脂县杨家沟革命纪念馆（424）
61. 足迹·温暖的回响丨米脂杨家沟革命旧址：走好赓续红色血脉之路（10）
62. 米脂县杨家沟革命纪念馆“四进”活动之走进米脂县移动公司（277）
63. 米脂县杨家沟革命纪念馆被中共榆林市纪委命名为榆林市廉政教育基地（444）
64. 米脂县杨家沟革命纪念馆迎来“七·一”党建红色旅游新高潮（341）
65. 米脂县杨家沟革命纪念馆传达学习贯彻习近平总书记来陕考察重要讲话重要指示精神（576）
66. 米脂县杨家沟革命纪念馆被省里“点名”！（16）
67. 米脂县杨家沟革命纪念馆被陕西省委组织部确定为“全省干部教育培训现场教学点”（215）
68. 神东煤炭集团党委办公室党支部与米脂县杨家沟革命纪念馆党支部举行共建活动（1408）
69. 张凯深入米脂县杨家沟革命纪念馆党风廉政教育基地调研（516）
70. 齐战疫情 共克时艰——米脂县杨家沟革命纪念馆疫情防控纪实（153）
71. 米脂县杨家沟革命纪念馆暂时闭馆通知（1097）
72. 米脂县杨家沟革命纪念馆教育教学基地揭牌仪式（331）
73. 米脂县杨家沟革命纪念馆传达学习党的十九届四中全会精神（321）
74. 共青团米脂县委、米脂县科协赴杨家沟革命旧址开展“不忘初心 牢记使命”主题党日活动（398）
75. 我校组织基层党组织书记赴陕西省米脂县杨家沟革命旧址参观学习（488）
76. 榆林市米脂县杨家沟革命旧址及纪念馆（71）
77. 米脂县举行杨家沟革命旧址毛主席塑像落成仪式（1237）
78. 让红色记忆代代传 ——走进米脂县杨家沟革命纪念馆（27）
79. 杨家沟革命旧址（114）
80. 米脂县杨家沟：中国革命从这里走向胜利（669）
81. 榆林红色遗址——杨家沟革命纪念馆（340）
82. 有一处红色革命圣地叫米脂杨家沟！（1.2万）
83. 【学习梁家河 筑梦新时代】米脂县杨家沟革命纪念馆组织学习《梁家河》（151）
84. 汉中市老干部考察团来米脂县杨家沟革命纪念馆参观学习（186）
85. 万万没想到，榆林这些革命旧址竟藏着这么多故事！（5993）
86. 体育学院师生党员干部赴米脂县杨家沟革命纪念馆参观学习（382）
87. “再走转战陕北路”活动走进米脂县杨家沟革命纪念馆（14）
88. 【红色记忆】杨家沟革命旧址（284）
89. [实践在路上]--米脂行（100）
90. 【美丽乡村】米脂县（3767）
91. 米脂县多措并举打造杨家沟精品旅游景区（935）
92. 杨家沟革命旧址旅游记（1208）
93. 米脂杨家沟入选《全国红色旅游经典景区名录》（1498）
94. 走进榆林市米脂县杨家沟革命旧址（27）
95. 【塞上旅游】米脂县杨家沟革命纪念馆（17）
96. 榆林市爱国主义教育基地巡礼：米脂县杨家沟革命纪念馆（8）
97. 米脂县杨家沟镇荣获省级生态镇称号（2610）
98. 走进陕西省榆林市米脂县杨家沟革命旧址（175）
99. 王岐山在陕西调研：说了什么？看了什么？（10万+）
100. 我院党员赴米脂县杨家沟革命纪念馆参观学习（148）

**佳县神泉堡革命纪念馆**

1. 改革创新 再接再厉丨经开快讯第九期（总九期）（214）
2. 佳小团陪您唠唠“佳”常（245）
3. 《这里是榆林》之红色榆林（219）
4. 佳县东方红和神泉堡纪念馆专题学习党的二十大精神（89）
5. 佳县东方红和神泉堡纪念馆10月27日作风建设专项行动推进会（57）
6. 【最深的缅怀是铭记，最好的纪念是传承】神泉堡革命纪念馆赋（111）
7. 我县残联党支部联合子州县残联党支部走进佳县神泉堡革命纪念馆进行参观学习（15）
8. 国庆假期不远行！诗和远方在榆林！（1.6万）
9. 关于佳县神泉堡革命纪念馆恢复对外开放的公告（79）
10. 关于佳县神泉堡革命纪念馆暂停对外开放的公告（385）
11. 西安学子三下乡：参观佳县神泉堡革命纪念馆（26）
12. 清华大学优秀学生培养计划第二十一期陕西省佳县支教团到佳县神泉堡革命纪念馆参观学习（274）
13. “三下乡”实践活动——参观杨家沟革命旧址及神泉堡革命纪念馆（219）
14. 三下乡｜红色地标打卡——神泉堡革命纪念馆（592）
15. “云游”红色展厅-走进佳县神泉堡革命纪念馆（14）
16. 老区新貌 | 佳县神泉堡：激活红色资源 推进绿色发展（160）
17. 神泉堡革命纪念馆成为佳县消防救援大队“爱国主义教育基地”（132）
18. 榆林福彩中心赴神泉堡革命纪念馆开展主题党日活动（266）
19. 【“七一”特辑】榆阳区委政法委党支部赴佳县神泉堡开展主题党日活动（74）
20. 区委政法委党支部赴佳县神泉堡开展主题党日活动（542）
21. 汤林带领在榆机关干部到佳县神泉堡革命纪念馆接受红色教育（1308）
22. 管理工程系师生党员赴佳县神泉堡革命纪念馆、杨家沟革命旧址开展主题教育活动（194）
23. 解锁博物馆的力量：在历史的星屑中诗意栖居，让文化遗产焕发新生！
24. （2299）
25. 【党日活动】煤业公司第五党支部赴佳县神泉堡革命纪念馆开展主题党日活动（365）
26. 五一不远游！在榆林来一场说走就走的旅行~（9）
27. 【佳县文化馆】基层全民艺术普及服务提质增效——神泉堡革命纪念馆（179）
28. 红旗漫卷——陕西革命旧址云上展之八十九：神泉堡中共中央驻地旧址（68）
29. 关于佳县神泉堡纪念馆恢复开放的公告（141）
30. 府谷县党外人士代表赴佳县神泉堡革命纪念馆参观学习（961）
31. 佳县神泉堡村：这里有革命纪念馆，又出了清华大学校长（30）
32. “扭转乾坤”的佳县神泉堡（204）
33. 赴塞上之约！领略长城黄河风光，畅游榆林“钻石线路”！（13）
34. 『学党史』榆林“红色之旅”来了，有你想看的地方吗？（53）
35. 榆林“红色之旅”来了，有你想看的地方吗？（5029）
36. ＂山水葭州，康养胜地＂佳县等你来！（6296）
37. 榆林市道协组织参观神泉堡革命纪念馆（71）
38. 榆林市农产品质量安全中心推动党的二十大精神在佳县乌镇张家沟村落地开花（37）
39. 【视频新闻】佳县组织开展纪念“神泉号令”“三大纪律八项注意”发布75周年暨党员干部廉政教育活动（36）
40. 佳县东方红和神泉堡纪念馆10月12日作风建设专题学习会议（45）
41. 【今日头条】佳县组织开展纪念“神泉号令”“三大纪律八项注意”发布75周年暨党员干部廉政教育活动（625）
42. 2022年佳县青少年社会实践夏令营活动圆满结束啦！（250）
43. 西科大高新“蒲公英支教队”| 神泉堡革命纪念馆打卡（140）
44. 佳县神泉堡革命纪念馆暂停对外开放的公告（32）
45. 红船破浪：神泉堡革命旧址（132）
46. 榆林市老龄健康与计划生育家庭发展服务中心组织党员干部赴佳县神泉堡革命纪念馆开展党史学习教育（98）
47. 榆林市道协组织参观神泉堡革命纪念馆（117）
48. 【建党百年】榆林红色景点：​神泉堡革命旧址（109）
49. 三下乡 | 神泉堡革命纪念馆宣讲（262）
50. 佳县神泉堡革命纪念馆暂停对外开放公告（331）
51. 佳县神泉堡革命纪念馆——省级爱国主义教育基地（210）
52. “百年党史树初心 红色基因代代传”—神泉堡革命纪念馆（303）
53. 党史学习教育｜追寻红色印记 讲好党史故事⑩走进佳县神泉堡革命纪念馆（400）
54. 佳县这个地方收藏着8000多枚毛泽东像章（679）
55. 学党史 | 魏墙煤业公司党委赴神泉堡革命纪念馆开展主题党日活动（235）
56. 学党史之走进佳县神泉堡革命纪念馆（190）
57. 红色遗迹寻访 | 榆林高科检测公司党支部走进佳县神泉堡革命纪念馆，探寻红色足迹（398）
58. 榆阳区军干所赴佳县神泉堡革命纪念馆开展“学党史 悟思想 重走转战陕北路”主题党日活动（54）
59. 横山区妇联赴佳县神泉堡革命纪念馆开展“学党史、悟思想、办实事、开新局”主题党日活动（441）
60. 红色起点·神泉堡革命纪念馆（56）
61. 红色遗迹寻访 | 集团装饰公司党支部走进佳县神泉堡革命纪念馆，追寻党的足迹（2259）
62. 党史学习教育￨佳县神泉堡革命纪念馆（273）
63. 名州镇赴佳县神泉堡革命纪念馆开展主题党日活动。（240）
64. 补浪河中学“神泉堡革命纪念馆、佳县赤牛坬民俗村”研学旅行活动纪实（495）
65. 【党史学习教育】▎米脂农商银行机关党员赴佳县神泉堡革命纪念馆参观学习（211）
66. 公司党委组织开展“百年党史树初心 红色基因代代传”—神泉堡革命纪念馆实地践学活动（263）
67. 【党史学习】信息化运维分公司：走进佳县神泉堡革命纪念馆 缅怀光辉历史（382）
68. 榆林市税务局机关团委赴佳县神泉堡革命纪念馆开展党史学习教育（715）
69. 【童心向党】佳县第一小学参观神泉堡革命纪念馆暨拉练活动（586）
70. 红色印记•榆林 | 神泉堡革命旧址（121）
71. 教育整顿｜佳县公安局交警大队赴神泉堡革命纪念馆开展党史学习教育活动（973）
72. 我要投稿（第二期）——参观佳县神泉堡革命纪念馆有感（88）
73. 榆林市化学化工学会组织党员参观佳县神泉堡革命历史纪念馆（36）
74. 榆林红色遗址——神泉堡革命纪念馆（145）
75. 正式揭牌！佳县神泉堡革命纪念馆成为省级爱国主义教育基地（180）
76. 清爽佳县，休闲胜地！够放松、够休闲、够好玩......选一个目的地出发吧！（1529）
77. 佳县神泉堡革命纪念馆举行省级爱国教育基地揭牌仪式（391）
78. 暑假去哪里度假---来佳县避暑吧（9893）
79. 佳县文旅局组织入党积极分子赴神泉堡革命纪念馆培训学习（31）
80. 榆神分公司组织党员赴佳县神泉堡革命纪念馆举办主题党日活动（49）
81. 佳县神泉堡革命纪念馆被命名为陕西省爱国主义教育基地（1031）
82. 佳县神泉堡革命纪念馆观后感（32）
83. 【不忘初心、牢记使命】市政府党组赴佳县神泉堡革命纪念馆开展革命传统教育（1208）
84. 【党员服务站】中文学生第二党支部赴榆林佳县神泉堡革命纪念馆暑期实践活动日 神泉堡中共中央驻地旧址参观学习（91）
85. 【转战故事】转战陕北第二十七站：佳县神泉堡（31）
86. 红色革命教育基地——佳县神泉堡革命旧址（91）
87. 佳县神泉堡革命纪念馆讲解员招聘（434）
88. 神木市工商联赴佳县神泉堡革命纪念馆接受红色教育（15）
89. 免费！免费！免费！你没看错，榆林这些景点都免费！（8886）
90. 黄河边上的这个小县城，今生别错过！（1.1万）
91. 法院新闻--佳县法院组织全体干警参观神泉堡革命纪念馆并举行“九九”重阳登高活动（390）
92. 佳县公安局指挥中心民警在神泉堡革命纪念馆进行红色教育（105）
93. 榆横供电分公司赴佳县神泉堡革命纪念馆参观学习（94）
94. 【大美榆林】佳县神泉堡革命纪念馆（365）
95. 佳县神泉堡革命纪念馆（32）
96. 【行游榆林】佳县神泉堡革命纪念馆（1029）
97. 榆林市公共就业和人才服务中心赴佳县神泉堡革命纪念馆参观学习（2011）
98. 榆林沿黄印象第三站——佳县（2033）
99. 山西省离石区西属巴街道考察组来佳县神泉堡革命纪念馆参观学习（131）
100. 追溯红色记忆 走进佳县神泉堡革命纪念馆（50）
101. 为你揭开佳县的神秘面纱……（2114）
102. 榆林市市委书记胡志强一行来到佳县神泉堡革命纪念馆（64）
103. 佳县神泉堡革命纪念馆简介（69）
104. 佳县神泉堡革命纪念馆介绍（62）
105. 佳县革命圣地——神泉堡革命纪念馆（24）
106. 每日推荐 | 佳县神泉堡革命纪念馆（242）
107. 中共中央旧址-佳县神泉堡革命纪念馆（32）

**绥德县革命历史纪念馆**

1. 文明单位|绥德县革命历史纪念馆被共青团陕西省委命名为“陕西省青少年教育基地”（430）
2. 绥德县革命历史纪念馆被共青团陕西省委命名为“陕西省青少年教育基地”（238）
3. 陕西日报访绥德革命纪念馆馆长蒲鹏（108）
4. 绥德县革命历史纪念馆入选长城主题国家级旅游线路（752）
5. 防风险 保安全丨绥德县革命历史纪念馆开展消防安全培训（243）
6. 喜迎二十大 全力保安全 | 绥德革命历史纪念馆开展国庆节前专项大检查（132）
7. 作风建设 | 绥德县革命历史纪念馆召开作风建设专项行动动员会（196）
8. 绥德革命历史纪念馆参加2022年陕西省革命文物融入高校思政教育培训 继续办好“纪念馆里的思政课”（435）
9. 绥德革命历史纪念馆派员参加2022年陕西省革命文物融入高校思政教育培训班（）349
10. 绥德县革命历史纪念馆召开安全生产整治“百日行动”工作动员部署会（242）
11. 陕西革命旧址（二十）：绥德革命历史纪念馆（18）
12. 彭德怀、李井泉、陆定一等革命先辈亲属代表参观绥德革命历史纪念馆（263）
13. 我和绥德县革命历史纪念馆 ▎讲述人：何倩倩（145）
14. 爱国主义教育基地|绥德县革命历史纪念馆“七一”期间迎来客流量高峰（75）
15. 我和绥德县革命历史纪念馆 ▎讲述人：汪瑶（299）
16. 我和绥德县革命历史纪念馆 ▎讲述人：曹旋旋（276）
17. 我和绥德县革命历史纪念馆 ▎讲述人：刘贝贝（381）
18. 文明单位|绥德县革命历史纪念馆组织广大青年集中观看学习庆祝中国共青团成立100周年大会直播（59）
19. 我和绥德县革命历史纪念馆 ▎讲述人：马义（243）
20. 绥德县革命历史纪念馆组织广大青年集中观看学习庆祝中国共青团成立100周年大会直播（166）
21. 我和绥德县革命历史纪念馆 ▎讲述人：王转（253）
22. 我和绥德县革命历史纪念馆 ▎讲述人：闫慧（254）
23. 世界知识产权日 | 保护知识产权 绥德县革命历史纪念馆在行动（85）
24. 我和绥德县革命历史纪念馆 ▎讲述人：武美丽（311）
25. 我和绥德县革命历史纪念馆 ▎讲述人：霍高换（375）
26. 我和绥德县革命历史纪念馆 ▎讲述人：郭彩平（403）
27. 【通知公告】绥德县革命历史纪念馆恢复开放公告（100）
28. 绥德县革命历史纪念馆恢复开放公告（486）
29. 我和绥德县革命历史纪念馆 ▎讲述人：田婷（295）
30. 位于学子大道的绥德革命纪念馆，其周边建筑立面及道路，将进行景观提升改造（832）
31. 何荷：我和绥德县革命历史纪念馆（91）
32. 我和绥德县革命历史纪念馆 ▎讲述人：何荷（402）
33. 我和绥德县革命历史纪念馆 ▎讲述人：刘欢（459）
34. 陆焕利和绥德县革命历史纪念馆的故事（238）
35. 我和绥德县革命历史纪念馆 ▎讲述人：陆焕利（461）
36. 绥德县革命历史纪念馆、博物馆、子洲图书馆暂停对外开放（97）
37. 绥德县革命历史纪念馆通知！（128）
38. 绥德县革命历史纪念馆暂停开放的通知​！（549）
39. @绥德人 郝家桥旧址 革命历史纪念馆恢复对外开放（398）
40. 绥德县革命历史纪念馆给您拜年啦！（991）
41. 绥德县革命历史纪念馆的2021年！（439）
42. 关于绥德县革命历史纪念馆暂停开放的通知​！（548）
43. 绥德县革命历史纪念馆积极参加中共榆林市委组织部关于讲解员现场教学技能竞赛（335）
44. 以交流促进步 | 绥德县革命历史纪念馆开展馆内交流学习活动（681）
45. 绥德县革命历史纪念馆和庞亮伟、刘翻获得市上表彰啦（871）
46. 文明实践基地|绥德县革命历史纪念馆“小小志愿讲解员”培训班开课啦（79）
47. 绥德县革命历史纪念馆“小小志愿讲解员”培训班第一期开课啦！（554）
48. 绥德县革命历史纪念馆——“小小讲解员”海选记（1152）
49. 疫情防控丨绥德县革命历史纪念馆通知（745）
50. 绥德县革命历史纪念馆关于加强近期疫情防控相关工作的通知（304）
51. 绥德：老兵张海碧向绥德县革命历史纪念馆捐赠藏品（754）
52. 老兵张海碧向绥德县革命历史纪念馆捐赠了一盏小马灯​（393）
53. “小马灯，映初心” | 老兵张海碧向绥德县革命历史纪念馆捐赠藏品（763）
54. 【旅游文化】国庆 | 近万名游客来绥德革命历史纪念馆接受爱国主义教育（107）
55. 国庆 | 近万名游客来绥德革命历史纪念馆接受爱国主义教育（406）
56. 关于绥德革命历史纪念馆闭馆的公告（1515）
57. 招募 | 欢迎加入绥德县革命历史纪念馆“小小志愿讲解员”（1560）
58. 绥德县革命历史纪念馆 学习习近平总书记来陕考察重要讲话精神（232）
59. 绥德县革命历史纪念馆全力做好“十一”黄金周旅游接待准备工作（360）
60. 绥德县革命历史纪念馆的曹垚和田婷，获奖啦（773）
61. 绥德县革命历史纪念馆参加榆林市红色故事讲解员大赛获佳绩（309）
62. 绥德县革命历史纪念馆举行文物捐赠仪式（2038）
63. 参观绥德县革命历史纪念馆防疫须知（355）
64. 寻找1400多名绥德籍烈士证明书， ​绥德县革命历史纪念馆向社会征集线索（332）
65. 文明单位风采|绥德县革命历史纪念馆历开展“学党史 办实事”主题党日志愿服务活动（463）
66. 绥德县革命历史纪念馆走进绥德县东门滩社区开展“学党史 办实事”志愿服务主题党日活动（185）
67. 绥德县革命历史纪念馆开展“国际博物馆日”宣传活动（59）
68. 点亮中国精神|绥德革命历史纪念馆（100）
69. 【公告】绥德革命历史纪念馆开馆公告（211）
70. 绥德革命历史纪念馆开馆公告（488）
71. 绥德县革命历史纪念馆召开疫情防控工作会（198）
72. 绥德革命纪念馆开工建设（915）
73. 绥德革命纪念馆开工建设啦！是榆林首座大型综合性历史性纪念场馆（1393）
74. 绥德革命历史纪念馆再添一牌（331）
75. 绥德县革命历史纪念馆全体党员干部踊跃捐款，支持新冠肺炎防疫工作（25）
76. 绥德县革命历史纪念馆开展春季爱国卫生运动，助力疫情防控（109）
77. 文明单位风采丨绥德县革命历史纪念馆（609）
78. 疫情防控：绥德县革命历史纪念馆党员干部进社区工作纪实（374）
79. 疫情防控 安全管理不打烊 —— 绥德县革命历史纪念馆工作纪实（30）
80. 绥德县革命历史纪念馆党员进社区 消毒防疫保健康（75）
81. 绥德县革命历史纪念馆扎实推进疫情防护工作（133）
82. 绥德：革命历史纪念馆、汉画像石馆等暂停开放（153）
83. 绥德县革命历史纪念馆入选全国红色旅游经典景区名录（128）
84. 绥德县革命历史纪念馆召开“不忘初心 牢记使命”主题教育动员会（19）
85. 三下乡/ 文学院 绥德县革命历史纪念馆（26）
86. 绥德县革命历史纪念馆扎实推进扫黑除恶专项斗争宣传工作（20）
87. 绥德县革命历史纪念馆召开扫黑除恶专项斗争工作推进会（28）
88. 绥德县革命历史纪念馆召开助力脱贫攻坚动员大会（33）
89. 绥德县革命历史纪念馆来了75岁志愿者，TM们只为做这件事……（15）
90. 绥德县革命历史纪念馆来了13位志愿者，TM们只为做这件事……（345）
91. 绥德县革命历史纪念馆开展“纪念建党96周年暨中共中央转战陕北70周年”主题展览活动（40）
92. 【文明旅游】绥德县革命历史纪念馆入选全国红色旅游经典景区名录（18）
93. 绥德县革命历史纪念馆（21）
94. 绥德县革命历史纪念馆简介（31）
95. 绥德革命历史纪念馆免费开放！抓紧时间！（241）
96. 带你走进绥德县革命历史纪念馆（155）
97. 好事】绥德县革命历史纪念馆被命名为“陕西省青少年教育基地”（312）
98. 【红色景点】绥德县革命历史纪念馆（141）
99. 绥德县革命历史纪念馆被命名为 “陕西省青少年教育基地”（104）
100. 绥德县革命历史纪念馆举办纪念抗战胜利70周年美术小品展（183）

**宝鸡市红色旅游系列景区(凤县两当起义纪念地（18）,眉县扶眉战役纪念馆（159）)**

**凤县两当起义纪念地**

1. 【热烈庆祝党的二十大胜利召开】陇山全域旅游背景下谈华亭文化旅游深度融合与创新（16）
2. 【红色旅游】陕西省13个红色旅游景区列入全国红色旅游经典景区名录（351）
3. 纪念“两当兵变”90周年理论研讨会在凤县召开（1405）
4. 『凤县革命纪念馆：闭馆不闭展 网上看展览』（一）两当兵变策源地旧址（162）
5. 【心中的歌儿唱给党】凤县革命纪念馆里的红色记忆——“两当兵变”（62）
6. 【特别关注】《全国红色旅游经典景区名录》公布300处景区入选 有你家乡吗（498）
7. 三下乡 | 凤县革命纪念馆（220）
8. 参观凤县革命纪念馆，了解两当兵变的历史，策划于陕西发生于甘肃（5）
9. 2021年裴家埪党支部重温红色历史党史学习活动（394）
10. 红色起点·宝鸡市红色教育基地（114）
11. 建党百年之际，带你畅游西北大地上的红色经典景区（134）
12. 百集微视 | 两当兵变策源地（凤州）的红色故事（第39集）（187）
13. 学党史守初心 传承红色基因 —— 参观学习凤县革命纪念馆 两当兵变纪念馆（127）
14. 红色起点·两当兵变纪念馆（109）
15. 两当：传承红色好基因 做活产业大文章（500）
16. 县政协组织部分委员赴甘肃两当兵变纪念馆和凤县开展学习教育等活动（199）
17. 县政协组织部分政协委员赴甘肃两当兵变纪念馆和凤县开展学习教育等活动（481）
18. 读百年党史•谱发展新篇丨探寻宝鸡红色记忆：来自凤县革命纪念馆里的红色记忆——“两当兵变”（41）
19. 山有水，吃喝可待！相约凤县看“花花世界”（2）
20. 有山有水，吃喝可待！相约凤县看“花花世界”（518）
21. 2021年凤县文化旅游十大亮点，七彩凤县诚邀您~（854）
22. 推荐丨2021年凤县文化旅游十大亮点，七彩凤县诚邀您~（上）（306）
23. 荐丨2021年凤县文化旅游十大亮点，七彩凤县诚邀您~（883）
24. 凤县旅游推介走进两当（435）
25. 【跟我学党史】两当起义的经过及其历史意义（402）
26. 2021凤县文化旅游十大亮点，七彩凤县诚邀您~（765）
27. 凤县文化旅游暨招商引资（重庆）推介会精彩举办 引资11.95亿元（104）
28. 2021年凤县文化旅游十大亮点，等您打call！（358）
29. 名胜古迹】甘肃两当兵变纪念馆（65）
30. 三秦大地上的红色印记 ——“两当兵变”策源地凤州（143）
31. 三秦大地上的红色印记丨“两当兵变”策源地凤州（397）
32. 【奋斗百年路 启航新征程】凤县革命纪念馆留存的红色记忆（368）
33. 凤县副县长穿羌服为家乡代言，向全国推介大秦岭的会客厅！（4606）
34. 2021年凤县开启旅游专列常态化，等春也等你！（1476）
35. 被中央表扬！凤县蝉联“全国县级文明城市”（1229）
36. 太凤高速有多美？旅游线路大推荐，带您打卡最美秋色（12）
37. 诗画关山•心仪华亭”关山历史文化研究学术研讨会论文之十九——陇山全域旅游背景下谈华亭文化旅游深度融合与创新（76）
38. 【基层直通车】陕西局一五九处党支部组织党员赴凤县革命纪念馆开展主题党日活动（238）
39. 缅怀革命先烈 接受红色洗礼------两当县综合执法局党支部赴凤县革命纪念馆开展主题党日活动（306）
40. 地名故事】听说张家窑一名，在凤县、两当县的中共党史上赫赫有名（436）
41. 激动！亮相国庆阅兵式的宝鸡造装备“回家” ！这些都是宝鸡的骄傲（1436）
42. 骄傲~参加国庆大阅兵的宝鸡军人和特战装备回家啦！这些都是宝鸡的......（7433）
43. 陕西人都来这里旅游打卡了！陕西“红色旅游”受热捧（2691）
44. 年轻人都来这里旅游打卡了！陕西“红色旅游”受热捧（219）
45. 【党日活动】两当兵变纪念馆党支部赶赴凤县革命纪念馆开展主题党日活动（264）
46. 追溯革命历史 接受红色洗礼 | 凤翔分局组织党员参观凤县两当兵变纪念馆（234）
47. 7月6日-7日（周末二日游）；凤县羌族村寨、紫柏山、两当兵变纪念馆2日游；368元/位！（65）
48. 7月6日-7日（周末）凤县羌族村寨、紫柏山、两当兵变纪念馆2日游；368元/位！（11）
49. 又一项省级称号拿到手！凤县革命纪念馆厉害了（1786）
50. 感怀峥嵘岁月，接受红色洗礼——原后勤党支部赴两当兵变纪念馆、凤县革命纪念馆参观学习（83）
51. 凤县革命纪念馆参加“纪念两当兵变87周年系列活动”（237）
52. 重磅好消息！凤县喜获“中国康养休闲旅游名县”称号（643）
53. 凤县革命纪念馆闭馆公告（825）
54. 凤县：发展全域旅游 打造别样西北小城（993）
55. 【红色传奇 · 忆征程】缅怀革命先烈 ，追寻红色记忆。凤县革命纪念馆开馆两年：铭记历史重在传承......（221）
56. 【两当旅游】两当兵变纪念馆2018年国庆假期正常开放（121）
57. 市地志办机关党支部赴凤县、两当县革命教育基地开展主题党日活动（138）
58. 宝鸡亮宝——从凤县开始的“两当兵变”之二在（73
59. 25个精彩瞬间燃爆2017宝鸡旅游，点开有门票！（918）
60. 宝鸡这三处获最佳生态奖，看看有没有你的家乡！（3399）
61. 今秋最“红”旅行在凤县！追忆红色革命，看红叶尽染！（366）
62. 饮山泉、吃泡面、住帐篷！凤县三小伙骑行千里，重温两当兵变红色历史（653）
63. 在宝鸡拥有这张卡的小伙伴们有福了！可“刷脸”免费游这些景区！（10）
64. 又上榜了！凤县被全国红色旅游点名了（1276）
65. 《全国红色旅游景点景区名录》公布 凤县两当起义纪念地榜上有名（670）
66. 《全国红色旅游经典景区名录》发布，泾阳安吴青训班入选！（683）
67. 安吴青训班入选全国红色游经典景区，红色泾阳欢迎您！（2029）
68. 中省文化名人、陕甘川宁媒体老总眼里的凤县......（631）
69. 今天凤县最红！所有人都在关注85年前的这件事（2910）
70. 走进秦岭花谷 发现红色凤县|两当起义为凤县播下红色火种（500）
71. 陕西省13个红色旅游景区列入全国红色旅游经典景区名录（21）
72. 低调的凤县又一次让我们震惊，已被围观！（3269）
73. 陕西日报专版报道凤县：凤县这块革命热土有着许多光荣的记忆（1742）
74. 【游记】凤县革命纪念馆工作人员到两当兵变纪念馆考察学习（319）
75. 额滴神啊！短短三天，竟然有3.84万人到凤县来耍！（1617）
76. 从红色凤县出发 重走不能被忘却的红色革命线路（224）
77. 四天三晚重走红色革命路，红色凤县一路向前！（1265）
78. “追寻凤县光辉历程 重走红色革命路线”行军进行中......（888）
79. 什么是“两当兵变”？让我们从凤县出发去看看（554）
80. 西洽会 | 张帆县长推介凤县旅游（33）
81. 【西洽会】2016凤县旅游看什么？县长张帆这样说：（949）
82. 西洽会|凤县县委书记、县长向省内外客商、游客全面推介凤县，都说了些啥（1365）
83. 凤县县长邀请全省人民到凤县过大年（3052）
84. 秦岭深处的红色火种——习仲勋在凤县的故事（2591）
85. 中国红色文物保护专项基金会来凤考察（18）

**眉县扶眉战役纪念馆**

1. 追寻红色足迹 赓续红色血脉——【宝鸡红色资源】推介（二）扶眉战役纪念馆（113）
2. 【红色资源推介】扶眉战役纪念馆（40）
3. 仪光赤心2022第五站丨宝鸡扶眉战役纪念馆（66）
4. 扶眉战役纪念馆缅怀革命先烈（875）
5. 秦都区妇联赴扶眉战役纪念馆开展主题党日活动（278）
6. 【眉县旅游】“七一”来临之际，社会各界人士来扶眉战役纪念馆开展活动，催热眉县红色旅游（106）
7. 扶眉战役纪念馆近期工作动态（110）
8. 金台区卫监所赴扶眉战役纪念馆开展主题党日活动（187）
9. 眉县扶眉战役纪念馆讲解员在全省干部教育培训现场教学点讲解员培训班暨现场教学技能比赛中获奖（104）
10. 陕西革命旧址云上展 (二十九) | 扶眉战役纪念馆（62）
11. 陕西省退役军人事务厅来我县扶眉战役纪念馆开展“2022·奋进·清明祭英烈”代祭扫暨主题党日活动（385）
12. 扶眉战役烈士陵园（纪念馆）业务提升正当时（224）
13. 打卡廉政教育基地 | 扶眉战役纪念馆（2408）
14. 西安市审计局组织退休党员干部赴扶眉战役纪念馆开展党史学习教育（245）
15. 【三秦文学】 张栓平：【 扶眉战役纪念馆参观记】（散文）（172）
16. 团建 | 同创文化开展百年党史教育活动，瞻仰扶眉战役纪念馆（185）
17. 三下乡 | 扶眉战役纪念馆（189）
18. 扶眉战役：一座纪念馆传承的红色基因（339）
19. 咸阳市统建办组织党员干部赴眉县扶眉战役纪念馆开展党史学习教育活动（219）
20. 廉播 · 西安 | 市纪委监委十四运监督办临时党支部赴扶眉战役纪念馆开展党史教育活动（582）
21. 参观扶眉战役纪念馆采风作品（3）（248）
22. 参观扶眉战役纪念馆作品（一）（188）
23. 国家市场监管总局食品生产司一行来扶眉战役纪念馆开展纪念活动（630）
24. 团市委机关党支部赴扶眉战役纪念馆开展主题党日活动（938）
25. 市人大常委会组织机关党员干部参观扶眉战役纪念馆（113）
26. 扶眉战役纪念馆 | 一封永远无法到达的信（219）
27. 公司工会赴扶眉战役纪念馆开展党史学习教育活动（946）
28. 港口局党支部赴扶眉战役纪念馆开展主题党日活动（52）
29. 走进扶眉战役纪念馆开展“学党史、祭英烈、铭初心、践使命”主题教育活动​（35）
30. 培训中心党支部赴扶眉战役纪念馆开展党建活动（49）
31. 扶眉战役纪念馆参观记（550）
32. 赴扶眉战役纪念馆开展党史学习教育主题党日活动（524）
33. 市投资局组织全体党员干部赴扶眉战役纪念馆开展主题党日活动（466）
34. 点燃初心使命 凝聚奋进力量——区委组织部机关党员参观扶眉战役纪念馆（94）
35. 未央区审计局赴扶眉战役纪念馆开展主题党日活动（54）
36. 赴“扶眉战役纪念馆”开展 “学党史、守初心、铸师魂”主题党日活动（1093）
37. 铜川书画院党支部 赴扶眉战役纪念馆开展主题党日活动（87）
38. 市档案局（馆）赴扶眉战役纪念馆开展主题党日活动（327）
39. 示范区人社局党员干部赴扶眉战役纪念馆学党史悟初心（1117）
40. 中共咸阳市环境监测站党支部赴扶眉战役纪念馆开展“学习百年党史弘扬英烈精神”主题党日活动（297）
41. 杨凌交巡警支队组织第二批党员参观扶眉战役纪念馆（1781）
42. 影视动画系党总支赴扶眉战役纪念馆开展主题党日活动（241）
43. 缅怀革命先烈 传承红色基因|所（公司）党委开展“走进扶眉战役纪念馆”活动（1012）
44. 庆祝建党百年【扶眉战役纪念馆2】（298）
45. 【工作动态】——扶眉战役烈士陵园（纪念馆）本周活动（122）
46. 红色起点·扶眉战役纪念馆（174）
47. 学党史、悟思想、办实事、开新局｜重温感天动地的扶眉战役（410）
48. 区政协赴扶眉战役纪念馆开展党史学习教育主题实践活动（492）
49. 局机关六个党支部在扶眉战役纪念馆开展主题党日活动（582）
50. 百篇红色金典I第四十三篇 扶眉战役纪念馆（196）
51. 党史学习教育 | 扶眉战役纪念馆参观学习（169）
52. 报名参见活动啦丨走进扶眉战役纪念馆（770）
53. 技工学校党支部赴扶眉战役纪念馆开展主题党日活动（540）
54. 【思想强基】扶眉战役纪念馆参观学习（1245）
55. 网络中国节·清明节丨​扶眉战役纪念馆（132）
56. 市级社会组织党员赴扶眉战役纪念馆开展公祭活动（258）
57. 局机关四个党支部赴扶眉战役纪念馆开展主题党日活动（724）
58. 西安市人防系统指挥通信业务骨干前往扶眉战役纪念馆开展主题党日活动（290）
59. 县政协机关赴扶眉战役纪念馆开展党史教育活动（149）
60. 扶眉战役纪念馆红色旅游公交专线开通啦！（5）
61. 党史学习教育丨省生态环境厅组织党员干部瞻仰扶眉战役纪念馆（1081）
62. 扶眉战役纪念馆关于2021年春节期间开放时间的公告（248）
63. 第三十期社会组织“DIY党课”在扶眉战役纪念馆举办（24）
64. 党建园地 | 管理联合党支部开展“走进扶眉战役纪念馆”主题党日活动（3）
65. 扶眉战役纪念馆成为“陕西省离退休干部教育基地”（629）
66. 我市扶眉战役纪念馆成为“陕西省离退休干部教育基地”（776）
67. 陕西省离退休干部教育基地”在扶眉战役纪念馆挂牌（27）
68. 全体党员赴扶眉战役纪念馆，开展主题党日活动（306）
69. 【教学动态】陕西社会主义学院扶眉战役纪念馆现场教学点揭牌（233）
70. 离退休党员群众参观参观扶眉战役纪念馆（374）
71. 陕西省军休中心赴扶眉战役纪念馆开展主题党日活动（560）
72. 市致公党直属一支部赴扶眉战役纪念馆开展社会主义核心价值观教育（56）
73. 陕西省爱国主义教育基地之四：扶眉战役纪念馆（34）
74. 扶眉战役纪念馆部分区域恢复对外开放，具体安排点开知晓！（63）
75. @亲们 扶眉战役纪念馆恢复开放啦~（516）
76. 扶眉战役纪念馆临时性闭馆公告（99）
77. 通知：扶眉战役纪念馆继续临时性闭馆（382）
78. 陕西眉县扶眉战役纪念馆景区被命名为国家AAAA级旅游景区（124）
79. 公示 |陕西拟新增12家国家4A级旅游景区，宝鸡扶眉战役纪念馆名列其中（1188）
80. 93岁老兵参观扶眉战役纪念馆（467）
81. 县委宣传部全体党员干部赴扶眉战役纪念馆、张载祠开展主题党日活动（135）
82. 关于征集扶眉战役纪念馆党性教育基地核心课程的公告（106）
83. 省市领导来眉瞻仰扶眉战役烈士纪念碑、参观纪念馆（2185）
84. 扶眉战役纪念馆党性教育基地（扶眉干部学院）召开规范化建设工作推进会（155）
85. 扶眉战役纪念馆顺利通过全省党性教育 基地质量评估验收（306）
86. 陕西省廉政教育示范基地巡礼（十）——扶眉战役纪念馆（1470）
87. 第一党支部组织党员参观扶眉战役纪念馆（178）
88. 公告，扶眉战役纪念馆闭馆公告！（1155）
89. 扶眉战役烈士陵园（纪念馆）闭馆公告（506）
90. 市长惠进才调研扶眉战役纪念馆维修改造工作（908）
91. 秦明奇调研扶眉战役纪念馆改扩建工作（336）
92. 扶眉战役纪念馆维修改造正式启动，宝鸡红色旅游时代来临！（893）
93. 贾村镇中心小学党支部 赴扶眉战役纪念馆开展主题党日活动（389）
94. 入伍新兵齐聚扶眉战役纪念馆 上好入伍“第一课”（745）
95. 扶眉战役纪念馆里缅怀先烈（274）
96. 【学院动态】纪念碑前忆英灵，观扶眉战役纪念馆（203）
97. 市安监局组织干部职工参观扶眉战役纪念馆（191）
98. 【旅游】全国红色旅游经典景区—扶眉战役纪念馆（185）
99. 【超级暑假】进扶眉战役烈士纪念馆[四二班暑期社会实践日]（591）
100. 红色旅游|全国红色旅游经典景区—扶眉战役纪念馆（14）

**咸阳市泾阳县安吴青训班革命旧址（14）**

1. 祝贺！咸阳市9家单位被命名为陕西省青少年教育基地（562）
2. 【关注】泾阳县安吴镇入选陕西省首批乡村振兴典型案例（158）
3. 国庆保电 | 咸阳公司：咸小电带你“云”逛咸阳（315）
4. 安吴青训班 | 追寻红色记忆，铭记光辉历程（18）
5. 「追光寻影•泾阳安吴青年训练班旧址」21.安吴青年训练班旧址，为抗日青年运动的蓬勃发展做出了卓著的贡献。（8）
6. 重温红色岁月 弘扬革命精神（94）
7. 革命青年的熔炉、中国青年运动史上的丰碑——机电工程学院组织教师党员赴安吴青训班开展主题党日活动（61）
8. 云游红色文化教育基地 | 咸阳安吴青训班革命旧址（577）
9. 【廉政教育】安吴堡战时青年训练班革命旧址（175）
10. 庆祝建团百年·历史资料 | 第四期：安吴青训班革命旧址（66）
11. 安吴青年训练班纪念馆重新布展，重磅开馆~~（764）
12. 【建团百年历史资料】之四：安吴青训班革命旧址（15）
13. 打卡廉政教育基地 | 安吴堡战时青年训练班革命旧址（1754）
14. 陕西革命旧址(六):安吴堡战时青年训练班革命旧址（23）
15. 打卡革命“中国红”|安吴青训班革命旧址（62）
16. 金秋赏菊，在这里→（366）
17. 咸阳“红色之旅”来啦！一起打卡吧！（3729）
18. 花好月圆话咸阳丨好消息！泾阳安吴青训班纪念馆布展一新，10月1日重装开馆！（134）
19. 泾阳：安吴青训班纪念馆布展一新，10月1日重装开馆！（80）
20. 安吴青训班纪念馆布展一新，10月1日重装开馆！（1820）
21. 咸阳地区庙宇中的革命历史旧址（上）（123）
22. 那年的你｜安吴青训班吹响集结号（109）
23. 安吴青训班简介（129）
24. 【泾检快讯】省院第十检察部到泾阳安吴青训班旧址开展主题党日活动（98）
25. 忆往昔峥嵘岁月，继续踏上红色旅程（585）
26. 红色起点·安吴青训班革命旧址（83）
27. 探寻红色文化！众多媒体纷纷聚焦泾阳 ……（400）
28. 【党史故事】安吴青训班旧址（47）
29. 渭阳西路街道彩虹社区党支部开展“探寻红色足迹，传承革命精神”安吴青训班参观活动（104）
30. 汲取红色营养 承载青春使命 |学校组织学生赴安吴青训班革命旧址参观学习（135）
31. 咸阳—铜川—庆阳三市联合推出9条陕甘边红色旅游精品线路（460）
32. 铜·线路丨咸阳-铜川-庆阳三市联合推出9条陕甘边红色旅游精品线路（728）
33. 咸阳：走在“一路向阳”的大道上（179）
34. 陕西外事 | 学党史、强信念、励斗志、跟党走 行政处党支部赴安吴青训班革命旧址开展主题党日活动（158）
35. 红色沃土上的初心使命--咸阳在党史学习教育中汲取前进力量（10）
36. ​咸阳：红色沃土上的初心使命（445）
37. 咸阳市：在红色沃土上砥砺初心担使命！（98）
38. 咸阳：在红色沃土上砥砺初心担使命！（248）
39. 陕西日报全媒体行动走进咸阳丨感受小城故“柿”慢生活（728）
40. 陕西日报看咸阳 | ​咸阳又多一处特色产业小镇（2282）
41. 打卡“红色记忆”，一起“陕”闪放光彩！娘家人又有好活动等你来！（1959）
42. 吴家堡街道毕塬路社区：参观红色革命基地 学习党史铭初心（40）
43. “党史故事现场讲”第四站——安吴青训班（792）
44. 安吴青训班：革命青年的熔炉，青运史上的丰碑（454）
45. 寻访红色地标丨我校第二十六期青年马克思主义者培训班学员赴安吴青训班革命旧址参观学习（912）
46. 2021“郑国渠杯”泾阳半程马拉松赛最新线路公布！（2489）
47. 五一攻略4.0 | 开启红色文化之旅，重温陕西的红色记忆（1576）
48. 收藏！今年五一咸阳“游”点不同，攻略来啦～（2194）
49. 西安北所组织干部民警参观安吴青训班纪念馆（565）
50. 校医院赴安吴青训班革命旧址开展党史学习教育主题党日活动（500）
51. 【社区党建】枣园西路社区开展安吴堡青训班革命旧址参观学习活动（111）
52. 【权威发布】县委常委班子到安吴爷台山亭口习仲勋革命活动旧址开展党史学习教育（507）
53. 《泾阳新闻》：县委常委班子走进安吴青训班革命旧址、云阳红色记忆展开展党史学习教育；全县新冠疫苗接种工作推进会召开（168）
54. 【金秋资讯】金秋杂志社赴安吴青训班开展党史学习教育主题党日活动（25）
55. 【党史学习教育】县委常委班子走进安吴青训班革命旧址、云阳红色记忆展开展党史学习教育（365）
56. 今年花开逛咸阳║学史力行之旅——走进泾阳安吴青训班（1007）
57. 百篇陕西红色经典|第八篇 安吴青训班纪念馆（466）
58. 咸阳市教育局@名师谈 -- 云端游名胜，双语庆大年！（4249）
59. 省院检务督察部与驻省院纪检监察组到泾阳安吴青训班旧址开展主题党日活动（182）
60. 【泾检快讯】省院检务督察部与驻省院纪检监察组到泾阳安吴青训班旧址开展主题党日活动（130）
61. 治安系党总支在泾阳县安吴青训班革命旧址开展主题党日活动（694）
62. 安吴青训班：游客同吃“国庆面” 为祖国庆生（960）
63. 喜讯！安吴青训班入选国家级抗战纪念设施、遗址名录啦~（520）
64. 保安公司党委赴安吴青训班革命旧址开展“倡廉洁、践使命”主题党日活动（414）
65. 泾阳法院党总支赴安吴青训班遗址接受革命传统教育（353）
66. 只要有这个证！咸阳这10个景区统统免费！！（2889）
67. 献礼"最可爱的人 "，咸阳10家景区推出退役军人免费游活动（384）
68. 青运史上的璀璨之星—安吴青训班（331）
69. 泾阳县入选第二批革命文物保护利用片区分县（764）
70. “红色”沃土一一泾阳（878）
71. 省委统战部副部长白慧芳一行深入安吴青训班革命旧址调研传统文化工作开展情况（430）
72. 【传承红色基因】泾阳县人民检察院在安吴青训班革命旧址开展庆祝建党99周年主题党日活动（334）
73. 泾阳县安吴青年训练班纪念馆 明日起将对外开放（921）
74. 直播预告丨泾阳融媒“云旅游”走起，带你云看最美泾阳……（1445）
75. 泾阳：用红色文化铸魂育人（315）
76. 我校第二十四期青年马克思主义者培训班学员赴安吴青训班革命旧址参观学习（1033）
77. 泾阳：中国红色记忆之城（626）
78. 泾阳安吴青训班纪念馆入选陕西100处“红色旅游地”（10）
79. 安吴青年训练班纪念馆入选“陕西100处红色旅游地” ​（528）
80. 红色旅游读咸阳：走进泾阳安吴青训班旧址（415）
81. 我院纪检干部和重点部门人员赴安吴青训班革命旧址开展学习教育活动（813）
82. 商洛市人民检察院组织党员干警赴安吴青训班旧址开展红色教育活动（116）
83. 民盟丨市二中支部到安吴堡青训班革命旧址参观学习（68）
84. 爱国奋斗，建功立业 丨商学院党委组织全体教职工党员赴安吴青训班开展实践学习活动（41）
85. 教育部发文，安吴青训班大名在列！（796）
86. 40年前的安吴青训班是个啥样子？（2111）
87. 学院师生赴泾阳县安吴青训班旧址开展主题教育活动（695）
88. 走进红色咸阳之安吴青训班（525）
89. 重温红色岁月 传承革命精神——陕西局一五九处组织党员干部参观安吴青训班革命旧址（229）
90. 我院党政支部组织党员前往“安吴青训班革命旧址”参观学习（150）
91. 九九重阳节 浓浓敬老情—我们的节日·重阳离休干部安吴青训班纪念馆学习（142）
92. 低调了2200年的陕西古县——泾阳，终于火了！（3.5万）
93. 《安吴青训班》荡气回肠，热血沸腾！（175）
94. 安吴青训班重新布展，胡乔木王有轩子女作指导！（457）
95. 胡乔木王有轩子女重回安吴青训班，这件事引起重视！（4285）
96. 安吴青训班入选全国红色游经典景区，红色泾阳欢迎您！（2029）
97. 大宅院里不朽的红色丰碑--安吴青训班（77）
98. 大西咸人最后的自留地！泾阳田园一日游~~（2.8万）
99. 安吴青训班游记（1286）
100. 泾阳安吴──旅游热土（1.3万）

**黄陵县陕甘边小石崖革命旧址（5）**

1. 【红色庆阳.遗址篇】关中苏维埃政府旧址（20）
2. 【学习党的二十大精神】行走的红色党课 别样的集中学习（690）
3. 红色教育洗礼心灵 铿锵誓言砥砺初心（79）
4. 省植保站来黄开展主题党日和绿色防控专题调研活动（66）
5. 红色传奇小石崖（21）
6. 2022年元旦，倪文东给你送电子台历来了！（1574）
7. 【特别关注】《全国红色旅游经典景区名录》公布300处景区入选 有你家乡吗（498）
8. 我在革命旧址讲党史：黄陵“小石崖”篇（2488）
9. 追寻革命足迹，传承红色精神——黄陵县小石崖革命旧址（20）
10. “陕”闪红星耀中国|走进小石崖革命旧址 感悟革命精神（16）
11. 小石崖的红色传奇故事 ​（58）
12. 王国平 | 关于对小石崖革命根据地历史贡献的探讨（ 中）（126）
13. 王国平 | 关于对小石崖革命根据地历史贡献的探讨（上）（197）
14. 建党100周年| 传承红色基因、追寻革命足迹，走进小石崖革命根据地教学基地！（759）
15. 解放军报一版头条聚焦省军区党史学习教育（1.2万）
16. 百篇陕西红色经典|第六十篇 小石崖苏区旧址（113）
17. 公司新闻｜聆听“崖壁上的党课” 传承红色革命基因（636）
18. 全国红色旅游经典景区名录（198）
19. 瑞能煤业：回忆军旅岁月 争当建功标兵（589）
20. 全国红色旅游经典景区名录公布 300处景区入选（12）
21. 【地理之美】全国红色旅游经典景区名录（81）
22. 红色印记之小石崖革命根据地旧址（33）
23. 地球与空间科学学院赴延安开展思想政治实践课程（555）
24. 开展游击战争创建陕甘边根据地（中）（174）
25. 开辟以南梁为中心的陕甘边革命根据地（二）（91）
26. 【百年党史•百日学】 小石崖革命苏区和中宜苏区的开辟（122）
27. 铜川市陕甘边革命根据地旧址列入全国红色旅游经典景区名录（559）
28. 忆陕甘边根据地（下）（179）

**靖边县小河会议旧址（33）**

1. 《这里是榆林》之红色榆林（112）
2. 踏寻革命足迹 重温红色故事 吴堡法院到靖边小河会议旧址开展主题党日活动（161）
3. 张晓光在定边 靖边 子洲调研（6421）
4. 红色信使寻访团第五站｜小河会议旧址（179）
5. 三下乡｜追寻红色足迹（二) ——靖边县小河会议旧址（107）
6. 靖边县退役军人事务局 召开“荣耀靖边 情暖老兵”八一座谈会（139）
7. 榆靖分公司党委理论学习中心组在靖边小河会议旧址参观学习（155）
8. 靖边事儿丨习远平等瞻仰了小河会议革命旧址（1070）
9. 【上级要情】纪念小河会议召开75周年座谈会在榆林举行（60）
10. 纪念小河会议召开75周年座谈会在榆林举行​ 刘国中出席并讲话 赵一德主持 习远平王均伟讲话（6）
11. 每日一学 | 纪念小河会议召开75周年座谈会在榆林举行（96）
12. 纪念小河会议召开75周年座谈会在榆林举行（1.1万）
13. 人文榆林丨重访小河会议旧址：“李得胜”在小河村的46天（141）
14. 【视频】走进小河会议旧址（404）
15. 走起！坐着动车游靖边（636）
16. 【学党史 悟思想 办实事 开新局】景德镇公安党建微课堂——​​老方说党史|小河会议旧址（总第1017期）（15）
17. 景德镇公安党建微课堂——​​老方说党史|小河会议旧址（总第1017期）（463）
18. 马岔镇赴小河会议革命旧址开展“传承红色基因 赓续红色血脉”主题党日活动（437）
19. 建团一百周年 | 《致敬峥嵘岁月，献礼建团百年》团史学习专栏（27）（15）
20. 打卡“红色印记”，走进靖边县小河会议旧址（670）
21. 【党史故事】陕西省榆林市小河村小河会议：革命战争胜利前夜（11）
22. 陕西省榆林市小河村小河会议：革命战争胜利前夜（181）
23. 红旗漫卷——陕西革命旧址云上展之九十四：小河会议旧址（69）
24. 红船破浪：小河革命旧址（133）
25. 《党史故事里的陕西》第二十期：转折关头的一次重要会议——探访靖边县小河镇小河会议旧址（14）
26. 第四届“寻梦统万 写意靖边”全国名家油画写生活动在靖边举行（445）
27. 七彩榆林，给你点“颜色”看看！（38）
28. 李世书到小河会议旧址重温峥嵘岁月 砥砺初心使命（5102）
29. 三下乡 | 靖边县小河会议旧址宣讲（425）
30. 总要去趟靖边吧！（4.7万）
31. 【建党百年】榆林红色景点：小河会议旧址（183）
32. 巾帼讲解员讲党史 | 小河会议旧址的红色故事（623）
33. 【寻访红色足迹】“传承红色基因 凝聚奋进力量”——定边县第二小学赴靖边县参观红色教育基地小河会议旧址（1008）
34. 我们一起学党史 | 小河会议旧址的红色记忆（50）
35. 榆林市补遗之靖边县小河会议旧址（74）
36. 战区陆军某直属旅：打通党史学习教育向实践转化的最后一公里（3782）
37. 靖边县各乡镇、部门积极开展主题党日活动（570）
38. 重访小河会议旧址：“李得胜”在小河村的46天（3）
39. 【自信世纪 红色讲堂】讲述小河会议的故事——世纪小学“红色讲堂——党史故事我来讲”系列活动（十三）（349）
40. 重走革命路·启航新征程 靖边县举办纪念中共中央转战陕北活动（167）
41. 百年荣光 | 追寻家乡红色记忆——陕西（193）
42. 学百年党史 践初心使命——绥德县林业局党支部走进靖边“小河会议”旧址开展主题党日活动（426）
43. 红色印记，走进榆林——小河会议旧址（97）
44. 学党史 守初心||转折关头的一次重要会议——探访靖边县小河镇小河会议旧址（73）
45. 没有共产党就没有新中国！罗援少将报告会在小河会议旧址开讲（595）
46. 财政局到靖边小河会议旧址开展“传承红色基因 牢记初心使命”主题教育党史学习（46）
47. 三秦大地上的红色印记 |小河会议召开地靖边小河村（177）
48. 转折关头的一次重要会议 ——探访靖边县小河镇小河会议旧址（349）
49. 红色印记•榆林 | 走进小河会议旧址（10）
50. 水投集团党支部走进靖边“小河会议”旧址 开展“重温红色记忆 传承革命精神”主题党日活动（114）
51. “印记”微党史摄制组走进 靖边小河会议旧址||花絮（32）
52. 靖边县人民政府关于小河会议旧址景区封闭施工的通告（89）
53. 绥德县政协机关党支部走进靖边“小河会议”旧址开展主题党日活动（50）
54. 靖边法院赴小河会议革命旧址重温党的光辉历史（756）
55. 微拍靖边丨小河会议旧址（97）
56. 靖边事儿丨小河会议旧址被列为全国重点文物保护单位（168）
57. 分享一篇文章。（118）
58. 子洲县公共就业和人才服务中心赴靖边小河革命会议旧址开展主题教育活动（488）
59. 民建榆林市委会赴靖边小河会议旧址深入开展主题教育活动（266）
60. 【工作动态】榆阳区司法局赴靖边县小河会议旧址开展“不忘初心、牢记使命”主题教育实践活动（80）
61. 榆阳区残联开展“不忘初心、牢记使命”党性教育 ——赴靖边县参观红色教育基地小河会议旧址（129）
62. 常氏庄园、小河会议旧址跻身“国保”（31）
63. 【重温革命历史 牢记使命担当】院党委组织党员干部赴靖边小河会议旧址开展主题党日活动（282）
64. 【不忘初心 牢记使命】安塞区检察院党员干警到小河会议旧址接受革命传统教育（57）
65. 靖边县委巡察办赴小河会议旧址开展 “不忘初心、牢记使命”主题教育活动（644）
66. 靖边团县委赴小河会议旧址开展“追寻红色记忆，践行初心使命”主题教育活动（195）
67. 毛主席在靖边小河村的故事，你听过吗？（856）
68. 【文化】榆林红色革命教育基地巡礼第8站丨小河会议旧址（）
69. 【巡礼】榆林红色革命教育基地：小河会议旧址（216）
70. 【不忘初心 牢记使命】榆林红色革命教育基地巡礼第7站丨青阳岔革命旧址（34）
71. 【非常关注】榆林红色革命教育基地巡礼第8站丨小河会议旧址（86）
72. 弘扬革命精神 传承红色基因---绥德县纪委监委全体干部赴靖边小河会议旧址参观学习（157）
73. 【基层党建】 我校机关党委第十党支部赴靖边县小河会议革命旧址开展“踏寻伟人足迹，追忆红色历史，传承革命精神”主题教育活动（225）
74. 【寻树记】匈奴故都寻古树，锦绣靖边览胜景（27）
75. 重访红色基地 缅怀革命先烈 ——靖边职教中心全体党员赴小河会议旧址参观（310）
76. 内有福利| 和土然一起，来一场说走就走的旅行（9）
77. 2018年春季中青年干部培训班深入靖边小河革命旧址开展现场教学实践（511）
78. 绥德县92名科级干部赴靖边“小河会议”革命旧址接受党性教育（398）
79. 小河会议旧址（7）
80. 榆林7个景区被评为陕西“平安景区”，靖边县小河会议旧址景区榜上有名！（88）
81. 【重大利好】我市靖边县小河会议旧址、绥德县革命历史纪念馆入选《全国红色旅游经典景区三期总体建设方案》（26）
82. 揭牌了！又一市级现场教学基地落户靖边小河会议旧址（3486）
83. 【品德教育】靖边一小“爱国主义教育基地”小河会议旧址挂牌仪式（499）
84. 图文 | 靖边一小“爱国主义教育基地”小河会议旧址挂牌仪式（403）
85. 迎国庆，靖边为何将党建例会开在小河？（711）
86. 纪念中共中央转战陕北胜利70周年：靖边小河会议旧址（41）
87. 好消息！榆林又添一处AAA景区！（2102）
88. 榆林又添一处3A级景区！（1877）
89. 靖边二中党支部组织全体党员到我县爱国主义教育基地——小河会议旧址参观学习（918）
90. 【行游榆林】靖边小河会议旧址（434）
91. 中共中央在小河纪念馆（73）
92. “美丽陕西·省界行”走进靖边小河革命旧址（128）
93. 集团公司全体党员赴靖边县小河会议旧址 进行参观学习教育（63）
94. 最受青睐的榆林红色旅游景点（3）
95. 旅游|靖边小河会议旧址图集（104）
96. 红色革命旧址——小河会议旧址（126）
97. 这些榆林的红色旅游景区，你都去过吗？（340）
98. 【靖边新闻】靖边县小河会议旧址被命名为市级廉政教育基地（2410）
99. 毛主席之女李讷一行到访靖边小河会议旧址（2106）

富平县红色旅游系列景区(富平县青少年教育基地（6）、八路军 120师抗日誓师纪念地（1）、渭北革命根据地交通联络站故址（4）、康庄战斗烈士陵园（2）)

富平县青少年教育基地

1. 创新普法，这里成了孩子们学法的打卡点！（36）
2. 渭南日报：打造乡村教育振兴的“最美样板”——富平县大力推进美丽乡村学校建设走笔（1730）
3. 我为群众办实事| 富平县“138”法推动乡村教育振兴再提升（714）
4. 【为群众办实事示范法院创建】创建平安校园 护航阳光成长——富平法院民法典宣讲走进富平县特殊教育学校（237）
5. 司法为民践初心 扛起检察新担当——富平县人民检察院亮点工作综述（21）
6. 聚焦二十大·奋进新征程| 富平教育十年发展综述⑤（965）
7. 党的二十大报告在富平县教育系统中引起热烈反响①（1579）
8. 【优秀办案团队】“红韵富检”“柿柿红” 守护明天事事红——富平县检察院“柿柿红”未检团队（658）
9. 富平县教育系统2022年度教育新闻征集展示活动开始啦！（869）
10. 点赞！富平县教育局荣获2021年度目标责任考核优秀单位（1万）
11. 【工作动态】富平县青少年校外活动中心成功开展社会实践活动（1177）
12. 【活动中心·动态】富平县青少年校外活动中心暑期班开课了!（643）
13. 富平县青少年校外活动中心开展劳动教育实践活动（416）
14. 【检察工作动态】检校携手，呵护少年的你——渭南市高新小学师生走进富平县青少年法治教育基地（193）
15. 【喜报】赞！富平县人民检察院“柿柿红”未检办案团队被表扬为2021年度全省优秀办案团队（491）
16. 渭南市检察院党组书记、检察长殷军到富平县人民检察院调研（图）（266）
17. 富平县“七坚持”推动义务教育优质均衡创建工作（748）
18. 陕西省青少年教育基地—富平县立诚中学（2518）
19. 富平县青少年校外活动中心召开“作风建设提升年”专题组织生活会及座谈会（439）
20. 【活动中心·动态】富平县青少年校外活动中心春季班开课了!（575）
21. 【法治陕西声音】红色铸魂为新时代检察工作持续赋能 陕西富平：打造“红韵富检”文化品牌（191）
22. “红韵富检”：红色铸魂为新时代检察工作持续赋能（3113）
23. 【检察工作动态】检爱同行 共护未来——刘集镇中心小学师生走进富平县青少年法治教育基地（180）
24. 【文化品牌】富平县检察院“红韵富检”被评为2021年全国检察机关十佳文化品牌（142）
25. 学用法律 构筑青春防线——实验中学学生赴富平县青少年法治教育基地参观学习（1051）
26. 【检察工作动态】检爱同行 共护未来——铁门小学师生走进富平县青少年法治教育基地（211）
27. 【检察工作动态】检爱同行 共护未来——东上官初级中学师生走进富平县青少年法治教育基地（309）
28. 【检察工作动态】未检护航 法佑青春——刘集镇初级中学师生走进富平县青少年法治教育基地（300）
29. 【检察工作动态】检校携手 共佑未来——和平小学师生走进富平县青少年法治教育基地（314）
30. 【检察工作动态】知法懂法 法在心中——东区小学师生走进富平县青少年法治教育基地（319）
31. 知法懂法 法在心中—东区小学师生走进富平县检察院青少年法治教育基地（1331）
32. 【活动中心•动态】富平县青少年校外活动中心2021年秋季班开课啦！（531）
33. 富平县检察院“柿柿红”未检团队入选《全省未成年人检察示范品牌》（465）
34. 【队伍教育整顿】省政法队伍教育整顿第五指导组到富平县人民检察院检查指导工作（380）
35. 特别的爱给特别的你——富平县特殊教育学校师生走进“柿柿红”青少年法治教育基地（337）
36. 法治之光照亮成长之路 ——富平县特殊教育学校走进富平县青少年法治 教育基地开展现场教学（281）
37. 【活动中心•培训】​富平县青少年校外活动中心2021年暑期班开始报名啦!（1651）
38. 吉米需要独角兽！——富平县检察院为富平县实验幼儿园花式普法（308）
39. 百年风华，点亮三秦“红色坐标”（662）
40. 镇渭一家亲——苏陕两地青年走进富平县爱国主义教育基地（50）
41. 【“三有”争创】为“柿柿红”插上翅膀，助青少年放飞梦想！（133）
42. 为“柿柿红”插上翅膀，助青少年放飞梦想！（363）
43. 走进法治教育基地，知法学法新体验！（348）
44. 富平检察“柿柿红”受陕西电视台关注！（1255）
45. 安全红丝带，青春须设防！教育局组织机关干部参观青少年法治教育基地（1919）
46. 【活动中心•科技】富平县青少年校外活动中心成功举办全县科技教师培训活动（512）
47. 【队伍教育整顿】检察英模 践行担当（857）
48. 万物萌动，身启美好！——富平县检察院青少年法治教育基地等你来（315）
49. 年度盘点·校长谈|孙敏：克坚攻难 众志成城 谱写新时代富平校外教育新篇章（543）
50. 莲湖初级中学师生代表赴富平县青少年法制教育基地开展现场法治教育活动（1927）
51. 渭南初级中学师生赴富平县青少年法治教育基地开展现场法治教育活动（2107）
52. “检察蓝”与“校园绿”的法治之约——渭南初级中学师生走进富平县青少年法治教育基地（407）
53. 水韵南小 ｜ 成长中的呵护 ——淡村镇南社小学走进富平县青少年法制教育基地研学教育活动纪实（232）
54. 携手关爱 共护明天 —富平县东华小学走进县检察院参观青少年法治教育基地（1184）
55. 【活动中心•培训】富平县青少年校外活动中心举办科技专项培训活动（313）
56. 富平县人民检察院业绩考评工作运行良好（232）
57. 未检护航 法佑青春 ——富平县实验中学师生走进青少年法治教育基地（6548）
58. 检爱护航 法护青春——实验中学学生走进富平县人民检察院青少年法治教育基地（4235）
59. 活动中心•党建】县委组织部检查富平县青少年校外活动中心党建工作。（406）
60. 【开学第一课】撑起“法治蓝天”，只为守护“少年的你”——实验小学师生走进富平县人民检察院（2229）
61. 开学第一课 用实际行动守护“少年的你”——实验小学师生走进富平县人民检察院（1911）
62. 12处！渭南新增的这些爱国主义教育基地你去过吗？（107）
63. 全市新增12处市级青少年爱国主义教育基地（490）
64. 守望正义之责，责亦润物耕心！ ——临渭区检察院走进富平县检察院就未检工作传经送宝交流经验（302）
65. 富平信息第17期（902）
66. 【活动中心•培训】富平县青少年校外活动中心暑期培训班开课啦！（371）
67. ​富平县青少年校外活动中心到白庙大王村进行帮扶脱贫（20）
68. 富平县青少年校外活动中心在富平县教育局2019年度目标责任考核中被评为一档，同时被授予2019年目标责任考核“优秀单位”称号。（309）
69. 富平县青少年校外活动中心积极投身战“疫”大军，肩负责任，勇于担当。（44）
70. 富宁县青少年法治教育基地初步建成并投入使用（39）
71. 富平县青少年校外活动中心建成数字化书法教室（275）
72. 富平青少年校外活动中心2018年暑期招生开始啦！（1099）
73. 富平县4个红色景区纳入全国建设方案！（6114）
74. 富平县青少年校外活动中心参加2017年全省青少年“三国文化 传承经典”夏令营（845）
75. 富平县青少年校外活动中心2017年科技教师培训圆满结束（120）
76. 第二站 | 西安eTV“2017研学旅行”走进富平陶艺村、爱国主义教育基地（1274）
77. 富平县青少年校外活动中心2017年春季培训班2月17日正式开课啦！（1811）
78. 韩城市校外同仁来富平县青少年校外活动中心参观交流（81）
79. 推荐丨富平县红色旅游系列景区成功入选全国红色旅游经典景区名录（56）
80. 【渭南资讯】富平县红色旅游系列景区 成功入选全国红色旅游经典景区名录（113）
81. 时速抢修 情浓子夜 ——富平全国青少年教育基地电力抢修侧记（184）
82. 富平县青少年校外中心被陕西省教育厅评为德育工作先进集体（237）
83. 习仲勋陵园成为爱国主义教育基地（581）
84. 我省两基地被命名为全国爱国主义教育示范基地（577）
85. 富平县青少年校外活动中心介绍（149）

八路军 120师抗日誓师纪念地

1. 【学党史 守初心】红色金融史（五十九）|成立兴县农民银行，保障八路军120师军需供应（5）
2. 一封闪烁着人性的信函（111）
3. 【党史天天学】八路军东渡黄河后的第一战，打破了日军“不可战胜”的神话！（6）
4. 八路军抗战老照片（250）
5. 献礼二十大系列片——八路军120师抗日誓师纪念地（85）
6. 镇长杨毛带队检查“国庆”节前安保维稳工作（1733）
7. 八路军一二〇师抗日誓师纪念地获评“省级党史教育基地”（597）
8. 好男儿志在军营 我县举行2022年下半年新兵出征誓师大会（785）
9. 【红色厚土】富平：八路军120师抗日誓师（94）
10. 八十五年后的重逢（478）
11. 八路军120师抗日誓师纪念地（11）
12. 石川河畔 玉兰花语 | 快乐地挥手吧，不为作别，是对未来的迎接（1214）
13. 红色初心 | 八路军120师抗日誓师纪念地（236）
14. 暑期实践 | “重走红色足迹”——探访八路军120师抗日誓师纪念地（62）
15. 庄里试验区开展建党101周年“五个一”系列活动（810）
16. 《红色印记•薪火相传》八路军120师抗日誓师纪念地（156）
17. 赓续红色血脉 当好红色传人 | 庄里试验区开展“迎七一”党性教育活动（1099）
18. 今天，重走这段路！（1707）
19. 【建团百年】庄里试验区举行庆建团百年主题团日活动暨新团员入团仪式（1090）
20. 共叙百年奋斗历程 赓续红色基因血脉 第37期｜八路军一二零师抗日誓师纪念碑（32）
21. 21式军装领取渠道公布！（10万+）
22. 铸魂育人 | 参观见学受洗礼 砥砺奋进正当时（2121）
23. 不忘来时路 | ㉙ 福尔曼为红军拍摄的珍贵照片（523）
24. 丝绸之路研究院党支部党史教育系列（18）| 八路军120师抗日誓师大会（47）
25. 红色初心之旅——八路军120师抗日誓师纪念地（229）
26. 【讲党史故事 迎建党百年】第六期——八路军120师抗日誓师（206）
27. 追寻红色记忆——关中八路军120抗日誓师纪念地（75）
28. 市委统战部来八路军120师抗日誓师纪念地参观学习（144）
29. 学党史 红色游 | 带您走进八路军120师抗日誓师纪念地（341）
30. 【富中·党建】踏着先烈的足迹前进（3234）
31. 八千壮士出秦东——八路军120师抗日誓师的故事（164）
32. 八路军120师抗日誓师纪念地招聘讲解员2名（1961）
33. 杨正前:今昔庄里镇（5136）
34. 【不忘初心 牢记使命】八路军120师抗日誓师纪念地：传承红色基因 汇聚奋进力量（828）
35. 浴血奋进照丹青 红色基因代代传——访八路军120师抗日誓师纪念地（966）
36. 【主题教育】渭运社区党总支前往八路军120师抗日誓师纪念地开展“不忘初心、牢记使命”主题教育专题学习（160）
37. 庄里社区党员到八路军120师抗日誓师纪念地开展“不忘初心、牢记使命”主题教育专题学习（368）
38. 【庆祝新中国成立70周年】我市隆重纪念八路军东渡黄河出师抗日82周年（附视频）（1833）
39. 王永丽带队到八路军120师抗日誓师纪念地开展“不忘初心、牢记使命”主题教育专题学习（1696）
40. 好男儿，当兵去！——富平县2019年新兵出征誓师大会在八路军120师抗日誓师纪念地成功举行（3279）
41. 120师从这里奔赴抗日前线（288）
42. 岁月无声，初心永存！ 八路军120师抗日誓师纪念碑下有传承（216）
43. 富平120师抗日誓师大会纪念地（32）
44. 县委巡察办党支部赴八路军120师抗日誓师纪念地开展主题党日活动（778）
45. 抗战精神 从这里传承——寻访八路军120 师抗日誓师纪念碑（108）
46. 八路军120师鏖兵三晋：纵横战场 不辱使命（1673）
47. 八路军一二零师抗日老战士遗孀参观纪念地（943）
48. 红色旅游新亮点——八路军120师抗日誓师纪念地迎来游客小高峰（73）
49. 国庆期间，八路军一二〇师抗日誓师纪念地首次迎来游客小高峰（854）
50. 八路军120师抗日誓师纪念地保护工作今与昔（729）
51. 八路军120师后代重访父辈抗日誓师出征地（4257）
52. 习远平在八路军120师抗日誓师纪念碑落成仪式上的讲话全文（2044）
53. 八路军120师抗日誓师纪念碑落成仪式在富平县举行（529）
54. [党政荐读] 八路军120师抗日誓师纪念碑落成仪式在渭南举行（3）
55. 八路军120师抗日誓师纪念地建成（1862）
56. 八路军120师抗日誓师纪念碑落成仪式在富平举行（143）
57. 八路军120师抗日誓师纪念碑落成仪式在渭南举行（1.2万）
58. 八路军120师抗日誓师纪念碑落成仪式在渭南举行, 胡和平习远平贺晓明讲话（1.3万）
59. 庄里试验区2018年重点建设项目动态（十七）八路军一二〇师抗日誓师纪念地（1479）
60. 县政协到庄里试验区八路军120师抗日誓师纪念地视察旅游工作（750）
61. 2018年重点建设项目动态（三）八路军一二〇师抗日誓师纪念地（757）
62. 【每日一文物】神池县近现代重要史迹及代表性建筑：八路军120师指挥部旧址（48）
63. 红色庄里：八路军120师80年前从庄里镇誓师出征抗日（3.4万）
64. 【历史事件】八路军120师致电大青山抗日根据地（24）
65. 从胜利走向胜利——献给中国人民解放军建军90周年（10万+）

康庄战斗烈士陵园

1. 沿着黄河游名城｜黄河华山 人文渭南（142）
2. 我县举行烈士纪念日公祭活动（286）
3. 【大学习铸忠诚】薛镇组织开展“致敬抗战英雄，传承红色基因”主题纪念活动（775）
4. 【暑期社会实践系列】“星火”暑期社会实践团探访抗战遗迹，学习抗战精神（二）（62）
5. 石川河畔 玉兰花语 | 快乐地挥手吧，不为作别，是对未来的迎接（1214）
6. 陕西革命旧址（十六）：富平县烈士陵园（86）
7. 【富平红色地名故事】康庄烈士陵园（207）
8. 渭南红色旅游 | 踏寻民族记忆 追仰吾辈英烈（313）
9. 清明，一名老兵的追思（6340）
10. 缅怀革命先烈 汲取奋进力量--县委巡察办党支部赴薛镇康庄烈士陵园开展祭扫活动（852）
11. 【底小·动态】“传承红色基因，争做时代新人”——薛镇底店小学清明节主题教育系列活动（565）
12. 【富中·党建】清明前夕：富中师生代表赴富平烈士陵园祭奠革命英烈（3464）
13. 看看咱富平的这位才女用画笔勾勒的“红色印记”（1678）
14. 桃李枝头春意浓 扬帆逐梦正当时——富平县实施“四大工程”办人民满意的教育（951）
15. 【法治陕西声音】红色铸魂为新时代检察工作持续赋能 陕西富平：打造“红韵富检”文化品牌（191）
16. 【媒体看检察】红色铸魂为新时代检察工作持续赋能（155）
17. 【媒体看检察】检察日报：红色铸魂为新时代检察工作持续赋能（273）
18. “红韵富检”：红色铸魂为新时代检察工作持续赋能（146）
19. 大美富平 | 玉虎迎春 女教师秦娟描绘红色印迹（955）
20. 【高举旗帜 响应号召 奋进新时代 启航新征程】薛镇举行康庄战斗胜利73周年纪念活动（1136）
21. 薛镇举行纪念康庄战斗胜利七十三周年大会（1977）
22. 烈士英灵贯长虹——走进富平县烈士陵园（235）
23. 缅怀革命先烈 凝聚奋进力量（81）
24. 富平县举行烈士纪念日公祭活动（5305）
25. 文艺助力乡村振兴系列报道之八（642）
26. 王 璐：康庄战斗烈士陵园赋（934）
27. 【文旅融合 魅力富平】红色初心之旅（三） —— 康庄战斗烈士陵园（162）
28. 【秦东风云录】富平康庄战斗（26）
29. 渭南市不可移动革命文物—富平县（502）
30. 【党史动态】《渭南日报》刊载富平县不可移动革命文物（124）
31. 寻访中感悟 红色富平铸辉煌（635）
32. 三下乡 党史学习 | 三秦学史力行 汲取奋进力量（1117）
33. 学党史 红色游丨带您走进临渭区红色地下交通站旧址（147）
34. 2021三下乡|人工智能学院“星火”党史学习实践团—— 第六站：富平康庄战斗革命烈士陵园（20）
35. 人工智能学院“星火”党史学习实践团—— 第六站：富平康庄战斗革命烈士陵园（25）
36. 暑期实践 | 探寻百年红色足迹·见证伟大攻坚成果——长安大学赴陕西省渭南市富平县暑期社会实践活动总结（101）
37. 【红色文化篇】：挖掘红色资源 汲取奋斗力量（430）
38. 长安大学地测学院赴延安“重走红军在陕路，奋斗百年新征程”暑期社会实践队富平分队社会实践纪实（四）（149）
39. 探寻百年红色足迹·见证伟大攻坚成果——长安大学赴陕西省渭南市富平县暑期社会实践活动总结（175）
40. 庆建党百年 | 渭南推出6条红色精品旅游线路（六）（1597）
41. 薛镇机关党总支：开展“缅怀革命先烈 传承红色精神”主题党日活动（659）
42. 庆建党百年 | 渭南推出6条红色精品旅游线路（一）（2128）
43. 学党史缅怀先烈 守初心砥砺前行 富平执法局赴县英烈纪念馆和米家窑开展党史学习教育（1102）
44. 庆建党百年 | 渭南推出6条红色精品旅游线路（二（225）
45. 【富平红色故事】红色记忆：康庄战斗（86）
46. 党史|富平康庄战斗（24）
47. 红色初心之旅——富平县烈士陵园（382）
48. 【奋斗百年路 启航新征程】富平红色故事（二十八）富平沟龙土城墙下忆康庄战斗（420）
49. 学党史 红色游｜带您走进渭华起义纪念馆（262）
50. 【富平红色故事】康庄战斗著史册 烈士英灵贯长虹（440）
51. 富阎产业合作园区管委会见习生招募！（2187）
52. 2021年推动关中平原城市群和新型城镇化发展重点工作任务（303）
53. 胡广鑫带队拜访西飞民机公司（1408）
54. 学党史 红色游丨带您走进康庄战斗烈士陵园（229）
55. 富平县米家窑地下交通站旧址迎来红色研学热（532）
56. 渭南推出六条红色精品旅游线路，“五一”等你来打卡！（841）
57. 【奋斗百年路 启航新征程】富平红色故事（五）康庄烈士陵园（151）
58. 富平红色故事|康庄烈士陵园（2278）
59. 渭南“红色+”精品旅游线路来了!（271）
60. 渭南市推出6条红色精品旅游线路（1664）
61. 甘棠镇瞻仰棋盘山战役烈士纪念碑（160）
62. 富平县康庄战斗烈士陵园红色旅游景区基础设施配套项目采购结果（37）
63. “缅怀革命先烈，弘扬爱国情怀” ——富平县帮帮乐公益协会祭扫烈士陵园活动（233）
64. 【学党史牢记使命，祭英烈传承精神】——富平县人武部、县退役军人事务局联合开展党史学习教育暨清明祭扫活动（643）
65. 【富平】康庄战斗烈士陵园红色旅游景区配套项目（30）
66. 县烈士陵园列入市级烈士纪念设施保护单位（463）
67. 富平县烈士陵园列入市级烈士纪念设施保护单位（598）
68. 【我们的十三五】文旅融合迈出新步伐（674）
69. 富平县素质教育成果在渭南市教育局长论坛展示交流（5066）
70. 从A到Z都是富阎新区的模样（1799）
71. “十一”黄金周 我县共接待游客38.5万人次（1982）
72. 我县在康庄烈士陵园举行烈士纪念日公祭活动（1356）
73. 富平县烈士公祭活动在县烈士陵园举行（1430）
74. “忆入党初心，葆党员本色，见落实行动” ——富平法院党员干警赴薛镇烈士陵园开展主题党日活动（437）
75. 【​山川履痕】陕西 王巧样‖拜谒康庄烈士陵园记（71）
76. 【最美富平】走进富平县烈士陵园（1112）
77. 【主题党日】渭运社区党建共建联合会开展主题党日活动（366）
78. “历史的丰碑” —— 中共富平县委退役军人事务领导小组办公室开展2020年清明节代祭扫活动（1029）
79. 【师说心语】讲好家乡故事 ，学活“战疫”教材，悟透“中国精神”（1352）
80. 兰州日报寻找安光孝烈士家人 烈士籍贯疑似安阳内黄县（1150）
81. 我县在康庄烈士陵园隆重举行烈士纪念日公祭活动（3622）
82. 梦想学校 | 队长来了·第二波（652）
83. 我县在康庄烈士陵园举行清明祭英烈活动（1380）
84. 致敬！一家三代73年守陵承诺终圆满，张家岗战斗抗日烈士迁葬溧水中山烈士陵园（189）
85. “缅怀革命先烈、弘扬民族精神”——富平县东屏幼儿园大班扫墓活动报道（2394）
86. 清明祭英烈：缅怀先烈 不忘初心（3492）
87. 富平县四处景点列入全国红色旅游经典景区建设名单，你知道都有哪些吗？（5142）
88. 发改委给228个红色纪念馆(旧址)发红包（6809）
89. 富平县举行烈士公祭活动（927）
90. 2017西安丝绸之路国际旅游博览会的陕西味|十二地市倾情推荐，N条线路嗨玩陕西（关中篇）（41）
91. 【富平薛镇派出所开展缅怀康庄战斗革命烈士活动】（117）
92. 【收藏】做为富平人，你应该知道的人文景点（4219）
93. 富平县红色旅游系列景区 ‖ 成功入选全国红色旅游经典景区名录（9405）
94. 国庆假日不想出远门？没关系，小编为你整理了渭南当地的多条旅游经典线路！（583）
95. 【县团要闻】 勿忘国耻•圆梦中华——暨康庄烈士陵园烈士纪念日公祭活动（551）
96. “追寻抗战足迹，缅怀革命先烈〞爱国主义教育活动在康庄烈士陵园举行！（359）
97. 小长假快来了“渭南周边旅游精品线路” !（556）

渭北革命根据地交通联络站故址

1. 米家窑的秘密（103）
2. 富平县渭北革命根据地交通联络站故址红色旅游景区道路工程（一期）项目中标结果公示（24）
3. 建党百年之际，带你畅游西北大地上的红色经典景区（134）
4. 陕西渭南红色旅游景点概要简介（449）
5. 米家窑地下交通站（84）
6. 红色米家窑这些不为人知的故事（1864）
7. 渭南师范学院经济与管理学院学子赴渭北革命根据地交通联络站故址——米家窑地下交通站（167）
8. 人间四月天丨最美富阎红色游（453）
9. 【富检文苑】传承红色精神，续写时代担当（260）
10. 富平历史 | 李先念夜宿米家窑（2523）
11. 实验中学“追寻红色印记 传承红色基因”研学实践活动（3036）
12. 万载湘鄂赣革命根据地旧址列入《全国红色旅游经典景区三期总体建设方案》（409）
13. 【重磅消息】喜迎二十大，奋进新征程（41）
14. 【富检文苑】传承红色基因 忠于法治使命（4526）
15. 李广远丨红色米家窑（1531）
16. 【文旅开发】120师抗日誓师纪念地、渭北革命根据地交通联络站、金粟山景区将故址开发和提升改造（227）
17. 【FM106.2旅游】革命老区红色旅游扶贫迎“利好” 这些景点基建将升级（27）
18. 富阎新区的“高级脸” 你说谁最上镜（429）
19. 【渭南资讯】富平县多举措推进红色旅游发展（84）
20. 【红色景区】国家发展改革委关于印发《全国红色旅游经典景区三期总体建设方案》的通知（544）
21. 又红又专！国务院发布的红色经典景区，陕西占了12个！（991）
22. 2022年省级旅游发展专项资金补助项目公示名单（70）
23. 国务院红色旅游景区三期建设项目方案，陕西省12个，富平竟占了4个！（1091）
24. 渭南师范学院经济与管理学院学子赴蒲城县将相故里——王鼎纪念馆（78）
25. 在这片黄土地上，播撒着中国革命的红色希望（863）
26. 好消息！庄里120师抗日誓师纪念地、渭北革命根据地交通联络站要开发啦！（571）
27. 全国红色旅游经典景区三期总体建设方案项目公布，宁夏五席固原占四席！（2754）
28. 电影《燃烧的麦子》主题歌隆重发布—— 《今生跟着你》 王泽洲词曲 石倚洁演唱（260）
29. 富平县与迪威视讯签约智慧文旅项目（581）
30. 昭通有一个！国家发改委印发《全国红色旅游经典景区三期总体建设方案》（3975）
31. 咸丰忠堡大捷遗址及烈士陵园成功纳入全国红色旅游经典景区三期总体建设方案中……（564）
32. 图解:全国红色旅游经典景区三期总体建设方案（335）
33. 《全国红色旅游经典景区三期总体建设方案》印发（454）
34. 《全国红色旅游经典景区三期总体建设方案》印发 | 附图解（1）
35. 会宁县红军长征会师旧址被纳入全国红色旅游经典景区三期总体建设方案项目（576）
36. 铜川市陕甘边革命根据地旧址列入全国红色旅游经典景区名录（560）
37. 机械工程学院暑期三下乡|追溯百年，重温党史（420）
38. 厉害了，我的大富平！厉害了，我的大高新！吃喝玩乐大盘点！（8379）
39. 国家发展改革委关于印发《全国红色旅游经典景区三期总体建设方案》的通知（54）
40. 红色基因 赓续传递——渭南红色文化建设综述（83）
41. 金融助推老区发展|走进富平（556）
42. 陕西13家景区入选全国红色旅游经典景区名录（909）
43. 又上榜了！凤县被全国红色旅游点名了（1276）
44. 2017年度富平“小城大事”，“最美瞬间”盘点（3814）
45. 【党史学习】渭南市组织开展“渭南卫健党旗红”活动纪实（58）
46. 十佳回访 || 红铸思想，专定方向——陈鹏（424）
47. 《全国红色旅游景点景区名录》公布 凤县两当起义纪念地榜上有名（670）
48. 【红色旅游】陕西省13个红色旅游景区列入全国红色旅游经典景区名录（351）
49. 我县四处景点列入全国红色旅游经典景区建设名单（844）
50. 【渭南资讯】富平县与深圳迪威举行合作项目签约仪式（225）
51. 在宝鸡拥有这张卡的小伙伴们有福了！可“刷脸”免费游这些景区！（10）
52. 【工作动态】富平县与深圳迪威举行合作项目签约仪式（48）
53. 富平系列景区入选全国红色旅游经典景区名录啦！（37）
54. 传承红色基因 陕西渭南新增两所红军小学（208）
55. 一切为了人民健康（71）
56. 《全国红色旅游经典景区名录》发布，泾阳安吴青训班入选（683）
57. 文化旅游｜国家发改委：全国红色旅游经典景区三期总体建设方案出台 228个建设项目（186）
58. 【标杆名企】迪威迅：深耕智慧化服务 挖掘物联网时代新价值（269）
59. 陕西省13个红色旅游景区列入全国红色旅游经典景区名录（22）
60. 沿着高速学党史—渭北革命根据地（264）
61. 【红色地标】渭北革命根据地烈士纪念碑（100）
62. 党史学习 | 渭北革命根据地（68）
63. 【党史博览】渭北革命根据地的历史定位：渭北革命根据地是陕甘边根据地发展巩固的重要依托（上）（31）
64. 【应用案例】陕西富平农村危房改造工程（1340）
65. 梁凤民会长受邀参加红军小学授旗授牌及爱国主义教育基地活动（159）
66. 渭北革命根据地（上）（395）
67. 【重磅消息】西安博信咨询2022年红色党建培训已开启（92）
68. 全国红色旅游经典景区三期总体建设方案出台，228个建设项目（1601）
69. 实践招募 | 清华大学学生社团“梦想学校”6.0启动招募（1496）
70. 【党史博览】渭北革命根据地的历史定位：渭北革命根据地是陕甘边根据地发展巩固的重要依托（下）（84）
71. 【重磅消息】西安博信咨询2022年红色党建培训火热报名中！（67）
72. 【教育】传承红色基因 富平这所学校新增为红军小学（1661）
73. 青春心向党，一起学党史（四十一）||渭北革命根据地的历史地位——西北地区第一块革命根据地（13）
74. 渭北革命根据地：西北地区创建的第一个革命根据地（83）
75. 党史上的今天·陕西（6月24日）（5）
76. 纪念西北第一块革命根据地——渭北革命根据地建成85周（8）
77. 纪念西北第一块革命根据地——渭北革命根据地建成85周年（组图）（1067）
78. 【渭北革命根据地的建立与发展】渭北地区早期的革命斗争（204）
79. 渭北革命根据地的历史定位（三）渭北革命根据地是陕甘边根据地发展巩固的重要依托（139）
80. 印记｜渭北革命根据地（198）
81. 发改委给228个红色纪念馆(旧址)发红包（370）
82. 渭北革命根据地的历史定位：渭北革命根据地是陕甘边根据地发展巩固的重要依托（下）（194）
83. 渭北革命根据地的历史定位：渭北革命根据地是中共在西北地区建立的第一块革命根据地（464）
84. 渭北革命根据地的历史定位：渭北革命根据地的创建是西北共产党人探索中国革命道路的有益尝试（162）
85. 纪念渭北革命根据地创建90周年暨渭北革命精神研讨会征文启事（184）
86. 汪锋与渭北革命根据地（31）
87. 渭北革命根据地（中）（214）
88. 渭北革命根据地（下）（207）
89. 渭北地区早期的革命斗争（83）
90. [党史读本]《 渭北革命根据地 》（66）
91. 西北根脉：诞生于农运烽火中的渭北革命根据地（29）
92. 【党史博览】渭北革命根据地的恢复（25）
93. 红色血脉 | 走近陕西的革命纪念馆（781）
94. 渭北革命根据地的历史定位（一）渭北革命根据地是中共在西北地区建立的第一块革命根据地（168）
95. 渭北革命根据地的历史定位：渭北革命根据地是陕甘边根据地发展巩固的重要依托（上）（221）
96. 渭北革命根据地的历史定位：渭北革命根据地的红色割据有力打击了国民党当局的统治（163）
97. 纪念渭北革命根据地建成85周年 暨学术研讨会征文启事（15）
98. 渭北革命根据地的战略北移（三）渭北革命根据地成为陕甘宁边区关中分区的重要组成部分（115）
99. 渭北革命根据地的战略北移（二）渭北革命根据地的战略北移与陕甘边南部苏区的创建（89）
100. 【陕西党史】渭北革命根据地烈士纪念碑举行奠基仪式（927）
101. 【党史博览】渭北革命根据地对中国革命的深刻启示（47）
102. 渭北革命根据地成为陕甘宁边区关中分区的重要组成部分（546）
103. 渭北革命根据地的恢复与发展（80）
104. 习水红军四渡赤水纪念地列入全国红色旅游经典景区三期总体建设方案（110）
105. 渭北革命根据地对中国革命的深刻启示（302）
106. 渭北革命根据地的恢复（124）
107. 《渭南旅游访谈》富平县3月15日录制结束（1481）
108. 【陕西日报】渭北高原起风云——探访渭北革命根据地革命前辈的英雄故事（50）
109. 泾三淳区域分中心党支部在渭北革命根据地烈士纪念碑开展缅怀先烈主题党日活动（132）
110. 陕西党史上的今天（35）
111. 2022年全国红色经典景区名录（27）
112. 【党史博览】渭北革命根据地的土地革命（37）
113. 渭北革命根据地的土地革命（160）
114. 我县与深圳迪威视讯公司签订合作协议（671）
115. 渭北革命根据地的历史定位（二）渭北革命根据地的红色割据有力打击了国民党当局的统治（183）
116. 【特别关注】《全国红色旅游经典景区名录》公布300处景区入选 有你家乡吗（498）
117. 100个红色基地打卡 | 党史旧址之陕西篇（218）
118. 100个红色基地打卡 | 伟人故居之陕西篇（299）
119. 【党史百年】苏皖边区政府旧址：传递不熄的红色火炬（51）
120. 好消息！八路军北村旧址入选国务院 第三批国家级抗战纪念设施、遗址名录！（978）
121. 渭北革命根据地的失守及其经验教训（258）
122. 渭北根据地的几件事（164）
123. 鲜为人知的渭北革命根据地，比陕北根据地还早，今只有块纪念碑！（572）

**陕南红军革命根据地系列景区(汉中市洋县华阳红二十五军司令部旧址（22）,西乡县红二十九军军部旧址及红四方面军总后医院旧址（6）;安康市汉滨区牛蹄岭战役遗址（59）;商洛市商南县前坡岭战斗遗址（1）)**

**商洛市商南县前坡岭战斗遗址**

1. “顺手牵羊”的六条公益诉讼线索（137）
2. 社会实践|“助力乡村振兴，建设美丽中国”专项计划——缅怀前坡岭革命先烈（91）
3. 红色旅游 | 中国工农红军第七十四师成立遗址暨师部旧址&前坡岭战斗遗址（203）
4. @商洛人，这些经典红色旅游地，带你重温峥嵘岁月！（166）
5. 【庆“七一”】 “重温革命记忆 传承红色基因”线上有奖拼图活动开始啦！（211）
6. 商洛之窗——赵川前坡岭！（87）
7. 赵川网络祭扫平台开通！快来为赵川前坡岭战斗遗址革命烈士祭奠（188）
8. 商洛记忆||不忘革命历史，追忆峥嵘岁月（153）
9. 【聚焦】商南县2021年度文化和旅游工作十大亮点（729）
10. 百年党史 · 红色商洛|商洛革命史上的重要战斗系列之前坡岭战斗（72）
11. 【红色记忆】:《红色商南》血战前坡岭（203）
12. 商洛革命文物图片展线上展览（四十四）︱前坡岭战斗遗址（43）
13. 电影《长津湖》爆火，商洛这几处红色旅游地你去过吗？（253）
14. 【乐游】西安⇋商南“绿巨人”发车！不到3小时就到商南，可以耍好几天（318）
15. 聆听商洛党史|前坡岭战斗（129）
16. 商南赵川：战火硝烟前坡岭 见证沧桑花门楼（44）
17. 全域旅游 | 红色印记 —— 商南赵川镇（142）
18. 红色记忆：《商山长歌》​血战前坡岭（166）
19. 红色旅游 | 建党100周年：追寻商洛的红色印记（414）
20. 庆祝建党100周年｜各级党组织在行动（602）
21. 美文 || 【红色印记】商南赵川镇（48）
22. 【红色印记】商南赵川镇（1905）
23. 【关注】“七一”建党节：追寻商南的红色印记（190）
24. 【智系列】—“七一”建党节：追寻商南的红色印记（87）
25. 【建党100年】血战前坡岭（162）
26. 寻访革命旧址 传承革命精神（99）
27. 赞！飞渡峡入选中华秦岭100景！实力顶流担当，养了眼、醉了心！（265）
28. 商南县公安局交警大队开展“红动商洛·血脉传承”主题党日活动（101）
29. 新鲜出炉！石泉3景入选中华秦岭100景！实力顶流担当，养了眼、醉了心！（174）
30. 安康9景入选中华秦岭100景！实力顶流担当，养了眼、醉了心！（564）
31. 【爱上安康】新鲜出炉！安康9景入选中华秦岭100景！实力顶流担当，养了眼、醉了心！（2）
32. 厉害了丨顶流担当！石泉3景入选中华秦岭100景！（6556）
33. 新鲜出炉！安康9景入选中华秦岭100景！实力顶流担当，养了眼、醉了心！（1284）
34. 访遗迹感悟红色精神 忆初心汲取奋进力量（46）
35. 中华秦岭100景”榜单出炉（278）
36. 【最美商洛检察人④】王甲：心系百姓 情满乡亲（576）
37. 两当云屏三峡入围“中华秦岭100景”推荐（93）
38. 【中华秦岭美景】新鲜出炉！商洛7景入选中华秦岭100景！（2）
39. “中华秦岭100景”榜单出炉，勉县这个景区榜上有名~（49）
40. 光雾山景区、米仓山景区、诺水河景区入选“中华秦岭100景”（109）
41. 我市金丝大峡谷等6景区入选中华秦岭100景（209）
42. 自然风光 I 历史遗存 I 文化胜景 I 民俗风情 I 入选“中华秦岭100景”榜单，你想要的陕西五龙洞国家森林公园全都有！（73）
43. “中华秦岭100景”榜单出炉，佛坪这个景区入榜！（313）
44. 【深呼吸小城】“中华秦岭100景”榜单出炉，云屏三峡国家AAAA级旅游景区入选!（290）
45. 【喜讯】商南2景入选中华秦岭100景（159）
46. 关注 | “建党百年，初心之路”红色地图主题纪念卡（250）
47. 商洛7景入选中华秦岭100景！（1396）
48. 喜讯 | 函谷关历史文化旅游区入选“中华秦岭100景”（523）
49. 巫山两大景区分别入选“中华秦岭100景”“大秦岭边际线上的最美风景”（1141）
50. 喜讯 | 巫山两大景区分别入选“中华秦岭100景”“大秦岭边际线上的最美风景”（800）
51. 新鲜出炉！商洛7景入选中华秦岭100景！（7793）
52. 给力！汉中多家景区入选“中华秦岭100景”榜单！（220）
53. “中华秦岭100景”榜单出炉，汉中这些景区入榜！（782）
54. “中华秦岭100景”榜单出炉，张良庙—紫柏山国家AAAA级旅游景区入选!（432）
55. 光雾山、米仓山景区入选“中华秦岭100景”（321）
56. 新鲜出炉！商洛7景入选中华秦岭100景！实力顶流担当，养了眼、醉了心！（757）
57. 资讯丨“中华秦岭100景”和“大秦岭边际线上的最美风景”推荐名单发布（115）
58. “中华秦岭100景”和“大秦岭边际线上的最美风景”推荐名单发布（519）
59. 打卡“红色记忆”，一起“陕”闪放光彩！娘家人又有好活动等你来！（26）
60. 追寻红色记忆——陕南商南县前坡岭战斗遗址（76）
61. 小马颂党恩（十一）——赵川前坡岭战斗遗址（131）
62. 商南竟然有这么多革命旧址！（1136）
63. 爱了！爱了！快来看看孩子们笔下的商洛市红色教育基地手绘图吧（895）
64. ​【活动】《商南红色文化》采编活动火热进行中（553）
65. 发扬斗争精神 凝聚信仰力量 — —商南县纪委监委开展“倡廉洁 践使命”主题党日活动（881）
66. 袁家沟、马炉...商洛这些红色旅游基地你去过吗？（203）
67. 转发收藏 | 商洛这些红色旅游基地你去过吗？（471）
68. 传承红色基因 锤炼提升“四力” 商洛日报社党员干部赴前坡岭开展主题党日活动（288）
69. 【喜讯】商南前坡岭战斗遗址被授予红色旅游基地​（1070）
70. 商南“双拥”工作让军民“一家亲”（225）
71. 【喜报】商南前坡岭战斗遗址荣获“红色旅游基地”称号，你去过吗（1372）
72. 商南赵川前坡岭战斗遗址荣获“红色旅游基地”称号（508）
73. 【喜讯】商南赵川前坡岭战斗遗址荣获“红色旅游基地”称号（515）
74. 突出“红”“绿”基调 发展滔河经济带 ——商南县“一心三带五协同”规划布局实施系列报道之四（301）
75. 【小编说新闻】商南命名一批爱国主义教育基地；商州纪委通报3起形式、官僚主义案例（248）
76. 商南县城关小学来赵川前坡岭战斗遗址啦！（536）
77. 昨日上午，商南县众领导一行来到赵川前坡岭​战斗遗址开展主题活动（433）
78. 国庆红·嗨西安 | 爱国传承9处“红色旅游地”，你都去过吗？（3202）
79. 年轻人都来这里旅游打卡了！陕西“红色旅游”受热捧（429）
80. 陕西人都来这里旅游打卡了！陕西“红色旅游”受热捧（2691）
81. 共青团商南县委开展 “庆祝建国70周年”系列活动（229）
82. 商洛市政协党组赴商南县红色革命教育基地开展专题学习（337）
83. 优秀投稿 | 边陲古堡赵川镇——前坡岭纪念碑（288）
84. 【清明】祭奠先烈英灵，传承红色基因！（458）
85. 分享一篇文章。（744）
86. 三下乡 | 西安建筑科技大学“红色印迹”实践团赴商南社会实践调研报告（141）
87. 【社会实践】西安建筑科技大学17级“红色印迹”实践团赴商南社会实践(上）（136）
88. 陕南红色旅游胜地、鄂入陕第一明星小城镇，商南县南部边陲赵川镇及 “赵川前坡岭遗址”（30）
89. 周末读史|商洛革命史上的重要战斗系列之前坡岭战斗（486）
90. 商南县赵川前坡岭战斗遗址（201）

**安康市汉滨区牛蹄岭战役遗址**

1. 三下乡｜“赓续红色血脉，助力乡村振兴”数字艺术学院“云中的红旗”实践团队赴安康开展社会调研（224）
2. 工学实践 | 红色回忆——牛蹄岭革命战役（411）
3. 摄影丨牛蹄岭战斗遗址公园（36）
4. 拜谒激战牛蹄岭的首战地——石头寨（255）
5. 红色汉滨故事汇：县河镇——安康牛蹄岭战役（934）
6. 医心向党 赓续红色血脉丨紫阳县中医医院组织党员干部到牛蹄岭战斗遗址开展主题党日活动（152）
7. 医心向党 赓续红色血脉丨紫阳县中医医院组织党员干部赴牛蹄岭战斗遗址开展主题党日活动（1720）
8. 追寻红色记忆，感悟初心使命 --重走战斗遗址牛蹄岭（24）
9. 朝阳社区开展“迎七一 祭英烈 传承革命精神”主题党日活动（216）
10. 牛蹄岭战役参战将士及烈士后裔祭奠革命先烈（260）
11. 【工作亮点】点赞！汉滨硒产业受到光明日报关注（25）
12. 点赞！汉滨硒产业受到光明日报关注（782）
13. 【光明日报】陕西安康：以‘硒’兴业 以‘硒’富民（310）
14. 红旗漫卷——陕西革命旧址云上展之二十六：牛蹄岭战役遗址（115）
15. 霸气！坦克“入住”牛蹄岭战役遗址公园（882）
16. 安康今天发生的事......5.31（5305）
17. 【国防教育】苍溪县 | 石家庄市鹿泉区 | 米易县 | 安康市汉滨区县河镇（89）
18. 霸气！坦克“入驻”牛蹄岭战役遗址公园（1699）
19. 汉斌记忆丨牛蹄岭战役（158）
20. 陕西革命旧址云上展（二十六）| 牛蹄岭战役遗址（28）
21. 【党史学习教育】安康红色地标：牛蹄岭战役旧址（23）
22. 安康市市场监管局举行“缅怀革命先烈 传承红色基因”清明祭扫主题党日活动（521）
23. 安康红色地标：牛蹄岭战役旧址（125）
24. 【校园文苑】牛蹄岭战役遗址公园参观记（535）
25. 安康汉调二黄研究院：组织党员干部赴牛蹄岭战斗遗址开展主题党日活动（170）
26. 陕西革命旧址(十):牛蹄岭战役遗址（42）
27. 致敬牛蹄岭（389）
28. “民情订单”变“满意清单”——汉滨区开展党史学习教育综述（80）
29. 【县域纵横·汉滨】“民情订单”变“满意清单”——汉滨区开展党史学习教育综述（25）
30. 假如2021年会发朋友圈！（572）
31. 踏上先烈足迹，缅怀逝去英雄——牛蹄岭战役（47）
32. 【党史学习教育】（177）激战牛蹄岭 碧血映山河（15）
33. 汉滨区大河中学党支部赴牛蹄岭战斗遗址开展主题党日活动（102）
34. 传承红色基因 赓续红色血脉——汉滨区大河中学党支部赴牛蹄岭战斗遗址开展主题党日活动（725）
35. 陕西省离退休干部教育基地在牛蹄岭战役遗址揭牌（346）
36. 安康“红色之旅”打卡地来啦！（511）
37. 电影《长津湖》爆火，安康这几处红色旅游地不可错过（837）
38. 汉滨“红色之旅”来啦！快来打卡吧！（101）
39. 安康“红色之旅”，石泉这里上榜！（470）
40. 去打卡丨安康“红色之旅”，石泉这里上榜！（99）
41. 安康“红色之旅”来啦！看看哪些地方值得打卡！（2917）
42. 校园新闻聚焦，一周新闻回顾（505）
43. 汉滨红色记忆172 牛蹄岭战役纪念设施（51）
44. 【烈士纪念日】今天，向先烈致敬！（383）
45. 【百年党史之汉滨记忆】牛蹄岭战役纪念设施（251）
46. 【打卡】在牛蹄岭战斗纪念碑前重温历史、致敬先烈（478）
47. 百集微视③ | 牛蹄岭战役的故事（15）
48. 全区认真学习贯彻习近平总书记“七一”重要讲话精神（四）（152）
49. 汉滨红色记忆106 安康牛蹄岭战役（55）
50. 【百年党史之汉滨记忆】安康牛蹄岭战役（444）
51. 【建党百年征文展】瞻仰革命先烈——牛蹄岭战斗遗址有感（174）
52. 安康汉滨区新党员在牛蹄岭战斗遗址公园集中宣誓（1）
53. 缅怀革命先烈·传承红色文化——华康源党支部赴牛蹄岭战役遗址开展党史学习活动（169）
54. 红色起点·牛蹄岭战斗遗址（131）
55. 汉滨红色记忆六十九 牛蹄岭战役纪念广场（33）
56. 安康市青少年争做红领巾讲解员实践体验活动成功举行（625）
57. 这就是安康少先队员的风采！（2512）
58. 镇坪县教体科技局赴牛蹄岭革命战斗遗址开展党史学习教育主题党日活动（482）
59. 打卡“红色记忆”，一起“陕”闪放光彩！娘家人又有好活动等你来！（1959）
60. 【家乡党史】三秦大地上的红色印记——牛蹄岭战役（109）
61. 【奋斗百年路 启航新征程】浴血重生牛蹄岭 安康人民谱新篇（461）
62. 追寻红色记忆——陕南安康市汉滨区牛蹄岭战役遗址（140）
63. 玩转汉滨超值攻略，值得拥有！|| 897推荐（375）
64. 镇坪县住建局赴牛蹄岭革命战斗遗址开展党史学习教育实践活动（62）
65. “宝塔山下话党史”┃第八集 牛蹄岭战役遗址——解放陕南的关键战役（1631）
66. 汉滨区果园小学党支部在牛蹄岭战斗遗址开展主题党日活动（216）
67. 汉滨区财政局赴安康牛蹄岭战斗遗址开展主题党日活动（261）
68. 【党史学习】红色故事｜激战牛蹄岭 碧血映山河（175）
69. 安康人的心愿实现啦，牛蹄岭战役要拍成电影！（3281）
70. 牛蹄岭战役将拍成电影！（7768）
71. 红色基因，代代永传承！（1406）
72. 红色故事｜激战牛蹄岭 碧血映山河（959）
73. 缅怀先烈奋力前行，安康各地开展祭英烈活动……（2346）
74. 弘扬英烈精神 争做忠诚卫士（529）
75. 安康市委常委班子到牛蹄岭战役遗址开展党史学习教育（805）
76. 【陕西党史】"印记”微党史摄制组走进 牛蹄岭战役遗址（34）
77. 马克思主义学院“安康微红”推送团队清明追思革命先烈，再访牛蹄岭战役遗址主题实践活动（534）
78. 牛蹄岭——红色记忆（2354）
79. 【汉滨红色故事】激战牛蹄岭——安康和平解放（582）
80. 【汉滨红色故事】激战牛蹄岭系列之三：牛蹄岭——雄关要塞（922）
81. 【汉滨红色故事】激战牛蹄岭系列之二：夺取关垭子（1213）
82. 【扩散】好消息！安康牛蹄岭战斗遗址公园一期修复工程建成对外开放（3054）
83. 【提醒】安康牛蹄岭战斗遗址公园一期修复工程对外开放（460）
84. 安康牛蹄岭战斗遗址公园一期修复工程建成对外开放（15）
85. 牛蹄岭战役（617）
86. 那年今日！激战牛蹄岭！（上）（3234）
87. 那年今日！激战牛蹄岭！（下）（1271）
88. 清明节去哪里祭拜烈士？原来安康就有这么多红色旅游景点！（718）

**汉中市洋县华阳红二十五军司令部旧址**

1. 【红色】洋县红二十五军司令部旧址（153）
2. 红色起点·红二十五军司令部旧址（106）
3. 【研学】红二十五军政治部华阳遗址被命名为陕西省青少年教育基地！（46）
4. 忆往昔峥嵘岁月，继续踏上红色旅程（587）
5. 第三站 | 重踏长征路，共筑英雄魂：参观红二十五军政治部华阳旧址（978）
6. 团队党建活动推荐(二)——华阳红25军司令部旧址（70）
7. 百年荣光 | 追寻家乡红色记忆——陕西（194）
8. 汉中红色旅游精品景点推荐（1821）
9. 去过才知道！汉中这个古镇韵味十足，你怎能错过？（1099）
10. 【美丽乡村】夏日炎炎，华阳避暑（109）
11. 红色印迹 | 华阳镇的红色星火——红二十五军司令部旧址（55）
12. 手绘H5 | 红色记忆历久弥新 峥嵘百年与党同行——打卡三秦红色地标（788）
13. 看罢这篇游记，让人想去华阳了（1935）
14. 梳理汉中红色旅游精品景点（197）
15. 西安周边红色教育地点！快收藏！（1083）
16. 仰望长征——访华阳红二十五军司令部旧址（642）
17. 划重点 | 周至这2个地方入选!（66）
18. 【视频】华阳镇·红二十五军司令部旧址（36）
19. 共叙百年奋斗历程 赓续红色基因血脉 第36期｜ 红二十五军司令部旧址（54）
20. 【视频】汉中洋县华阳镇红二十五军司令部旧址（47）
21. 三秦大地上的红色印记丨红二十五军司令部旧址所在地洋县（59）
22. 【视频】洋县·红二十五军司令部华阳旧址（14）
23. 红二十五军政治部旧址在洋县华阳景区正式对外开放（155）
24. 【红色故事会之汉中洋县】红色华阳的峥嵘岁月（35）
25. 追寻红色记忆——陕南汉中市洋县华阳红二十五军司令部旧址（134）
26. 三秦大地上的红色印记 | 红色华阳的峥嵘岁月（113）
27. 省报记者带你领略红军在华阳的峥嵘岁月（484）
28. 追寻红色记忆——陕西汉中（33）
29. 人文学院暑假三下乡（二）| 走进洋县红二十五军司令部旧址（217）
30. 红色记忆，永记心中 ——红二十五军在洋县（36）
31. 省委党史研究室宣教处赴红二十五军司令部华阳旧址调研（56）
32. 2019级美术专业学生参观红二十军司令部旧址，开展爱国主义教育（407）
33. 红二十五军政治部华阳旧址开馆并面向社会开放（284）
34. 省委党史研究室赴红二十五军司令部华阳镇旧址调研（699）
35. 军地检察机关共同调研保护 发扬红色基因（435）
36. 今天，红二十五军政治部华阳旧址开馆啦！（1287）
37. 红二十五军政治部旧址在华阳景区正式对外开放（1513）
38. 百篇陕西红色经典|第八十四篇 红二十五军政治部华阳旧址（156）
39. 永远跟党走 | 红色旅游精品景区推荐之汉中篇（1291）
40. 红二十五军政治部洋县华阳旧址开馆啦！（1370）
41. 汉中这些红色旅游景点您打卡了吗？“行走的党课”启程啦→（1069）
42. 一个外地人眼中的洋县是这样的！（4581）
43. 打卡“红色记忆”，一起“陕”闪放光彩！娘家人又有好活动等你来！（1959）
44. 聚焦 | 今天，红二十五军政治部华阳旧址开馆啦！（801）
45. 汉中“红色之旅”来了，去打卡吧！（5467）
46. 西乡这处红色经典跻身全市红色旅游线路推荐（449）
47. 洋县最新航拍，美爆了！！！（5491）
48. 华阳古镇上的红色火种 ——走进华阳镇红二十五军司令部旧址（58）
49. 每一步都是红色印记！汉中这些红色景点邀你来打卡！（2400）
50. “红色之旅”上线啦！一起看看洋县有什么打卡地（412）
51. 【全警实战大练兵】洋县公安：组织开展“追寻红色记忆、筑牢忠诚警魂”系列活动（二）（389）
52. 圣本动态｜陕西圣本律师事务所参观华阳镇红二十五军司令部旧址，庆祝中国共产党建党101周年（159）
53. 实力圈粉！汉中这些红色旅游景点爆火，你都去过吗？（1074）
54. 感悟汉中红色文化，红二十五军政治部旧址昨日正式对外开放（369）
55. “追寻青春足迹·献礼建党百年”优秀作品展 || 从华阳苏区到华阳古镇（169）
56. 传承红色基因 永葆军人本色——宗营镇退役军人赴华阳红二十五军司令部旧址参观学习（246）
57. 推荐丨重温红色记忆 汉中市推出红色精品旅游线路（110）
58. 【红色洋州】红二十五军在洋县之石塔河战斗（182）
59. 【红色鹮乡记忆】红二十五军在洋县之红色政权组织（557）
60. 市档案馆在洋县华阳红二十五军司令部旧址开展主题党日活动（125）
61. 【党日活动】我院医技支部参观华阳红二十五军司令部旧址（112）
62. 洋县：红二十五军政治部旧址开馆啦（1719）
63. 汉中再登央视，霸屏二十二分钟！带你寻找身边的战争遗迹！（1781）
64. 杨凌新华书店全体党员赴华阳镇红二十五军司令部旧址参观学习（387）
65. “中华秦岭100景”榜单出炉！藏在汉中的那些秦岭美景你都去过吗？（3685）
66. 祝贺！我市三处革命旧址获评“省级党史教育基地”（103）
67. 现实版4K！这才是汉中的正确打开方式~（1121）
68. 重温红色记忆！汉中市红色精品旅游线路推荐！（728）
69. 陕西省第二届全民健身运动会（市区组、行业组）围棋比赛即将在洋县华阳景区拉开帷幕（940）
70. 新鲜出炉！石泉3景入选中华秦岭100景！实力担当（937）
71. “永远跟党走”红色旅游精品线路（1312）
72. 春到长安 | “永远跟党走”红色旅游精品线路（676）
73. 洋州采风行诗友作品选萃（69）
74. 五月主题党日+活动展示（379）
75. 洋县最新航拍纪录片，太美了！（1192）
76. 县委统战部组织机关干部参观红二十五军司令部旧址（225）
77. 陕西省关注森林活动执委会办公室开展调研及主题党日活动（241）
78. 红色引擎在新筑|追寻红色印记 砥砺初心使命（164）
79. 【聚焦】市委书记参观红二十五军政治部华阳旧址 祭奠革命先烈（128）
80. 陕西省红色旅游指南——红二十五军军部旧址（78）
81. 喜讯!函谷关历史文化旅游区入选“中华秦岭100景”（583）
82. 新华社 x 西北大学 || 走华阳入留坝，沿红军足迹寻时代记忆（150）
83. 洋县华阳红二十五军政治部旧址开馆（164）
84. 洋县产学研项目闪亮 “学研在汉中”推介活动周 | 洋县教育（834）
85. 红色大别山——走进红二十五军司令部旧址（23）
86. 汉中市科协组织党员干部学思践悟迎“七一”（423）
87. 【红色洋州】红二十五军在洋县之华阳会议（67）
88. 燃！他们这样为党99周年华诞庆生（858）
89. 国庆红·嗨西安 | 爱国传承9处“红色旅游地”，你都去过吗？（517）
90. 硬核！党的99岁生日，全县各级党组织花式献礼齐上阵！（1356）
91. 汉中市科协党支部联合洋县科协党支部 组织党员干部学思践悟迎“七一”（135）
92. 年轻人都来这里旅游打卡了！陕西“红色旅游”受热捧（219）
93. 红二十五军在华阳（530）
94. @洋县人：拍抖音短视频赢取万元大奖？这是真的！（503）
95. 为了这件事，多位中国书法名家走进汉中（1026）
96. 洋县图书馆党支部开展“喜迎二十大 启航新征程”主题党日活动（79）
97. 红色文化助推陕南革命老区乡村振兴路径探析（310）
98. 【红色鹮乡记忆】红二十五军在洋县之华阳会议（374）
99. 【红色故事会之宝鸡凤县】 凤县革命纪念馆：凤州星火耀苍穹（34）
100. 中国旅游日陕西分会场暨“十百千” 引客入铜活动启动仪式成功举行（58）
101. 洋县摄影家协会开展党史学习教育实践活动（347）
102. 中共汉中市汉台区民政局机关支部委员会开展庆祝建党99周年系列活动（440）
103. 人行洋县支行深入推进网络安全宣传周活动（170）
104. 洋县人大机关念好“三字决”助推党史学习教育提质增效（42）
105. 政协快讯（4月27日）| 加强思想政治引领，提升建言资政能力（405）
106. 明天！陕西景区免费玩！最强攻略速收藏，不然你就亏大了~（4375）
107. 青龙观社区党委热烈庆祝中国共产党成立100周年暨“七一”表彰大会（175）
108. 洋县各级各部门集中开展党史学习教育活动（十二）（576）
109. 洋县创新利用“土”资源助推党史学习教育走深走实（8）
110. 【专栏】洋县教体系统“建功立业新时代”征文⑤｜洋县教育（424）
111. 快去嗨！本周末大唐芙蓉园、秦岭动物园等一大波景点半价，博物馆还免费（280）
112. 【深呼吸小城】“中华秦岭100景”榜单出炉，云屏三峡国家AAAA级旅游景区入选!（94）
113. 5.19中国旅游日，陕西一大波景区免费玩！速度收藏，不然你就亏大了~（699）
114. 党史学习教育丨人大办：念好“三字决”助推党史学习教育提质增效（212）
115. 强党性 取“真经”！ 鹮乡青干班第二站出发……（1419）
116. 庆七一｜县直机关各党组织多种形式庆祝建党101周年（661）
117. 汉中教育系统扎实开展四月份主题党日+活动（837）
118. 6月专题“挖掘本土历史资源、坚定历史文化自信”研学研讨活动——洋县站（134）
119. 学党史、守初心、担使命-陕西省建筑装饰协会党支部主题党日活动（99）
120. 【关注】洋县“小小红色讲解员”总决赛圆满落幕 | 洋县教育（450）
121. 在线访谈 | 汉中市洋县纪委书记郭华做客“纪委书记面对面”（2067）
122. 【探访旧址，寻找初心】第二十期：红二十五军司令部旧址（80）
123. 【寻访札记】寻访红色旧址 感受革命生活 传承革命精神（427）
124. 【二十大专访】漫步汉中，犹如进入一个没有围墙的革命历史博物馆（1）
125. 【杂谈】学习小英雄蒲海水，宁死不屈、至死忠心的精神（376）
126. 【动态】12月8日教育动态 | 洋县教育（940）
127. 庆建党百年丨中天西北集团党委荣获“五星级党组织”称号（3484）
128. 党史学习教育，勉县在行动（347）
129. 【红色鹮乡记忆】红二十五军在洋县建立的革命武（132）
130. 抹不去的记忆 | 红二十五军在洋县（875）
131. 宁强县退役军人事务局联合社会组织赴红25军旧址汲取红色营养（124）
132. 他们为万元大奖奋力拼搏的样子真美！“恋朱鹮游洋县”抖音短视频大赛作品展播（41）
133. 令人心动的主题党日活动，你Pick哪一个？⑦（3069）
134. 洋县：“三个坚持”扎实推进党史学习教育（238）
135. 【动态】6月28日教育动态 | 洋县教育（1116）
136. 全国重点文物保护单位：中国工农红军第二十五军司令部旧址（1054）
137. 生日快乐丨全市教育系统多形式庆祝建党99周年（730）
138. 长征中战斗在洋县的红二十五军！（900）
139. 2021年度洋县目标责任考核 “工作成效大家评”系列网络评选（十五）（5157）
140. 践行初心使命，汉中文旅在行动——文旅融合篇（356）
141. 洋县政协来洋县检察院专题调研公益诉讼工作开展情况（688）
142. 在青春的赛道上奋力奔跑——这个中青班不一“young”！（526）
143. 康红霞丨洋县华阳古镇游记（295）
144. 【交通动态】市公路局开展志愿服务活动助力全国文明城市创建（190）
145. 【红色鹮乡记忆】红二十五军在洋县之石塔河战斗（563）
146. 难忘，那一抹红色 | 张玮（401）
147. 区直机关优秀党组织经验交流展播（5140）
148. 红二十五军在洋县的那些年……（939）
149. 洋县戚氏街道各基层党组织依托主题党日活动，推动党史学习教育有声有色（498）
150. 要闻|市政协机关党史学习教育“重走红军路”活动启动（443）
151. 【庆祝建党100周年】九三学社陕西理工支社组织部分社员进行党史学习实践活动（64）
152. 【随手拍检察蓝】陕检君等你来pick最美的那抹儿蓝（2021年7月优秀摄影作品评选）（3186）
153. 长安银行汉中西环路支行联合汉中市经济技术开发区机关 第三党支部开展主题党日活动（297）
154. 红二十五军在洋县建立的革命武装（57）
155. 全县各级党组织热烈庆祝中国共产党成立101周年（一）（1543）
156. 红色记忆（108）
157. 【动态】4月11日教育动态 | 洋县教育（1095）
158. 汉中市总工会喜迎“七一”系列活动集锦（222）
159. 【转载】他们为万元大奖奋力拼搏的样子真美！“恋朱鹮游洋县”抖音短视频大赛作品展播 | 洋县教育（201）
160. 百年恰是风华正茂 创业园企业这样庆祝建党百年华诞（1069）
161. 【视频】红二十五军在洋县的那些年（43）
162. 汉中职院的一周（824）
163. 彪炳千秋，万古流芳——拜读《华阳风云》游览红色革命胜地（196）
164. 孟丽华【纪实文学】有一种精神叫“周大黑”！（2472）
165. 红二十五军长征到达洋县和华阳苏区后颁布的《告国民党士兵书》（155）
166. 【青柠书鸢·“周大黑朱鹮杯征文”019】天作之缘——“黑珍珠”与“东方宝石”|孟丽华(吉林)（309）
167. 洋县“五一”旅游火爆：累计接待46.36万人次，旅游综合收入2.41亿元（1276）
168. 【培训动态】西安欧中材料科技有限公司党支部“学党史 忆先烈 守初心”参观红色教育基地 深化党史学习教育（12）
169. 传承红色精神 筑牢红色阵地——汉中市环卫处党支部开展四月主题党日活动（510）
170. 【廉政教育】勉县联社开展2022年廉政警示教育活动（62）
171. 【学习百年党史 践行初心使命】这里是葛牌镇革命旧址（185）
172. 周治富：华阳游记（197）
173. 中国工农红军第二十五军司令部旧址（27）
174. 佛坪县退役军人事务局组织部分优抚对象开展党史学习教育（317）
175. 市统计局组织党员干部赴洋县华阳开展革命传统教育（320）
176. 红二十五军在洋县，留下了哪些感人的故事？（1722）
177. 汉中市人大常委会办公室2019年上半年工作总结（2335）
178. 洋县革命根据地遗址，大家知道吗！（289）
179. 经典案例｜红色旅游深度探索和规划实践（1358）
180. 周末休闲好去处｜全省文化旅游名镇——华阳古镇（1.5万）
181. “追寻红色记忆、弘扬红色文化”教育活动（111）
182. 洋县华阳古镇踏青之旅（九）工农红军二十五军司令部旧址（30）
183. 汉中旅游推荐线路： 红色经典旅游线路（558）
184. 纪念抗战胜七十周年之红二十五军在华阳（127）
185. 洋县华阳二十五军司令部旧址又添新的头衔！（639）
186. 红二十五军在华阳古镇十二天的红色记忆（119）
187. 华阳要闻 | 二十五军司令部旧址又添新的头衔！（434）
188. 每天上万游客 | 洋县华阳景区红25军司令部旧址 感怀长征路精神（1804）
189. 【陕西乡村旅游十百千品牌推荐】十大旅游名镇之一|洋县华阳古镇（326）
190. 【洋县之行】闪闪红星，铭记历史（107）
191. 【家乡的红色记忆】红25军在洋县（上）（566）
192. 秦岭深闺第一镇，曾是巴蜀古道的著名驿站，如今与云南大理相媲美！（6731）
193. 汉中这座与大理相媲美的秦岭深闺第一镇，曾是秦蜀古道的著名驿站（2068）
194. 【自驾活动】醉美汉中洋县“金色花海”“华阳古镇、景区”两日游招募开始啦！赠大礼（797）
195. 王燕调研指导红二十五军政治部华阳遗址布展工作（366）
196. 安吴青训班入选全国红色游经典景区，红色泾阳欢迎您！（2030）
197. 洋县教育暑期回眸（1481）
198. 汉中这座秦岭深闺第一镇，曾是秦蜀古道的著名驿站！（1967）
199. 【动态】6月27日校园动态（1080）
200. 市委党史研究室主任王燕调研指导红二十五军政治部华阳遗址布展工作（62）
201. 一样的古镇，不一样的华阳！一起来看华阳新“网事”（1531）
202. 【动态】5月4日校园动态速递（1594）
203. 周边踏青| 本周末我们去的这个地方美到逆天，你还不约吗？（2114）
204. 周边踏青丨人间三月天，让我们在洋县花海情深处，品味小江南！（2151）
205. 活动记录丨我们畅游在山间与花海，就像是走进诗歌和童话~（1214）
206. 周边踏青| 这里曾承载着一个美好的故事，周末我们一起去聆听吧！（1162）
207. 50年前在洋县插队过的知青，今天给华阳景区无偿捐赠”红色钱币“！（4600）
208. 洋县华阳红25军司令部旧址 每日接待游客过万（740）
209. 教体局到华阳开展党风廉政教育（1721）
210. 我们电视台又岀大片啦！《重走长征路▪寻找六安人》等你来看！（690）
211. 石塔河的枪声（100）
212. 周末福利】官微君带你去恋爱吧👫！（285）
213. 陕西理工大学走进洋县红石窑村，开展青年红色筑梦之旅（391）
214. 活动招募丨在这里，让我们的爱情如花一样绽放（1143）
215. 红二十五军长征中不能不提的五次战斗（369）
216. 三下乡 | 陕理工学子在华阳：学习长征精神，传承红色文化（126）
217. 【旅游百事通】7月16日华阳古镇观秦岭四宝、探傥骆古道—龙吟峡深度二日游：430元/人（21）

**西乡县红二十九军军部旧址**

1. 汉中红军遗址，西乡私渡镇鸡公田起义旧址，红二十九军军部遗址（180）
2. 红领巾学党史 | 马儿崖悲歌（23）
3. 红色引领促发展 税检联合迎七一（48）
4. 马儿崖的“灵魂“洗礼（613）
5. 全国红色景点展馆展播——陕西篇（33）
6. 建党百年之际，带你畅游西北大地上的红色经典景区（134）
7. 陕西红色革命纪念地摄影美图大放送：汉中篇！（364）
8. 【媒体关注】（174）
9. 汉台、西乡两区县摄影家赴西乡茶乡、骆家坝红29军旧址采风（237）
10. 探访汉中西乡红29军军部，仅存一个窝棚，当年2个月遭到失败！（294）
11. 安康铁检在红二十九军军部旧址开展党建活动（213）
12. 建党节，带您走遍大西北红色旅游景点景区（22）
13. 汉中多家景区入选“中华秦岭100景”榜单！（20）
14. 暑期“三下乡” | 机械工程学院“红”细胞暑期社会实践队赴汉中市开展党史学习教育实践活动（56）
15. 主题党日学党史，这些活动很亮眼！（361）
16. 汉中10个旅游景区入选“中华秦岭100景”（171）
17. “中华秦岭100景”榜单出炉，勉县这个景区入选（286）
18. 中华秦岭100景、大秦岭边际最美风景名单：陕西最多，商洛7个，山阳柞水最抢眼，景区数量最多的洛南县意料之中还是出乎意料？（336）
19. 忆党史 铭党恩 强党性 ——中所中学党支部开展庆祝建党100周年系列活动（174）
20. 西乡私渡河红军驿站打卡46
21. “中华秦岭100景”榜单出炉，秦岭佛坪国宝旅游度假区这个景区上榜啦！（25）
22. 西乡县旅游行业协会欢迎全国游客来西乡县骆家坝国家AAAA级景区游玩（299）
23. 【党史学习教育（四）】旬阳市 | 西乡县 | 五华县 | 平阳县（109）
24. 【信·三下乡】历百年党史艰辛岁月，看盛世秦地惊艳四方（62）
25. 【风沐三秦~迹循故地】天汉瞻仰英灵魂（13）
26. 解密！陕南红二十九军失败的真正原因！（729）
27. 全中!光雾山景区、米仓山景区、诺水河景区入选“中华秦岭100景”（824）
28. “中华秦岭100景”和“大秦岭边际线上的最美风景”重磅推出（627）
29. 【红色旅游】陕西省13个红色旅游景区列入全国红色旅游经典景区名录（351）
30. 【陕西红色旅游】100处红色旅游目的地（下篇）：延安/榆林/汉中/安康/商洛/杨凌（9）
31. 新鲜出炉！汉江燕翔洞生态旅游区入选中华秦岭100景！实力顶流担当，养了眼、醉了心！（229）
32. 全市教育系统高标准高质量推动党史学习教育③（694）
33. 陕西党史汉中篇：1933年，汉中成立红军29军，两个月遭到失败，教训深刻（337）
34. 庆祝中国共产党97华诞，书院党支部这样过……（1019）
35. 清廉陕西 | 激活传播阵地 厚植廉洁根基（3508）
36. 汉风汉韵汉中年|2019汉中春节旅游攻略之西乡、勉县篇（942）
37. 主题教育守初心 志愿服务暖人心（260）
38. 骆家坝的麻柳树（115）
39. 陕西蓝波湾集团 “不忘初心、重温记忆” 优秀员工团建活动（254）
40. 他造成红29军全军覆没, 抗战中立下战功, 起义成解放军副司令员（395）
41. 喜迎党的百年华诞！300处红色经典旅游景点，回首红色百年路（96）
42. 全国红色旅游经典景区名录（198）
43. 旅游：全国红色旅游300处经典景区名录（329）
44. 全国红色旅游经典景区名录公布 300处景区入选（12）
45. 孟芳洲（11）
46. 1930年，中国共产党陕南特别委员会在汉中千年古寺召开第一次代表大会（309）
47. 作风能力建设丨汉中市教育系统推进“作风能力建设专项行动”走深走实⑧（667）
48. 童心向党耀西乡 红色基因代代传 ——西乡二中高秋85级同学会六一红色之旅活动（316）
49. 【辉煌百年路 奋进新鹰城】红九军诞生地：红色精神永流传（3373）
50. 东征西战的红二十八军及红二十九军的组建（508）
51. 【西安旅游】陕南红军革命根据地系列景区（84）
52. 陕西13家景区入选全国红色旅游经典景区名录（909）
53. 历史回放|西乡·红色故里，鸡公田起义旧址（30）
54. 又上榜了！凤县被全国红色旅游点名了（1276）
55. 铜川市陕甘边革命根据地旧址列入全国红色旅游经典景区名录（560）
56. 陕西理工学院暑期“瞻仰抗战遗迹”大学生纪念抗战胜利70周年寻访活动圆满结束（168）
57. 陕西省13个红色旅游景区列入全国红色旅游经典景区名录（22）
58. 《全国红色旅游景点景区名录》公布 凤县两当起义纪念地榜上有名（670）
59. 《全国红色旅游经典景区名录》发布，泾阳安吴青训班入选！（683）
60. 在宝鸡拥有这张卡的小伙伴们有福了！可“刷脸”免费游这些景区！（10）
61. 以史为鉴！震惊陕南地区的马儿岩事变！（439）
62. 解读 | “马儿崖事变”八十周年祭（终篇）（604）
63. 全国红色旅游经典景区名录公布，红旗渠赫然在榜！（1446）
64. 【旅游】西乡骆家坝镇文化周开始啦！（79）

**西乡县红四方面军总后医院旧址**

1. 很多人不知道汉中有一个必须去的地方！（490）
2. 这个夏天西乡樱桃王子带你走进大河镇 十八怪（1493）
3. 西乡樱桃王子618大河行西乡新媒体协会第一次筹备会成功召开（259）
4. 西乡县9处革命旧址入选！（153）
5. 看“门户村”开启的“党建+”模式（975）
6. 西乡新闻快讯，速览→→→（372）
7. 这次红色精神之旅，他们满载而归（940）
8. 红四方面军在这里作出建立川陕革命根据地的重要决定……（1784）
9. 【特别关注】《全国红色旅游经典景区名录》公布300处景区入选 有你家乡吗（498）
10. 喜迎二十大：汉中监狱基层党支部在行动（868）
11. 【寻访札记】西乡检察院到大河镇寻访革命旧址 追寻红色记忆（267）
12. 全国300处红色旅游经典景区名录发布 山东省13处入选（53）
13. 《全国红色旅游经典景区名录》公布（112）
14. 一座大院的红色记忆——中展利用复原陈列技术，再现“全国重点文物保护单位-通江红四方面军总医院”旧貌（349）
15. 《全国红色旅游经典景区名录》,你知道几个？（171）
16. 旅游：全国红色旅游300处经典景区名录（22）
17. 【国庆假期】2019新版中国红色旅游景区名录，太实用了！（56）
18. 【招商引资】2017年汉中重点推介招商引资项目——西乡县红四方面军暨红二十九军革命遗址建设项目（30）

**3.延安市延安革命纪念地系列景区(延安革命纪念馆,枣园革命旧址,杨家岭革命旧址,王家坪革命旧址,凤凰山革命旧址,清凉山革命旧址,“四八”烈士陵园,洛川县洛川会议纪念馆,子长县瓦窑堡会议旧址,宝塔山景区,桥儿沟革命旧址,南泥湾革命旧址,中共中央西北局革命旧址,陕甘宁边区政府旧址,志丹县保安革命旧址,吴起镇革命旧址,中国人民抗日军政大学纪念馆)**

1. **中国人民抗日军政大学纪念馆**
2. 听我校刘红老师畅谈：《延安颂》中的“中国精神”（501）
3. 学习贯彻党的二十大精神(21)】习近平：弘扬伟大建党精神和延安精神 为实现党的二十大提出的目标任务而团结奋斗（46）
4. 李延声：群星闪耀延河边（467）
5. “喜迎二十大 永远跟党走 奋进新征程”2022年全国大学生延安暑期社会实践专项活动合辑（二）（511）
6. 国庆假期红色旅游持续火热！这些红色“打卡地”你都去过吗？（1399）
7. 延安抗日军政大学纪念馆（38）
8. 弘扬延安精神 创新文旅发展（1044）
9. 实训基地丨延安地区（20）
10. 传承红色基因 赓续精神血脉｜州政协机关开展党史学习教育纪实（197）
11. 党史故事 20丨延安抗日军政大学（63）
12. 暑期红色社会实践丨“跟党走，忆初心，担使命——追溯延安根，砥砺新征程”（1736）
13. 湖工青年的入党誓词，在延安响起！（1531）
14. 从延安到北安⑭｜抗大：三句话，八个字，钢铁的干部在这里养成（749）
15. 延安市延安革命遗址之中国人民抗日军政大学旧址（66）
16. 党史学习教育||“学党史促成长——党史教育进商会”第二期主题活动--走进延安抗日军政大学纪念馆（37）
17. 【江工人学党史】中国人民抗日军政大学旧址探访：十万将士出“抗大”（78）
18. 寻找烈士苌征亲人的前前后后（3882）
19. 【党史今日】1937年1月20日，中国人民抗日军政大学（简称抗大）在延安成立（162）
20. 中国人民抗日军政大学纪念馆文物史料征集启事（32）
21. 中国人民抗日军政大学陈列馆被评为国家二级博物馆（4）
22. 全国红色景点展馆展播——陕西篇（134）
23. 【培训·动态】观摩延安实验小学，探访抗大纪念馆（522）
24. 中国人民抗日大学（简称“抗大”）纪念馆（31）
25. 陕西有10处国家级抗战纪念设施、遗址，咱延安就有6处！（569）
26. 圣地延安 民族希望 ——写在中国人民抗日战争暨世界反法西斯战争胜利75周年之际（92）
27. 恢复开放分类施策 疫情防控毫不放松——革命纪念地有序恢复开放（2030）
28. 延安凤凰山革命旧址（中国人民抗日军政大学纪念馆）恢复开放公告（473）
29. 中国人民抗日军政大学纪念馆自1月24日起闭馆 恢复开放时间另行通知（408）
30. 【红典心声】来延安接受红色教育 这两所曾经的学府一定要去（8）
31. “不忘初心 牢记使命”主题教育基地 |中国抗日军政大学（42）
32. 长安大学赴延安“承先烈伟志·育爱国青年”暑期社会实践圆满结束（221）
33. 延安之行·肆 | 系革命情怀 弘延安辉煌（47）
34. 暑期社会实践| 赴延安凤凰山麓革命旧址暨抗日军政大学纪念馆学习调研（268）
35. 【红色记忆】中国革命圣地、红色摇篮——延安⑤（82）
36. 【红色延安】现场教学之中国人民抗日军政大学纪念馆（100）
37. 从延安精神中汲取力量（5161）
38. 学校组织党员干部赴延安开展培训（1896）
39. 【延安专辑】中国抗日军政大学纪念馆（155）
40. 线路在手，寻梦延安！（50）
41. 青，怀远团干部带你延安寻根（411）
42. 十九大精神照我前行|优秀团干延安行！他们都学了啥？（1123）
43. 追寻革命历史 感悟延安精神（128）
44. 延安寻根 北京外国语大学延安抗大纪念馆校址纪念碑揭碑（781）
45. 黑龙江大学与抗大纪念馆签订全面合作协议（407）
46. 【光辉历程】十万将士出“抗大” ——中国人民抗日军政大学旧址探访（9）
47. 【光辉历程】中国人民抗日军政大学旧址探访（285）
48. 中国人民抗日军政大学纪念馆入选第二批国家级抗战纪念遗址（3）
49. **视频：36**

**吴起镇革命旧址**

1. 、红色传承专栏||忆延安，昔往日“岁月峥嵘”（585）
2. 【党的故事我来讲】中央红军二万五千里长征落脚吴起镇的故事——薛志金（86）
3. 【红色记忆】开始长征⑧：奔向吴起镇‖蔡长风(（54）
4. 【转发】红色寻踪：吴起县红色文化追旅（2042）
5. 追随领袖足迹，感悟思想伟力，体会延安精神（2093）
6. 陕西延安：小苹果助力富民兴村 红色文化激励振兴发展（120）
7. 长城主题国家级旅游线路发布，陕西“刷屏”了！（1642）
8. 吴起中央红军长征胜利纪念园（149）
9. 若你厌倦了大城市的喧闹，不妨来吴起领略不一样的美！（11）
10. 花染春意 吴起邀您共赏满城春色!（861）
11. 【海气|学党史】“寻访红色地标专题行动”第五十八站：吴起镇革命旧址纪念馆（77）
12. 红船破浪：吴起革命旧址（5）
13. 红旗漫卷——陕西革命旧址云上展：吴起革命旧址（1）
14. 红旗漫卷——陕西革命旧址云上展（吴起镇“切尾巴”战役遗址）（32）
15. 陕西省吴起县，毛主席长征结束前最后一处旧居，依山而建的窑洞（2992）
16. 红旗漫卷——陕西革命旧址云上展之八十一：吴起镇“切尾巴”战役遗址（98）
17. 【夜读•党史故事】征师胜利到吴起（1277）
18. 征师胜利到吴起（94）
19. 【教育基地】吴起中央红军长征胜利纪念园（20）
20. 党史上的今天丨10.22（3）
21. 【百炼成钢·党史上的今天】1935年10月22日，中共中央政治局在陕北吴起镇召开会议（2）
22. 1935年，中央红军到达吴起镇，从此陕北成为中国革命中心（376）
23. 延安“红色之旅”来了，一起去看宝塔山！（5590）
24. 【缅怀革命先烈，赓续长征精神】| 峥嵘岁月实践团—寻红之旅第七站：吴起镇（20）
25. 吴起革命纪念馆关于最新疫情防控措施的通知（192）
26. 长征胜利地，吴起乡音亲（255）
27. 【荐读】打卡红色圣地，追寻革命精神（524）
28. 《到吴起镇》| 毛主席和中央红军在吴起的13天（95）
29. 百年荣光|革命圣地-延安（216）
30. 新时代跟着总书记讲党史故事丨革命圣地延安（249）
31. 【党史学习教育】武警白山支队 | 金昌市金川区 | 吴起县 | 晴隆县 | 渝中第一离休所（202）
32. 红色旧址（一） | 延安红色革命地（55）
33. 新书预告 | 《中央红军长征落脚点——吴起革命纪念馆 吴起中央红军长征胜利纪念馆》（39）
34. 党史天天学|长征——吴起镇战斗（169）
35. 吴起革命纪念馆数字化自助讲解正式上线—请大家扫码体验（81）
36. 党史学习教育进行时之百名青年党员讲党史|第三十四讲：吴起镇—中央红军二万五千里长征在这里胜利结束（102）
37. 奋斗百年路·启航新征程·党史学习教育 | 到达吴起，迎来胜利（41）
38. 到达吴起，迎来胜利（2.1万）
39. 革命圣地照亮神州① 吴起镇——并非偶然的“落脚点”（218）
40. 延安市吴起县中央红军长征胜利纪念园（84）
41. 再走长征路|寻访吴起镇革命旧址（76）
42. 吴起县中央红军长征胜利纪念园创建国家4A级景区迎接省检验收（1118）
43. 【学在大华】红色景点之吴起革命纪念馆（24）
44. 延安吴起县：陕北有个县城以战国大将名字命名，现因中央红军长征名扬天下（695）
45. 吴起县政协调研全县革命遗址和历史遗迹保护工程（80）
46. 【吴起融媒】吴起县政协调研全县革命遗址和历史遗迹保护工程（646）
47. 吴起革命旧址（28）
48. 【延安革命遗址 二十二】中央红军长征落脚点——吴起镇（10）
49. 【中红延育】吴起镇革命旧址“那年的岁月”（21）
50. 【转载】中共中央和中央红军长征经华池县铁角城到达陕甘革命根据地辖域吴起镇（470）
51. 【文清说党史】 陕北吴起镇（1）
52. “十一”去哪玩儿？ 吴起邀您来！（97）
53. 快闪 | 长征落脚点—吴起镇（36）
54. 吴起县组织全体县级领导干部到中央红军长征胜利纪念园接受革命传统再教育（41）
55. 市人大来吴起县开展《延安革命旧址保护实施办法》立法调研（168）
56. 西北革命根据地的吴起镇———中央红军长征胜利的落脚点（51）
57. 记者再走长征路│寻访吴起革命旧址│ 聆听黄土高坡上的红军情（50）
58. 《中国红色旅游影像志》征集启事（958）
59. 您的摄影作品，有机会入选《中国红色旅游影像志》| 征集（2995）
60. 【辉煌70年】长征落脚点的红色记忆：长征精神 永放光芒（3）
61. 延安吴起县：参观吴起革命旧址（643）
62. 吴起革命遗址一日游（3）
63. 感受长征精神就去吴起胜利山（26）
64. 【薛书记今日播报·革命圣地延安吴起镇】每天60秒，累计播报新闻1934期（204）
65. 吴起 吴起（34）
66. 【工作会议】《吴起县革命老区发展史》编纂工作会议召开（7）
67. 吴起镇，红军长征的落脚点（66）
68. 【印象采三】参观吴起革命纪念馆（335）
69. 大美吴起丨红色土地 英雄故里（415）
70. 红色圣地——吴起镇革命旧址（284）
71. 【重走红军长征路】第二十四篇：《吴起镇：中国革命出发点，踏上全面小康新征程》（41）
72. 厉害了！“我心中的长征纪念地”全国才入选20个 延安就占了3个（1145）
73. 【喜讯】巴中一红色旅游景区上榜“我心中的长征纪念地” 全国仅20个（327）
74. 【延岸良品】带你畅游吴起！（990）
75. 长征胜利80周年｜吴起：中央红军长征落脚点（456）
76. 征途 | 吴起的红色记忆（3803）
77. 【重走长征路】探访红军长征会师地吴起:铁律军魂，薪火相传！（177）
78. 长征精神耀法院】吴起：从胜利走向更伟大的胜利（367）
79. 红色记忆 | 探访红军长征会师地吴起：铁律军魂 薪火相传（270）
80. 延安保安、瓦窑堡、吴起和延川永坪一个都不能少！（842）
81. 11位大学生，356小时，4659公里，只为向同样青春的他们致敬！（4589）
82. 重走长征路｜10月12日跟记者一起红色之旅！（114）
83. 【不忘初心再长征】中央红军长征胜利到吴起（300）
84. 重走长征路｜红军三次翻越夹金山创造奇迹（173）
85. 延安旅游景点（吴起镇革命旧址）（25）
86. 重走长征路｜“小鲜肉”团发回最新照片（215）
87. 【不忘初心再长征】毛泽东杜梨树下指挥“切尾巴”战斗（359）
88. 重走长征路｜有人冒充《今日》！报名请看清！（423）
89. 【青春闪耀长征路·第十七期】陕西·吴起-江山如此多娇，引无数英雄竞折腰（378）
90. 抗战主题和红色景点成为我省旅游热门目的地（99）
91. **视频：33**

志丹县保安革命旧址

1. 【以案释法】陕西省志丹县人民检察院督促保护保安革命旧址行政公益诉讼案（3）
2. 老游历史板话 二八七 刘志丹陵园（3）
3. 【志丹电商】志丹县十大旅游景点——保安革命旧址纪念馆（16）
4. 汪建业||参观保安革命旧址感赋（6首）（193）
5. 十一假期去哪儿玩？公益诉讼检察官倾力推荐……（606）
6. 延安市志丹县：红色教育培训赋能乡村振兴（212）
7. 保安革命纪念馆（120）
8. 志丹县举行东润启航奖学金 益师奖捐赠发放仪式（233）
9. 【媒体看检察】法治日报：能动履职持续深化红色资源保护（384）
10. 主题党日活动 | 同心筑梦跟党走 公益有我新征程（903）
11. 陕西志丹县：讲好红色故事，传承红色基因 | 共青团基层改革进行时（1236）
12. 志丹县图书馆 | “阅美三秦，从这里开始”4.23世界读书日陕西省“分享读书”短视频作品展播（16）
13. 阅美三秦 从这里开始 | 红星从这里照耀中国（57）
14. 保安革命旧址（23）
15. “正月里来是新年，陕北出了个刘志丹……”人们把对英雄的怀念唱进了民谣（5.4万）
16. 八十多年前，这位美国记者写下一部不朽经典，首次告诉世界红星如何照耀中国（5.8万）
17. 云上畅游，每日VR一景：志丹县保安革命旧址纪念馆（8）
18. 迈开稳稳的步伐 踏出精彩的回响——从最高检发布的典型案例看公益诉讼检察发展变化（483）
19. 【追忆党史】红色故事咏流传，党史文化心中记：保安革命旧址（15）
20. 2021，案例陕检（1406）
21. 2021，公益诉讼检察大事记（1.8万）
22. 关于志丹县保安革命旧址、中国人民抗日红军大学旧址暂停开放的公告（296）
23. 红旗漫卷——陕西革命旧址云上展（保安革命旧址）（8）
24. 百年党史百日学丨保安革命旧址（39）
25. 今天《参考消息》整版刊发新华社记者采写的关于我旗“埃德加·斯诺觉醒点”文章（1289）
26. 西电前身西北军委通信学校志丹办学旧址寻访记，59名学员从这里学成毕业（683）
27. 国家税务总局志丹县税务局：传承红色基因 汲取奋进力量（233）
28. 党课第三弹 | 保安革命旧址（39）
29. 老城遗珍 · 马路的故事｜志丹路，提醒我们不要忘记这位革命先烈（270）
30. 红船破浪：保安革命旧址（134）
31. 志丹保安革命旧址保护行政公益诉讼起诉案例入选最高检典型案例（63）
32. 【寻访特辑】刘惠生检察长周末到志丹县寻访革命旧址（174）
33. 刘惠生周末到志丹县寻访革命旧址（1162）
34. 【寻访札记】窑 洞 漫 记（639）
35. 到“红色中国”去！（2.5万）
36. 检察日报｜红色资源保护是篇“大文章”——陕西延安保安革命旧址绿植满满风貌一新（275）
37. 这两处珍贵的革命文物经历了什么？做好红色资源保护大文章（138）
38. 关于志丹县保安革命旧址、中国人民抗日红军大学旧址临时关闭的公告（536）
39. 《延安13年·沿着先辈的足迹》（志丹篇）第一集 毛主席红星八角帽照片：来自志丹石窑前的留念（444）
40. 破坏革命文物风貌，依法起诉！（278）
41. 【典型案例】陕西省志丹县人民检察院督促保护保安革命旧址行政公益诉讼案（93）
42. 居民违建严重破坏革命文物历史风貌，检察机关督促拆除（5）
43. 甘肃这例入选！最高检和退役军人事务部联合发布红色资源保护公益诉讼典型案例（11）
44. 竟在保安革命旧址上违建？治理违法建设，维护公平正义！（1190）
45. 福建晋江检察院一起案例入选最高人民检察院和退役军人事务部联合发布的红色资源保护公益诉讼典型案例（1604）
46. 平江县这个案例入选最高检和退役军人事务部联合发布的红色资源保护公益诉讼典型案例（256）
47. 相关负责人就红色资源保护公益诉讼典型案例答记者问（2999）
48. 志丹县举行“光荣在党50年”纪念章颁发仪式（1711）
49. “弘毅计划”实践招募｜陕西志丹：红都山河不朽 非遗文脉相传（205）
50. 志丹：让百年党史“飞入寻常百姓家”（202）
51. 奋斗百年路 启航新征程 | 保安县，为他改名为志丹县（1011）
52. 探访革命圣地，追寻红色记忆之西北精品线路（584）
53. 权威发布 | 志丹县“十四五”规划 和二〇三五年远景目标纲要（第一篇）（413）
54. 苏斌到安塞区、志丹县调研黄河流域生态保护和革命文物保护利用工作（2756）
55. 志丹县：文明实践“沉下去” 党史学习“活起来”（280）
56. 追寻红色印记 传承革命精神——宝塔区检察院第四检察部赴志丹开展党性教育（376）
57. 志丹县委理论学习中心组在保安革命旧址纪念馆开展党史学习教（339）
58. 【八个课堂--“初心课堂”】发扬抗大精神，凝聚奋进力量——志丹县人民检察院组织干警赴保安革命旧址开展红色教育活动（46）
59. 【队伍教育整顿】发扬抗大精神，凝聚奋进力量——志丹县人民检察院组织干警赴保安革命旧址开展红色教育活动（280）
60. 追寻志丹 体验红都（746）
61. 延安市志丹县保安革命旧址（116）
62. 分享一篇文章。（12）
63. 陕西曝光13家单位！只因……（2.9万）
64. 陕西：火灾隐患曝光（一）（78）
65. 基层动态|志丹县民政局坚定信心跟党走 担当作为感党恩（13）
66. 志丹县新型冠状病毒感染的肺炎 疫情防控工作领导小组 公告（第2号）（4500）
67. 红色旅游篇——保安革命旧址（49）
68. 以人民为中心！“洋记者”访革命圣地延安（2.2万）
69. 彭安季来志丹县开展《延安革命旧址保护实施办法》立法调研（206）
70. 志丹，中国革命的第二个红色首都（586）
71. 【记者再走长征路】走进志丹保安革命旧址 感知红色“革命熔炉”（594）
72. 重温革命历史 感悟初心使命（38）
73. 【七一】邵阳：寻根革命圣地 奋发砥砺前行（200）
74. 寻根革命圣地 奋发砥砺前行——邵阳市检察机关“不忘初心 牢记使命”党务干部培训班侧记（600）
75. 延安志丹县：参观保安革命旧址（430）
76. 【媒体聚焦】苹果为什么这样红———英雄故里志丹县的转型探索（64）
77. 红都之旅“八部曲” —— 畅游志丹攻略（855）
78. 让红色基因在志丹大地代代相传——志丹县党员干部开展红色教育小记（21）
79. 牢记初心使命 我为党旗添彩—志丹县公安局交警大队党支部组织全体党员走进刘志丹烈士陵园、抗日红军大学、保安革命旧址接受革命传统教育（1096）
80. 【经验】命名志丹县“党性教育基地”（82）
81. 保安中央旧址——我的长征路（169）（34）
82. 革命圣地之县城风采！（59）
83. 革命圣地 红色延安（志丹篇）（27）
84. 红色基因•中国心️徳邦足迹万里行～走进陕西志丹县保安革命旧址（40）
85. “非热门系列”|“十一”出游， 这些地方人少（2013）
86. 【红色旅游】志丹县--中国人民抗日红军大学旧址（81）
87. 走进红都志丹 忆革命峥嵘岁月（103）
88. 红色圣地——保安革命旧址（179）
89. 志丹县文物管理所（保安革命纪念馆）讲解员业务考核（68）
90. “红都保安、将军故里”：志丹县文化工作剪影（18）
91. 志丹县刘志丹烈士陵园和保安革命旧址·中国人民抗日红军大学旧址迎接国家3A级旅游景区评定验收（434）
92. 志丹县：依托红色教育资源 加强党员党性教育（342）
93. 【投票】请为“我心中的长征纪念地——保安革命旧址”投上您宝贵的一票！（54）
94. 延安旅游景点（保安革命旧址）（65）
95. 志丹县保安革命旧址纪念馆（30）
96. 【红都志丹】志丹县保安革命旧址纪念馆（89）
97. 【党史】保安革命旧址纪念馆（42）
98. 纪念抗战胜利70周年广播户外主题体验活动第二站在志丹县保安革命旧址圆满结束《木木读诗》《你好延安》（193）
99. 【抗战我们来讲述】纪念抗日战争胜利70周年大型广播户外直播主题体验活动第二站明日在志丹县保安革命旧址启动（80）
100. **视频：40**

**陕甘宁边区政府旧址**

1、党史学习||【建党百年事记】——1937（1）

2、马蹄沟为什么被称为中国革命的第二个延安（727）

3、关于陕甘宁边区政府旧址管理处陈列展览改造期间不对外开放的公告（129）

4、陕甘宁边区政府旧址（92）

5、红色证券遗珍｜陕甘宁边区政府建设救国公债（150）

6、童心向党 革命旧址少年说--陕甘宁边区政府旧址小小讲解员风采展示（四）（123）

7、【历史文化】陕甘宁边区政府的“扶贫”之路（24）

8、童心向党 革命旧址少年说--陕甘宁边区政府旧址小小讲解员风采展示(三)（49）

9、陕甘宁边区政府旧址管理处组织全体党员干部职工观看警示教育片（49）

10、浅谈陕甘宁边区政府防疫政策（374）

11、化学学院‖“三下乡”社会实践活动（八）——传承红色基因，激发爱国情怀——陕甘宁边区政府旧址篇（89）

12、陕甘宁边区政府旧址管理处传达学习局党委第五次中心组学习会议精神（46）

13、陕甘宁边区政府旧址管理处传达学习局党委第四次中心组学习会议精神（35）

14、【西部风物】陕西延安：陕甘宁边区政府旧（18）

15、【党史知识】红旗漫卷——陕西革命旧址云上展：陕甘宁边区政府旧址（1615）

16、游遍陕西——延安地区（414）：陕甘宁边区政府旧址（17）

17、【公安红色印记】陕甘宁边区政府保安处（100）

18、【党史学习】陕甘宁边区政府成立（45）

19、海警学党史丨陕甘宁边区政府成立（177）

20、百年征程 不忘初心｜百年瞬间：陕甘宁边区政府成立（114）

21、百年瞬间丨陕甘宁边区政府成立（第二百四十五期）（48）

22、红色灯塔 | 珍贵的陕甘宁边区政府文件选编原件陈列（148）

23、【特别话题·百年党史】延安时期陕甘宁边区政府的“扶贫”之路（283）

24、陕甘宁边区政府旧址管理处国庆节前安全检查（41）

25、陕甘宁边区政府旧址，这里是新中国成立的雏形（9）

26、陕甘宁边区政府成立（970）

27、【百年瞬间】陕甘宁边区政府成立（23）

28、党史日历丨这天，陕甘宁边区写入历史（41）

29、今日党史‖陕甘宁边区政府成立（47）

30、百炼成钢·党史上的今天：1937年9月6日，陕甘宁边区政府成立（55）

31、党史故事·百年瞬间｜陕甘宁边区政府成立（23）

32、党史日历（9月6日）｜陕甘宁边区政府正式命名（13）

33、红色血脉——党史军史上的今天丨9月6日 陕甘宁边区政府（598）

34、延安革命旧址——陕甘宁边区政府（28）

35、陕甘宁边区政府旧址管理处学习党的二十大通过的《中国共产党章程（修正案）》（35）

36、杨家岭革命旧址一行赴陕甘宁边区政府旧址参观学习（316）

37、政治交接主题教育·传统教育基地巡展 | 延安陕甘宁边区政府交际处旧址（52）

38、童心向党 革命旧址少年说--陕甘宁边区政府旧址小小讲解员风采展示（253）

39、行走笔记-延安市-宝塔区-陕甘宁边区政府旧址（39）

40、陕甘宁边区政府旧址管理处2022第二季度消防知识培训演练（58）

41、【文化旅游】绥德红色革命旧址---陕甘宁边区政府机关驻地（126）

42、陕甘宁边区政府旧址暂停开放通知（104）

43、红旗漫卷——陕西革命旧址云上展（陕甘宁边区政府参议会礼堂）（13）

44、陕甘宁边区政府成立（21）

45、【百年党史】陕甘宁边区政府成立（5）

46、党史上的今天丨陕甘宁革命根据地的苏维埃政府正式改称陕甘宁边区政府（52）

47、薛耀军调研指导陕甘宁边区政府交际处旧址保护修缮工程（748）

48、陕甘宁边区政府旧址管理处党支部 召开党史学习教育专题组织生活会（41）

49、交际处旧址保护提升改造项目加快建设 预计年底全面开放（330）

50、参观陕甘宁边区政府旧址景区疫情防控须知（166）

51、红色印记｜峥嵘岁月铸伟业——探访陕甘宁边区政府旧址（58）

52、”筑筑有话讲！“ 第一站：陕甘宁边区政府旧址（74）

53、陕甘宁边区政府旧址管理处关于调整讲解收费标准的公示（220）

54、【1077最关注】陕甘宁边区政府交际处旧址预计年底全面对外开放（47）

55、延安12345 | 陕甘宁边区政府交际处旧址预计年底全面对外开放（391）

56、陕甘宁边区政府交际处旧址预计年底全面对外开放（89）

57、延安市延安革命遗址之陕甘宁边区政府旧址（46）

58、陕甘宁边区政府交际处旧址：保护修缮工作进展顺利 7月底对外开放（409）

59、百篇陕西红色经典|第六十七篇 陕甘宁边区政府旧址（194）

60、陕西革命旧址云上展（十一）| 陕甘宁边区政府参议会礼堂（65）

61、【党史学习】重游初心之地⑨——陕西革命旧址：陕甘宁边区政府旧址（124）

62、陕西革命旧址云上展（十）| 陕甘宁边区政府旧址（121）

63、陕甘宁边区政府旧址管理处开展“五一”节前安全大检查（74）

64、陕甘宁边区政府旧址管理处联合市场沟小学开展“同心并肩铸党魂 传播红色基因 讲好延安故事”教育活动启动仪式（62）

65、【1077最关注】陕甘宁边区政府交际处旧址保护修缮工作昨日正式启动（44）

66、陕甘宁边区政府交际处旧址 保护修缮工程开工仪式4月6日举行（356）

67、陕甘宁边区政府交际处旧址保护修缮工作启动（690）

68、每日一景 | 你好！陕甘宁边区政府旧址（78）

69、红旗漫卷 || 陕西革命旧址云上展之十一：陕甘宁边区政府参议会礼堂（131）

70、陕甘宁边区政府旧址管理处开展春季消防知识讲座（65）

71、红旗漫卷 || 陕西革命旧址云上展之十：陕甘宁边区政府旧（32）

72、延安之旅——陕甘宁边区政府旧址（210）

73、红旗漫卷——陕西革命旧址云上展（陕甘宁边区政府旧址）（23）

74、陕甘宁边区政府旧址管理处开展节前安全大检查（69）

75、陕甘宁边区政府旧址管理处党支部召开2020年度党支部书记抓党建工作述职评议会（52）

76、陕甘宁边区政府旧址管理处召开延安时期统计史略展座谈会（103）

77、陕甘宁边区政府旧址管理处传达局党委第八次中心组学习精神（28）

78、市科技局赴陕甘宁边区政府旧址 开展“倡廉洁、践使命”主题党日活动（41）

79、延安市科技局赴陕甘宁边区政府旧址开展“倡廉洁、践使命”主题党日活动（107）

80、【四史宣讲】肝胆相照，贻范古今——走进陕甘宁边区政府旧址（31）

81、坚守初心使命、传承红色基因︱区机关事业养老保险经办中心赴陕甘宁边区政府旧址开展主题党日活动（10）

82、陕甘宁边区政府旧址管理处开展”文物珍藏记忆·见证抗疫历程暨消除安全隐患 、筑牢安全防线”主题活动（47）

83、陕甘宁边区政府保安处旧址革命纪念馆建设座谈会召开（757）

84、【本地要闻】陕甘宁边区政府保安处旧址革命纪念馆建设座谈会召开（167）

85、陕甘宁边区政府旧址管理处即日起对外开放（27）

86、82年前的今天陕甘宁边区政府成立（36）

1. 陕甘宁边区政府旧址从今日起闭馆修缮（38）
2. 游玩的朋友注意了！陕甘宁边区政府旧址从今天起闭馆修缮（1）
3. 游玩的朋友注意了！陕甘宁边区政府旧址从明天起闭馆修缮（125）
4. 弘扬延安精神 传承红色基因 筑梦新时代 陕甘宁边区政府旧址管理处红色文化进校园活动（39）
5. 延安城南：参观陕甘宁边区政府旧址（213）
6. 榆林红色遗址——陕甘宁边区政府临时驻地旧址（203）
7. 陕甘宁边区政府旧址管理处举行文物捐赠仪式（196）
8. 陕甘宁边区政府旧址管理处开展春季消防安全知识讲座及演练活动（69）
9. 陕甘宁边区政府旧址管理处组织党员及部分骨干职工学习 《中国共产党支部工作条例（试行）》（55）
10. 红色路线」延安五大红色旅游景点之一——陕甘宁边区政府旧址（155
11. 延安12345 | 陕甘宁边区政府旧址管理处关于征集文物的公告（157）
12. 革命旧址调研 | 陕甘宁边区政府保安处旧址（82）
13. 陕甘宁边区政府旧址管理处关于文物征集的公告（209）
14. 陕甘宁边区政府旧址管理处隆重举行边区政府成立81周年纪念活动（132）
15. 不忘初心：历史上的今天——陕甘宁边区政府成立（47）
16. 陕甘宁边区政府旧址管理处党支部启动“113+445”党建工作模式（22）
17. 为了搞好党建，陕甘宁边区政府旧址启动“113+445”工作模式！（227）
18. 榆林红色遗址——陕甘宁边区政府机关驻地旧址（219）
19. 参观陕甘宁边区政府旧址（60）
20. 讲边区历史故事 品先辈革命情怀—陕甘宁边区政府旧址“历史讲堂”活动（108）
21. 三下乡|忆往昔，思初心——陕甘宁边区政府旧址、延安新闻纪念馆（256）
22. 不忘初心 砥砺前行——鲁艺文化园区党日活动走进新闻纪念馆和陕甘宁边区政府旧址（218）
23. "岁月回音壁"--陕甘宁边区政府旧址庆“五· 一”活动（55）
24. 带您走进陕甘宁边区政府旧址（78）
25. 陕甘宁边区政府旧址管理处开局之年踏上新征程（97）
26. 陕甘宁边区政府旧址管理处组织召开民主生活会（79）
27. 陕甘宁边区政府旧址学习贯彻市委五届四次全会精神（55）
28. 让我们携梦前行---陕甘宁边区政府旧址元旦献辞（133）
29. 陕甘宁边区政府旧址管理处举行冬季消防灭火演练（50）
30. 陕甘宁边区政府旧址管理处举行“认识火灾，学会逃生”专题讲座（78）
31. 延安大礼堂（陕甘宁边区政府旧址）开展“五 · 一”安全检查工作（67）
32. VR带您走进陕甘宁边区政府旧址（34）
33. **视频：57**

**中共中央西北局革命旧址**

1. 西北局革命旧址管理处召开“加强文物保护 坚定文化自信”专题学习会议（9）
2. 延安市省级党史教育基地座谈会召开（160）
3. 【清廉机关创建专刊】（七）新一届中央领导集体赴延安，宣示了什么？（79）
4. 铭记西北局历史 缅怀革命先烈（人物篇 第四十七期）（34）
5. 西北局革命旧址管理处召开解读党的二十大报告专题学习会议（40）
6. 时政新闻眼丨新一届中央领导集体赴延安，宣示了什么？（96）
7. 【组工讲堂】新一届中央领导集体赴延安，宣示了什么？（197）
8. 在西北局的日子里——百年大党的精神密码（第六十七期）（41）
9. 铭记西北局历史 缅怀革命先烈 （人物篇 第四十六期）（34）
10. 【陕西宝藏】跟着总书记看看陕西的红色宝藏（193）
11. 新一届中央领导集体赴延安，宣示了什么？（1.1万）
12. 陕西延安革命文物国家文物保护利用示范区建设加快（26）
13. 220家文博单位入选八部委实践教学基地名单！（1153）
14. “云游”红色展馆-走进中共中央西北局革命旧址（21）
15. 红色展馆推介|中共中央西北局纪念馆（12）
16. 中共中央西北局纪念馆组织开展讲解组业务考核“大练兵”（212）
17. 中共中央西北局纪念馆（19）
18. 红色故事 | 延安革命遗址（154）
19. 5·18国际博物馆日 中共中央西北局纪念馆走进校园（95）
20. 中共中央西北局纪念馆组织2022年反恐防暴综合演练（60）
21. 绥德红色革命旧址---中共中央西北局驻地旧址（14）
22. 中共中央西北局旧址（9）
23. 青年 | 红色题材 匠心力作——大型寿山石雕《中共中央西北局旧址》问世（701）
24. 中共中央西北局纪念馆2021年讲解员等级评定考核（755）
25. 中共中央西北局旧址：传承革命精神 赓续红色血脉（50）
26. Day 2 其二 中共中央西北局旧址（16）
27. 【暑期三下乡（二十七）】参观西南旧址，学习红色精神（90）
28. 解码中共中央西北局革命旧址红色魅力（73）
29. 转载自《榆林红色遗址——中共中央西北局驻地旧址》（41）
30. 中共中央西北局设立的调查研究机构（1550）
31. 他们为什么能把党史故事讲得直抵人心？（3267）
32. 践行使命讲好红色故事 赓续奋斗献礼建党百年（7741）
33. 【党史一起学】陕西革命旧址云上展：中共中央西北局旧址（43）
34. 中共中央西北局：峥嵘岁月八十载 革命精神历久弥新（910）
35. 【一起学党史】1941年5月13日 中共中央西北局成立（84）
36. 中共中央西北局纪念馆征集文物（275）
37. 党史学习||经管学院学生党支部组织第58期积极分子开展“学党史 明担当 再奋进”实践教学活动（三（225）
38. 他们走进了西北局革命旧址，从党史里寻找初心！（603）
39. 陕西革命旧址云上展（七）| 中共中央西北局旧址（52）
40. 【解德辉“行走吕梁”游记⑩】中共中央西北局旧址参观记（423）
41. 【党史学习】重游初心之地①——陕西革命旧址：中共中央西北局旧址（287）
42. 党史学习|红色印迹：陕西篇（七）中共中央西北局旧址（28）
43. 博物馆之旅——中共中央西北局革命纪念馆（47）
44. 中共中央西北局旧址和凤凰山麓革命旧址入选国务院第三批国家级抗战纪念设施、遗址名录（12）
45. 第三批国家级抗战纪念设施、遗址名录公布，请收藏！（179）
46. 国务院公布第三批国家级抗战纪念设施、遗址名录（4.1万）
47. 第三批国家级抗战纪念设施、遗址名录（35）
48. 国务院关于公布第三批国家级抗战纪念设施、遗址名录的通知（10万+）
49. 走进中共中央西北局纪念馆，追忆革命志士峥嵘岁月（80）
50. 中共中央西北局旧址（40）
51. 中共中央西北局旧址景区开展春节前安全大检查（86）
52. 【关注】中央统战部组织45位党外干部宝塔山下上“党课”！（5977）
53. 抗日战争研究︱黄道炫：1944年西北局关于群众组织的讨论（193）
54. 2019新版中国红色旅游景区名录，太实用了！（861）
55. 我们，与社会同行 | E路先锋 E路领航——承革命初心，启时代征程（151）
56. 【聚焦5A级景区建设】中共中央西北局革命旧址精细化服务获点赞（156）
57. 榆林红色革命教育基地巡礼丨中共中央西北局及义合会议旧址（6）
58. 太珍贵了！西北局革命纪念馆获赠“贺龙用过的手枪”（399）
59. 参观西北局旧址，瞻仰革命先烈（125）
60. 中共中央西北局纪念馆 志愿者及“小小讲解员”招募啦（1189）
61. 中共中央西北局纪念馆宣教部2018年度工作总结（705）
62. VR全景游览 · 中共中央西北局旧址（60）
63. 枣园、杨家岭、中共中央西北局革命旧址数字博物馆上线，圣地延安数字博物馆群初具规模！（73）
64. 枣园、杨家岭、中共中央西北局革命旧址数字博物馆正式上线（175）
65. 枣园、杨家岭、中共中央西北局革命旧址数字博物馆上线啦（130）
66. 感受红色革命精神，可别忘了中共中央西北局旧址（12）
67. 【红色延安】现场教学之中共中央西北局革命旧址（24）
68. 党委中心组学习走进西北局旧址（102）
69. 【媒体关注】走进中共中央西北局旧址 追忆革命志士峥嵘岁月（65）
70. 两位政治局委员出京首站均赴同一地（4.4万）
71. 图解:全国红色旅游经典景区三期总体建设方案（335）
72. 《全国红色旅游经典景区三期总体建设方案》印发 | 附图解（6746）
73. 红色圣地——中共中央西北局革命旧址（43）
74. 创建国家5A级旅游景区（二）中共中央西北局革命旧址（38）
75. 文博新闻(语音版)2017.4.19（895）
76. 【讲革命故事 忆峥嵘岁月】西北局旧址看点多（692）
77. VR带您走进陕西 | 中共中央西北局旧址（9）
78. 西北革命历史网网站工作人员赴中共中央西北局旧址及其纪念馆参观学习（840）
79. 中共中央西北局纪念馆简介（83）
80. 中共中央西北局革命纪念馆（353）
81. 你好！我叫延安，这是我的简历……（4512）
82. 邦奇电子承接中共中央西北局革命纪念馆项目（2）
83. **视频：92**

**南泥湾革命旧址**

1. 《延安<解放日报>上的南泥湾》第十四期丨 延安南泥湾革命旧址深入开展“奋进新征程，谱写新篇章”主题活动（32）
2. 延安南泥湾革命旧址管理处举办学习贯彻党的二十大精神专题宣讲会（69）
3. 延安南泥湾革命旧址管理处举行反恐防暴应急演练（48）
4. 延安南泥湾革命旧址管理处召开党的二十大会议精神第二次集体学习会议（95）
5. 延安南泥湾革命旧址第三十四次工作例会（97）
6. 延安南泥湾革命旧址管理处第三十二次工作例会（79）
7. 打卡廉政教育基地 | 南泥湾革命旧址（22）
8. 【党史知识】陕西革命旧址云上展：南泥湾革命旧址（503）
9. 延安南泥湾革命旧址管理处召开第三十次工作例会（129）
10. 延安南泥湾革命旧址管理处召开第二十七次工作例会（139）
11. “云游”红色展厅—走进南泥湾革命旧址（14）
12. 游遍陕西——延安地区（418）：南泥湾革命旧址（19）
13. 延安南泥湾革命旧址管理处召开2022年目标任务半年工作推进会（313）
14. 延安南泥湾革命旧址管理处有关规范参观的通告（688）
15. 延安南泥湾革命旧址管理处开展第二季度讲解员业务考核工作（433）
16. 红色展馆推介|南泥湾革命旧址（16）
17. 延安南泥湾革命旧址管理处第十七次周例会（129）
18. 延安南泥湾革命旧址管理处 “五一” 参观须知（233）
19. 延安南泥湾革命旧址管理处开展“五一”节前安全生产大检查（236）
20. 延安南泥湾革命旧址管理处第十六次周例会（144）
21. 铭记嘱托 忠诚担当|临镇中队到南泥湾革命旧址开展学习实践活动（100）
22. 延安南泥湾革命旧址管理处 第十五次周例会（64）
23. 延安南泥湾革命旧址管理处召开第十次工作例会（68）
24. 延安南泥湾革命旧址管理处召开第七次工作例会（313）
25. 南泥湾革命旧址管理处开展“猜灯谜、庆元宵”主题活动（88）
26. 南泥湾革命旧址管理处——志愿有我，“疫”不 容辞。（265）
27. 每日名胜 | 陕西·南泥湾革命旧址（6）
28. 延安南泥湾革命旧址管理处 “12.4”全国法治宣传日主题活动（148）
29. 延安南泥湾革命旧址管理处 传达学习十九届六中全会精神（125）
30. 延安南泥湾革命旧址管理处2021年讲解员等级评定考核（448）
31. 党史展馆巡展I南泥湾革命旧址（14）
32. 延安南泥湾革命旧址管理处召开新冠疫情防控紧急会议（274）
33. 延安南泥湾革命旧址管理处疫情防控公告（597）
34. 【红色堡垒·百年党史】红色地图 | 陕西•南泥湾革命旧址（43）
35. 延安南泥湾革命旧址管理处 召开近期工作安排部署会议（214）
36. 延安十大旅游景点-南泥湾革命旧址（361）
37. 延安南泥湾革命旧址管理处安排部署“十一”假期安全工作（143）
38. 循党史 访红迹(第23期) | 南泥湾革命旧址（13）
39. 党史学习|红色印迹：南泥湾革命旧址（9）
40. 【追寻“红色印迹”】陕西·南泥湾革命旧址（5）
41. ​延安南泥湾革命旧址管理处召开疫情防控工作部署会议（338）
42. 延安革命旧址——南泥湾（56）
43. 延安南泥湾革命旧址管理处召开半年工作推进会（217）
44. 子长市工商联（总商会）组织民营企业人士 赴南泥湾革命旧址开展党史学习现场教学活动（130）
45. 建党百年|红色景点云端游（八十二）南泥湾革命旧址（36）
46. 【党史故事汇】南泥湾精神：永远的传家宝（3176）
47. 《革命圣地延安-南泥湾革命旧址》（93）
48. 延安市延安革命遗址之南泥湾革命旧址（108）
49. 喜迎建党百年 云访红色基地|第十三站：南泥湾革命旧址（78）
50. 百篇陕西红色经典|第七十三篇 南泥湾革命旧址（180）
51. 百年党史日日学 | 陕西革命旧址云上展：南泥湾革命旧址（76）
52. 【党史教育专栏】陕西革命旧址云上展：南泥湾革命旧址（229）
53. 五一国际劳动节南泥湾革命旧址参观须知（79）
54. 延安南泥湾革命旧址管理处 开展“五一”节前安全检查部署（131）
55. 陕西革命旧址云上展（九）|南泥湾革命旧址（141）
56. 安全不懈怠——南泥湾革命旧址开展消防安全培训（134）
57. 百年辉煌•党史小讲堂⑧ | 发扬“南泥湾精神” ，做奋进新时代的拓荒牛（73）
58. 【追寻“红色印迹”】陕西革命旧址云上展：南泥湾革命旧址（6）
59. 南泥湾革命旧址（63）
60. 党史学习|红色印迹：陕西篇（二）南泥湾革命旧址（16）
61. 【党史学习】陕西革命旧址：南泥湾革命旧址（22）
62. 党史百年|初心之地陕西革命旧址：南泥湾革命旧址（196）
63. 百舸争流 更进一步丨南泥湾革命旧址讲解员第一季度考核（187）
64. 南泥湾革命旧址管理处召开艺术品小样评审会（172）
65. 每日一景 | 你好！南泥湾革命旧址（147）
66. 红旗漫卷——陕西革命旧址云上展(南泥湾革命旧址)（50）
67. 红旗漫卷 || 陕西革命旧址云上展之九：南泥湾革命旧址（97）
68. 延安南泥湾革命旧址管理处召开工作推进会（281）
69. 延安革命纪念地管理局局长兰爱平调研指导南泥湾革命旧址工作（720）
70. 这里是落脚点，也是出发点（1.3万）
71. 延安南泥湾革命旧址管理处 召开春节后工作安排部署会（250）
72. 延安南泥湾革命旧址管理处开展节前消防安全检查及防疫部署（204）
73. 南泥湾革命旧址管理处开展了“无联不成春，有联春更浓”活动（239）
74. 南泥湾革命旧址管理处组织召开2021年“春节”节前安排部署会（285）
75. 南泥湾革命旧址传达学习延安革命纪念地管理局党委中心组会议精神（206）
76. 分享一篇文章。（25）
77. 南泥湾革命旧址开展中秋、国庆节前安全大检查（188）
78. 【基层党建】我校生科院教工党支部赴南泥湾革命旧址开展延安精神教育主题党日活动（280）
79. 延安南泥湾革命旧址管理处开展健康知识讲座（284）
80. 延安南泥湾革命旧址管理处召开创文工作安排部署会议（286）
81. 南泥湾革命旧址管理处召开2020年半年工作推进会（264）
82. 市房产信息中心赴南泥湾革命旧址开展庆祝建党99周年主题党日活动（20）
83. 全国红色旅游经典景区—南泥湾革命旧址（119）
84. 后勤党支部在南泥湾革命旧址开展“学用新思想、奋进新时代”主题党日活动（181）
85. 宜川县城市管理执法局党支部组织党员干部赴南泥湾革命旧址开展“弘扬延安精神 传承红色基因”庆“七一”活动（1324）
86. 市建筑行业统筹办党支部赴南泥湾革命旧址开展固定党日活动（32）
87. 南泥湾革命旧址“五一”假期不停歇，各项工作有序开展。（247）
88. 奋斗路上永不停歇---延安南泥湾革命旧址“五四青年节”（352）
89. 通知一一延安南泥湾革命旧址管理处 关于文物藏品资料征集公告（1069）
90. 南泥湾革命旧址管理处恢复对外开放公告（477）
91. 延安南泥湾革命旧址管理处开展消防安全检查与疫情防护工作（222）
92. 薛耀军检查局系统疫情防控工作调研南泥湾革命旧址陈列布展情况（740）
93. 南泥湾革命旧址管理处推进疫情防护工作（416）
94. 延安南泥湾革命旧址管理处临时展馆关于闭馆的通知（529）
95. 动态丨南泥湾革命旧址管理处开展消防安全大检查（227）
96. “不忘初心 、牢记使命”弘扬延安精神 —市国资委赴南泥湾革命旧址参观学习（113）
97. 想当个红色志愿者吗？南泥湾革命旧址向您发出邀请了！（92）
98. 延安南泥湾革命旧址管理处 关于招募社会志愿者的公告（319）
99. 安沟镇党委在南泥湾革命旧址开展“不忘初心、牢记使命”现场教学活动（1168）
100. 相约延安 祝福祖国。南泥湾革命旧址红歌快闪激情上演（919）
101. 昨天，红歌快闪在南泥湾革命旧址激情上演！（361）
102. 活动丨延安南泥湾革命旧址管理处开展十一节前安全大检查（226）
103. 延安 南泥湾革命旧址（48）
104. 活动｜南泥湾革命旧址管理处开展打击防范文物犯罪宣传活动（189）
105. 参观南泥湾革命旧址 重温延安精神（150）
106. 真棒！南泥湾革命旧址来了“小小讲解员”（104）
107. 活动｜南泥湾革命旧址管理处开展“小小讲解员社会实践活动”（319）
108. 动态｜南泥湾革命旧址开展安全大检查（237）
109. 快闪|延安南泥湾革命旧址举行“传红色精神，唱爱国歌曲”系列主题活动（352）
110. 会议｜南泥湾革命旧址召开2019年纪律工作会议（256）
111. 动态｜南泥湾革命旧址召开2019年宣教工作部署会议（139）
112. 动态｜南泥湾革命旧址召开扫黑除恶专项斗争专题会议（307）
113. 动态｜延安南泥湾革命旧址管理处春节前开展安全大检查（362）
114. 总结｜延安南泥湾革命旧址管理处2018年上半年工作总结（485）
115. 2018陕西延安市南泥湾革命旧址管理处招聘公告（434）
116. 好消息！南泥湾革命旧址管理处招人啦（286）
117. 延安南泥湾革命旧址管理处2018春节安全大排查（291）
118. 延安南泥湾革命旧址管理处文物征集启事（634）
119. 延安南泥湾革命旧址管理处冬季消防演练活动（33）
120. 延安南泥湾革命旧址管理处举办“红色教育进课堂”主题教育活动（59）
121. 延安南泥湾革命旧址雷锋月活动之 --关爱老人让社会充满爱！（3）
122. 杜芳华局长一行调研南泥湾革命旧址（60）
123. 南泥湾革命旧址 “送万福进万家义写春联”活动（13）
124. 南泥湾革命旧址管理处举行消防演练活动（44）
125. 南泥湾革命旧址管理处—消防演练活动（4）
126. 南泥湾革命旧址—长征故事进校园活动（3）
127. 南泥湾革命旧址迎来千余知青（124）
128. 创卫快讯——南泥湾革命旧址卫生自查工作（98）
129. 南泥湾革命旧址举行“庆七一”活动（55）
130. 南泥湾革命旧址消防安全演练（106）
131. 6.11日南泥湾革命旧址管理处组织开展”让文化遗产融入现代生活“的主题宣传活动（199）
132. 南泥湾革命旧址快讯（110）
133. 南泥湾革命旧址“2+1”三城联创工作动态（35）
134. 南泥湾革命旧址关于"2+1"三城联创快讯（29）
135. 延安南泥湾革命旧址被冲垮 毛泽东旧居遭雨水浸泡（1）
136. **视频：134**

**桥儿沟革命旧址**

1. 【党史天天读】党史上的11月8日（25）
2. 【汉中蜀道石刻艺术博物馆】党史知识:陕西革命旧址云上展“桥儿沟革命旧址”！（9）
3. 弘扬延安精神，团结奋斗交出新的优异答卷——习近平总书记在瞻仰延安革命纪念地时的重要讲话在我市引发热烈反响（226）
4. 【党史知识】陕西革命旧址云上展：桥儿沟革命旧址（118）
5. 重走长征路 || 感悟红色长征路，延安精神青年说（一（2072）
6. 三下乡 | 历史文化学院举行“赓续红色精神—调研延安革命旧址保护情况”实践活动（一）（238）
7. 游遍陕西——延安地区（416）：桥儿沟革命旧址（19）
8. “小故事”里的“大情怀”：你所不知道的延安文艺座谈会（4）
9. 西北局革命旧址、中国医科大学旧址被授予“陕西省爱国主义教育基地”（110）
10. 中国医科大学旧址 陕西省爱国主义教育基地揭牌仪式举行（337）
11. 学史知来•时代觉醒 | 桥儿沟教堂缘何著名？（一百八十四）（17）
12. 青年大学习第十二季第七期来啦！（825）
13. 【团团课堂】中国共产党历史上的今天——11月8日（31）
14. 每日党史 | 2021年11月8日（151）
15. 党史天天学 | 2021年11月8日（2378）
16. 2021年11月7日 | “六中全会是决定中国之命运的”（2784）
17. 毛泽东思想的指导地位是怎么确立的？（2165）
18. 国庆假期红色旅游持续火热！这些红色“打卡地”你都去过吗？（1399）
19. 延安“红色之旅”来了，一起去看宝塔山！（5591）
20. 【云展厅】线上纪念馆第五十六站——延安桥儿沟革命旧址（13）
21. 延安，革命文物保护管理利用工作这样做（1413）
22. 三下乡 | 西安财经大学党史学习教育实践活动（1529）
23. 三下乡 | 红色传家宝（四）：延安新闻纪念馆（173）
24. 中央党校旧址陈列布展工作汇报会在京召开（545）
25. 照亮前路的真理光芒——中国共产党成立100周年启示录之“求索篇”（6355）
26. 奋斗百年路 启航新征程·启示录丨照亮前路的真理光芒——中国共产党成立100周年启示录之“求索篇”（59）
27. 党史回眸 | “决定中国之命运”的六届六中全会（2174）
28. ​陕西延安桥儿沟革命旧址：用文艺吹响民族解放的号角（9）
29. 【党史百年】桥儿沟教堂缘何著名？党的六届六中全会里有回答（26）
30. 党史百年|初心之地陕西革命旧址云上展：桥儿沟革命旧址 （82）
31. 【画说党史】延安桥儿沟革命旧址（64）
32. 陕西百篇红色经典|第七十篇 桥儿沟“鲁艺”旧址（183）
33. 从机场出发，打卡红色旅游航线（474）
34. 【峥嵘岁月】党的七大——汇聚从胜利走向更大胜利的力量（2353）
35. 【奋斗百年路 启航新征程】“六中全会是决定中国之命运的”（175）
36. 法治文苑 ▏“六中全会是决定中国之命运的”（43）
37. “六中全会是决定中国之命运的”（4.8万）
38. 百年党史日日学 | 陕西革命旧址云上展：桥儿沟革命旧址（100）
39. 【党史学习】重游初心之地⑦——陕西革命旧址：桥儿沟革命旧址（133）
40. 蓝天幼儿园走进延安线上主题教育活动——云观革命旧址之“桥儿沟革命旧址”（501）
41. 百年党史天天学（485）
42. 【学党史】党史微课 | “决定中国之命运”的六届六中全会⑬（25）
43. 党史学习|红色印迹：陕西篇（十）桥儿沟革命旧址（10）
44. 党史微课 | “决定中国之命运”的六届六中全会⑬（3301）
45. 陕西革命旧址云上展（二）| 桥儿沟革命旧址（60）
46. 红色精神引领民族复兴（2237）
47. 陕西延安举办“鲁艺故事进校园”活动，推行美育教学（175）
48. 延安精神永放光芒（708）
49. 红旗漫卷——陕西革命旧址云上展（二）（390）
50. 音乐欣赏《圣地记忆》（456）
51. 红旗漫卷｜陕西革命旧址云上展——王家坪革命旧址（395）
52. 红旗漫卷 | 陕西革命旧址云上展——杨家岭革命旧址（314）
53. “决定中国之命运的”中共六届六中全会（1087）
54. 红旗漫卷|陕西革命旧址云上展——桥儿沟革命旧址（147）
55. 解释中心党支部在桥儿沟革命旧址开展主题党日活动（118）
56. 延安鲁艺 邀请您开启十一文艺圣地云游之旅（906）
57. 延安市扶贫开发局走进桥儿沟革命旧址开展“庆祝建国71周年”主题党日活动（289）
58. 桥儿沟革命旧址举行长安大学暑期社会实践基地挂牌仪式（353）
59. 红色遗产专题 | 延安革命旧址群整体性保护实践的探索（846）
60. 延安桥儿沟革命旧址 陕西省爱国主义教育基地揭牌仪式举行（669）
61. 2020年度“5·18国际博物馆日” 鲁艺文化园区举办《红色桥儿沟》展览（1124）
62. 西安6处！陕西命名17处爱国主义教育基地（670）
63. 陕西命名17处爱国主义教育基地（391）
64. 榆林3处！陕西新命名17处爱国主义教育基地（4947）
65. 眉县张载祠、红光沟航天六院旧址被命名为陕西省爱国主义教育基地（922）
66. 坚守初心使命 陕西文博人的文物保护利用之路（2042）
67. 延安这些旧址，你去过哪些？（1373）
68. 全国革命文物保护利用十佳案例全放（805）
69. “桥儿沟革命旧址东、西山旧址保护维修工程”获全国革命文物保护利用十佳案例（100）
70. 【聚焦】全国革命文物保护利用十佳案例在京发布！信阳榜上有名！（161）
71. 【政•聚焦】信阳鄂豫皖革命纪念馆上榜这项在京发布的名单（688）
72. 全国革命文物保护利用十佳案例在京发布（1766）
73. 【红色经典永流传】第六期：才学报国陈康白（598）
74. 赣州这个博物馆，火了！（2564）
75. 江西这两个博物馆，火了！（3956）
76. 我与祖国共成长·历史钩沉 | 从延安走来（1918）
77. 来吧，在延安留下你独特的国庆记忆！（3417）
78. 延安为什么非去不可？看完这些你就懂了（4515）
79. 回望历史 再谱新篇——“我们心中的鲁艺”座谈会在延安桥儿沟革命旧址举行（223）
80. 延安桥儿沟革命纪念馆游览之感悟（1009）
81. 市口岸办开展固定党日活动——党员干部走进桥儿沟革命旧址（21）
82. 【工作动态】民进延安市委讲师团走进桥儿沟革命旧址开展送课活动（17）
83. 民进延安市委讲师团走进桥儿沟革命旧址开展送课活动（57）
84. 民进延安市讲师团 走进桥儿沟革命旧址送课活动（76）
85. 延安桥儿沟革命旧址管理处文物征集函（875）
86. 干啥呢？桥儿沟鲁艺旧址这么红火热闹！（908）
87. 五一小长假，来延安你必须知道的事！（3752）
88. 桥儿沟革命旧址招募“小小讲解员”公告（349）
89. 重温革命历史，传承红色基因：中央戏剧学院赴延安参加纪念鲁迅艺术学院成立80周年系列活动（2530）
90. “鲁艺”80年：穿越时空，追忆那段“光辉岁月”（808）
91. 2017，我们这一年……（1750）
92. 桥儿沟旧址防汛安全警钟长鸣（216）
93. 延安革命纪念地管理局系统 纪念建党96周年主题党日活动走进桥儿沟旧址（409）
94. 刘炽馆方案讨论会在桥儿沟旧址举行（111）
95. 六届六中全会陈列方案评审会在桥儿沟革命旧址举行（58）
96. 六届六中全会陈列方案第四次评审会 在桥儿沟革命旧址举行（74）
97. 延安计划将5个景区捆绑创建国家5A级景区，看看有哪些……（1304）
98. 红色地标—红色景区中国万里行—延安桥儿沟革命旧址直击现场（54）
99. **视频：10**

**宝塔山景区**

1. 宝塔山摩崖石刻——历史的回音墙（148）
2. “延安有宝塔，巍巍高山上。” 走进红色延安，打卡宝塔山景区（7）
3. 【1077温馨提示】关于宝塔山景区临时关闭的通知80）
4. 关于宝塔山景区临时关闭的通知（4422）
5. 足迹 | 第一期：走进宝塔山景区，寻觅中国共产党人的初心（21）
6. 第二站 宝塔山景区（21）
7. 延安之旅——宝塔山景区（466）
8. 宝塔山景区明日起开放，暂不接待高、中风险地区游客（24）
9. 宝塔山景区开园公告（2839）
10. 新岁添新禧，新年新气象！宝塔山景区给大家拜年啦（753）
11. 受雨雪天气影响，宝塔山景区临时关闭（41）
12. 宝塔山景区恢复参观了！游客需提供核酸检测阴性证明（14）
13. 宝塔山景区恢复参观，暂不接待疫情中高风险地区游客（51）
14. 受冰雪天气影响 延安宝塔山景区临时关闭（211）
15. 【HVAC】红色文化建筑（28）：延安市宝塔山景区保护提升项目暖通空调设计（483）
16. 秋游宝塔山（145）
17. 宝塔山景区开展讲解员第三季度考核工作（276）
18. 国庆节期间，宝塔山景区实行限量、预约、错峰入园（350）
19. 延安：宝塔山景区发布公告！（46）
20. 宝塔山景区中秋国庆双节“限量、预约、错峰”公告（442）
21. @延安人 今日起延安宝塔山景区开放（475）
22. 因暴雨天气，宝塔山景区临时关闭！（162）
23. 宝塔山景区全力以赴做好疫情防控工作（124）
24. 宝塔山景区介绍（243）
25. 转发周知！今起宝塔山景区暂时闭园，明日恢复开放（208）
26. 关于宝塔山景区网络系统检修闭园的公告（25）
27. 民生关注 | 关于宝塔山景区网络系统检修闭园的公告（35）
28. 最新！宝塔山景区网络系统检修明起闭园 7日恢复开放（314）
29. 【我为群众办实事】一大队二中队到宝塔山景区开展道路交通安全宣传（170）
30. 这场在宝塔山的直播吸引了201万网友在线观看（898）
31. 圣地延安，宝塔山下见初心（3751）
32. 宝塔山景区：唱响红色歌曲 诵读红色诗词（105）
33. “五一假期”第二天 宝塔山景区游人如织 秩序井然（655）
34. 6号未来酒店 | 宝塔山景区旅游，请看过来（407）
35. 重要通知：今天起，宝塔山景区恢复门票收费！（7212）
36. 宝塔山景区恢复正常收费的公告（114）
37. 【1077温馨提示】宝塔山景区从4月1日零时起恢复门票收费（63）
38. 通知|宝塔山景区从4月1日零时起恢复门票收费（90）
39. 延安：关于宝塔山景区恢复正常收费的公告（526）
40. 【1077最关注】关于宝塔山景区恢复正常收费的公告（26）
41. 延安宝塔山景区4月1日起恢复门票收费！（3007）
42. 重要通知 | 自2021年4月1日起，宝塔山景区恢复门票收费！（164）
43. 关于宝塔山景区恢复正常收费的公告（1.6万）
44. 宝塔山景区（82）
45. 郭周成：宝塔山景区的“美容师”（486）
46. 宝塔山景区疫情防控和安全生产大排查（304）
47. 通知！雨雪天气保障游客安全，延安宝塔山景区临时关闭（94）
48. 宝塔山景区开放通知（151）
49. 宝塔山景区滑坡抢修通知（603）
50. 走进宝塔山景区工作人员的假日时光！（539）
51. 宝塔山景区节前安全大检查（134）
52. 宝塔山景区在延安市首届金牌讲解员大赛中荣获佳绩（304）
53. 今起，宝塔山景区正式成为省级爱国主义教育基地！（1346）
54. 宝塔山景区接待演练大练兵开赛（682）
55. “五一”旅游，就来延安宝塔山景区（631）
56. 宝塔山景区“五一”假期游览温馨提示（1684）
57. 宝塔山景区给市民朋友们送福利了！（5022）
58. 宝塔山景区疫情防控宣传漫画（207）
59. 为打赢疫情狙击战，宝塔山景区做了不少事儿！（170）
60. 宝塔山景区临时关闭通知（101）
61. 好消息！宝塔山景区免费了，有想去的安排上（23）
62. @延安人~好消息！宝塔山景区元旦起免费了，有想去的安排上（8）
63. 宝塔山景区现阶段免费开放啦（350）
64. 好消息！宝塔山景区元旦起免费了，有想去的安排上（4139）
65. 宝塔山景区近期工作安排部署（76）
66. 延安革命纪念地管理局领导深入宝塔山景区调研（55）
67. 宝塔山景区展开文物自查自纠第一阶段工作（69）
68. 宝塔山景区宣教团队学习观摩系列活动——延安北京知青博物馆（32）
69. 新旧建筑融合共生 - 延安宝塔山游客中心（2636）
70. 宝塔山景区附近怡然阁（苹果嘉儿）大床房（4）
71. “相约延安，祝福祖国”——宝塔山景区在这里等您的到来！（51）
72. 宝塔山景区荣获“2019 陕西最美秋色”称号（690）
73. 向辛勤的园丁致敬，宝塔山景区献礼教师节！（1759）
74. 庄惟敏：延安地域建筑文化的传承与创新——宝塔山游客中心暨宝塔山景区保护提升工程（1686）
75. 宝塔山景区讲解路线调整通知（73）
76. 宝塔山景区游客服务中心商铺招商公告（194）
77. 宝塔山森林公园提升美化亮化 成为市民休闲好去处（675）
78. 延安宝塔山游客中心暨宝塔山景区保护提升工程荣膺UIA(国际建筑师协会)2019年第四届巴库国际建筑大奖（9808）
79. 宝塔山景区积极落实夏季防汛工作（31）
80. 宝塔山景区祝1031万考生一举高“粽”！！！（487）
81. 【魅力延安】宝塔山景区升级改造 游客中心全新亮相 景点内容更加丰富（157）
82. 宝塔山景区升级改造 游客中心全新亮相 景点内容更加丰富（2511）
83. 正能量！宝塔山景区好人好事蔚然成风，受游客点赞！（6）
84. 宝塔山景区人气旺 游客秩序井然（67
85. 5月2日宝塔山景区迎来“五一”小长假高峰（46）
86. 延安12345 | 新的起点、新的征程、宝塔山景区游客中心正式启用（508）
87. 新的起点、新的征程、宝塔山景区游客中心正式启用（1739）
88. 延安宝塔山景区又有新变化啦！（1394）
89. 注意！宝塔山景区出入口及游览线路调整啦（1319）
90. 好嗨哟， 宝塔山景区感觉不一样噢！（281）
91. 宝塔山景区保护提升工程内部装修已基本完工（10）
92. 宝塔山景区保护提升工程内部装修已基本完工 五一将正式对外开放（19）
93. 宝塔山景区内部装修已基本完工 五一将正式对外开放（436）
94. 宝塔山景区保护提升工程要完工了 五一小长假等你来！（244）
95. 宝塔山景区商铺招商火爆进行中，你心动了吗？（782）
96. 号外！号外！宝塔山景区商铺招商火爆进行中!（1559）
97. 宝塔山景区狠抓安全生产 严密部署清明节工作（413）
98. 宝塔山景区节前安全检查（174）
99. 全市导游技能教育培训现场教学在枣园革命旧址、宝塔山景区开展（83）
100. 宝塔山景区消防暨反恐知识技能培训及演练（144）
101. 宝塔山景区接待礼仪培训（1091）
102. 宝塔山景区创文及创5A初评阶段工作推进会（465）
103. 宝塔山景区迎来自驾游高峰（31）
104. 迎国庆、宝塔山景区欢迎您！！！（338）
105. 宝塔山景区召开2018年国庆节工作安排部署会（328）
106. 电影《周恩来回延安》剧组在宝塔山景区拍摄（41）
107. 秋分与中秋相遇！宝塔山景区邀您相约！！（135）
108. 有福利丨从今起持人民警察证可免费参观宝塔山等景区（5118）
109. 【陕西名胜】宝塔山（595）
110. 【期待】宝塔山景区保护提升工程 今年10月份建成投用（1023）
111. 宝塔山景区：来延安 追寻初心（1818）
112. 宝塔山景区清明节前安排部署（199）
113. 宝塔山景区环境整治房屋征收工作圆满完成（196）
114. AAAAA金山风景区 宝塔山风景区一日游 99元 4月6日发班（1060）
115. 宝塔山景区获得延安市旅游营销突出贡献奖（122）
116. 宝塔山景区召开创建全国文明城市迎检工作动员会（255）
117. AAAAA金山风景区 宝塔山风景区一日游 99元（67）
118. 宝塔山旅游景区“红歌快闪” 欢庆十九大胜利闭幕（56）
119. 文明旅游，宝塔山景区志愿者在行动（287）
120. 网络购票扫码入园，宝塔山景区欢迎您！（96）
121. 宝塔山景区举办“培育好家风、建设和谐家庭”知识讲座（202）
122. 【陕建·新闻】陕建承建的宝塔山景区城改暨南泥湾红色小镇项目建设启动（800）
123. 宝塔山景区保护提升项目让宝塔山更靓丽（19）
124. 【关注】宝塔山景区保护提升项目让宝塔山更靓丽(927
125. 宝塔山景区暨城改民生项目和南泥湾红色文化小镇项目启动建设（3）
126. 宝塔山景区暨城改民生项目建设 集中启动仪式在宝塔山下举行（575）
127. 红色圣地——宝塔山景区（89）
128. 网曝工作人员“衣冠不整” 宝塔山景区处理两名当事人（261）
129. 延安的旅游景区（景点、演出）之二：宝塔山景区（56）
130. 宝塔山景区举办汉文化公益展演活动（28）
131. 宝塔山景区举行“讲述红色故事 传承延安精神”主题活动（171）
132. 国庆假期，我们在宝塔山景区为祖国母亲保驾护航】（143）
133. 国庆节宝塔山景区临时停车指引（497）
134. 【动态】宝塔山景区同仁来博览园交流考察（60）
135. 为城市添一抹绿——延安市交警一大队宝塔山景区植树（18）
136. 关于规范宝塔山景区晨练活动的通告（2774）
137. 【百姓关注】：宝塔山景区升级进行中摩崖石刻将成开放式景观（277）
138. 宝塔山景区游客和车辆增多（157）
139. **视频：149**

**子长县瓦窑堡会议旧址**

1. 党史学习教育丨探访红都：瓦窑堡会议旧址（116）
2. 百篇陕西红色经典|第六十四篇 瓦窑堡会议旧址（241）
3. 瓦窑堡会议旧址（51）
4. 陕西红色地标（七）——瓦窑堡会议旧址（37）
5. 红色足迹 | 瓦窑堡会议旧址（37）
6. 党史故事接力 |瓦窑堡会议，中国革命焕发生机！（39）
7. 【党史故事】瓦窑堡会议，中国革命焕发生机！（40）
8. 党史丨瓦窑堡会议 | 中国革命焕发生机！（211）
9. 头条丨瓦窑堡会议 中国革命焕发生机！（4）
10. 【热点关注】瓦窑堡会议 | 中国革命焕发生机！（7）
11. 陕西 | 瓦窑堡会议——中国革命焕发生机！（8）
12. 党史故事接力传播| 瓦窑堡会议，中国革命焕发生机！（18）
13. 瓦窑堡会议丨中国革命焕发生机！（19）
14. 【关注】瓦窑堡会议 | 中国革命焕发生机！（789）
15. 【聚焦】瓦窑堡会议 | 中国革命焕发生机！（65）
16. 探访瓦窑堡会议旧址（12）
17. ☀【妇联头条】瓦窑堡会议 | 中国革命焕发生机！（10）
18. 【巾帼心向党】瓦窑堡会议——中国革命焕发生机！（43）
19. 【转发】瓦窑堡会议 | 中国革命焕发生机！（1）
20. 头条 | 瓦窑堡会议——中国革命焕发生机！（921）
21. 致敬！长征！（3836）
22. 【党史知识100讲】抗战篇第二十讲——瓦窑堡会议！（14）
23. “一道道的那个山来哟一道道水”，鸟瞰陕北革命旧址新风貌（667）
24. 建党百年丨建立抗日民族统一战线：瓦窑堡会议（十一）（112）
25. 【巾帼心向党】党史故事接力——瓦窑堡会议 ， 中国革命焕发生机！（40）
26. 头条||党史故事传播:陕西--瓦窑堡会议 | 中国革命焕发生机！（189）
27. 瓦窑堡会议 | 中国革命焕发生机！（1130）
28. 党史 ▏瓦窑堡会议 中国革命焕发生机！（48）
29. 【党史故事】开会啦！开会啦！原来是“瓦窑堡会议”！（143）
30. 党史漫谈 | 党史故事100讲之第十八讲 瓦窑堡会议（49）
31. 巾帼心向党▕ 党史故事接力传播——瓦窑堡会议 中国革命焕发生机！
32. （5）
33. 红都子长瓦窑堡会议（60）
34. 丝路上的一处瑰宝之地，这里不是敦煌但胜似莫高窟，却少有人知晓！（2635）
35. 红色印象 | 子长瓦窑堡会议旧址（91）
36. 【陕西传媒网】瓦窑堡会议 | 中国革命焕发生机！（67）
37. 巾帼心向党丨瓦窑堡会议：中国革命焕发生机！（5）
38. 重走长征路 | 探访瓦窑堡革命旧址（573）
39. 【每日一学】瓦窑堡：“人民共和国”的名字从这里响起（273）
40. 瓦窑堡会议：“一次极关重要的会议”（4万）
41. 瓦窑堡：“人民共和国”的名字从这里响起（914）
42. 子长县在瓦窑堡会议旧址召开纪念中共中央发布“五一口号”70周年座谈会（57）
43. 瓦窑堡会议丨中国共产党的百年足迹（32）（41）
44. “我心中的长征纪念地”评选揭晓 子长县瓦窑堡会议旧址入选（165）
45. 瓦窑堡会议期间，陪伴在领导人身边有哪些不平凡的女性?（828）
46. 党史学习教育专题七十八：瓦窑堡会议（732）
47. 瓦窑堡会议会址 陕西省子长市 第三批全国重点文物保护单位（10）
48. 延安13年•沿着先辈的足迹 (子长篇) 第一集 子长窑洞里的瓦窑堡会议（301）
49. 【党史教育】瓦窑堡会议：“一次极关重要的会议”（545）
50. 寻访瓦窑堡革命旧址：山丹丹花开红艳艳（378）
51. 党的历史上的重要会议之瓦窑堡会议（261）
52. 妇声音 | 中国革命焕发生机！（54）
53. 四史学习第二十期 | 瓦窑堡会议（31）
54. 【奋斗百年路 启航新征程】瓦窑堡会议：“一次极关重要的会议”（121）
55. 【党史学习教育】“百个瞬间说百年”之二十六｜瓦窑堡会议（21）
56. 国庆假期红色旅游持续火热！这些红色“打卡地”你都去过吗？（1399）
57. 再次穿越，给你留下怎样一番光景？（976）
58. 瓦窑堡会议：奏响全面抗战序曲（412）
59. 每日一战：瓦窑决策 联合抗日（4296
60. 【青春心向党】每日党史：瓦窑堡会议（21）
61. 带你打卡革命红都——瓦窑堡（1461）
62. 陕西这份旅游百名榜，西安占9个！“十一”假期正好去看看（2053）
63. 【专栏】红色旅游：八路军西安办事处纪念馆、西安事变纪念馆、瓦窑堡会议旧址（585）
64. 【美文】管朝 · 子长印象（677）
65. 档案馆（校史办）赴瓦窑堡会议旧址 开展十九大精神实践学习活动（648）
66. 【走进陕甘宁红色老区】第三站：安塞腰鼓、红都瓦窑堡（93）
67. 中国共产党实现重大战略转变的瓦窑堡会议（93）
68. 瓦窑堡穿越硝烟待花开（594）
69. 瓦窑堡会议：团结全民族一切革命力量抗日（1073）
70. 【媒体上的子长】瓦窑堡会议：向全中国吹响抗日号角（21）
71. 全国科普日 | 科普去哪儿？来瓦窑堡革命旧址追寻红色记忆~（349）
72. 纪念抗战胜利75周年：铭记历史 砥砺奋进（1.4万）
73. 【历史节点】瓦窑堡会议（5）
74. 抗战主题和红色景点成为我省旅游热门目的地（99）
75. 永远跟党走 | 红色旅游精品景区推荐之延安篇（1667）
76. 瓦窑堡会议 | 纪念日（147）
77. 红船破浪：瓦窑堡革命旧址（5）
78. 《抗战中的延安》（四） 抗日民族统一战线的形成——瓦窑堡会议（300）
79. 瓦窑堡会议：党内政治路线转折 奏响抗日战争序曲（97）
80. 【浴血抗战 历史丰碑】圣地延安 民族希望（166）
81. 基层党建|我校机关党委第五支部全体党员干部赴瓦窑堡会议旧址开展十九大精神宣讲实践活动（159）
82. 【学习百年党史 开启辉煌征程】记瓦窑堡会议（32）
83. 百名女大学生讲述100个党史故事 | 瓦窑堡会议：确定建立抗日民族统一战线（1167）
84. 【县区动态】子长县召开纪念中共中央发布 “五一口号”70周年座谈会（196）
85. 瓦窑堡：奏响全面抗战序曲（2501）
86. 中共子长市委理论学习中心组会议召开（822）
87. “长征路 强国梦”党史大篷车巡展活动走进延安子长（1万）
88. 瓦窑堡会议：解决党的政治路线 奏响全面抗战序曲（404）
89. 党史学习第十八期：瓦窑堡会议（5）
90. “非热门系列”|“十一”出游， 这些地方人少（2013）
91. 【历史今日】瓦窑堡会议召开（11）
92. 【 百年党史】之：瓦窑堡会议（64）
93. 9月小长假去哪里？ 延安15处红色旅游景点任你选！（894）
94. 分享一篇文章。（16）
95. 瓦窑堡革命旧址纪念馆：用好红色资源 提升旅游品质（229）
96. 75周年纪念日！新华社重磅文章……（354）
97. 【抗战我们来讲述】纪念抗战胜利70周年广播户外主题体验活动第一站在子长县瓦窑堡革命旧址圆满结束（190）
98. 【子长红色印记】重要机构旧址之中共瓦窑堡支部旧址（26）
99. (子长篇)洞里的瓦窑堡会议（32）
100. ​长征期间，瓦窑堡会议召开（85）
101. 党史故事丨永远的红飘带：从革命长征路到发展新征程（112）
102. 子长市市级领导集体参观纪念瓦窑堡会议召开85周年“瓦窑堡杯”摄影作品展（1119）
103. 【县区动态】子长、富县召开常委会议传达学习市委统战工作会议精神（75）
104. 【荐读】永远的红飘带——从革命长征路到发展新征程（176）
105. 媒体上的子长】“记者再走长征路”你一定知道的瓦窑堡会议（173）
106. 瓦窑堡印象（二）▎原创（215）
107. 省红十字会赴子长开展党史学习教育培训暨“跟党走”感恩奋进活动（122）
108. 【党史知识】|瓦窑堡会议和洛川会议（271）
109. 《学党史·微党课》第三十一集：瓦窑堡会议（120）
110. 永远的红飘带——从革命长征路到发展新征程（1万）
111. 【县区动态】子长市委统战部出版发行《纪念瓦窑堡会议召开85周年——瓦窑堡杯摄影作品集》（120）
112. 子长：一曲信天游 传唱双拥情（326）
113. “陕西100处红色旅游地”发布，西安有这些地方......（663）
114. 您的摄影作品，有机会入选《中国红色旅游影像志》| 征集（2996）
115. 延安干部学院培训部党支部来子长开展党日活动（304）
116. 《中国红色旅游影像志》征集启事（959）
117. 再走长征路 | 瓦窑堡会议：确定建立抗日民族统一战线（137）
118. 子长：恪守初心学党史 为民办事显担当（1113）
119. 最全陕西博物馆春节刷馆攻略 囊括79项展览（310）
120. 陕西子长市：厚植高质量发展红色底蕴（1058）
121. 党史学习教育 || 瓦窑堡会议：“一次极关重要的会议”（13）
122. 子长市举行纪念瓦窑堡会议召开85周年“瓦窑堡杯”摄影作品展（410）
123. 撤县设市一年多，子长交出怎样的答卷？（1555）
124. 恭喜！国务院批准同意子长撤县设市！（428）
125. 抗战胜利纪念日，一起走进陕西这些红色旅游热门目的地！（1208）
126. 子长县瓦窑堡革命旧址需招聘讲解员两名（145）
127. 陕西省延安市延长县、子长县贺氏概况（183）
128. 全国人大图书馆来我市调研红色资源（159）
129. 子长举行纪念瓦窑堡会议召开85周年“瓦窑堡杯”摄影作品展（359）
130. 西安音乐学院来子长市开展陕北红色民歌调研采风系列活动（1467）
131. 【学党史】86年前的今天，瓦窑堡会议召开（518）
132. 《纪念瓦窑堡会议召开85周年——瓦窑堡杯摄影作品集》出​版发行（65）
133. 迎接建党百年丨倾听青年税务党员对党的真情告白（5099）
134. 延安市子长县瓦窑堡革命旧址（84）
135. 我和永嘉信的故事 | 穆东：“嘉"业兴旺，当仁不让（1527）
136. 1935年12月17日，中共中央在陕北子长县瓦窑堡召开重要的政治局扩大会议【瓦窑堡会议】（21）
137. 强根铸魂 立德树人 ——子长市教育科技体育局全面推进思政课建设工作小记（798）
138. 好案例，很给力 ！（1132）
139. 铭记历史 砥砺奋进——写在中国人民抗日战争暨世界反法西斯战争胜利75周年之际（6150）
140. “建党百年红色旅游百条精品线路”发布！江西这些线路你都去过吗？（2430）
141. 中国影像方志 | 子长：黄土高原小城以英雄命名 瓦窑堡凝聚民族抗日力量（8706）
142. 每周聚焦｜西安音乐学院“重走红军路 再唱红军歌” 陕北红色歌曲调研采风组走进子长（191）
143. 【党史日历】（12月17日）瓦窑堡会议召开（12）
144. 文旅部中宣部公布100条红色旅游精品路线！（1.8万）
145. 致敬建党百年 · 红色足迹 | 中国旅游报庆祝建党百年特刊（807）
146. 榆林两红色景区入选全国抗战主题红色旅游精品线路啦！（1.6万）
147. 喜讯！兴安盟被列入“建党百年红色旅游百条精品线路（326）
148. 【庆建党百年 谋开局新篇】兴平马嵬驿入选建党百年红色旅游百条精品线路景点（583）
149. 【日常积累】常识：瓦窑堡会议（270）
150. 资讯 | 文化和旅游部 中央宣传部 中央党史和文献研究院 国家发展改革委关于发布“建党百年 红色旅游百条精品线路”的公告（1994）
151. 【常识】瓦窑堡会议、洛川会议（230）
152. 权威快报 | “建党百年红色旅游百条精品线路”发布（2684）
153. 中省市媒体“记者再走长征路”主题采访活动走进子长县（880）
154. 尤溪这两个地方，上榜“建党百年红色旅游百条精品线路”名单（7329）
155. 官方正式发布！值得收藏！今年，南昌这些地方必去！（3133）
156. 文旅部等四部门联合推出“建党百年红色旅游百条精品线路”（329）
157. 陕西3条线路入选“建党百年红色旅游百条精品线路”（768）
158. 完整版｜CCTV-10中国影像方志—子长：黄土高原小城以英雄命名，瓦窑堡凝聚民族抗日力量（3187）
159. 红色记忆｜子长籍在校大学生闫明慧讲述瓦窑堡会议的故事（1271）
160. “建党百年红色旅游百条精品线路”公布 海南有这3条→（66）
161. 子长市党员干部参观纪念瓦窑堡会议召开85周年“瓦窑堡杯”摄影作品展（167）
162. 两条线路在阿坝！国家多部门联合推出“建党百年红色旅游百条精品线路”（502）
163. “全国红色旅游万里行”今天启动，发布65条抗战主题红色旅游精品线路（2754）
164. 瓦窑堡会议（1）
165. 内蒙古三条线路入选“建党百年红色旅游百条精品线路” 你都去过了吗？（170）
166. 喜讯！特大喜讯！！我市牤牛屯村跻身文化和旅游部等四部门联合发布的“建党百年红色旅游百条精品线路”！！！（1174）
167. 9月3天小长假去哪儿？陕西23处红色旅游热门目的地任你选！（1631）
168. 750）铭记历史 砥砺奋进——写在中国人民抗日战争暨世界反法西斯战争胜利75周年之际（15）
169. 我省三条线路入选“建党百年 红色旅游百条精品线路”（137）
170. |青生活| 9月3天小长假去哪儿？陕西23处红色旅游热门目的地任你选（104）
171. 【快报】榆林两红色景区入选全国抗战主题红色旅游精品线路 | 榆阳区进行食品药品安全检查（198）
172. 安吴青训班入选全国红色游经典景区，红色泾阳欢迎您！（2030）
173. 中央广播电视总台“长征路万里行”移动直播报道团队 在子长县圆满完成“瓦窑堡会议”直播（491）
174. 延安子长： 瓦窑堡会议-----建立抗日民族统一战线（39）
175. 致敬建党百年 · 红色宣讲 | 中国旅游报庆祝建党百年特刊（840）
176. 【资讯】陕西启动长征精神火炬传递 全国火炬将于11月上旬在延安汇集点燃（71）
177. 65条抗战主题红色旅游线路（609）
178. 瓦窑堡会议：吹响民族抗战的号角（148）
179. 毛泽东与瓦窑堡会议（141）
180. 9月3天小长假去哪儿？陕西23处红色旅游热门目的地（2113）
181. 今天，来省内这些红色景区重温历史吧！（306）
182. 看完大阅兵，这些旅游区千万别忘记打卡！（2523）
183. 此间活动 | 爱国与创新——一份来自寒假实践团的邀请（965）
184. 难忘子长（1656）
185. 延川县、子长县要闻（1756）
186. “我最喜爱的十大抗战歌曲”评选 陕北民歌《南泥湾》入选（360）
187. 全国30条红色旅游精品线路推荐（685）
188. 全国308个红色教育基地（排名不分先后）（215）
189. 延安市融媒体中心宣教中心走进子长市感悟革命初心，践行为民办事（213）
190. 重磅！国务院同意陕西子长撤县设市（2023）
191. 【国家公祭日】燃起红色记忆！（289）
192. 红色印记 · 第11期｜党史中的地名：瓦窑堡（425）
193. 【伟大瞬间】瓦窑堡会议（24）
194. 分享一篇文章。（447）
195. 陕西子长 瓦窑堡会议：建立抗日民族统一战线（68）
196. 学党史，守初心 | 瓦窑堡会议：确定建立抗日民族统一战线（159）
197. 子长籍在校女大学生史雪麦讲述瓦窑堡会议的故事（2129）
198. 国庆红·嗨西安 | 爱国传承9处“红色旅游地”，你都去过吗？（3202）
199. 瓦窑堡会议（上）（22）
200. 参观预约：子长市瓦窑堡革命旧址文物管理所联系方式（74）
201. 盈科旅游延安研学夏令营开营：追忆红色岁月，传承延安精神（1482）
202. 特别策划｜疫情期间的会奖旅游全攻略（651）
203. 子长：按下发展“快进键”（800）
204. 30条红色旅游线路早知道（508）
205. 陕西人都来这里旅游打卡了！陕西“红色旅游”受热捧（2691）
206. 瓦窑堡会议（下）（23）
207. 又上榜了！凤县被全国红色旅游点名了（1276）
208. 高原情韵瓦窑堡 山清水秀子长城（1529）
209. 一键收藏，四部门联合推出“建党百年红色旅游百条精品线路”（4073）
210. 【精品线路】一键收藏，四部门联合推出“建党百年红色旅游百条精品线路”（79）
211. 【骑行新篇】亲吻陕甘宁，抱拥腾格里！（内附视频）（1158）
212. 子长：天下堡，瓦窑堡（47）
213. 红色路线大合集，记得收藏！（128）
214. 子长县:百余棵柏树精心护养四十年竟被子长路政大队无端砍伐了（1434）
215. 年轻人都来这里旅游打卡了！陕西“红色旅游”受热捧（219）
216. 红色旅行正当时，4条红色旅游美食路线大盘点（7029）
217. 第24期 瓦窑堡会议（7）
218. 纪念抗战胜利70周年广播户外主题体验活动第二站在志丹县保安革命旧址圆满结束《木木读诗》《你好延安》（193）
219. 每日一学|瓦窑堡会议（59）
220. 在“落脚点”落脚，在“出发点”出发（1万）
221. 你！不知道的潮流红色旅游打卡景点--拒绝呆板，玩出新花样（114）
222. 国务院公布第一批国家级抗战纪念设施、遗址名录（956）
223. 瓦窑堡会议召开（24）
224. 关于组织会员企业2018-2020年国内外考察调研征求意见的通知（323）
225. 央视一套黄金档热播剧《淬火成钢》编剧李茂林解读瓦窑堡会议（597）
226. **视频：58**
227. **延安革命纪念馆,枣园革命旧址,杨家岭革命旧址,王家坪革命旧址,凤凰山革命旧址,清凉山革命旧址,“四八”烈士陵园,洛川县洛川会议纪念馆,**

**洛川县洛川会议纪念馆**

1. 党史课堂 | 洛川会议纪念馆（117）
2. 喜报！洛川会议纪念馆被命名为陕西省青少年教育基地！（26
3. 洛川会议纪念馆召开“五一”节前工作安排部署会（80）
4. “五一”小长假洛川会议纪念馆迎来旅游“热”（209）
5. 观“洛川会议”遗址，重温革命历史（97）
6. 洛川会议纪念馆（56）
7. 【红色堡垒·百年党史】红色地图 | 爱国主义教育基地：洛川会议纪念馆（31）
8. 【美丽瞬间随手拍】洛川会议纪念馆（89）
9. 【旅游】洛川会议纪念馆（856）
10. 洛川会议纪念馆疫情防控须知（300）
11. 【沿着高速学党史】洛川会议纪念馆（88）
12. 洛川会议纪念馆以“铭记历史，勿忘九一八”为主题开展纪念活动（418）
13. 乡村游必去打卡地！美的不止是风景~（1470）
14. 洛川会议纪念馆获赠一批珍藏文史档案资料（989）
15. 百篇陕西红色经典|第六十六篇 洛川会议纪念馆（179）
16. 洛川会议纪念馆闭馆公告（238）
17. 陕西日报 | 一次具有重大转折意义的会议 ——探访洛川会议纪念馆（780）
18. 洛川会议纪念馆开展“传承红色基因 建设文明家园”进社区宣讲活动——走进城市社区服务中心（160）
19. 百年华诞 旅动中国 — — 走进洛川会议纪念馆（2113）
20. 关于洛川会议纪念馆恢复开放的公告（164）
21. 云游红色纪念馆——【陕西篇】洛川会议纪念馆（55）
22. 洛川会议纪念馆临时闭馆公告（328）
23. 【史钩 屈发全】赵紫阳总理参观“洛川会议”纪念馆走访冯家村农民家庭记实（372）
24. “四史”学习|经典打卡——洛川会议纪念馆（292）
25. 全国网媒延安行｜走访革命圣地——洛川会议纪念馆（121）
26. 铭记|国家级抗战纪念设施、遗址名录-洛川会议纪念馆（38）
27. 喜迎建党百年 云访红色基地|第七站：洛川会议纪念馆（245）
28. 【视频】用声音迎接春天—洛川会议纪念馆在行动（456）
29. 洛川会议纪念馆举办“虔诚守望 砥砺前行”文物保护历程展（150）
30. 党史展馆巡展I洛川会议纪念馆（18）
31. 陕北信天游：洛川会议旧址纪念馆（56）
32. 【魅力洛川】洛川会议纪念馆（25）
33. 刘全芳 | 洛川会议旧址的自然传奇（之三）（279）
34. 洛川会议纪念馆开展“四进”活动，为大家送去红色文化“大餐”（8）
35. 在学习中成长——洛川会议纪念馆开展讲解员业务知识提升月活动（70）
36. 【党史博览】一次具有重大转折意义的会议——探访洛川会议纪念馆（99）
37. 延安市洛川县洛川会议旧址暨纪念馆（77）
38. 分享一篇文章。（29）
39. 活 动 | 洛川会议纪念馆开展红色文化进军营活动（730）
40. 百年党史重要会议 | 历史转折关头的重要会议——洛川会议（68）
41. 洛川会议纪念馆线上讲解《抗战灯塔—洛川会议》第二期（295）
42. 【党史珍闻】一次具有重大转折意义的会议——探访洛川会议纪念馆（131）
43. 【红色记忆】洛川会议纪念馆（397）
44. 洛川会议纪念馆启动文化和自然遗产日系列活动二献礼建党百年 传承红色基因（29）
45. 洛川会议纪念馆开展“弘扬爱国精神 强化爱国意识”主题宣讲活动——走进洛川县中学（149）
46. 王贞祥：洛川会议纪念馆——红色旅游之一（96）
47. 洛川会议纪念馆开展“致敬最可爱的人 红色文化进军营”为主题的宣传活动（12）
48. 洛川会议纪念馆启动文化和自然遗产日系列活动—传承红色基因 助力科学普及（11）
49. 洛川会议纪念馆线上讲解《抗战灯塔—洛川会议》第一期新鲜出炉（265）
50. 在学习中成长—洛川会议纪念馆开展讲解员业务知识提升月活动（二）（52）
51. 洛川会议纪念馆与洛川县消防救援站开展“红蓝交融·党建联盟”活动（38）
52. 德坤干训 | 爱国主义教育基地系列——陕西篇（8）
53. 前方发现火情！别慌，是洛川会议纪念馆在演练！（230）
54. 第一话：洛川会议纪念馆（25）
55. 洛川会议纪念馆走进交口河镇中心小学开展主题教育活动（248）
56. 百年百家红色馆第二期（95）
57. 全国乡村旅游精品线路之陕西篇——“走进革命圣地 感悟初心使命”红色之旅（582）
58. 铭记光辉历史、传承红色基因—海外华媒代表探访洛川会议纪念馆（23）
59. 延安这条线路入选全国乡村旅游精品线路！国庆就安排~~（347）
60. 一场红色之旅，不能少了去洛川会议纪念馆（5）
61. 【青海机场航班换季，一起追寻红色足迹 | 西北篇】（1250）
62. 参观“洛川会议旧址”有感 ​作者: 靳梦虎（227）
63. 洛川会议纪念馆线上讲解活动第六期—《抗战灯塔-洛川会议之游击战》（299）
64. 党史知识云课堂 | 洛川会议（98）
65. 【党史学习教育】洛川会议背后的故事（134）
66. 洛川会议纪念馆线上讲解活动第八期-《抗战灯塔-洛川会议之隐蔽战线上的较量》（203）
67. 洛川县举行纪念中国人民抗日战争暨世界反法西斯战争胜利75周年活动（1396）
68. 洛川会议纪念馆，毛主席和周总理来过这个地方，带大家完整走一遍（514）
69. 洛川小伙带你看（洛川会议纪念馆）（270）
70. 洛川会议纪念馆开展红色文化进军营活动（185）
71. 洛川会议纪念馆线上讲解活动第三期《抗战灯塔—洛川会议之洛川会谈》》（118）
72. 领略红色基因，海内外华文媒体走进洛川会议纪念馆参观学习（3）
73. 徒步西藏108天，到达洛川会议纪念馆（9）
74. 敬礼，中国军人！此生必去的红色旅游景点（144）
75. 百年·留声机㉙ |《洛川会议——为全党指明抗战胜利的道路》（121）
76. “中国梦肢队”重走长征路走进洛川会议纪念馆（7）
77. 洛川会议：历史转折关头的重要会议（59）
78. @延安人，一大波“五一”文旅活动正向您靠近~~（640）
79. 同心筑梦向未来---洛川县市场监管局非公经济组织部分党员走进洛川会议纪念馆开展党日活动（242）
80. 《革命圣地 洛川会议》剪纸艺术讲座在洛川文化馆成功举办（1205）
81. 陕西洛川会议纪念馆 爱国主义教育的课堂（15）
82. 全面抗战 全民抗战——洛川会议背后的故事（964）
83. 【八一建军节】此生必去的红色旅游景点！（49）
84. 老区新貌 | 洛川：革命老区果香飘（1040）
85. 【非遗 屈发全】洛川蹩鼓（230）
86. 【灞桥教育 宇小德育】红领巾爱学习——记宇航小学假期爱国教育德育实践活动（四）（192）
87. 张广生丨金色九月相约洛川（322）
88. 欢迎参观洛川会议旧址！（308）
89. 【百年华彩】第六讲：全面抗战 全民抗战——洛川会议背后的故事（182）
90. 微党课 | 洛川会议（533）
91. 学习百年党史，汲取奋进力量——富县交警大队走进“洛川会议”旧址纪念馆，接受革命传统教育（412）
92. 【党史课堂】| 第十九课 |全面抗战 全民抗战——洛川会议背后的故事（159）
93. 传承红色基因，铭记光辉历程——九三学社医学部支社赴洛川会议纪念馆开展党史教育活动（11）
94. 照金纪念馆应邀参加“洛川会议”召开80周年纪念活动（364）
95. 洛川会议的旧址竟然在这里！！（50）
96. 文旅部推荐，这条红色旅游线路带你感悟初心使命！（1260）
97. 洛川县政府机关党委开展庆祝“七一”活动（212）
98. 策划·党史上的今天 | 洛川会议（1105）
99. 抗战胜利纪念日，一起走进陕西这些红色旅游热门目的地！（1208）
100. 采 访 | 听他们谈洛川会议（393）
101. 【党史课堂】| 第十七讲 |全面抗战 全民抗战——洛川会议背后的故事（24）
102. 党建 | 脱贫攻坚、暖心助农，地理标识寻源行（119）
103. 今天，来省内这些红色景区重温历史吧！（306）
104. 刘全芳丨红色洛川原（486）
105. 政府机关党委开展庆祝“七一”活动（824）
106. 陕西这份旅游百名榜，西安占9个！“十一”假期正好去看看（2053）
107. 一路向北，骑行内蒙古之1：初试4+2，阎良-甘泉，参观洛川会议纪念馆（171）
108. “陕西100处红色旅游地”发布，西安有这些地方......（663）
109. 创文进行时 | 现场指导，促进洛川景区景点创文工作高质量开展（180）
110. 红色旅游，我们在路上（107）
111. 9月小长假去哪里？ 延安15处红色旅游景点任你选！（894）
112. 纪念抗日战争胜利70周年大型广播户外直播主题体验活动第三站今天在洛川会议旧址圆满结束（127）
113. 五一·一起浪·走——延安乡村旅游之洛川篇（2633）
114. 精选陕西这几条红色旅游线路，带娃踏上红色之旅吧！（1701）
115. 头 条 | 洛川会议80周年纪念大会在洛川县召开（3624）
116. 洛川县纪委监委组织新任职年轻干部开展廉洁从政现场教育活动（693）
117. 党史天天读 | 洛川会议：全面抗战 全民抗战（113）
118. “喜迎二十大，庆建团百年”团干部讲团史系列（九十八）——“重走抗战路·青春向延安”（105）
119. 【党史故事汇】洛川会议：党的全面抗战路线形成（545）
120. 【陕西洛川 王智斌】原创//八月 邀君洛川来观光（248）
121. 纪念洛川会议胜利召开80周年重走红色之路陕西省山地越野徒步公开赛在洛川成功举办（1.1万）
122. 党史学习教育专题七十九：洛川会议的召开（575）
123. 春节假期 洛川县接待游客10.84万人次（629）
124. 庆国庆 迎重阳 贺丰收 感党恩 洛川县中心敬老院走进阿寺村、洛川会议纪念馆（394）
125. 受天气影响，这些地方有的景区暂停开放（1480）
126. 延安大部分县城在山沟沟里，只有这个县城是一马平川的塬！（484）
127. 陕西有10处国家级抗战纪念设施、遗址，咱延安就有6处！（569）
128. “非热门系列”|“十一”出游， 这些地方人少（2104）
129. 洛川“双节”旅游平稳有序 接待游客 5.5万人次（1432）
130. 南京学子说西安|文化氛围浓厚 交通问题颇多（86）
131. 组织建设 | 洛川县举行“青春心向党，百年正当时”百名新党员集体宣誓活动（674）
132. 青春心向党：洛川县举行百名新党员集体宣誓仪式（695）
133. “传承红色基因 助力科学普及”大型义诊走进洛川县（230）
134. 赛 事｜挑战自我！快来洛川县参加山地徒步越野公开赛吧！（1440）
135. 百年党史掠影 | 历史上的大事⑥（309）
136. 洛川：多种形式开展“主题党日”活动（847）
137. 华纳文化：国庆期间，为你准备了陕西这些红色旅游景区攻略！（43）
138. 具有代表性、重要性、文化性的历史遗产——陕西首批历史文化名镇名村街区公布（1557）
139. 洛川县举行2021年新发展党员集体入党宣誓活动（598）
140. “一道道的那个山来哟一道道水”，鸟瞰陕北革命旧址新风貌（668）
141. 省健康环境研究所赴洛川会议纪念馆和马栏革命旧址开展党史学习教育实践活动（78）
142. 桑兵 | 抗日战争的持久战要多久——国共高层的抗战时长预判（3616）
143. 周边特价| 国庆假期到处人人人，玩得不过瘾，那周末继续吧！（1854）
144. 来来来 ，看“五一”延安乡村旅游菜单啦！（998）
145. 文明旅游 | “五一”无须去他乡今日延安胜可游（173）
146. 我在延安等你来（1.6万）
147. 银川市委组织部考察我县红色美丽村庄建设工作（404）
148. 每日一战：敌进我进 敌后抗战（2390）
149. 洛川县开展“企地共建”廉政建设暨加强年轻干部廉洁从业教育监督管理主题活动（529）
150. 榆林两红色景区入选全国抗战主题红色旅游精品线路啦！（1.6万）
151. 荣心向党 礼赞百年 |学党史、知党情、跟党走（第五期）（294）
152. 共青团洛川县委开展“喜迎二十大、永远跟党走、奋进新征程”主题团日活动（449）
153. 红色回响——延安革命纪念馆（180）
154. 特别策划｜疫情期间的会奖旅游全攻略（655）
155. 延安：庆祝建军90周年 军民鱼水一家亲（1102）
156. 乡村四时好风光，来汉中千万别错过这些地方→（619）
157. 党建在线｜轨道系各党支部开展喜迎十九大特色党日活（356）
158. 我院学生赴洛川县见习（386）
159. ​贠恩凤领衔洛川群众再唱《十唱共产党》（330）
160. |青生活| 9月3天小长假去哪儿？陕西23处红色旅游热门目的地任你选！（104）
161. 重走洛川革命老区，探寻党的足迹——西安思源学院理工学院暑期到洛川“三下乡”活动纪实（508）
162. “五一”无须去他乡 今日延安胜可游（707）
163. “五一假期”无须去他乡 今日延安胜可游（2179）
164. 洛川县2017年十大新闻出炉啦！（6364）
165. 【慢城 ·时光】西安周边免费景点大全，民俗村、王顺山...一个也不能错过！（483）
166. 黄玉良 | 致敬传统 踏雪迎春赏春联（1276）
167. 【微曝光】延安：公开曝光7起违反中央八项规定精神问题（841）
168. 2022年洛川怎么干？全会这样部署（2518）
169. 喜迎二十大、奋进新征程——洛川县中学团委开展五四主题团日活动（392）
170. 陕西最新免费景点大全，不知道就亏大了！（3222）
171. 社会实践|“喜迎二十大 助力乡村振兴”专项计划——走进民俗博物馆、探访阿寺村（185）
172. 延安市各级团组织积极开展“喜迎二十大、永远跟党走、奋进新征程”主题教育实践活动（1553）
173. 5月6日 洛川将举行这项体育赛事 喜欢的可千万不要错过哦！（905）
174. 重走抗战路Day2：我们在路上，涤荡青春！（2348）
175. 【“四史”记忆】为什么说洛川会议吹响了全面抗战的号角（961）
176. 收藏！这14条陕西乡村旅游路线个个皆精品（674）
177. 抗战主题景点受热捧 盘点陕西红色旅游热门目的地（1506）
178. 9月3天小长假去哪儿？陕西23处红色旅游热门目的地任你选！（2584）
179. 乡村四时好风光，这14条陕西乡村旅游精品路线快快收藏！（3255）
180. 你！不知道的潮流红色旅游打卡景点--拒绝呆板，玩出新花样（115）
181. 【不要钱】2017陕西最新免费景点大全，这些A级景区通通不要钱，不知道就亏大了！（194）
182. 「旅游」9月3天小长假去哪儿？陕西23处红色旅游热门目的地任你选！（1551）
183. 献礼百篇|第九十一期：青春心向党 建功新时代 ——记2015年“重走抗战路·青春向延安”主题教育实践活动（117）
184. 这里曾经所经历的战火纷飞，需要你我走近才能了解——（508）
185. 中央媒体“走进红色美丽村庄”网络主题宣传采访活动走进洛川（1687）
186. 【从红船到巨轮】抗战篇 第二章：洛川会议 全面抗战（753）
187. 【党建课堂】从红船到巨轮| 抗战篇 第二章：洛川会议 全面抗战（15）
188. 文旅部最新乡村旅游线路公布，佛坪这个地方上榜！（157）
189. 学技术 塑理念 观世界 提认知 学员直呼：干货太多（55）
190. 【“四史”学习时间（十四）】为什么说洛川会议吹响了全面抗战的号角？（211）
191. 洛川会议中的保密工作（671）
192. 红色印迹 | 寻访洛川会议纪念馆，从抗战精神中凝聚奋进力量（32）
193. 【争做新青年 奋进新征程】洛川会议纪念馆喊你来打卡！（60）
194. 沿着高速学党史—洛川会议纪念馆（290）
195. 【探访】一次具有重大转折意义的会议 ——探访洛川会议纪念馆（311）
196. 【学习卡片】全国爱国主义教育示范基地（陕西篇）：洛川会议纪念馆（14）
197. 洛川会议纪念馆开展2020年“5.18”国际博物馆日主题宣传活动（171）
198. 洛川会议纪念馆召开“国庆”节前安全工作暨疫情防控安排部署会（40）
199. 致敬先辈 吾辈自强——洛川会议纪念馆举行纪念全民族抗战爆发85周年活动（202）
200. 【党史大学习(五十二】之走进洛川会议纪念馆（131）
201. 陕西中红延育 | 见证历史——洛川会议纪念馆（15）
202. 洛川会议纪念馆简介（274）
203. 洛川会议纪念馆开展2022年文化和遗产日活动——同心共绘时代梦（255）
204. 洛川会议纪念馆开展2022年“5.18国际博物馆日”主题宣传活动（243）
205. 洛川会议纪念馆开展“致敬最可爱的人 红色文化进军营”为主题的宣传活动（197）
206. 洛川会议纪念馆开展“重温红色记忆 追忆峥嵘岁月”主题宣讲活动——走进洛川县消防大队（175）
207. 玉麟小学红色印记班本课程之洛川会议纪念馆（31）
208. 洛川会议纪念馆开展“全民参与保护 资源你我共享”文化和自然遗产日宣传活动（120）
209. 洛川会议纪念馆官方网站首发信息（0）
210. 喜报！我市20家单位被命名为陕西省青少年教育基地！（1918）
211. 欢迎关注洛川会议纪念馆官方网站！（朋友圈分享版）（446）
212. 洛川会议纪念馆开展“新生开学第一课 革命精神永传承”红色文化进校园活动（147）
213. 洛川会议纪念馆举行纪念全民族抗战爆发83周年活动（300）
214. 洛川会议纪念馆开展“传承革命传统 讲好红色故事”进企业宣讲活动——走进陕西顶端果业科技有限公司（304）
215. 【图片新闻】国际博物馆日：洛川会议纪念馆开展主题宣传活动（306）
216. 红色地标—红色景区中国万里行—洛川会议纪念馆直击现场（59）
217. 洛川会议纪念馆开展“纪念中国人民抗日战争暨世界反法西斯战争胜利75周年”系列活动（194）
218. 2022年2月24日 | 他把活字印刷搬上屏幕，让汉字也能网上“冲浪”（1758）
219. 刘瑶女士向洛川会议纪念馆捐赠珍贵照片（38）
220. 刘全芳 | 洛川会议旧址的自然传奇（之一）（651）
221. @延安人，一大波“五一”文旅活动正向您靠近~~（438）
222. 领略红色基因，海内外华文媒体走进洛川会议纪念馆参观学习（2）
223. 洛川会议纪念馆开展“弘扬传统优秀文化 建设美丽和谐家园”主题宣讲活动（50）
224. 洛川会议纪念馆、洛川县博物馆共同举办“学用新思想、奋进新时代”庆七一红歌大家唱主题党日活动（152）
225. 招聘| 洛川会议纪念馆讲解员招聘，可愿赴场未来之约？（866）
226. 全国各地纪念馆同步举行纪念全民族抗战爆发85周年活动（635）
227. 基层单位学党史——准备队 参观洛川会议纪念馆之感（84）
228. 铭记历史缅怀先烈 开创更美好的未来（195）
229. 参观洛川会议纪念馆——记盟直属展馆讲解员培训第五日（27）
230. 洛川会议纪念馆开展消防演练活动（165）
231. 洛川会议纪念馆“红色文化”进企业活动走进洛川琦泉生物质发电有限公司（110）
232. 百年·留声机㉙ | 洛川会议——为全党指明抗战胜利的道路（259）
233. 以史为鉴 开创未来——各地纪念馆联动纪念全民族抗战爆发84周年（1207）
234. 【党史故事汇】洛川会谈：洛川会议背后的故事（1689）
235. 【党史学习教育】林甸县水务局开展云参观洛川会议纪念馆活动（216）
236. 【党史学习教育】林甸县交通运输局云连线参观洛川会议纪念馆（168）
237. 洛川会议纪念馆线上讲解活动第九期-《抗战灯塔-洛川会议之解放洛川之战》（207）
238. 传承红色基因 培育时代新人 —旧县中小徒步洛川会议纪念馆研学活动（229）
239. 天安人力党支部纪念建党99周年活动——参观洛川会议纪念馆（97）
240. 分享一篇文章。（14）
241. 市文化和旅游局调研员刘晓虎检查指导洛川会议纪念馆“五一”疫情防控工作（219）
242. 学党史 强信念 跟党走 | 歇赛期 铜川篮协组织球队赴洛川会议纪念馆开展党史学习教育（468）
243. 盘点陕西23处红色旅游热门目的地（544）
244. 洛川会议纪念馆线上讲解活动第十期-《抗战灯塔-洛川会议之中共特工之王李克农》（160）
245. 洛川会议纪念馆线上讲解活动第四期——八路军的改编（112）
246. 洛川会议纪念馆线上讲解活动第五期—《抗战灯塔-洛川会议之抗日救国十大纲领（131）
247. 中国延安干部学院厅局级干部培训班来洛川县开展现场教学（1174）
248. 陕西14条乡村旅游精品路线中那些不容错过的体验地（附地图（252）
249. 宜富分公司机关党支部赴洛川会议革命纪念馆开展党史学习教育实践活动（299）
250. 铭记历史、缅怀先烈、珍爱和平、开创未来——国内外纪念馆联动开展“纪念中国人民抗日战争暨世界反法西斯战争胜利75周年”（2216）
251. 【假日自助游】|乡村四时好风光，陕西乡村旅游渭南精品路线快快收藏！（10）
252. 【百年荣光·党史知识】我党提出全面抗战路线的会议是？（4170）
253. 洛川会议纪念馆里学党史（116）
254. 西安这些景点向全国人民免费开放啦！景区名单已列好，速戳！（2.6万）
255. 洛川会议纪念馆开展“四进”活动，为大家送去红色文化“大餐”（233）
256. 洛川会议纪念馆全体职工为中国共产党成立100周年献礼（211）
257. 【党史学习】歇赛期 铜川篮协组织球队赴洛川会议纪念馆开展党史学习教育（95）
258. 传承红色基因 培育时代新人 ——旧县镇中心小学徒步洛川会议纪念馆研学活动（96）
259. 2017陕西最新免费景点大全，这些A级景区通通不要钱，不知道就亏大了！（1.5万）
260. 洛川会议纪念馆成为党史学习大课堂（255）
261. 洛川会议纪念馆启动文化和自然遗产日系列活动二献礼建党百年 传承红色基因（77）
262. 学党史 悟思想 | 数统学子学党史之洛川会议（1296）
263. “重走长征路 精神永相传 ”第四站 西安八路军办事处、洛川会议纪念馆（240）
264. 洛川会议纪念馆启动文化和自然遗产日系列活动—传承红色基因 助力科学普及（150）
265. 陕西红色革命纪念地摄影美图大放送：延安篇！（997）
266. 收藏！陕西这209个绝美景点全免费，坐等下一个假期！（6938）
267. 踏遍陕西之洛川—高原苹果（洛川会议纪念馆和民俗馆）（61）
268. 2017陕西最新景区门票大全，没想到有这么多免费的！收好够用一整年（8054）
269. 【爱国教育】播种精神 孕育未来——洛川会议纪念馆爱国主义教育走进秦关中心小学（261）
270. 中国文联文艺志愿者参观洛川苹果标准化生产示范园并在洛川会议纪念馆进行慰问演出（824）
271. 9月3天小长假去哪儿？陕西23处红色旅游热门目的地（2113）
272. 洛川会议纪念馆线上讲解活动第七期-《抗战灯塔-洛川会议之鄜城桥伏击战》（253）
273. 国庆红·嗨西安 | 爱国传承9处“红色旅游地”，你都去过吗？（3202）
274. 读文献 学党史 | 党的建设“伟大工程”的提出（1821）
275. 吐血整理！最新2017陕西景区门票价格大全！竟有这么多免费的！！（1.7万）
276. 陕西209个免费景点，230多个收费景点大全！建议收藏（5109）
277. 洛川会议纪念馆开展“5.18国际博物馆日”系列活动（143）
278. 张怀清丨走进洛川中心敬老院（2363）
279. 【浴血抗战 历史丰碑】圣地延安 民族希望（193）
280. 中共铜川市住房公积金管理中心支部赴洛川会议纪念馆开展四月份主题党日活动（535）
281. 【学习】凤栖社区服务中心东关社区20多名志愿者到洛川会议纪念馆参观学习（256）
282. 陕菜情缘 | 芳香四溢 饭香果香说洛川（963）
283. 【纪念建党95周年】伟大的历程——洛川会议（51）
284. 14条陕西乡村旅游精品路线爸妈快快收藏！（30）
285. 这一天，不能忘记！（120）
286. 本周就是五一小长假了！出行的小伙伴们来看看2017陕西景区门票价格大全！竟有这么多免费的！！（1735）
287. 学党史 | 党的建设“伟大工程”的提出（973）
288. 乡村四时好风光 | 14条陕西乡村旅游精品路线最新发布！（54）
289. 洛川职专与洛川会议纪念馆共建校外实训基地协议签订仪式（228）
290. 陕西：黄帝陵景区等众多景区将对全国医护工作者免费开放（498）
291. 浴血抗战 历史丰碑丨敌我力量悬殊 中国共产党如何将敌后变为前线？（177）
292. 双节合一，家国同庆！今天，渭师人这样向祖国深情表白！（2865）
293. 果博专列 | 大美洛川 快乐采摘——第五届洛川苹果观光旅游采摘月拉开帷幕（6520）
294. 红色路线大合集，记得收藏！（128）
295. 甘泉县、洛川县要闻（180）
296. 陕西省书法家协会与贵州省遵义市书法家协会红色联合采风剪影（6）
297. 我的2021 | 侃侃我的这一年（658
298. 学党史悟思想| 美育教育融入课程思政——学校“学四史”主题美术作品成果展(二)（517）
299. 勿忘国耻，让“九一八”的历史警钟常驻心中（176）
300. 苹果树下的党课（1536）
301. 别被抖音带歪了，我用200张图带你领略陕西旅游的真正魅力！（5297）
302. 新闻 | 陕西命名一批青少年教育基地，多数为文博场馆，大唐秦王陵博物馆榜上有名（45）
303. 【湘潭抗战英雄谱】“人民战争”缔造者毛泽东（245）
304. “中国梦肢队”重走长征路走进洛川会议纪念馆（242）
305. 【轻歌 屈发全】人民敬仰毛泽东（131）
306. 30条红色旅游线路早知道（509）
307. 高压线下野蛮施工，罚款10000元！（7万）
308. 【贯彻全会精神 奋力谱写新篇章】向人民报告——洛川县永乡镇（928）
309. 【跟党走的一百年·团史故事100讲】 第七十九期:维护网络安全，共忆抗战70周年（23）
310. 大型历史篆刻作品《篆刻红色革命 延安印》捐赠仪式在洛川举行（208）
311. 喜报｜八路军西安办事处纪念馆入选“陕西青少年教育基地”（106）
312. 喜报！邓宝珊将军纪念馆被命名为陕西省青少年教育基地（218）
313. 刘建锋检查指导永乡镇防汛救灾工作（1093）
314. 洛川会议纪念馆开展2020年冬季火灾防控培训及演练活动（91）
315. 全国30条红色旅游精品线路推荐（686）
316. 魅力打通致富之路！红色旅游全域旅游带动延安脱贫（107）
317. 延安日报 | 村容村貌大变样 颜值内涵齐提升——洛川县永乡镇冯家村依托红色文化建设美丽村庄（583）
318. 洛川会议纪念馆开展“党课开讲啦”讲解员大练兵活动（103）
319. 【党史学习教育在一线】47所：党史学习有“滋”又有“味”（834）
320. 《德育报·校长与班主任工作》总第1631期目录（457）
321. “西安年”后“醉西安”！灯光节、风筝节……这样的长安春色让人沉醉！（9888）
322. 会 议｜张继东在听取永乡镇特色小镇策划方案时强调：要结合洛川本地特色，打造中国第一苹果小镇（580）
323. 社会实践联播 | 渭南师范学院“书记小喇叭”赴圣地延安传承红色精神实践团开展“永远跟党走”主题社会实践活动（790）
324. 属于我们洛川人的洛原风情旅行社今日开业了！（1112）
325. 党史学习教育 | 西北分公司“三字经”推进党史学习教育走深走实（3245）
326. 陕西命名一批青少年教育基地，榆林多个文博场馆上榜（469）
327. 党史微课 | 党的建设“伟大工程”的提出（20）
328. 春满中国醉西安！42家景区优惠活动来袭，灯光节、风筝节…等你约（3560）
329. 就在明天！“西安年”后“醉西安”，这场沉醉于春色的直播，你不能错过！（1824）
330. 李世斌丨集票随感（152）
331. 江西省文联赴陕参加“血脉传承 不忘初心”走进革命圣地延安红色文化 联合采风活动（483）
332. 渭南师范学院“书记小喇叭”暑期赴革命圣地延安深入开展党史学习教育（2231）
333. 奋斗百年路 启航新征程 | 立塔架线为革命老区（1164）
334. 洛川：别开生面庆“七一”（3399）
335. 【陕西传媒网】9月3日放假 旅行社欲推“抗战游” 当天可拿双薪（88）
336. 中国共产党在卢沟桥事变中的策略与应对（下）（2465）
337. “重温百年党史 坚定初心使命”西安石油大学党史教育培训邀请函（72）
338. 陕西命名一批青少年教育基地，多数为文博场馆（1661）
339. 陕西命名一批青少年教育基地，多数为文博场馆，快来看看吧！（76）
340. 不忘初心 继往开来——洛川召开“洛川会议”80周年纪念大会（388）
341. 年轻人都来这里旅游打卡了！陕西“红色旅游”受热捧（220）
342. 乡村四时好风光，陕西乡村旅游渭南精品路线快快收藏！（143）
343. 【贯彻全会精神 奋力谱写新篇章】向人民报告——洛川县杨舒便民服务中心（1668）
344. 【百年党史】——洛川会议：历史转折关头的重要会议（9）
345. 2019年文化和自然遗产日 来延安感悟“人生的来处”（24）
346. 纪念抗战胜利70周年广播户外主题体验活动第二站在志丹县保安革命旧址圆满结束《木木读诗》《你好延安》（193）
347. 延安亲人泣血呼唤：娃呀，你们在哪里？能吃饱穿暖吗？（1万）
348. 今起，陕西新一轮降雨来袭！省防总发布最新通知要求！（1478）
349. 历史应该被铭记！ 2019年文化和自然遗产日 来延安感悟“人生的来处”（246）
350. “几回回梦里回延安”！明天是文化和自然遗产日，第一新闻带您走进延安看革命文物（439）
351. 【国家公祭日】燃起红色记忆！（289）
352. “几回回梦里回延安”！明天，陕视新闻、昌明坊带您走近革命文物（177）
353. 革命文物保护利用的陕西经验（1177）
354. 延安路上，你不能不知的安吴青训班（2118）
355. 向榜样学习丨我校学生陈柯帆、韩雨馨荣获2021年度“中国大学生自强之星”奖学金！（9986）
356. 【新工院】传承延安精神 牢记育人使命——马克思主义学院暑期实践研修顺利结束（2670）
357. 旅游景点天气预（4）
358. 点赞！山阳县1家单位被命名为陕西省青少年教育基地（85）
359. 向榜样学习丨我校两名学生荣获2021年度“中国大学生自强之星”奖学金（2234）
360. 点赞！镇安1家单位被命名为陕西省青少年教育基地（46）
361. 商洛7家单位被命名为陕西省青少年教育基地（83）
362. 重走红色革命路 延安精神永流传——记2016年国家林业局直属机关青年干部 “学党史、知党情、跟党走”主题团日活动（177）
363. 周末天气转好，早晨气温较低注意保暖！（附旅游景点天气预报及未来一周天气）（70）
364. 点赞！商洛7家单位被命名为陕西省青少年教育基地（7611）
365. 【骑行新篇】亲吻陕甘宁，抱拥腾格里！（内附视频）（1158）
366. 300名著名抗日英烈 和英雄群体名录公布（271）
367. 韩城市4个单位被命名为陕西省青少年教育基地（991）
368. 陕西命名一批青少年教育基地，商洛这些单位入选！（158）
369. 恭喜!吴起一地被省上正式命名!（281）
370. 重走抗战路 青春向延安（5534）
371. 又上榜了！凤县被全国红色旅游点名了（1276
372. 【快报】榆林两红色景区入选全国抗战主题红色旅游精品线路 | 榆阳区进行食品药品安全检查（198）
373. 【奋斗百年路 启航新征程】党史直播课　走“新”更走心（99）
374. 安吴青训班入选全国红色游经典景区，红色泾阳欢迎您！（2030）
375. 国务院公布第一批国家级抗战纪念设施、遗址名录（956）
376. 以史明志激发奋进力量！烟草行业这样扎实开展党史学习教育（5886）
377. ​韩城市薛峰水库建设事迹陈列馆被命名为陕西省青少年教育基地（54）
378. 企业占11个，研学基地有3个！2022年青少年教育基地名单公布！（616）
379. ​西安博物院等131个单位被命名为陕西省青少年教育基地（620）
380. |青追忆| “重走抗战路·青春向延安”全国优秀青年代表 纪念抗战胜利70周年主题活动 在西安启动（20）
381. **视频：**

**“四八”烈士陵园**

1. 【党史知识】陕西革命旧址云上展：延安“四八”烈士陵园（205）
2. 打卡廉政教育基地 | 陕西革命旧址云上展：延安“四八”烈士陵园（22）
3. 支部学堂丨“四八”烈士纪念碑碑文敬读（17）
4. 致敬英魂——“四 · 八”烈士陵园祭扫活动（302）
5. 红旗漫卷——陕西革命旧址云上展之十六：延安四八烈士陵园（16）
6. 为人民而死虽死犹荣——“四八”烈士纪念碑碑文敬读（58）
7. 百个红色景点“云”参观㊹ | “四·八”烈士陵园（48）
8. 百日百步新跨越 | 第七十六步：四八烈士陵园（54）
9. 【党史学习】重游初心之地⑤——陕西革命旧址：延安“四八”烈士陵园（203）
10. 延安四八烈士陵园——青山埋忠骨，延河佑英魂（56）
11. 陕西革命旧址云上展：延安“四八”烈士陵园（10）
12. 【阳光军魂】第1012期<张勇>第一吻献给英雄烈士的张茹姐姐走进延安“四八”烈士陵园祭英烈！（133）
13. 暑期三下乡｜瞻仰革命先烈，祭奠烈士英魂：四八烈士陵园（112）
14. 夜——瞻仰“四八烈士陵园”有感（135）
15. 【红色映象】延安四八烈士陵园（41）
16. 延安红色遗址：“四八”烈士陵园（56）
17. 为有牺牲多壮志（28）（5）
18. 【二支部党史学习|红色映像】陕西革命旧址云上展：延安“四八”烈士陵园（96）
19. 主题党日活动:清明节“四八”烈士陵园扫墓（535）
20. 四八烈士陵园祭奠|“继承先烈遗志，献身民族复兴”（328）
21. 娄勤俭、胡和平向“四·八”烈士敬献花篮（350）
22. 忠魂永驻延安“四八”烈士陵园 ——记盱眙籍革命烈士毛培春（293）
23. 四八烈士陵园祭扫活动（104）
24. 青协大事件 | 缅怀先烈，祭扫“四八”烈士陵园（164）
25. 延安四八烈士陵园管理处向全社会征集烈士史料及遗物（620）
26. 史志故事】“四八”烈士遇难后的飞机残片（38）
27. 四八烈士陵园（13）
28. 【1077最关注】延安四八烈士陵园关于征集烈士史料和遗物的启事（21）
29. 【红典心声】四八烈士陵园里的不朽英魂（55）
30. 体育学院组织学生祭扫“四八”烈士陵园接受爱国主义教育（96）
31. 学四史，守初心 | 中文19-3“追寻先烈足迹，践行党史精神”第二次实践活动之走进四八烈士陵园（193）
32. 清明节前赴四八烈士陵园缅怀先烈的人次再次突破新高（140）
33. 祭扫“四八烈士陵园”户外训练活动（788）
34. 广大市民请踊跃捐赠烈士史料和遗物 延安四八烈士陵园管理处公开征集（10）
35. 四八烈士陵园收到珍贵资料（27）
36. 【英雄礼赞——听我来讲英烈故事】 纪念烈士遇难72周年活动在“四八”烈士陵园举行（233）
37. 学院新闻 | 我院先进团员前往“四八”烈士陵园参加主题教育活动（349）
38. 我院党委组织特立班赴“四八”烈士陵园祭奠英烈（429）
39. 四八烈士遇难73周年纪念活动在延安四八烈士陵园隆重举行（420）
40. “缅怀革命先烈，寄托永恒追思”——我院学生走进四八烈士陵园（385）
41. 今天，让我们一起缅怀“四八”烈士！（19）
42. 缅怀先烈，不忘初心 ——洁能公司清明节祭扫“四八”烈士陵园（71）
43. 延安四八烈士陵园管理处公开征集烈士史料和遗物 ——广大市民请踊跃捐赠烈士史料和遗物（62）
44. 缅怀烈士、砥砺前行——我班走进四八烈士陵园（127）
45. 怎么学梁家河大学问才算深入？四八烈士陵园：“六学”！（87）
46. 市招商局党支部赴“四八”烈士陵园扫墓（86）
47. Momemts‖缅怀先烈，传承精神 教科院赴四八烈士陵园祭扫活动（209）
48. 我校师生清明前夕赴四八烈士陵园进行祭扫活动（152）
49. 未雨绸缪 英烈纪念馆（四八烈士陵园管理处）及早安排部署2017年工作（49）
50. 【缅怀】英雄的四八烈士（802）
51. 今天是“四八”烈士遇难75周年，王若飞、秦邦宪、叶挺、邓发、黄齐生等十三位烈士永垂不朽！（695）
52. 【党史中的安顺】今天是“四八”烈士遇难75周年，王若飞、秦邦宪、叶挺、邓发、黄齐生等十三位烈士永垂不朽！（85）
53. 延安王家坪革命旧址管理处 与延安“四八”烈士陵园管理处 举行“四八”烈士陵园祭扫活动（199）
54. 岗位作贡献 争先树典范 延安四八烈士陵园管理处党支部开展共产党员先锋岗评选活动（31）
55. 铁嘴侃新闻 | 徐绿山：新媒体延安行，没想到的收获（482）
56. “非热门系列”|“十一”出游， 这些地方人少（2014）
57. 王若飞“四八”烈士遇难记（566）
58. 一群大校军官的“课堂突围（1.1万）
59. “延安精神”小学段社会实践班祭奠四八革命先烈（158）
60. 推动退役军人工作高质量发展，延安这个会定了调子！（1.4万）
61. 坚守初心、锤炼党性、砥砺奋斗的一次成功实践 ——国防大学党史学习教育综述（4084）
62. 《光明日报》报道了国防大学这项工作......（9334）
63. 快讯：汪志斌晋升中将军衔，沈汉江晋升少将军衔（7689）
64. 反响强烈！文献纪录片《关向应在晋绥》上线获好评！（4492）
65. 金普融媒倾情力作！文献纪录片《关向应在晋绥》正式上线（7097）
66. 十八局红色“大片”，重磅来袭！你值得拥有！（6773）
67. 党史百年故事会 | 叶挺：在烈火与热血中永生（72）
68. 党史100年 · 天天读 （95）| 叶挺：烈火热血铸名将（181）
69. 喜迎二十大 | 团干部思政技能大比武基层团支部书记专项赛校内选拔赛精彩回顾!（657）
70. 在十九大持续热议的背后……（410）
71. 汪志斌少将出任81集团军政委，带队前往梁家河（3.5万）
72. “宁”听党史 | 叶挺：烈火热血铸名将（94）
73. 书画蜗牛漫游记 （654）歙县 徽州古城（5） 叶挺将军囚禁处（1）
74. 现代文阅读训练410：散文阅读《​清凉境界》｜每天10分钟，满分很轻松（532）
75. 传承红色文化，弘扬延安精神（一）（1047）
76. 【幸福一小 • 百年党史天天学】悟思想 •6月23日（168）
77. “北伐名将”被囚5年多，出狱即申请重新入党（2万）
78. 奋斗百年路 启航新征程·数风流人物｜叶挺：烈火热血铸名将（83）
79. 【数风流人物】叶挺：烈火热血铸名将（543）
80. 掖县第一任县委书记！（7417）
81. 专访开国少将王政柱之子：叶挺未能回延安，是父亲心中抹不去的痛（2.5万）
82. 【老干部工作】扬州市开展“寻根问初心”党性教育培训活动（73）
83. 红旗漫卷——陕西革命旧址云上展之十六：延安四八烈士陵园（144）
84. 微党课 | 延安“四八”烈士陵园（317）
85. 红旗漫卷 || 陕西革命旧址云上展十二：延安四八烈士陵园（255）
86. 【汉中蜀道石刻艺术博物馆】党史知识:陕西革命旧址云上展：延安“四八”烈士陵园！（13）
87. 缅怀革命先烈 传承红色基因——延安四八烈士陵园宣传片（377）
88. 陕西革命旧址云上展（十六）| 延安四八烈士陵园（27）
89. 赓续英烈精神 汲取奋进力量——延安四八烈士陵园清明期间祭扫实录（95）
90. 延安市“四•八”烈士陵园（309）
91. 红旗漫卷——陕西革命旧址云上展（延安四八烈士陵园（24
92. 【党史学习·初心之地】陕西革命旧址云上展：延安“四八”烈士陵园(（28）
93. 陕西革命旧址(七):延安四八烈士陵园（45）
94. 延安旅游景点（四八烈士陵园）（20）
95. 踏着春风，一群“诚朴”少年走进延安“四八”烈士陵园……（3730）
96. 永垂不朽的丰碑—延安“四八”烈士陵园（164）
97. 缅怀革命先烈 追寻红色记忆|清华附中延安学校四八烈士陵园追思活动（1646）
98. 党史学习 ——初心之地|延安“四八”烈士陵园（35）
99. 缅怀革命先烈 追寻红色记忆-延安培植小学四八烈士陵园祭扫活动（1641）
100. 用指尖传递哀思 延安四八烈士陵园倡议“云”祭扫（411）
101. 延安四八烈士陵园简介（140）
102. 延安“四八”烈士陵园周边环境整治工程进展顺利（803）
103. 忠魂永驻，延安“四八”烈士陵园（18）
104. 【温馨提示】延安“四八”烈士陵园周边道路改造，请绕行！（373）
105. 延安四八烈士陵园庆祝建党95周年（48）
106. 百篇陕西红色经典|第七十七篇 “四八”纪念馆（214）
107. 党史学习教育 | 碑文敬读 ⑪ “四八”烈士纪念碑碑文敬读（179）
108. 为有牺牲多壮志（27）（4）
109. 【延岸良品】烈士陵园祭英雄，烈士精神传万代（549）
110. 【陕建古建园林】铭记历史|延安“四八”烈士陵园项目部在行动（297）
111. 我有一个故乡叫延安（564）
112. 延安四八烈士陵园 5.18国际博物馆日（48）
113. 【延安专辑】“四八”烈士陵园（50）
114. 【红色延安】现场教学之延安四八烈士陵园（82）
115. 延安四八烈士陵园开展“世界文化遗产日”活动（23）
116. 【987今日关注】“四八”烈士陵园周边环境整治工程 建设中彰显“延安速度”（484）
117. 清明节就要到了，一群学生走进了“四八”烈士陵园……（3624）
118. 疫情防控 | 注意啦！延安“四八”烈士陵园周边道路改造，请绕行！（320）
119. 因为改造施工，后天起四八烈士陵园将暂停对外开放！（149）
120. 公司党员干部瞻仰“四·八”烈士陵园（675）
121. 缅怀先烈，祭扫“四八”烈士陵园（74）
122. 【1077最关注】“四八”烈士陵园周边环境整治工程 建设中彰显“延安速度”（306）
123. 延安12345 | 广大市民请踊跃捐赠烈士史料和遗物 延安四八烈士陵园管理处公开征集（149）
124. 为了他，一群延安学生走进了“四八”烈士陵园！（3280）
125. 注意啦！延安“四八”烈士陵园周边道路改造，请绕行！（503）
126. 延安12345 | 注意啦！延安“四八”烈士陵园周边道路改造，请绕行！(（1224）
127. 延安王家坪革命旧址管理处开展祭奠“四·八烈士遇难日”主题纪念活动（282）
128. “四八”烈士陵园周边道路改造，路咋走看公告！（168）
129. 延安四八烈士陵园 开展“岁月回音壁——延安红故事讲台”暨庆祝建党95周年、“两学一做” 学习教育活动（252）
130. “传承红色基因，弘扬延安精神”研修班一行参观“四八”烈士陵园，缅怀先烈（105）
131. 四八烈士陵园扫墓活动（897）
132. 活力基层 | 我校数计学院举行党委组织赴延安“四八”烈士陵园缅怀革命先烈的活动（109）
133. 缅怀革命英烈 传承红色基因 ——延安市新区第一中学赴“四八烈士陵园”扫墓（2726）
134. 缅怀四八烈士 传承红色基因|延安革命纪念馆赴四八烈士陵园开展主题活动（301）
135. 【环境·暑期社会实践】念革命先烈 ,向知青看齐——走访延安知青纪念馆及“四八”烈士陵园（105）
136. 未雨绸缪 英烈纪念馆（四八烈士陵园管理处）安排部署2017年工作（42）
137. 英雄丰碑——中华民族最闪亮的坐标（1878）
138. 延安市各级少先队组织开展以“红领巾心向党”为主题的清明祭英烈活动（782）
139. 寻梦延安（6）（74）
140. 延安职业技术学院爱国主义教育基地揭牌仪式在延安四八烈士陵园举行（311）
141. 清明祭扫 | 公司党委组织党员干部赴延安四八烈士陵园祭奠英烈（576）
142. 关于延安“四八”烈士陵园周边环境整治工程（“四八”广场）道路改造连接临时调整交通组织的公告（2375）
143. 延安:关于“四八”烈士陵园周边环境整治工程（“四八”广场）道路改造连接临时调整交通组织的公告（977）
144. 缅怀革命先烈 传承红色基因 延安革命纪念地管理局举行纪念“四·八”烈士遇难75周年公祭仪式（15）
145. 延安四八烈士陵园管理处党支部开展“牢记初心使命、我为党旗添彩"主题实践活动（231）
146. 缅怀先烈，铭记党史—陕西省延安体育运动学校在“四八”烈士陵园举行党史学习教育活动（24）
147. 昨天，延安育才红军小学的孩子们走进了“四八”烈士陵园！（1924）
148. 缅怀革命先烈 传承红色基因 延安市举行纪念“四·八”烈士遇难75周年公祭仪式（469）
149. 延安市财政局团工委在“四八”烈士陵园举行祭奠革命英烈活动（416）
150. 一束束小白花，寄托着孩子们的无尽哀思……（1908）
151. 【延安事】延安“四八”烈士陵园周边环境整治工程进展顺利（14）
152. “书记小喇叭”赴圣地延安传承红色精神实践团顺利抵达第六站：四八烈士陵园（243）
153. 英雄 也需要聆听（138）
154. 扩散！延安“四八”烈士陵园周边道路改造，这些路段请绕行！（61）
155. 数计学院党委组织师生赴延安“四八”烈士陵园缅怀革命先烈（4）
156. 延安职业技术学院革命传统教育基地揭牌仪式在延安四八烈士陵园纪念碑广场举行（956）
157. 新闻 | 延安大学组织师生祭扫“四八”烈士陵园（2886）
158. 【基层党建】我校生命科学学院组织党员师生祭扫延安“四八” 烈士陵园（151）
159. 缅怀先烈 砥砺前行（122）
160. 延安四八烈士陵园 召开“两学一做”学习教育工作动员大会（25）
161. 缅怀先烈 铭记历史|宝塔区妇女联合会赴延安“四八”烈士陵园开展主题党日活动（196）
162. 生科院组织党员师生祭扫延安“四八” 烈士陵园（465）
163. “传承红色基因 缅怀革命烈士”红色经典诵读会在延安“四八”烈士陵园举行（230）
164. 暑期社会实践｜​“书记小喇叭”赴圣地延安传承红色精神实践团 顺利抵达第六站：四八烈士陵园（34）
165. 蔓啦格美尚会所--延安四八烈士陵园扫墓活动（14）
166. 陕西23处红色旅游热门目的地 胜利小长假就去这里吧！（2811）
167. 缅怀革命先烈 弘扬爱国精神---延安王家坪革命旧址管理处赴四八烈士陵园开展主题公祭活动（100）
168. 秦邦宪孙女秦红向四八烈士陵园捐赠珍贵资料（137）
169. 陕甘宁边区革命英烈纪念馆（延安四八烈士陵园管理处)召开创建全国文明城市工作推进会（86）
170. 延安四八烈士陵园管理处党支部召开“牢记初心使命、我为党旗添彩”主题实践活动动员会（141）
171. 2018年清明“祭奠英烈，共筑中华魂”主题纪念活动在延安四八烈士陵园举行（91）
172. 延安水文局组织职工赴四八烈士陵园祭奠革命先烈传承红色精神（51）
173. 提升学习效果 提高理论水平 延安四八烈士陵园管理处党支部开展党员干部轮流讲党课活动（40）
174. 清明节丨缅怀为铭记，更为传承（2593）
175. 延安四八烈士陵园管理处党支部开展“悟初心、守初心、践初心”主题党日活动（48）
176. 延安市宝塔区杜甫川小学少先队员赴“四八”烈士陵园接受爱国主义教育（209）
177. 瞻精神丰碑，走学习之路 | 延安分队参观“四•八”烈士陵（54）
178. 延安实验小学2021“缅怀革命先烈 传承红色基因” 清明活动（1073）
179. 青年三下乡丨访革命遗址 受思想洗礼 学延安精神（87）
180. 青春向延安，大骨情谊记心间（2879）
181. 陕甘宁边区革命英烈纪念馆（延安四八烈士陵园管理处）召开春节收心会（62）
182. 延安市公证处组织党员干部前往“四•八”烈士陵园举行清明祭奠活动（21）
183. 延安四八烈士陵园管理处党支部召开党的**十九大精神研讨会（85）**
184. 《延安·延安》第二十六集 四八烈士 虽死犹荣（6）
185. 解放思想大讨论丨红岛经济区举办社区“两委”干部示范培训班（478）
186. 传承延安精神 恪守职业使命——广州市律师行业党委党性教育延安培训班圆满结业（241）
187. 1946年：“四八空难”，中共代表团乘飞机离开重庆前往延安向中共中央汇报途中在山西省兴县黑茶山失事，机上所有人全部罹难。（882）
188. 延安职业技术学院附属小学简介（2581）
189. 那双罪恶的黑手：1946年重庆谈判后的中共遭遇“四八”空难的真相（1748）
190. 宣医新闻|宣武医院党委对支部书记开展党性修养培训（1349）
191. 清廉金融文化建设宣传月 | 中邮保险广东分公司积极开展清廉金融文化宣传月宣传工作……（192）
192. 【学习培训】我镇举办党务干部专题培训班（672）
193. 菏泽仲裁办赴延安开展“不忘初心、牢记使命”主题教育培训（230）
194. 【党性】兰德培训中心井冈山培训心得（659）
195. 追寻革命足迹 传承延安精神 ——延安中学举办红色远足研学旅行活动（2419）
196. 学习心得 | 岭南所党支部延续延安精神，坚守职业精神（178）
197. 「美师讲习所」第四期在延安顺利举办（1510）
198. 【梦想青实践】2014年暑期社会实践之优秀团队是怎么炼成的（三）（12）
199. 《延安·延安》四八烈士虽死犹荣（53）
200. 《延安·延安》丨第二十六集 四八烈士 虽死犹荣（2）
201. 两学一做 | 法院英模展播 • 王玉珍：发扬延安精神 做合格党员（1010）
202. 清华大学附属中学延安学校高中宣传介绍（1.5万）
203. 迪庆州总工会举办专兼职工会干部能力素质提升延安培训班（1346）
204. Moments|“四八”烈士陵园扫墓公祭活动（111）
205. 《陕西红色旅游》考试重点（289）
206. 王鹏：继承发扬延安精神 做好基层党建工作（基层书记赴延安培训心得连载之二）（553）
207. 沐着春风，职院附小数百孩子走进“四八”烈士陵园（1388）
208. 培训研修丨学校举办思政骨干赴延安“传承红色基因，争当育人先锋”专题培训班（总第六期（1942）
209. 聚焦 | 将军为何带队千里寻（2036）
210. 市儿童医院党委组织党员赴延安开展“不忘初心 牢记使命”党日活动（1327）
211. 记录丨红色传人寻根之旅——空军工程大学组织2014级学员赴延安（2221）
212. 【培训动态】河池学院中层干部综合素质能力提升培训班在延安大学泽东干部学院顺利举办（52）
213. 延安行（图/文）（615）
214. 初心固本 信念铸魂（1147）
215. 我的国庆假日——陕北自驾游（170）
216. 【弘扬延安精神 传承红色基因 】我包头职业技术学院共青团团干部辅导员暑期延安培训圆满结束（226）
217. 共青团延安市委开展清明节祭奠革命烈士活动（2406）
218. 时局关注 | 安徽等12位新任省委书记 “第一把火”怎么烧？（图）（3480）
219. 锡山2018年中青班精华集锦——锤炼奋勇生力军 打造优选理想地（2386）
220. 第71集•骑跨嘉陵拜老总（118）
221. 第81集团军首长机关回望圣地延安，追寻共产党人的“精神密码”（5900）
222. 延安之恋//马宏茂（857）
223. 【青马风采】青马延安行 学员谈感触（三）（321）
224. 首发！您收到一条来自延大国护队的消息，请查收（1119）
225. 玉屏政协委员赴延安进行专题培训（2136）
226. 【微新闻】荥阳市举办“守初心 尽职责 担使命”驻村第一书记延安专题培训班（1194）
227. “四八”烈士陵园，育才红军小学孩子们的清明祭！（1067）
228. 【延安路·青春行】京津冀沪宁晋川交通职教集团联盟高校师生红色之旅走进延安（744）
229. 《山河岁月》| 第十五集、第十六集|【奋斗百年路 启航新征程·数风流人物】叶挺 左权 彭雪枫 罗炳辉（42）
230. 学习心得 | 将延安精神融入到律师工作中（483）
231. 曹谷溪：心中的图腾／《延安，延安》序（262）
232. 【学习时报】党课里的故事 故事里的初心——中央党校报刊社的一次党课（4061）
233. 革命先烈的诗(2)《囚歌》作者 叶挺（924）
234. 历史人物简介！叶挺（982）
235. 党支部书记们到延安培训，他们都学了些啥？（1481）
236. 故乡行（375）
237. 【政工影像 】一次延安行， 一生延安情！（949）
238. 全国红色教育基地党性教育（685）
239. 山西人应该记住七十年前的今天……（273）
240. 黄陵服务队 | 十月工作总结（439）
241. 党史学习手册：“北伐名将”被囚5年多，出狱即申请重新入党（21）
242. 交院印象【第一百三十一期】（922）
243. 数风流人物｜叶挺：烈火热血铸名将（7）
244. 党史学习教育丨“学党史、忆初心、担使命”—我院组织党员干部赴延安开展红色教育培训（1785）
245. 旅游摄影004——关于红色旅游我有话要说（12）
246. 奋斗百年路 启航新征程·数风流人物 | 杨靖宇 | 赵一曼 | 叶挺 | 左权（28）
247. 【奋斗百年路 启航新征程·数风流人物】叶挺 左权 ​彭雪枫 罗炳辉（53）
248. 喜迎党的二十大 | 延安大学党建创新成果展（二十二）（134）
249. 【馆长数家珍】（第九十八期）毛培春：潜伏兰州入军统 谍海无涯建奇功（三）（49）
250. 百合师生到四八烈士陵园祭奠英烈（）（488）
251. 【学院新闻】学院56名师生分赴延安保定两地开展红色教育（804）
252. 宝塔山下回望初心，延水河畔再次出发！（336）
253. 【要闻】江苏省人防办赴延安组织“学习党史、寻梦初心”主题教育培训（470）
254. 奋斗百年路 启航新征程·数风流人物丨叶挺：烈火热血铸名将（128）
255. 纪念关向应同志原创诗歌征集大赛圆满落幕 | 附获奖名单（1920）
256. 【建党一百周年作品展·2021年第34期】韩惠民：百年峥嵘路 秦声愈昂扬（65）
257. 党史天天读 | “北伐名将”被囚5年多，出狱即申请重新入党（297）
258. ​【荟悦读·数风流人物】叶挺：烈火热血铸名将（189）
259. 延安革命纪念馆干部教育培训中心红色教育邀请函（2034）
260. 党史学习教育 | “北伐名将”被囚5年多，出狱即申请重新入党（148）
261. 【学党史·正青春】“北伐名将”被囚5年多，出狱即申请重新入党（142）
262. 又到清明 文明祭奠寄哀思（229）
263. 【党史中的安顺】今天是“四八”烈士遇难75周年，王若飞、秦邦宪、叶挺、邓发、黄齐生等十三位烈士永垂不朽！（42）
264. 贺子珍离家出走后，毛主席继续照顾岳母，一直到她去世（387）
265. 三下乡专刊 | 感悟延安革命精神，争做新时代重邮人（615）
266. 【青马风采】青马延安行 学员谈感触（二）（357）
267. 【奋斗百年路 启航新征程·数风流人物】叶挺 左权（31）
268. 【延安归来】信仰在招手（160）
269. 【党史学习】奋斗百年路 启航新征程·数风流人物之叶挺：烈火热血铸名将（18）
270. 今天，来省内这些红色景区重温历史吧！（306）
271. 9月3天小长假去哪儿？陕西23处红色旅游热门目的地任你选！（1631）
272. 延安四八烈士陵园管理处党支部开展“不忘初心跟党走，牢记使命勇争先”主题党日活动（25）
273. 延安革命纪念地管理局局长薛耀军深入延安四八烈士陵园调研指导工作（207）
274. “传承红色基因 缅怀革命烈士”纪念活动在延安“四八”烈士陵园举行（48）
275. 缅怀先烈 励志远足丨宝塔区第七中学第一届红色研学活动（1502）
276. 全国专项｜江苏大学能动学院团队参加全国大学生延安暑期社会实践专项活动！（1045）
277. 延安职业技术学院师生代表到“四八”烈士陵园缅怀先烈（588）
278. 教育实验学院——寻找红色记忆 践行核心价值（1027）
279. 缅怀革命先烈，弘扬爱国精神——延安革命纪念馆党委赴四八烈士陵园开展主题党日活动（31）
280. 蔓啦格形象设计有限公司及旗下品牌缔一国际全体员工---延安四八烈士陵园扫墓活动（338）
281. |青生活| 9月3天小长假去哪儿？陕西23处红色旅游热门目的地任你选！（104）
282. 【党课纪实】我校文学与新闻传播学院组织第52期入党积极分子赴四八烈士陵园举行“缅怀革命先烈，寄托永恒追思”祭扫活动（152）
283. 走进延安 | 楚博霖党支部在”四八”烈士陵园向革命烈士敬献花篮（100）
284. 陕甘宁边区革命英烈纪念馆 延安四八烈士陵园管理处召开“奋力追赶超越 提升陵园形象”座谈研讨会（44）
285. 永远跟党走：国网追寻红色足迹 汲取力量源泉（93）
286. 我院在“四八”烈士陵园举行党史学习教育活动（315）
287. 陕甘宁边区革命英烈纪念馆 （延安四八烈士陵园管理处） 积极备战局系统首届讲解员演讲比赛（80）
288. 天灾还是人祸？1946年“四八”空难真相追述（1106）
289. 分享 | 魂牵延安 逐梦前行（200）
290. 三下乡|江西师大大学生宣讲团赴延安四八烈士陵园缅怀革命先烈（171）
291. 缅怀革命先烈 传承革命薪火（139）
292. 建团百年丨党旗引领团旗 红色点亮青春（198）
293. 陕甘宁边区革命英烈纪念馆（延安四八烈士陵园管理处）党支部组织全体党员开展“党日活动进书院”主题教育活动（65）
294. 【致敬·缅怀·奋进】陕西省延安市消防救援支队组织开展“人民不会忘记”清明节特殊党课（1417）
295. “踏寻红色足迹，学习延安精神，践行大爱魂”康亦健慈善公益行（4028）
296. 【西散原创 • 初语阅读】梅雨墨作品 | 这片海，汹涌澎湃（1952）
297. 缅怀先烈 致敬英雄 陕西各地开展线上线下清明祭英烈系列活动（3249）
298. 延安革命纪念馆开展“诵读英烈故事”主题纪念活动——以国家之名悼念，以历史之名缅怀，以红色记忆讲述英烈故事（298）
299. 喜报！洛川会议纪念馆被命名为陕西省青少年教育基地！（26）
300. 嘀嗒动态|弘扬延安精神，我们在路上（947）
301. 青年头条 | 我校组织开展“传承·2019清明祭英烈”主题教育活动（627）
302. 延安市退役军人事务工作动态 （8.1-8.7）（442）
303. 三下乡 | 机电工程学院赴延安一带一路老革命催化新经济暑期社会实践团——参观四八陵园，缅怀革命先辈（193）
304. 清明期间延安少先队组织开展“红领巾心向党”学习实践活动（860）
305. 喜报！我市20家单位被命名为陕西省青少年教育基地！（1918）
306. 延安亲亲：2019金秋团建——“我与祖国同行”（605）
307. 熹微晨光中，一群延安学子踏上了红色远足的征程！ ​（4273）
308. 文院党建 | 党员先锋岗：学党史 祭英烈—文学院组织第57期入党积极分子开展党史学习教育活动（768）
309. 安康军休中心开展“喜迎二十大 感悟延安精神 牢记初心使命” 主题党日活动（360）
310. 包头这座建于民国的客栈，曾上演现实版的“潜伏”（4468）
311. 【连载】盱城老北头||敌营孤胆毛培春（605）
312. 解码中共“后备干部”（4.8万）
313. 喜讯 | 我校“校团委党史学习实践团”荣获全国暑期“三下乡”社会实践活动优秀团队（799）
314. 【党史专栏】六张图，重温烈士笔记（339）
315. 分享 | 重新学习《老三篇》 牢记使命不懈怠（453）
316. 青·行 | E路先锋——访延安风土民俗，寻建党百年征程（164）
317. 红色延安｜如沐春风 学员心里话（二）（336）
318. 孙绍骋在陕西调研退役军人工作（6.4万）
319. 【我在定远学党史】六张图，重温烈士笔记（267）
320. 【土木工程（建筑）学院简报第15期】追忆革命英烈，继承革命精神（75）
321. 2021第二期党组织书记、党务工作者培训班简报（四）（563）
322. 建筑工人“永远跟党走”主题宣传教育活动在延安市举行（175）
323. 陕西人都来这里旅游打卡了！陕西“红色旅游”受热捧（2692）
324. 民政部部长李立国率慰问团赴我市慰问红军老战士（925）
325. 叶挺：烈火热血铸名将（3704）
326. 六张图，重温烈士笔记（105）
327. 记录 | 岁月沧桑——哈尔滨历史日历（8月8日）（23）
328. 学党史 铸警魂丨叶挺：烈火热血铸名将（219）
329. 深度揭秘“后备干部”: 80%以上被埋没在名册里（8681）
330. 齐向┃走进延安——共产党人的精神家园侧记（441）
331. 清明节，师生们这样致敬英雄！（1.1万）
332. 孙绍骋在陕西调研，关注自主干部就业、退役军人培训等工作（2304）
333. 缅怀革命先烈告慰革命英灵 传承红色基因奋力追赶超越 延安市举行2019年烈士纪念日公祭仪式（643）
334. 深度揭秘中共选拔接班人的制度设计（3227）
335. 今日油田快讯（1226）
336. 喜迎团代会·基层团组织工作巡礼——艺术教育学院团总支（270）
337. 重温延安革命情 不忘初心向前行---玉溪市公安局举办基层党组织书记（党务干部）延安培训班（305）
338. 八月，他们奔赴延安收获满满……（3227）
339. 曹光哲｜一年飞越17省 在历史场景中寻找“人间正道”（193）
340. 【培训心得】秦志杰：让延安精神成为激励工作的强大动力（749）
341. 青年精神素养提升工程 | 宝塔山下学延安精神 梁家河畔悟初心使命（935）
342. 中心介绍丨陕西省当代红色文化培训教育中心欢迎您的到来（16）
343. 国庆献礼｜郑乃谦：心灵洗礼延水畔（624）
344. 老干部延安学习之行.心得体会（437）
345. 祭奠英烈 | 延安大学在清明上了这样一堂党史学习课（899）
346. 历史揭秘：叶挺的后人现在过得怎么样？（211）
347. 久违的感动—延安之路（6）
348. 小艺的延安行（134）
349. **视频：100**

**清凉山革命旧址**

1. 延安清凉山革命旧址窑洞活化利用（158）
2. 讲好革命文物故事 引导青年“扣好人生中的第一粒扣子”（9）
3. 陕西革命旧址(八):清凉山旧址（22）
4. 延安，一座信仰的城（604）
5. 清凉山革命旧址文物“活化”利用纪实（133）
6. 陕北行2020.9——（7）红色延安（21）
7. 【党史知识】陕西革命旧址云上展：清凉山新闻出版部门旧址（1674）
8. 陕西革命旧址云上展（六） | 清凉山新闻出版部门旧址（131）
9. 重磅 | 延安清凉山革命旧址红培教育营地窑洞活化利用走上国外主流媒体（332）
10. 红旗漫卷——陕西革命旧址云上展（六）（191）
11. 延安之旅——清凉山（25）
12. 一波美图、视频，让你看看延安清凉山革命旧址红培教育营地的研学多热闹（112）
13. 不忘初心跟党走，红色精神代代传 ——内蒙古志愿者走进延安革命圣地之三：清凉山（353）
14. 清凉山革命旧址红培教育营地的昼与夜（68）
15. 革命旧址作“教室”，文物史料成“教材”（107）
16. 【检察文苑】延安党史之旅——清凉山（151）
17. 孩儿们的乐园——清凉山革命旧址红培教育营地（275）
18. 延安“红色之旅”来了，一起去看宝塔山！（5599）
19. 【学党史、守初心】延安党史之旅——清凉山篇（154）
20. 红旗漫卷 | 陕西革命旧址云上展——清凉山新闻出版社旧址（53）
21. 【学习卡片】全国爱国主义教育示范基地（陕西篇）：延安革命纪念地之清凉山新闻出版部门旧址（13）
22. 清凉山旧址（40）
23. 【宁品读】清凉山（482）
24. 凤栖原上没有凤凰，只有枫林尽染的美景（604）
25. 延安市延安革命遗址之清凉山新闻出版部门旧址（176）
26. 聚焦延安 | 红旗漫卷——陕西革命旧址云上展：清凉山新闻出版社旧址（45）
27. 红旗漫卷——陕西革命旧址云上展之六：清凉山新闻出版社旧址（13）
28. 国庆假期红色旅游持续火热！这些红色“打卡地”你都去过吗（1399）
29. 百年党史日日学 | 陕西革命旧址云上展：清凉山新闻出版部门旧址（64）
30. 延安精神永放光芒 ——追寻革命圣地延安的红色足迹（2241）
31. 清凉山革命旧址管理处又多了一个合作伙伴！（229）
32. “百年华诞，青春向党” ｜ 延安革命史学习第二站 ——延安清凉山、延安新闻纪念馆（279）
33. 两个“长庆油田革命传统教育基地”落户延安（954）
34. DAY.8 参观清凉山革命旧址，体会报刊上的延安精神（36）
35. 风景名胜：清凉山（221）
36. 红培动态 | 入住博物馆窑洞 接受沉浸式教育——一撕得星火游学来到延安清凉山革命旧址红培教育营地（200）
37. 雪后的延安革命旧址及各景区竟然可以这么美！（4801
38. 拥军爱民 清凉山革命旧址红培教育营地“军民合作社”启动服务（62）
39. 延安 ——清凉山（221）
40. 革命圣地—延安（107）
41. 清凉山丨每周一县·延安（552）
42. 我院与延安清凉山革命旧址管理处签订“清凉山革命旧址红培教育基地”共建合作协议（295）
43. 中国最美避暑胜地！震撼登场！！！（133）
44. 【延安记忆】万众瞩目清凉山（468）
45. 西游记之延安清凉山（1.3万）
46. 走进延安 感受中国革命精神标识（150）
47. 渭南市1700多名小学生来延安市清凉山革命旧址红培教育营地举行研学活动（128）
48. 门票、图片与学党史（379）
49. 溯源寻根传承延安精神（三）| 重返革命圣地 点亮延安记忆​（355）
50. 【红领·青春党建 】陕西红培清凉山革命旧址教育营地一行莅临中信蓝海延安分公司参观学习（86）
51. 站在清凉山上，俯瞰革命圣地延安，这个市区城建怎么样（4）
52. 【红色陕西】万众瞩目清凉山（181）
53. 党史学习 | 延安清凉山——红色新闻山（33）
54. “凝心聚力 你我同行”陕西北方民爆集团延安分公司在清凉山革命旧址营地开展主题党日活动和工会团建活动（73）
55. 兰州飞马摄影诗 清凉山（146）
56. 延安第一名胜清凉山（4）
57. 延安十大旅游景点-延安清凉山（179）
58. 延安革命纪念地管理局：打造全国规模最大的旧址窑洞群现场教学体验基地（825）
59. 【走进陕甘宁红色老区】第一站：宝塔山、清凉山、梁村、川口（68）
60. 春天在哪里？春天就在咱延安的各个旧址里！（661）
61. 延安清凉山——红色新闻山（49）
62. 从延安到北安⑫｜清凉山：没有硝烟的战场（650）
63. 盘点延安免费的革命旧址或纪念馆，朝圣不需花钱（1019）
64. 陕西延安+枣园革命旧址+杨家岭革命旧址+王家坪革命旧址+宝塔山+清凉山2日1晚私家团（7）
65. 【方志四川•红色文化】蓬州闲士 ‖ 延安掠影之七：延安清凉山（73）
66. 【高铁游记】忆抗战：延安红色旅游（三）（385）
67. 【延安之行】第六站——清凉山+新闻纪念馆（67）
68. 今天，黑大学子在延安点燃火炬！背后的故事好燃！（4767）
69. 第三站｜清凉山，延安新闻纪念馆，我们在路上！（226）
70. 延安清凉山掠影（301）
71. 延安+宝塔山+红秀《延安延安》4日3晚私家团【体悟延安精神·革命老区追忆】清凉山+杨家岭革命旧址+枣园革命旧址+甘泉雨岔大峡谷（8）
72. 追根溯源悟初心 | 黑龙江大学赴延安探访学校办学旧址并开展合作共建活动（3094）
73. 展览预告|我的延安——陕西画家写生展（4407）
74. 我到访过的纪念馆之三 | 延安新闻纪念馆（127）
75. 直播预告 | 中央军委俄文学校旧址落成开展仪式（6477）
76. 守护“红色财富”，保护利用好革命旧址，延安一直在努力（104）
77. 双城记（153）
78. 陕北游记（11）——延安清凉山（2014.9.5）（22）
79. 延安市社科联马主席一行来到清凉山红培营地走访调研（318）
80. 柚实践 | 传承红色经典，烈焰实践队赴延安革命旧址进行参观（119）
81. 【延安掠影续六】延安清凉山（142）
82. 薛耀军调研革命旧址窑洞活化利用（805）
83. 革命文物保护利用，来自陕西的行动与经验（224）
84. 天空之眼瞰延安：老区不老 再焕新颜（3149）
85. 中国新闻第一山——清凉山（161）
86. ​“建党百年，我学党史，我来讲”第八期：延安革命纪念馆（55）
87. 点赞延安 | 守护“红色财富”，保护利用好革命旧址，延安一直在努力（49）
88. 延安革命红色圣地——魅力宝塔山、凤凰山、清凉山（50）
89. 来延安一定要去清凉山（6）
90. 人大人回到延安！重走红色之路，精神代代相传（1.3万）
91. 革命圣地学党史 红色基因铸党魂（636）
92. 中央军委俄文学校旧址落成开展仪式！黑大学子在延安点燃火炬！（59）
93. “沉浸式”教学让党史学习教育走深走实走心（375）
94. 沉思 | 当年轻的央视记者走进清凉山……（1411）
95. 红培已为您规划一条学习最佳路线 请查收！（303）
96. 【夏令营】红色研学“延安”||庆建党百年——重走革命路（464）
97. 期待！延安老城区将打造成为中国革命博物馆城（1163）
98. 【重走长征路·再访陕甘宁】追寻延安红色遗迹，感受长征光辉历程！（313）
99. 清凉山上的苦与乐 | 参考消息90年史话⑩（9473）
100. 原来，你是这样的延安。（4284）
101. 品游荟丨游革命圣地陕西延安（24）
102. 社会实践｜【延安之行】第六站——清凉山+新闻纪念馆（35）
103. 荐读 | 清凉山上“万众瞩目”的奥秘所在（2051）
104. 【校外实践活动】“走近新中国的声音”——小小记者团走进延安新闻纪念馆开展红色教育主题实践活动（525）
105. 我在延安清凉山（709）
106. 红色延安之旅（21）
107. 延安清凉山景区道路出现“车费路霸”，相关部门称已经备案（4237）
108. 重温红色历程，传承革命精神（377）
109. 游遍陕西——延安地区（412）：清凉山（26）
110. 【新增8月团期】延安红色研学之旅——重走革命路【重磅礼遇】（177）
111. 马东坡深入清凉山等景区旧址调研督查（43）
112. 骄傲！长庆人镜头里的延安巨变（2626）
113. 如果你是新闻人，请到延安清凉山来充电吧！（350）
114. （54）第七节 西北地区抗战纪念地旅游补选（一）（8）
115. 延安革命纪念地管理局：决战决胜创建国家文明城市（541）
116. 擦亮延安的“金名片”（430）
117. 五一在陕西该怎么玩？一条微信帮你搞定！（732）
118. “延安时期的新闻出版事业”复旦开展（58
119. 鲜为人知：延安文艺座谈会发生的故事（202）
120. 延安：以实干推进党史学习教育见实（179）
121. 赓续红色血脉 锤炼政治品格 | 学校举办党史学习教育延安精神专题培训班（1299）
122. 万众瞩目清凉山（132）
123. 【菌菌帮推】暑期实践：瞻仰革命旧址，汲取精神力量 ——记武大红色文化旅游实践队赴延安寻访调研圆满结束（117）
124. 线路在手，寻梦延安！(50
125. 延安整风（180）
126. @马鞍山人，低至171元！春节白菜价机票来了！（317）
127. 【专题文章】导读：父母不要打扰正在做事的孩子（83）
128. 新华书店延安发祥地旧址在北京复建落成（86）
129. 【惠游陕西】黄土风情，圣地延安（二）（2017）
130. 本周就是五一小长假了！出行的小伙伴们来看看2017陕西景区门票价格大全！竟有这么多免费的！！（1735）
131. 2018端午节延安最佳旅游地，不热不堵还不是革命红（89）
132. 【总结一】“周恩来志愿服务班”走入拥有“至高荣誉”的延安杨家岭红军小学，载誉归来！（84）
133. 七七纪念日 | 广州画院：“北上”写生创作展（944）
134. 来一次“虚拟旅行”吧，1000多景区，免费看！（11）
135. ▶昨天延安清凉山，人山人海，你去了吗？（132）
136. 市区公交旅游专线开通啦（3317）
137. ▶今天的延安清凉山，人山人海...（138）
138. 延安“厕所革命”采访见闻（724）
139. 厂工会开展“守初心 聚合力”工会干部团建活动（318）
140. 学党史 悟思想 学业务 强本领——西安供电段党委举办专兼职党支部书记及党小组长骨干培训班（532）
141. 延安双修之绿道篇——延安市老城区绿道网络建设规划工作体会（3126）
142. 什么是延安精神？（5747）
143. 张锦秋：历史文化是城市的灵魂（6455）
144. 迎新特辑||延大的正确打开方式（521）
145. 今日的延安清凉山...（271）
146. 致敬英雄，感谢你为我们拼过命！延安市欢送557名抗疫英雄回家！（1657）
147. 学生第六党支部“万众瞩目清凉山——延安出版事业展”云参观 党日活动顺利进行（52）
148. 中共七大代表在延安公祭革命烈士（430）
149. 昨天延安清凉山，人山人海（1763）
150. 又上榜了！凤县被全国红色旅游点名了（1277）
151. 高校学子传承延安精神，勇担青春使命（6057）
152. 散文||延安精神照我前行（506）
153. ▶今天延安清凉山，人山人海，你去了吗？（969）
154. 旅行与美食 | 梨花风起正清明 游子寻春半出城（129）
155. 岳双才作品(132)高万兵的文化梦（307）
156. 《清凉山新闻写作训练营》学生习作（二）丨延安新华广播电台旧址寻踪——王皮湾（347）
157. 4月机票便宜到爆！坐飞机出发到这些地方，低至50元！（848）
158. 行程单1（17）
159. 延安赋（之一）（41）
160. ▶延安清凉山，人山人海，你去了吗？（34）
161. 市文化广电和旅游局参观《万众瞩目清凉山 ——延安时期的新闻出版事业》专题展览（195）
162. 话说延安“三座山”（28）
163. 改革 | 中国人民大学研究生会：寻访初心，践行改革（1738）
164. 尖叫！！包茂通车全程3130公里 ，一路高速景点太美了！（2.2万）
165. 【聚焦】新中国新闻出版事业的摇篮，有机会一定要转转！（123）
166. 当石库门“对画”宝塔山，红色基因与延安精神碰撞出全新艺术火花（437）
167. 延安市“易公交”智能出行明日上线，公交旅游专线和电话预约出租上新城已经开通（997）
168. 观枣园旧址（16）
169. 延安清凉山，十一黄金周好去处！（24）
170. 探寻法治初心 缅怀革命先烈（242）
171. 马东坡再次深入杨家岭革命旧址等旅游场所调研督查 任学明陪同（49）
172. 延安未调色的清凉山（5）
173. ​甘志伟：走近延安（48）
174. 从谁的青春走过（280）
175. 寻访革命“新闻山”，畅游“陕北好江南”，“书本”带你重温延安红色岁月（3044）
176. “潇湘文化杯”征文选登｜｜清风·清水·清凉山（10）
177. 革命旧址作“教室”，文物史料成“教材”（9）
178. 【红西路军战士胡有贵】后人向延安清凉山气象纪念馆赠送珍贵文物（64）
179. 革命文物保护利用，来自陕西的行动与经验（5）
180. 一个苹果告诉你的“延安”故事（239）
181. 延安的山山水水竟然如此之美（2975）
182. 延安清凉山突然间人山人海！震惊…（5223）
183. 魂牵梦绕！我们回来了！（4952）
184. 宝塔山下游延安（317）
185. 锵锵锵 | 手绘明信片上的大美延安，太攒劲啦！（514）
186. 【不忘初心牢记使命 寻根追梦三秦行】全媒体采访团走进延安新闻纪念馆（155）
187. 延安醉美舞台在哪里？来我清凉山红培营地！（302）
188. 巡礼延安凤凰山—2018沿黄走晋秦（11）（69）
189. 沿着高速看中国｜延安宝塔区：革命老区的人情味与“烟火气”（38）
190. "重走革命路——延安红色研学之旅”2019夏令营（139）
191. 微观实践 | 烈焰实践队赴延安开展探寻革命圣地调研活动（230）
192. 清凉山红培教育营地 军事夏令营 开营啦！（1121）
193. 延安清凉山——历来被称为延安第一名胜（20）
194. 新华书店总店简史 延安十年 （1937-1947）（675）
195. 成仿吾雕像在延安陕北公学旧址落成揭幕（313）
196. 独家 | 2019上半年陕西景区群雄逐鹿！口碑50强花落谁家？（4395）
197. 传承红色基因 寻访红色印记 (第一期)（3179）
198. 出游不扎堆！这才是“五一”游三秦的正确方式~（1184）
199. 兵马俑酒店地址西安怎么去壶口瀑布？西安壶口瀑布延安二日游｜到壶口瀑布旅游攻略（16）
200. 助力成长，梦想起航〡学员清凉山红培教育营地一日拓展活动（130）
201. 延安清凉山旅游攻略（130）
202. 你知道吗？新华书店80岁了！（1599）
203. 这些地方，我们想免费邀请全国的警察都过来看看（6878）
204. 柚实践 | 传承红色经典，体悟延安精神（187）
205. 延安新闻纪念馆（117）
206. 大漠雄鹰｜红色基因传承系列之 《延安行》（255）
207. 【茹心玉】陕西行系列（450）
208. 延安归来----革命圣地探访录（836）
209. 【延安之行】清凉山一行（46）
210. 学在深处，干在实处！（665）
211. 【实践在路上】改革开放四十年，翻天覆地延安城 ——“国安”暑期社会实践团队赴延安纪实（94）
212. 沿着特高压看中国 | 陕北之“红”——延安精神代代传，光明“搂定”宝塔山（1144）
213. 团团带你看｜从延安到北安⑱清凉山：没有硝烟的战场（9）
214. 靳诺书记一行赴延安调研（1068）
215. 心中有信仰，脚下有力量——陕北9日行（541）
216. “到延安去”（2948）
217. 参观革命圣地延安（240）
218. 毛泽东为新华书店三次题写招牌——历史细节中的百年红色出版（759）
219. 延安红色之行（七）：没有去爬的宝塔山和清凉山（17）
220. 走访革命圣地，学习延安精神 | 东方金诚（462）
221. 3、2、1，开始!中央电视台在清凉山设直播室向老外介绍长征（823）
222. 暑期社会实践 | 古道热肠延安行——红色文化革命精神薪火相传（472）
223. 【“庆祝建党100周年”征文】走进延安圣地，领略延安精神​/任欢欢（陕西）（散文）（382）
224. 延安第一名山一一清凉胜境清凉山之旅（64）
225. 赵安华：心向延安（167）
226. 17、革命圣地的智能配电系统（之二）（63）
227. 红色圣地延安（1291）
228. 我为祖国献石油，长庆安塞油田唱得格外动听（396）
229. 景点巡礼丨延安第一名胜——清凉山（60）
230. 第25期〔影像空间〕延安清凉山（二）（245）
231. 正式公布｜“丝绸之路·神奇西北100处摄影地” 推荐名单！宝鸡这几处入选其中！！（808）
232. 学思践悟 | 延安记忆的青春启示（434）
233. 延旅20年系列活动之 “千万钜惠游延安”（134）
234. 再过1400天，陕西这里将迎接全世界的目光！（3635）
235. 洛阳到周边各地公路里程表！太造了！赶紧收藏！（1266）
236. 记者节，让我们为自己喝彩（1.3万）
237. 系列：2021年的暑假——圣地（93）
238. 第23期〔影像空间〕延安清凉山（一）（323）
239. 每日名胜 | 陕西·延安清凉山新闻出版部门旧址（7）
240. 延安清凉山，人山人海现场（130）
241. 组图 | 缅怀高岗同志逝世66周年（4.5万）
242. 重要公告！清凉山对全国高考生免门票！（44）
243. 正式公布｜“丝绸之路·神奇西北100处摄影地” 推荐名单！看看有没有你心仪的地方！（2276）
244. 【汇动态】参观革命圣地 传承延安精神（187）
245. 【1077爱延安】清凉山景区~（80）
246. 为啥今天都在传延安清凉山？快来看看现场视频、图片（1133）
247. 2021年，西安将迎接全世界的目光（1.1万）
248. 参观延安革命旧址（67）
249. 【经典游】克拉玛依上榜“丝绸之路·神奇西北100处摄影地” 推荐名单（96）
250. 红色延安｜追寻革命圣地（94）
251. 书馆 | 当特色主题书店披上红装……（303）
252. 李富东陪同，步行清凉山。山门前俯瞰延安。（7）
253. 红色基因说 | 我们的延安现地教学（1410）
254. 「经典回顾」中国园林·建党100周年｜修复革命圣地的绿水青山——延安生态修复工作实践探索（613）
255. 五月的鲜花开遍了延安的原野——我的杨家岭采访本（1309）
256. 我与一座城 | 延安新貌（2268）
257. 延安印象（50）
258. 延安的四山————清凉山（18）
259. 推动革命文物保护利用 陕西这样做→（295）
260. 《家乡记》杜甫川小学 三年级五班（188）
261. 图闻｜87周岁，生日快乐！（1665）
262. 延安记（37）
263. 党员之声 | 化学与化工学院教工党支部举行“弘扬延安精神，培塑优良作风”主题党日活动（308）
264. 再过1400天，西安将迎接全世界的目光！（828）
265. 安排！“五一”小长假，陕西这些地方够你玩！（1201）
266. 开学第一课 || 萌新看这里（369）
267. 陕西延安清凉山新闻出版部门旧址（3）
268. 讲好革命文物故事 引导青年“扣好人生中的第一粒扣子”（22）
269. 革命圣地延安介绍）（473）
270. 你以为延安是黄土、窑洞，其实早就是生态绿地，现代城市！（880）
271. 十天，能走多远（461）
272. 五一去哪儿3 | 这20条全新推出的旅游线路 带你玩转陕西~（829）
273. 天呐！4月西安飞这些地方的机票便宜到爆！这个清明假期一起浪走（1923）
274. 《砥柱中流：伟大的敌后抗战》连载42 试问九州谁做主，万众瞩目清凉山！（73）
275. 天呐！3月西安飞这些地方的机票便宜到爆！最低50块~（64）
276. 延安市清凉山（28）
277. 延安：共产党的成长与壮大（4万）
278. 崛起“新延安”之一：延安清凉山上建新城（1804）
279. 高塘镇入选陕西 “100处红色旅游地”，名列渭南榜首（1013）
280. 【践行录】弘扬革命传统，践行延安精神（97）
281. 【健康新闻】不忘初心，牢记使命，永远奋斗 ——西安市红会医院部分党员参观延安革命纪念馆及杨家岭革命旧址（176）
282. “五一”去哪玩？ 来陕西就对了！（含自驾线路、景区安排、惠民福利）（1427）
283. 欢迎回家 | 致敬最可爱的人，子长57名“逆行者”凯旋归来！（5279）
284. **视频：**

**凤凰山革命旧址,**

1. 凤凰山革命旧址——忆峥嵘岁月，传延安精神（54）
2. 凤凰山革命旧址（196）
3. 一次延安行，一生延安情—凤凰山革命旧址（22）
4. 革命圣地延安-凤凰山（53）、
5. 延安凤凰山革命旧址（22）
6. 【红色堡垒·百年党史】红色地图 | 陕西革命旧址云上展：凤凰山革命旧址（43）
7. 延安市延安革命遗址之凤凰山麓革命旧址（51）
8. 延安革命旧址——凤凰山（49）
9. 游遍陕西——延安地区（406）：凤凰山革命旧址（24）
10. 【党史百年 · 天天读】1月13日（2108）
11. 延安凤凰山革命旧址疫情防控公（608）
12. 【西部风物】陕西：延安凤凰山麓革命旧址（10）
13. 红色记忆巡礼|河北爱国主义基地——凤凰山旧址（29）
14. 参观延安凤凰山革命旧址景区防疫须知（150）
15. 百年党史日日学 | 陕西革命旧址云上展：凤凰山革命旧址（96）
16. 红色印迹 | 中共中央进驻延安第一站——凤凰山革命旧址（6）
17. 延安凤凰山革命旧址（中国人民抗日军政大学纪念馆）恢复开放公告（473）
18. 心连心、共战“疫”——延安凤凰山革命旧址在行动（204）
19. 延安凤凰山革命旧址开展国庆节前安全大检查（90）
20. 畅游攻略（12）
21. 延安凤凰山革命旧址的另一种“开放”姿态（306）
22. 第七期|听党史学习实践团讲延安红色教育基地——凤凰山麓革命旧址（58）
23. “喜迎二十大 忠诚保平安” 延安凤凰山革命旧址开展反恐防暴应急演练（123）
24. 延安凤凰山革命旧址召开2021年度工作总结表彰大会（223）
25. VR全景旅游 | 红色地标—延安凤凰山革命旧址（114）
26. 延安凤凰山革命旧址管理处召开疫情防控工作会（67）
27. 延安凤凰山革命旧址迎来了一群大学生（150）
28. 延安凤凰山革命旧址开展第三季度消防安全教育培训（75）
29. 延安凤凰山革命旧址开展中秋节前安全大检查（68）
30. 延安凤凰山革命旧址管理处 关于调整讲解收费标准的公示（197）
31. 【延安革命遗址 四十七】凤凰山麓（6）
32. 延安精神：凤凰山革命旧址（62）
33. 延安凤凰山革命旧址举办庆三八趣味活动会（113）
34. 延安凤凰山革命旧址召开二十大期间安全防范工作会（73）
35. 延安凤凰山革命旧址清明节前安全大检查（115）
36. 延安凤凰山革命旧址宣教部：总结上半年 冲刺下半年（109）
37. 延安凤凰山革命旧址管理处开展疫情防控大检查（96）
38. “不忘初心 牢记使命”主题教育基地 | 凤凰山革命旧址（64）
39. 【延安专辑】凤凰山革命旧址（76）
40. 延安凤凰山革命旧址召开近期疫情防控工作部署会议（93）
41. 落实安全责任 推动安全发展—— 延安凤凰山革命旧址2021年安全生产月主题活动（148）
42. 十堰乘飞机出发，到革命圣地延安追寻红色记忆!（2322）
43. 延安凤凰山革命旧址管理处 关于旧址临时闭馆的通知（551）
44. 延安凤凰山革命旧址开展反有组织犯罪法培训活动（38）
45. 延安凤凰山革命旧址管理处党支部传达学习局党委中心组集体学习会议精神（70）
46. 【档案故事】纪念白求恩（216）
47. 延安凤凰山革命旧址开展冬季消防安全大检查（118）
48. 延安凤凰山革命旧址管理处党支部开展作风建设专题学习（48）
49. 延安凤凰山革命旧址管理处党支部开展 “敬老月”进社区慰问活动（81）
50. 延安凤凰山革命旧址管理处党支部开展纪律教育学习宣传月主题党日活动（105）
51. 延安凤凰山革命旧址管理处党支部传达学习局党委会议精神（85）、
52. 延安凤凰山革命旧址开展春节前安全大检查（119）
53. 延安凤凰山革命旧址开展端午节前安全检查（105）
54. 延安凤凰山革命旧址庆祝建党100周年开展进企业宣传活动（87）
55. 延安凤凰山革命旧址宣教部2020上半年工作回顾（242）
56. 学党史，延安凤凰山革命旧址这样开展（94）
57. 延安凤凰山革命旧址管理处组织党员干部集中收看党的二十大开幕式盛况（59）
58. 延安凤凰山革命旧址管理处党支部组织开展“喜迎二十大 永远跟党走 重走红大路 薪火永相传”主题党日活动（237）
59. 延安凤凰山革命旧址召开“五一”节前宣教工作部署会议（92）
60. 延安凤凰山革命旧址开展“青春五四”摄影比赛初选作品网络投票开始啦！（2552）
61. 延安凤凰山革命旧址 新冠肺炎防控 旧址恢复开放应急预案（263）
62. 延安凤凰山革命旧址开展再读经典著作之《论持久战》专题讲座（121）
63. 延安凤凰山革命旧址召开“五一”节前安全工作部署会（124）
64. 延安凤凰山革命旧址开展第一季度讲解员业务考核工作（208）
65. 延安凤凰山革命旧址管理处开展“五一”节前安全检查）（100）
66. 延安凤凰山革命旧址管理处党支部召开专题会议传达学习局党委第三次中心组会议精神（76）
67. 延安凤凰山革命旧址：平安春节疫情防控小贴士（59）
68. 延安凤凰山革命旧址管理处2021年讲解员等级评定考核（132）
69. 延安凤凰山革命旧址管理处党支部传达学习局党委第四次中心组学习会议精神（123）
70. 延安凤凰山革命旧址“我是小八路”暑期研学社教活动开营啦！（740）
71. 延安凤凰山革命旧址管理处党支部 积极开展“我为群众办实事”实践活动着力解决群众“急难愁盼”具体问题（239）
72. 延安凤凰山革命旧址开展“我是小八路”精品研学主题教育活动（278）
73. 延安凤凰山革命旧址开展第四季度消防知识培训暨消防演练活动（78）
74. 延安凤凰山革命旧址管理处党支部传达学习延安革命纪念地管理局党委（扩大）会议精神（88）
75. 昨天，祝福祖国活动在延安凤凰山旧址精彩上演！（380）
76. 延安凤凰山革命旧址管理处5·18国际博物馆日（54）
77. 延安凤凰山革命旧址管理处开展中秋节前安全大检查（77）
78. 延安凤凰山革命旧址开展第二季度安全知识培训会（90）
79. 抢抓新机遇，增创新优势，再创新辉煌­——延安凤凰山革命旧址召开2019年度总结表彰大会（158）
80. 学习党史 提升服务//延安凤凰山革命旧址开展讲解员考核活动（147）
81. 延安凤凰山革命旧址举行 白求恩精神研究专家、北京白求恩公益基金会名誉理事长李深清资料捐赠仪式（85）
82. 【方志四川•红色文化】蓬州闲士 ‖ 延安掠影之六：延安凤凰山（101）
83. 延安记忆 | 凤凰山革命旧址的那些人与事（302）
84. 延安：凤凰山麓革命旧址（254）
85. 延安凤凰山革命旧址管理处党支部传达学习局党委第五次中心组集体学习会议精神（90）
86. 延安凤凰山革命旧址管理处党支部 结合工作实际 将“我为群众办实事”推深做实（59）
87. 延安凤凰山革命旧址管理处召开“五一”节前安全部署会议（72）
88. 延安凤凰山革命旧址开展第三季度消防安全培训（72）
89. 延安凤凰山革命旧址管理处党支部 召开专题会议 重温习近平总书记来陕考察重要讲话（66）
90. 延安凤凰山革命旧址管理处党支部 开展“学党史 忆初心 强党性” 主题党日活动（199）
91. 延安凤凰山革命旧址管理处党支部召开专题会议深入学习贯彻党的十九届六中全会精神（87）
92. 延安凤凰山革命旧址开展第二季度“遵守安全生产法 当好第一责任人”主题消防知识讲座（82）
93. 延安凤凰山革命旧址恭贺新禧（147）
94. 延安凤凰山革命旧址防汛检查（98）
95. 查漏补缺 延安凤凰山革命旧址开展防汛安全隐患大排查（67）
96. 延安凤凰山革命旧址开展“消除事故隐患 筑牢安全防线”安全教育培训（69）
97. 凤凰山革命旧址聚集了一群“小八路” 他们在干啥？（618）
98. 宝塔区“小桔灯”延安故事宣讲团“党的故事我来讲”主题宣讲活动走进凤凰山革命旧址（283）
99. 延安凤凰山革命旧址宣教部专题学习习近平总书记来陕视察重要讲话（215）
100. 延安凤凰山革命旧址管理处党支部 赴南泥湾开展主题党日活动（96）
101. 延安凤凰山革命旧址举办“落实消防责任 防范安全风险”119消防演练（115）
102. 延安凤凰山革命旧址管理处组织召开民主生活会（156）
103. 延安凤凰山革命旧址管理处党支部举办 “学党史、悟思想、增党性” 党史学习教育“线上+线下” 交流活动（93）
104. 延安凤凰山革命旧址管理处党支部开展 “党员心声大家谈”活动（67）
105. 延安凤凰山革命旧址管理处举行消除安全隐患 筑牢安全防线 疫情防控暨消防安全演练（441）
106. 党史学习|红色印迹：陕西篇（三）凤凰山革命旧址（13）
107. 延安凤凰山革命旧址管理处坚决打赢疫情阻击战！（75）
108. 延安凤凰山革命旧址管理处党支部深入学习宣传贯彻习近平总书记来陕考察重要讲话精神（58）
109. 【红色延安】现场教学之凤凰山革命旧址（29）
110. 延安凤凰山革命旧址等单位走进甘泉县旅游开发集团有限公司庆元旦（30）
111. 延安凤凰山革命旧址管理处党支部联合宣教部开展“追思感恩祭英烈 缅怀先辈慰英魂”主题党日活动（237）
112. 延安凤凰山革命旧址 小小讲解员获奖啦！（269）
113. 延安凤凰山革命旧址开展“我学我讲”小小讲解员培训（106）
114. 延安凤凰山革命旧址走进延安大学附属医院红色宣讲（110）
115. 延安凤凰山革命旧址管理处党支部 开展庆祝建党99周年主题党日活动（203）
116. 延安凤凰山革命旧址管理处党支部开展“暖心义诊服务 守护职工健康” 诊疗活动（84）
117. 延安凤凰山革命旧址管理处党支部开展“清除杂草 美化环境”主题党日活动（81）
118. 延安凤凰山革命旧址首期“我是小八路”主题实践活动（13）
119. 延安凤凰山革命旧址管理处党支部 召开党史学习教育专题组织生活会
120. （67）
121. 【党史学习教育】市环科所参观凤凰山革命旧址开展主题党日活动（97）
122. 延安凤凰山革命旧址管理处党支部 开展党员“政治生日”活动（55）
123. 延安凤凰山革命旧址第一季度消防安全暨疫情防控知识培训（73）
124. 延安凤凰山革命旧址全体人员收看纪念抗美援朝70周年大会直播（202）
125. 延安凤凰山革命旧址管理处学习领会十九大会议精神（82）
126. 延安凤凰山革命旧址管理处党支部 召开落实意识形态工作责任制专题会议（67）
127. 延安凤凰山革命旧址“青春五四” 摄影比赛圆满落幕（123）
128. 内外兼修 有礼有仪 凤凰山革命旧址讲解员接待礼仪专项培训（29）
129. 延安凤凰山革命旧址老孙的故事（25）
130. 延安凤凰山革命旧址暑假举办腰鼓培训班活动（23）
131. 延安凤凰山革命旧址管理处闭馆通知（169）
132. 聚焦革命文物保护，带你一览延安革命遗址（上）（605）
133. 延安凤凰山革命旧址管理处党支部 开展党史学习教育应知应会知识测试（27）
134. 延安凤凰山革命旧址管理处召开“五一”假期安全防范部署会（93）
135. 关于延安凤凰山革命旧址临时关闭的通知（1317）
136. 延安凤凰山革命旧址管理处百日安全生产整治行动部署会（19）
137. 延安凤凰山革命旧址走进新区第一小学，共贺少年先锋队69周年诞辰（11）
138. 参观延安凤凰山革命纪念馆！怀念革命先烈！（21）
139. 防疫侧记』巾帼不让须眉—凤凰山革命旧址讲解员杨默（95）
140. 科普延安 | 凤凰山革命旧址文物小讲堂开讲了！（42）
141. 延安凤凰山革命旧址“我是小八路”研学活动 精彩瞬间（25）
142. “延安的窑洞有马列主义”（2.5万）
143. 延安凤凰山革命旧址党员及讲解员共赴新区书院参观学习（49）
144. 身边的感动 | 巾帼不让须眉—凤凰山革命旧址讲解员杨默（79）
145. 董世民一行督查指导凤凰山革命旧址安全保卫工作（1066）
146. 延安凤凰山革命旧址管理处党支部 组织全体党员干部参观“回望延安--中国共产党人的初心和使命”展览（42）
147. 【延安革命遗址 四十八】凤凰山麓防空洞（15）
148. 广安经开区党务干部党性修养提升班在延安凤凰山革命遗址、梁家河考察学习（312）
149. 延安凤凰山革命旧址管理处党支部组织 全体党员干部到延安革命纪念馆参观学习（195）
150. 延安凤凰山革命旧址管理处党支部开展“不忘初心、牢记使命”主题教育集体学习（78）
151. 延安凤凰山革命旧址管理处开展2016年度讲解员培训工作（82）
152. 为了“造一块好地”，浙江这样做（961）
153. 延安革命纪念地管理局董世民副局长一行 指导凤凰山革命旧址安全保卫工作（192）
154. 延安——枣园革命旧址（161）
155. 故事里的中国共产党------中国革命圣地延安（3249）
156. 延安革命纪念地管理局领导莅临凤凰山革命旧址检查指导工作（63）
157. 张守福 : 延安之“圣”——顺着宝塔山寻根追脉（2178）
158. 新闻汇｜春运将启！高速“免通”政策、利好因素来了→（2623）
159. 忆党史│党史故事：郭沫若为延安革命纪念馆题写的馆名（3）
160. 陕西命名一批青少年教育基地，多数为文博场馆，快来看看吧！（76）
161. ​西安博物院等131个单位被命名为陕西省青少年教育基地（621）
162. 陕西依托革命遗迹旧址 打造廉洁阵地培育精神家园（1918）
163. 习近平带领中共中央政治局常委瞻仰延安革命纪念地（9.1万）
164. 陕西命名一批青少年教育基地，多数为文博场馆（1664）
165. 企业占11个，研学基地有3个！2022年青少年教育基地名单公布！（619）
166. 点赞！镇安1家单位被命名为陕西省青少年教育基地（46）
167. 商洛7家单位被命名为陕西省青少年教育基地（83）
168. 喜报｜八路军西安办事处纪念馆入选“陕西青少年教育基地”（107）
169. 向着新的奋斗目标，出发！（10万+）
170. 1973年周恩来总理在延安（189）
171. 瞻仰延安革命纪念地（二）（13）
172. 喜报！邓宝珊将军纪念馆被命名为陕西省青少年教育基地（219）
173. 陕西命名一批青少年教育基地，榆林多个文博场馆上榜（471）
174. 参观凤凰山革命旧址（74）
175. ​韩城市薛峰水库建设事迹陈列馆被命名为陕西省青少年教育基地（54）
176. 点赞！商洛7家单位被命名为陕西省青少年教育基地（7611）
177. 陕西命名一批青少年教育基地，商洛这些单位入选！（156）
178. 习近平总书记带领中共中央政治局常委瞻仰延安革命纪念地（5016）
179. 新闻联播丨习近平带领中共中央政治局常委瞻仰延安革命纪念地（10万+）
180. 韩城市4个单位被命名为陕西省青少年教育基地（991）
181. 2022年延安市事业单位公开招聘(募)324人！三支一扶”计划60名（1.1万）
182. 中国纪检监察报头版报道了我省这项工作！（5600）
183. 习近平：红旗渠精神同延安精神一脉相承，永远震撼人心（1.2万）
184. 延 安 之 旅（883）
185. 习近平在瞻仰延安革命纪念地时的重要讲话金句（6320）
186. 党建|红旗渠精神同延安精神一脉相承，永远震撼人心（419）
187. 新华社 | 【第一观察·瞬间 】向着新的奋斗目标，出发！（975）
188. 弘扬延安精神 重温峥嵘岁月（521）
189. 郭沫若为延安革命纪念馆题写的馆名（158）
190. 党史青年说 | 向着新的奋斗目标，出发！（103）
191. 党政前沿|弘扬伟大建党精神和延安精神（46）
192. 安平发布｜向着新的奋斗目标，出发！（594）
193. 【每日益读】听总书记的话（12）
194. 学习宣传贯彻党的二十大精神 | 赵克红：《窑洞灯光》（525）
195. 【延安新故事3】习近平带领中共中央政治局常委赴陕西瞻仰延安革命纪念地（31）
196. 新一届中央领导集体赴延安，宣示了什么（4023）
197. 【学习正当时】领会重要言论 深入学习党章（255）
198. 每周一学 | 习近平在陕西延安和河南安阳考察讲话摘要（2781）
199. 习近平带领中共中央政治局常委赴陕西延安瞻仰延安革命纪念地（3.4万）
200. 学习二十大 永远跟党走｜向着新的奋斗目标，出发！（80）
201. 窑洞灯光 || 文/赵克红 诵/果子（112）
202. 先锋工程 | 我的延安故事（二十五）（81）
203. 团干部领学诵读二十大 | 第五期（256）
204. 团中央主题教育检视问题列清单；江西暴雨青年志愿者冲在救灾第一线丨共青团新闻联播（附微信、微博排行榜）（7.8万）
205. 【学习红旗渠精神】习近平：红旗渠精神同延安精神一脉相承，永远震撼人心（45）
206. 延安凤凰山（162）
207. 习近平：为实现党的二十大提出的目标任务而团结奋斗）（10）
208. 【党员学习】为实现党的二十大提出的目标任务而团结奋斗（2）
209. 弘扬伟大建党精神和延安精神 为实现党的二十大提出的目标任务而团结奋斗——桥外所政治理论学习第十期（45）
210. 【学习宣传贯彻二十大精神 静宜党建专栏】（三十五）习近平：红旗渠精神同延安精神一脉相承，永远震撼人心（66）
211. 关注丨习近平在瞻仰延安革命纪念地时强调 弘扬伟大建党精神和延安精神 为实现党的二十大提出的目标任务而团结奋斗（79）
212. 弘扬伟大建党精神和延安精神，为实现党的二十大提出的目标任务而团结奋斗（72）
213. 学习二十大 || 转载 |习近平在瞻仰延安革命纪念地时强调 弘扬伟大建党精神和延安精神 为实现党的二十大提出的目标任务而团结奋斗（39）
214. 【云德党建】习近平：弘扬伟大建党精神和延安精神 为实现党的二十大提出的目标任务而团结奋斗（47）
215. 【学“习”园地】习近平：红旗渠精神同延安精神一脉相承，永远震撼人心（3）
216. 【推荐阅读】习近平在瞻仰延安革命纪念地时强调 弘扬伟大建党精神和延安精神 为实现党的二十大提出的目标任务而团结奋斗（615）
217. 致公党十五届十六次中常会在延安召开​（2957）
218. 【时习之】弘扬伟大建党精神和延安精神 为实现党的二十大提出的目标任务而团结奋（184）
219. 【思想速递】习近平：红旗渠精神同延安精神一脉相承，永远震撼人心（25）
220. 【关注】习近平在瞻仰延安革命纪念地时强调 弘扬伟大建党精神和延安精神 为实现党的二十大提出的目标任务而团结奋斗（43）
221. “沂”起学理论丨大庄镇机关干部“讲故事 学理论”上讲台（13）
222. 登延安凤凰山（67）
223. 延安市各级团组织积极开展“喜迎二十大、永远跟党走、奋进新征程”主题教育实践活动（1554）
224. 习近平2022年10月27日在瞻仰延安革命纪念地时强调 弘扬伟大建党精神和延安精神 为实现党的二十大提出的目标任务而团结奋斗（204）
225. 【先学】 弘扬伟大建党精神和延安精神 为实现党的二十大提出的目标任务而团结奋（114）
226. 【学习贯彻党的二十大精神(21)】习近平：弘扬伟大建党精神和延安精神 为实现党的二十大提出的目标任务而团结奋斗（163
227. 【紫阳教育】习近平：红旗渠精神同延安精神一脉相承，永远震撼人心（109）
228. 习近平总书记：弘扬伟大建党精神和延安精神 为实现党的二十大提出的目标任务而团结奋斗（508）
229. <3819期>习近平在瞻仰延安革命纪念地时强调 弘扬伟大建党精神和延安精神 为实现党的二十大提出的目标任务而团结奋斗（146）
230. 习近平在瞻仰延安革命纪念地时强调 弘扬伟大建党精神和延安精神 为实现党的二十大提出的目标任务而团结奋（557）
231. 习语苏递 | 习近平在瞻仰延安革命纪念地时强调 弘扬伟大建党精神和延安精神 为实现党的二十大提出的目标任务而团结奋斗（320）
232. 习近平瞻仰延安革命纪念地时强调 弘扬伟大建党精神和延安精神 为实现党的二十大提出的目标任务而团结奋斗（590）
233. 微团课 | 习近平在瞻仰延安革命纪念地时强调 弘扬伟大建党精神和延安精神 为实现党的二十大提出的目标任务而团结奋斗（8）
234. 【学习宣讲】习近平在瞻仰延安革命纪念地时强调 弘扬伟大建党精神和延安精神 为实现党的二十大提出的目标任务而团结奋斗（10）
235. 踔厉奋发 聚焦二十大 谱写新征程​|数学与统计学院学习宣传贯彻党的二十大精神第十四期（85）
236. 习近平：红旗渠精神同延安精神一脉相承，永远震撼人心约精神（799）
237. 学习进行时 | 习近平：红旗渠精神同延安精神一脉相承，永远震撼人心（1493）
238. 集结号｜习近平：弘扬伟大建党精神和延安精神 为实现党的二十大提出的目标任务而团结奋斗（3055）
239. 精神的追寻｜“新思想引领新征程”——学习红旗渠精神系列（七）（52）
240. 【党建阵地】百年党史 第五十五期 弘扬伟大建党精神和延安精神 为实现党的二十大提出的目标任务而团结奋斗（528）
241. 关于认真学习《习近平在瞻仰延安革命纪念地时强调 弘扬伟大建党精神和延安精神 为实现党的二十大提出的目标任务而团结奋斗》的学习倡议（254）
242. 【学習新思想】习近平在瞻仰延安革命纪念地时强调，弘扬伟大建党精神和延安精神，为实现党的二十大提出的目标任务而团结奋斗（286）
243. 【理论学习】习近平：红旗渠精神同延安精神一脉相承，永远震撼人心（325）
244. 学习笔记丨总书记反复强调这“两种精神”，有何深意？（566）
245. 【专题学习】习近平总书记在瞻仰延安革命纪念地和河南安阳考察时重要讲话精神（1169）
246. 习近平在瞻仰延安革命纪念地时强调 弘扬伟大建党精神和延安精神 为实现党的二十大提出的目标任务而团结奋斗（10万+）
247. 【二十大时间】习近平：红旗渠精神同延安精神一脉相承，永远震撼人心（164）
248. 团干部领学诵读二十大 | 第五期（257）
249. 【党员教育】习近平在瞻仰延安革命纪念地时强调 弘扬伟大建党精神和延安精神 为实现党的二十大提出的目标任务而团结奋（99）
250. 多家主流媒体报道，一起围观！（3344）
251. 田野“思想号” | 学习宣传贯彻党的二十大精神，学原文悟原义——第十二讲：弘扬伟大建党精神，赓续红色血脉，夺取新胜利（68）
252. **视频：126**
253. 王家坪革命旧址
254. 红色印迹 | 走进王家坪革命旧址，追溯奋斗党史，传承延安精神（28）
255. 打卡廉政教育基地 | 陕西革命旧址云上展：延安王家坪革命旧址（19）
256. 王家坪革命旧址（51）
257. 红旗漫卷｜陕西革命旧址云上展——王家坪革命旧址（396）
258. 【党史知识】陕西革命旧址云上展：延安王家坪革命旧址（263）
259. 延安革命旧址——王家坪（73）
260. 王家坪革命旧址——延安红旅（26）
261. 延安旅记三 走进王家坪革命旧址（356）
262. 党史学习|红色印迹：陕西篇（八）延安王家坪革命旧址（25）
263. 红船破浪：王家坪革命旧址（180）
264. 延安王家坪革命旧址管理处“喜迎二十大 永远跟党走 奋进新征程”主题宣讲活动走进延长县城关派出所（353）
265. 【NO.29】红色印记 | 走进延安王家坪革命纪念馆（204）
266. 延安王家坪革命旧址管理处关于文物藏品资料征集公告（123）
267. 延安市延安革命遗址之王家坪革命旧址（110）
268. 王家坪革命旧址组织传达学习全市文物工作会议精神暨开展第三次文物安全消防培训（161）
269. 五四特辑｜参观王家坪革命旧址（126）
270. 云访革命旧址丨一起打卡延安革命纪念馆（15）
271. 延安王家坪革命旧址管理处 作风建设第二期集体学习（125）
272. 延安王家坪革命旧址管理处召开作风建设专题会议暨第一次专题学习（138）
273. 延安王家坪革命旧址开展2022年度消防演练 筑牢消防安全防线（71）
274. 延安王家坪革命旧址管理处传达学习局党委中心组第三次学习会会议精神（128）
275. 延安王家坪革命旧址管理处 召开专题会议传达学习全省文物局长会议精神（161）
276. 延安王家坪革命旧址管理处 2022年“5.18”国际博物馆日活动 --- 走进延安北关小学（187）
277. 延安王家坪革命旧址管理处开展主题党日活动 ---赴绥德、米脂参观学习（287）
278. 延安王家坪革命旧址 走进延安市监管医疗所（397）
279. 云访革命旧址丨一起打卡延安革命纪念馆（15）
280. 关于王家坪革命旧址恢复正常开放的公告（359）
281. 游遍陕西——延安地区（409）：王家坪革命旧址（21）
282. 延安王家坪革命旧址管理处组织传达学习局党委中心组第七次学习内容（208）
283. 延安王家坪革命旧址管理处关于调整讲解收费标准的公示（859）
284. 通知一一延安王家坪革命旧址管理处 关于文物藏品资料征集公告（552）
285. “相约王家坪，共谱爱国曲” ---延安王家坪革命旧址管理处国庆系列活动（127）
286. 延安12345 | 延安王家坪革命旧址管理处最新防控公告（621）
287. 护佑旧址安全 延安王家坪革命旧址开展反恐防暴应急演练（74）
288. 延安王家坪革命旧址管理处最新防控公告（323）
289. 王家坪革命旧址“5.18”国际博物馆日活动---走进延安知新小学（541）
290. 第一期｜听党史学习实践团讲延安红色教育基地——王家坪革命旧址（81）
291. 延安王家坪革命旧址管理处开展文物法规学习活动（218）
292. 延安王家坪革命旧址管理处传达延安革命纪念地管理局党委第五次中心组会议精神（175）
293. 延安王家坪革命旧址管理处走进延河分局开展”喜迎二十大 永远跟党走“主题宣讲暨 “公安心向党 护航新征程”主题党日活动（203）
294. 延安王家坪革命旧址管理处开展2022年度第一期防暴、防恐演练（344）
295. 延安王家坪革命旧址管理处 组织传达学习局党委中心组第六次学习内容（102）
296. 延安王家坪革命旧址：游客持48小时内核酸检测阴性证明方可参观（518）
297. 延安王家坪革命旧址管理处召开2022年度工作会议（294）
298. 【青讲】延安红色教育基地系列讲解（第一期） | 王家坪革命旧址（128）
299. 延安王家坪革命旧址管理处党支部 召开2021年度专题组织生活会（224）
300. 王家坪革命旧址等景区恢复运营（57）
301. 延安王家坪革命旧址管理处公开征集文物藏品资料啦（57）
302. 延安王家坪革命旧址党支部走进为人民服务讲话台开展“增强党性修养 加强作风建设”主题党日活动（158）
303. 延安王家坪革命旧址管理处召开学习宣传贯彻党的二十大报告工作部署会议（90）
304. 【延安事】延安王家坪革命旧址管理处公开征集文物藏品资料啦（431）
305. 在延安精神的照耀下勇毅前行……（1426）
306. 延安王家坪革命旧址管理处开展“慰问社区困难群众，真情关怀暖人心”活动（166）
307. 延安王家坪革命旧址管理处召开疫情防控工作会议（114）
308. 延安王家坪革命旧址管理处组织传达局党委第四次中心组会议精神（143）
309. 延安王家坪革命旧址管理处召开全体人员工作大会（103）
310. 王家坪革命旧址管理处赴甘泉开展“缅怀革命先烈 弘扬民族精神”主题党日暨“讲好延安故事 弘扬延安精神”主题宣讲进农村活动（320）
311. 延安王家坪革命旧址管理处党支部开展“增强党性修养 加强作风建设”主题党日活动（127）
312. 延安王家坪革命旧址管理处参观“从敦煌到延安”石窟艺术数字影像展 ——感受科技与文化融合的魅力（216）
313. 延安王家坪革命旧址管理处传达习近平总书记给中国国家博物馆老专家回信精神（193）
314. 学党史听党话跟党走 勇担使命庆祝建党百年 ---延安王家坪革命旧址管理处庆祝建党百年活动（159）
315. 学习百年党史，汲取奋进力量---延安王家坪革命旧址管理处党史学习教育活动之一（314）
316. 延安王家坪革命旧址管理处开展春节节前安全生产大检查（72）
317. 团委【学工通讯】第一期｜听党史学习实践团讲延安红色教育基地——王家坪革命旧址（73）
318. 延安王家坪革命旧址管理处开展绿化主题活动（199）
319. 延安王家坪革命旧址管理处党支部 开展“赓续伟大建党精神 接续奋进新时代新征程”主题党日活动（322）
320. 延安王家坪革命旧址管理处走进武警延安支队执勤四大队宝塔中队开展“同庆建军佳节 共叙鱼水深情”主题活动（319）
321. 延安王家坪革命旧址管理处开展祭奠“四·八烈士遇难日”主题纪念活动（282）
322. 关于延安王家坪革命旧址、杨家岭革命旧址、枣园革命旧址临时关闭的公告（1.4万）
323. 延安王家坪革命旧址管理处--传达学习习近平总书记来陕考察重要讲话重要指示精神（226）
324. 陕西延安王家坪革命旧址等场所恢复开放（91）
325. 学习百年党史，汲取奋进力量---延安王家坪革命旧址管理处党支部党史学习教育活动之五（114）
326. 延安王家坪革命旧址等景区恢复运营（12）
327. 延安王家坪革命旧址管理处 --开展党史学习教育进家庭“六一”儿童节亲子活动（328）
328. 延安“红色之旅”来了，一起去看宝塔山！（5601）
329. 情暖战“疫”一线——延安王家坪革命旧址管理处看望慰问一线下沉干部（556）
330. 延安王家坪革命旧址管理处党支部携手延安市粮食和物资储备系统党支部共同举办“学党史、悟思想、办实事、开新局” 党史学习活动（329）
331. 打好防疫攻坚战 闭关修炼正当时---延安王家坪革命旧址管理处开展全体党员干部线上学习活动（95）
332. 延安王家坪革命旧址管理处开展第二期消防安全知识培训（106）
333. 学习百年党史，汲取奋进力量---延安王家坪革命旧址管理处党支部党史学习教育活动之十三（114）
334. 延安王家坪革命旧址管理处党支部组织开展过政治生日·忆入党初心主题活动（184）
335. 【打卡红色地标】走进延安革命纪念馆（227）
336. 延安王家坪革命旧址党支部 召开2020年度组织生活会（139）
337. 学习百年党史，汲取奋进力量---延安王家坪革命旧址管理处党支部党史学习教育活动之四（114）
338. 延安王家坪革命旧址管理处进行国庆节前安全大检查（112）
339. 【红色足迹】学员参观延安革命纪念馆和王家坪革命旧址（188）
340. 学习百年党史，汲取奋进力量---延安王家坪革命旧址管理处党支部党史学习教育活动之十一（86）
341. 延安王家坪革命旧址管理处 组织学习《中华人民共和国反有组织犯罪法》（107）
342. 延安王家坪革命旧址管理处 “学党史 悟思想 我为群众办实事” 主题宣讲活动走进国网铜川供电公司（299）
343. 延安王家坪革命旧址管理处开展“迎七一，保平安”安全检查（89）
344. 延安王家坪革命旧址管理处 开展“党员心声大家谈”活动（118）
345. 学党史 悟思想 我为群众办实事延安王家坪革命旧址管理处走进延长石油延安能化公司（159）
346. 延安王家坪革命旧址管理处开展春季消防安全知识讲座（76）
347. 延安王家坪革命旧址管理处工会开展“关爱女性 呵护健康”三八节妇女活动（235）
348. 县旅游服务中心（文保中心）联合延安新闻纪念馆、王家坪革命旧址开展红色故事主题宣讲“六进”活动（61）
349. 学习百年党史，汲取奋进力量---延安王家坪革命旧址管理处党史学习教育活动之十七（105）
350. 打好防疫攻坚战 闭关修炼正当时---延安王家坪革命旧址管理处开展讲解员线上学习活动（140）
351. 延安王家坪革命旧址管理处开展领会党的十九届六中全会学习交流（212）
352. 延安王家坪革命旧址管理处党支部走进中国陆上第一口油井（149）
353. 延安王家坪革命旧址管理处开展“美丽中国 我是行动者”环保专题讲座和节前安排部署会（144）
354. 延安王家坪革命旧址管理处 开展讲解员党史知识测试（148）
355. 永远跟党走 奋进新时代 | 延安红浪实践队在王家坪革命旧址进行采访（54）
356. 延安王家坪革命旧址管理处党支部 组织党员志愿者把温暖送给抗疫一线工作人员（591）
357. 延安王家坪革命旧址管理处党支部与王家坪社区党支部开展“慰问贫困户，爱心帮扶送温暖”活动（227）
358. （周末版）革命旧址—王家坪（264）
359. 延安：共产党的成长与壮大（4万）
360. 党史学习在延安丨这一系列红色教育，生动又深刻！（7834）
361. 红色革命圣地--延安革命纪念馆（36）
362. 从延安到西柏坡 | 24个字的入党誓词？这里有故事！（230）
363. 【“英”者万象】百个红色景点“云”参观（53）| 王家坪革命旧址（111）
364. 王家坪革命旧址——最小的指挥所，却领导百万雄师！（65）
365. 【特别关心】2021年寒期红色足迹--延安之旅，追寻革命先辈足迹——关于王家坪旧址的峥嵘岁月（214）
366. 【卫星x红色旅游】陕西延安革命旧址（220）
367. 云观革命旧址丨走进延安革命纪念馆（24）
368. 延安王家坪革命旧址管理处“八一”建军节前夕慰问延安消防官兵（280）
369. 延安王家坪革命旧址管理处 【我为群众办实事】把温暖送给抗疫一线工作人员（948）
370. 延安3处革命旧址临时关闭！西安城墙部分区域封控！澳门确诊病例在陕轨迹公布！（2267）
371. 【红领巾寻访】弘扬延安精神 争做时代新人 | 记二（2）中队主题寻访活动（236）
372. 【环境·暑期社会实践】踏红色沃土 读峥嵘史诗——走访延安革命纪念馆 及革命旧址（212）
373. 延安王家坪革命旧址管理处携手王家坪社区共同举办“红色故事会” 让党史学习更入心（60）
374. 1973年周恩来总理在延安（195）
375. 【食·暑期社会实践】踏红色沃土 读峥嵘史诗——走访延安革命纪念馆 及革命旧址（172）
376. 延安王家坪革命旧址管理处 组织全体党员干部观看电影《1921》（132）
377. 【中国共产党人的精神谱系】延安精神永放光芒（47）
378. 锚定目标 | 2023年底延安建成全国一流革命文物保护利用示范区（1561）
379. 重温红色岁月，感悟延安精神——南京市鼓楼区察哈尔路社区开展党史学习讲座（350）
380. 【情怀依旧】羊杂碎（1406）
381. 学习贯彻党的二十大精神 | 丰富川采油队党支部联合丰富川联合站党支部开展“弘扬延安精神 培塑优良作风”主题党日活动（217）
382. 延安王家坪革命旧址关于闭馆的通知（314）
383. 【红色故事】毛岸英和父亲毛泽东二三事（22）
384. 红色记忆|王晓建：开国少将贺光华的延安岁月（141）
385. 【云参观】云游红色教育基地 学习红色文化精神——喀什地区消防救援支队各级开展“云参观”红色教育基地（236）
386. 【诵读新声】《一张纸币的思考》（作者/龚伟 诵读/彭孟琦）（16）
387. 延安王家坪革命旧址（33）
388. 王家坪革命旧址背后的故事（28）
389. 中国最早建立的革命纪念馆之一——王家坪革命纪念馆（119）
390. 【云溪山人游记】延安红色之旅5--王家坪革命旧址（228）
391. 讲好红色故事 传承革命精神---王家坪革命旧址管理处走进西河口小学（233）
392. 陕西延安王家坪革命旧址 八路军总部领导的旧居（40）
393. 亲情中华·云上基地：走进延安王家坪革命旧址（199）
394. 王家坪革命旧址恢复对外开放公告（45）
395. 【红色延安】现场教学之王家坪革命旧址（59）
396. 【应用案例】延安王家坪革命纪念馆加固改造工程（433）
397. 延安王家坪革命旧址管理处开展新冠状病毒感染疫情防控工作（142）
398. 延安红色调研活动 | 王家坪革命旧址（129）
399. 心连心 同携手 战疫情 延安文博人在行动---延安王家坪革命旧址庆祝2020年“5.18国际博物馆日”（122）
400. 延安王家坪革命旧址管理处开展节前消防安全检查及防疫部署（89）
401. 延安王家坪革命旧址开展讲解员专题培训（152）
402. 【媒体关注】王家坪革命旧址—追忆远去的红色烙印（139）
403. 【陕西名胜】王家坪革命旧址（47）
404. 相约延安 祝福祖国---庆祝新中国成立70周年王家坪革命旧址系列活动（206）
405. 【中红延育】延安之行·王家坪革命旧址（15）
406. 【不忘初心，牢记使命】重走长征路之延安“王家坪革命旧址”（55）
407. 【走遍中国·王家坪革命旧址】-（33）
408. 延安王家坪革命旧址管理处组织学习十九届五中全会精神（116）
409. 延安王家坪革命旧址管理处开展清雪活动（236）
410. “绘出我心中的王家坪革命旧址”（64）
411. 延安讲乐营之王家坪革命旧址（129）
412. 以青春的名义---记延安王家坪革命旧址“五四”青年节（150）
413. 传承革命传统 讲好红色故事---延安王家坪革命旧址管理处走进宝塔区蟠龙镇（217）
414. 延安王家坪革命旧址管理处 召开创文工作部署会议（78）
415. 延安王家坪革命旧址管理处传达学习局党委第十一次中心组学习会议精神
416. （87）
417. 延安王家坪革命旧址管理处党支部开展庆祝建党九十九周年主题活动（153）
418. 延安王家坪革命旧址管理处消防培训和消防演练（111）
419. 延安王家坪革命旧址管理处荣获“延安市先进集体”荣誉称号（96）
420. 延安王家坪革命旧址管理处召开专题会议，传达关于王西荣等五人违纪违法问题的通报（229）
421. 延安王家坪革命旧址管理处组织传达学习局党委中心组学习内容（105）
422. 延安王家坪革命旧址管理处学习传达局党委第八次中心组会议精神（79）
423. 延安王家坪革命旧址管理处开展消防安全知识讲座（150）
424. 延安王家坪革命旧址管理处 传达学习延安革命纪念地管理局党委中心组会议精神（114）
425. 延安王家坪革命旧址管理处开展讲解员培训心得交流讨论会（118）
426. 牢记初心·不忘使命 我为党旗添光彩 延安王家坪革命旧址管理处举办主题演讲比赛（222）
427. 缅怀革命先烈 弘扬爱国精神---延安王家坪革命旧址管理处赴四八烈士陵园开展主题公祭活动（100）
428. 走进王家坪革命旧址（49）
429. 红色延安|延安革命纪念馆&王家坪旧址（207）
430. “重走长征路、追忆红色魂”之王家坪旧址（199）
431. 延安王家坪革命旧址管理处党支部参观《东方欲晓》---延安革命纪念馆馆藏精品书画展（148）
432. 王家坪革命旧址管理处开展“寻心梁家河•传承延安魂”主题活动（176）
433. 延安王家坪革命旧址管理处同延安知新小学签订馆校合作协议（455）
434. 延安王家坪革命旧址管理处党支部组织党员学习专题宣讲（288）
435. 【成长学子】征文延安行之王家坪革命旧址（132）
436. 延安王家坪革命旧址管理处领导班子召开 赵正永严重违纪违法案以案促改专题民主生活会（122）
437. 延安王家坪革命旧址管理处 开展春节前消防安全大检查（39）
438. 延安王家坪革命旧址管理处 ---开展安全健康知识专题讲座（50）
439. 延安王家坪革命旧址全体党员干部 组织开展“研学十九大报告”学习活动（112）
440. 传承革命传统，讲好红色故事---延安王家坪革命旧址管理处走进延安市戒毒所（106）
441. 延安王家坪革命旧址管理处召开“讲政治、敢担当、改作风”专题教育集体学习会 （62）
442. 延安王家坪革命旧址管理处迎接 年终目标责任考核（256）
443. 王家坪革命旧址管理处八一建军节 走进敬老院（8）
444. 延安王家坪革命旧址管理处举行思政课校外实践教学基地挂牌仪式（106）
445. 延安王家坪革命旧址管理处召开消防安全工作会议（113）
446. 延安王家坪革命旧址管理处召开安全工作会议（116）
447. 延安王家坪革命旧址管理处党支部 组织党员干部参观“回望延安—中国共产党人的初心和使命”展览（100）
448. 延安王家坪革命旧址管理处党支部 召开专题组织生活会（43）
449. 延安王家坪革命旧址管理处 开展春季消防安全培训（131）
450. 延安王家坪革命旧址管理处党支部 组织传达学习《党委（党组）落实全面从严治党主体责任规定》精神（128）
451. 延安王家坪革命旧址管理处开展消防安全培训讲座）（75）
452. 延安王家坪革命旧址管理处党支部 “牢记初心使命 我为党旗添彩” ----走进枣园重温“初心”（107）
453. “不忘初心 牢记使命” 延安王家坪革命旧址管理处党支部 ----支部书记讲党课走进凤凰山革命旧址（65）
454. 延安王家坪革命旧址管理处党支部学习习近平总书记来陕重要讲话重要指示分享交流（92）
455. 延安王家坪革命旧址管理处 召开全体职工干部大会（159）
456. 延安王家坪革命旧址管理处开展消防演练（74）
457. 延安王家坪革命旧址管理处党支部开展祭奠“四·八烈士遇难日”主题纪念活动（60）
458. 延安王家坪革命旧址管理处党支部 组织学习《习近平对张富清同志先进事迹重要指示》（67）
459. 延安王家坪革命旧址管理处 开展警钟日消防安全大检查（81）
460. 现场感受|延安革命纪念馆和王家坪革命旧址（500）
461. 庆祝中国共产党成立98周年 ——延安王家坪革命旧址管理处党支部走进王家坪社区慰问老党员（37）
462. 延安王家坪革命旧址管理处党支部 开展廉政警示教育活动（100）
463. 延安王家坪革命旧址管理处党支部 “牢记初心使命 我为党旗添彩” ----走进梁家河“秉承初心使命、牢记责任担当”（124）
464. 延安王家坪革命旧址管理处党支部 组织学习《中共中央关于加强党的政治建设的意见》和《中国共产党重大事项请示报告条例》（112）
465. 延安王家坪革命旧址管理处党支部 组织学习十九届四中全会精神和胡和平来延安调研指示精神（107）
466. 延安王家坪革命旧址管理处党支部 “牢记初心使命 我为党旗添彩” ----走进杨家岭“再誓忠诚、牢记纪律”（112）
467. 延安王家坪革命旧址管理处 开展安全警钟日检查（63）
468. 延安王家坪革命旧址管理处召开干部作风整治会议（95）
469. 延安王家坪革命旧址管理处党支部 --开展党员活动日卫生大扫除活动（62）
470. 在职党员进社区 扶贫帮困暖人心 -----延安王家坪革命旧址管理处党支部走进王家坪社区（43）
471. 延安王家坪革命旧址管理处党支部 --组织观看《厉害了，我的国》为祖国点赞（72）
472. 王家坪革命旧址管理处走进延安职业技术学院（118）
473. 延安王家坪革命旧址管理处 进行消防安全演练（66）
474. 延安——王家坪（83）
475. 王家坪革命旧址管理处党支部组织党员干部观看电影《周恩来回延安》（97）
476. 延安王家坪革命旧址管理处党支部 组织观看《榜样4》（92）
477. 参观王家坪革命旧址（24）
478. 不忘初心牢记使命” 延安王家坪革命旧址管理处党支部 组织党员干部观看电影《音乐家》（68）
479. 延安王家坪革命旧址管理处 组织全体党员干部观看电影《我和我的祖国》（96）
480. 弘扬延安精神 勇担时代使命——县委组织部、县财政局党支部赴延安开展主题教育活动（1327
481. VR带您走进陕西 | 八路军总司令部旧址王家坪（68）
482. 传承弘扬延安精神 不忘初心携手前进 ——市委会领导班子成员赴延安参加杭州市各民主党派工商联领导干部和无党派人士读书班（173）
483. 延安王家坪革命旧址管理处 --开展“警钟日”安全大检查（81）
484. 延安王家坪革命旧址管理处党支部召开全体党员会向 “三秦楷模”陈士橹、柯小海、刘永生同志学习（118）
485. **视频：107**

**杨家岭革命旧址**

1. 云观革命旧址丨走进延安杨家岭革命旧址（10）
2. 希望在延安 | 杨家岭革命旧址（429）
3. 【美丽瞬间随手拍】延安杨家岭革命旧址（81）
4. 【延安革命旧址：一本读不完的书】杨家岭的菜地（73）
5. 【党史知识】陕西革命旧址云上展：延安枣园革命旧址（1251）
6. 红色起点·延安杨家岭革命旧址（15）
7. 延安革命圣地——杨家岭（213）
8. 走进杨家岭革命旧址，感悟延安精神（1.6万）
9. 红旗漫卷——陕西革命旧址云上展：延安杨家岭革命旧址（450）
10. 云观革命旧址丨走进延安枣园革命旧址（11）
11. 陕西革命旧址云上展（四） | 延安杨家岭革命旧址（126）
12. 红色印记 ｜ 延安杨家岭旧址（第54期）（88）
13. 革命圣地延安-杨家岭（76）
14. 红旗漫卷 | 陕西革命旧址云上展——杨家岭革命旧址（315）
15. 让杨家岭革命旧址成为青少年教育的生动课堂（939）
16. 足迹·温暖的回响 | 杨家岭七大会址：红色基因代代传（753）
17. 第八期 延安杨家岭革命旧址（13）
18. 距离天安门955公里 | 星火燎原，带你走进延安枣园革命旧址​（623）
19. 【学党史 悟思想】党史学习||走进杨家岭革命旧址，感悟延安精神（184）
20. 循党史 访红迹(第8期) | 杨家岭革命旧址（50）
21. 建党百年10 | 杨家岭革命旧址（34）
22. 重温初心使命 传承红色精神——走进杨家岭革命旧址（1170）
23. 足迹·温暖的回响 | 杨家岭革命旧址：殷殷嘱托记心间 红色基因代代传（1234）
24. 八路军西安办事处纪念馆与延安杨家岭革命旧址管理处签署战略合作协议（544）
25. 【奋进新征程 建功新时代·老区新貌】杨家岭革命旧址：殷殷嘱托记心间 红色基因代代传（528）
26. 【红色堡垒·百年党史】红色地图 | 陕西革命旧址云上展：杨家岭革命旧址（80）
27. 红色延安-杨家岭革命旧址学习（321）
28. “百年华诞，青春向党” ｜ 延安革命史学习第五站 ——杨家岭革命旧（279）
29. 延安之旅——杨家岭革命旧址（264）
30. 【党史故事汇】杨家岭七大会址：光辉历程 伟大业绩（1150）
31. 巾帼讲解员讲党史 | 杨家岭旧址的红色革命记忆（276）
32. “云游”红色景区 | 延安枣园革命旧址（147）
33. 百个红色景点“云”参观㊵ | 延安枣园革命旧址（228）
34. 【党史学习教育】陕西延安枣园革命旧址（205）
35. 【光辉100年•党史天天读】陕西延安枣园革命旧址（70）
36. 延安杨家岭革命旧址组织开展国庆节前反恐防暴安全综合演练活动（140）
37. 延安市延安革命遗址之杨家岭革命旧址（65）
38. 一次延安行，一生延安情—杨家岭革命旧址（26）
39. 【红色地标】杨家岭革命旧址（34）
40. 我心向党︱馆长讲党史：延安杨家岭革命旧址（26）
41. 延安市延安革命遗址之杨家岭革命旧址中共七大会址（314）
42. 延安杨家岭革命旧址图赏（277）
43. 党史学习|红色印迹：陕西篇（一）杨家岭革命旧址（67
44. 延安杨家岭革命旧址管理处对新冠肺炎疫情防控工作进行再部署、再落实（153
45. 陕西革命旧址云上展：延安枣园革命旧址（40）
46. 务必参加！11月23日宝塔区6办3镇核酸检测！红军哥带您游览延安杨家岭革命旧址（10万+）
47. 青听党史 | 陕西延安枣园革命旧址（134）
48. 红色足迹 | 延安杨家岭革命旧址（86）
49. 11月23日宝塔主城区重点区域核酸检测！红军哥带您揽胜延安杨家岭革命旧址！（1320）
50. 【杨家岭歌者】《中央大礼堂的光芒——瞻仰延安杨家岭革命旧址有感》作者：陈希学 朗读者：白晓玲（146）
51. 陕西延安革命纪念地之杨家岭革命旧址（120）
52. 游遍陕西——延安地区（407）：杨家岭革命旧址（19）
53. 二十大时光｜杨家岭革命旧址管理处举办党的二十大精神专题辅导讲座（472）
54. 二十大时光｜延安杨家岭革命旧址管理处传达延安革命纪念地管理局党委理论学习中心组第八次学习会议精神（193）
55. 延安马文瑞红军小学（延安职业技术学院附属小学）走进杨家岭革命旧址（390）
56. 【延安革命旧址：一本读不完的书】延安窑洞对（65）
57. 延安杨家岭革命旧址管理处开展例行安全检查活动（98）
58. 经开交警：参观杨家岭革命旧址，学习宣传贯彻党的二十大精神（112）
59. 革命文物保护利用，陕西经验值得推广！（3）
60. 陕西延安杨家岭革命旧址：讲好红色故事 走向新的胜利（45）
61. 延安杨家岭革命旧址管理处 关于调整讲解收费标准的公示（202）
62. 习近平带领中共中央政治局常委瞻仰延安革命纪念地（9.1万）
63. 百年党史天天读5月6日 走进杨家岭革命旧址，感悟延安精神（108）
64. 走进杨家岭革命旧址 延安精神滋养初心（1777）
65. 延安杨家岭革命旧址召开“五一”小长假安全工作部署会（89）
66. 延安杨家岭革命旧址管理处开展国庆节前安全大检查活动（98）
67. 一百堂党史课（51）：延安杨家岭革命旧址——全国人民的希望都寄托在这儿的土窑洞里（17）
68. 我们一起学党史 | 延安杨家岭革命旧址的红色记忆（35）
69. 延安杨家岭革命旧址管理处传达学习局党委中心组集体学习会议精神（136）
70. 青年讲党史丨杨家岭革命旧址（209）
71. 延安杨家岭革命旧址暂停开放公告（487）
72. 深度阅读丨走进杨家岭革命旧址 延安精神滋养初心（22）
73. 【忆百年征程 学党史专栏 第二十五期】陕西延安杨家岭革命旧址：讲好红色故事 走向新的胜利（132）
74. 聚焦延安 | 走进杨家岭革命旧址 延安精神滋养初心（91）
75. 喜报！延安杨家岭革命旧址荣获“陕西省三八红旗集体”称号（382）
76. 学党史 | 山丹丹花开红艳艳（5）
77. 金台潮声 | 作风建设永远在路上（21）
78. 一百堂党史课32 | 走进杨家岭革命旧址 延安精神滋养初心（14）
79. 一百堂党史课丨走进杨家岭革命旧址 延安精神滋养初心（24）
80. 红色印象（六）—杨家岭革命旧址（170）
81. 管好用好革命文物 传承弘扬红色文化——革命文物保护利用的“陕西经验（217）
82. 延安杨家岭革命旧址传达学习全省文物局长会议精神（135）
83. 【红色基地】红色摇篮•延安精神——延安杨家岭革命旧址（36）
84. 南泥湾采油厂领导班子赴杨家岭革命旧址开展主题党日活动（457）
85. 在新的赶考之路上向历史和人民交出新的优异答卷（1132）
86. 周五，延中与杨家岭革命旧址签署了一个重要协议！（3161）
87. 到延安去！丨窑洞小院里的一次特别宴请（32）
88. 听党史学习实践团讲延安红色教育基地——杨家岭革命旧址（55）
89. 上海周浦监狱警察线上瞻仰延安杨家岭革命旧址（573）
90. 学党史丨延安行之杨家岭革命旧址（151）
91. 延安杨家岭革命旧址学习传达延安革命纪念地管理局党委（扩大）会议精神（122）
92. 延安杨家岭革命旧址管理处 召开2月份安全工作会议（121）
93. 延安杨家岭革命旧址管理处 召开党史学习教育专题组织生活会（144）
94. 延安杨家岭革命旧址管理处开展 文物法规宣传周活动（120）
95. 《理想照耀中国》系列融媒直播聚焦延安杨家岭革命旧址（189）
96. 一百堂党史课·馆长讲党史 _ 延安杨家岭革命旧址（11）
97. 延安杨家岭革命旧址管理处召开半年工作推进会（36）
98. 深沉缅怀 致敬英魂 ——延安杨家岭革命旧址管理处（160）
99. 延安杨家岭革命旧址管理处传达学习 党的十九届六中全会精神（125）
100. 鉴往知来，跟着总书记学历史丨杨家岭，追寻“再出发”的密钥（961）
101. 到延安去！丨窑洞小院里的一次特别宴请（32）
102. 让延安精神照亮逐梦新征程（270）
103. 百年路·新征程⑤ | 对照过去认不出了你，圣地延安（4963）
104. 延安杨家岭革命旧址管理处召开重点工作推进会（18）
105. 【红色足迹】巍巍宝塔 初心永照——习近平总书记到过的红色圣地之陕西篇（1296）
106. 诗歌◇写在延安杨家岭革命旧址/萧习华（127）
107. 延安杨家岭革命旧址管理处 传达局党委中心组学习会议精神（131）
108. 延安日记第三天 | 杨家岭革命旧址（176）
109. 延安杨家岭革命旧址管理处2021年讲解员等级评定考核（643）
110. 延安吃着小米饭、穿着麻草鞋，你为什么还奔延安？（7.5万）
111. 延安杨家岭革命旧址管理处 深入学习贯彻党的十九届六中全会精神（31）
112. 永远跟党走 奋进新时代|延安红浪实践队前往杨家岭革命旧址 学习贯彻延安革命精神（201）
113. 党史课 | 延安精神——我们的传家宝（1329）
114. 二十大时光｜杨家岭革命旧址管理处党支部走进延安中学举行“红色育人资源平台”和“红色育人实践基地”签约授牌仪式（409）
115. 我们在路上 | 陕西吴起实践分团集体参观杨家岭革命旧址和延安革命纪念馆（338）
116. 【党风廉政专栏】延安精神，党的宝贵精神财富（53）
117. 【理响合肥】习近平讲故事 | 怎样才叫“领导”（0）
118. 百年芳华丨几回回梦里回延安（7.7万）
119. 学党史 跟党走|延安中学党委赴杨家岭革命旧址开展“学党史 明理增信 崇德力行”主题党日活动（1416）
120. 厚重延安到底有多“厚”？延安市政协主席告诉你（3）
121. 革命文物保护利用，来自陕西的行动与经验（159）
122. 延安精神是我们党的宝贵精神财富 ——学习《论中国共产党历史》（十四）（1.7万）
123. 延安科技馆组织党员干部赴杨家岭革命旧址开展党史学习教育主题党日活动（264）
124. 党史学习教育常态化长效化┃跟着总书记学党史之杨家岭，追寻“再出发”的密钥（28）
125. 学习二十大 | “窑洞对”的“第二个答案”（30）
126. 延安精神是我们党的宝贵精神财富 | 笔记君学党史(（5419）
127. 党的二十大精神“学习微课堂”（十）（61）
128. 【党的二十大精神丨学习感悟】读懂“窑洞对”的两个答案（47）
129. 陕西延安：将红色文化植入城市文明建设（3176）
130. 二十大学习聚焦｜支部书记问答：“窑洞对”的“第二个答案”是什么？（88）
131. 陕西延安杨家岭革命旧址：讲好党史故事 让红色基因代代相传（5）
132. 参观延安杨家岭革命旧址（785）
133. 延安杨家岭革命旧址管理处 开展环境保护暨安全健康知识讲座（34）
134. 党群阵地@你 | 东城区党群服务中心迅速掀起学习宣传贯彻党的二十大精神热潮（三（183）
135. 延安杨家岭革命旧址变电站西安制造（127）
136. 让延安精神长久融入纪检监察血脉（4820）
137. 让红色地标成为“沉浸式”课堂，让红色基因融入青年成长血脉（70）
138. 弘扬延安精神 赓续红色传承——民革厦门市委常委会组成人员瞻仰延安革命纪念地（87）
139. 【延安革命故事】杨家岭的菜地（212）
140. 以党的二十大精神为指导，不断推进延安精神研究宣传工作上新的台阶（90）
141. 平安建设工作篇】甘泉公安：狠抓“六个聚焦” 提升公安队伍建设效能（55）
142. 葛晓燕：坚持以更高质量的人民司法 切实回应好新时代人民群众司法需求（31）
143. 行业动态 ‖ 浦发银行西安分行：传承红色基因 “延安精神”铸魂（9）
144. 红色景点丨延安杨家岭革命旧址（109）
145. 延安杨家岭革命旧址9（109）
146. 晋陕游记（12）：延安杨家岭革命旧址景区（183）
147. 寻红色文化印记--延安枣园革命旧址（53）
148. “不忘初心 牢记使命”主题教育基地 | 杨家岭革命旧址（90）
149. 【不忘初心，牢记使命】重走长征路之延安“杨家岭革命旧址”（41）
150. 延安记忆 | 杨家岭革命旧址（148）
151. 杨家岭革命旧址（331）
152. 延安精神：杨家岭革命旧址（221）
153. 探访延安杨家岭革命旧址）（192）
154. 杨家岭革命旧址：处处绽放圣地风采 游客参观学习好去处（282）
155. VR全景游览 · 延安杨家岭革命旧址（162）
156. 延安杨家岭革命旧址管理处：让游客玩的高兴 玩的放心（292）
157. 【旅游资讯】延安——杨家岭（革命旧址）·宝塔山！（17）
158. “延安杨家岭革命旧址——追寻先辈足迹，感受红色文化”（222）
159. 全国网媒延安行｜杨家岭革命旧址：回望历史感受红色文化（139）
160. 延安红色之行（四）：杨家岭革命旧址（68）
161. 延安杨家岭革命旧址管理处关于新冠肺炎防控与旧址开放应急预案（351）
162. 延安杨家岭革命旧址管理处征集抗击新冠肺炎疫情见证物的公告（67）
163. 杨家岭革命旧址：点燃红色火种 讲好革命故事（715）
164. 陕西省委常委班子走进延安杨家岭七大革命旧址 重温入党誓词（1.3万）
165. 延安杨家岭革命旧址管理处召开《百姓问政》问题整改部署会(（184）
166. 走进杨家岭革命旧址（52）
167. 参观杨家岭革命旧址（59）
168. 【红色延安】现场教学之杨家岭革命旧址（19）
169. 【红色记忆】杨家岭革命旧址（52）
170. 延安杨家岭革命旧址管理处召开创文工作推进会（212）
171. 延安杨家岭革命旧址管理处召开国家5A级旅游景区创建工作座谈会（76）
172. 延安杨家岭革命旧址管理处传达学习市委五届九次全会精神（133）
173. 延安杨家岭革命旧址举行“防风险 迎大庆”消防实战演练（171）
174. 延安杨家岭革命旧址管理处召开半年工作总结会（228）
175. 延安杨家岭旧址讲解词（140）
176. 延安杨家岭革命旧址管理处开展健康知识讲座（147）
177. 延安杨家岭革命旧址管理处“微型消防队”成立了（29）
178. 延安杨家岭革命旧址管理处党支部 圆满完成换届选举工作（224）
179. 延安杨家岭革命旧址开展迎建国70周年大庆消防宣传和消防演练（12）
180. 延安杨家岭革命旧址管理处举办创建国家5A级旅游景区讲解员语言技能提升培训（65）
181. 延安杨家岭革命旧址管理处开展春节前安全检查（26）
182. 延安杨家岭革命旧址管理处组织召开扫黑除恶专项斗争整改落实会议（112）
183. 延安杨家岭革命旧址管理处开展冬季消防安全知识培训（10）
184. ​延安杨家岭革命旧址管理处组织入冬前消防安全知识培训（100）
185. 【最新】关于延安杨家岭、枣园革命旧址参观游览路线的调整公告（747）
186. 延安杨家岭革命旧址管理处工会委员会 “共建美丽旧址 共享美好生活”活动（112）
187. 延安杨家岭革命旧址管理处成立了“微型消防队”（23）
188. 【"五一"我在岗】杨家岭革命旧址讲解员 坚守也快乐（720）
189. 延安杨家岭革命旧址管理处深入学习贯彻党的十九届四中全会精神（74）
190. 红色文化 永恒记忆” —纪念“5.23”讲话75周年活动在杨家岭革命旧址举行（549）
191. 延安杨家岭革命旧址管理处召开“两学一做”学习教育工作会议（34）
192. 杨家岭革命旧址宣解小分队走进延安大学鲁艺学院（48）
193. 杨家岭革命旧址讲解员：讲好延安故事 坚守也快乐（40）
194. “不忘初心、继续前进”延安行(之二）一一杨家岭的党课（2361）
195. 延安杨家岭革命旧址，看到这才感受到真正的“窑洞建筑”，是啥样（8）
196. **视频：159**

**枣园革命旧址,**

1. 云访革命旧址丨一起打卡延安枣园革命旧址（5）
2. 老区新貌｜巍巍宝塔，滔滔延河，这里是革命老区延安（5596）
3. 【汉中蜀道石刻艺术博物馆】党史知识：陕西革命旧址云上展：延安枣园革命旧址！（19）
4. 景区推荐 | 星火燎原，带你走进延安枣园革命旧址​（82）
5. 延安之旅——参观枣园中国革命旧址 ​（787）
6. “苏&陕红色云旅游”暑期社会实践|第一小分队：延安枣园革命旧址（42）
7. 红旗漫卷——陕西革命旧址云上展（延安枣园革命旧址）（63）
8. 西革命旧址云上展（三） | 延安枣园革命旧址（125）
9. 学党史 | 【 共和国从这里走来】陕西延安枣园革命旧址（179）
10. 学党史 正青春|宝塔城指和您一起打卡红色地标—“延安枣园革命旧址”（26）
11. 延安革命旧址——枣园（156）
12. 《革命圣地延安-枣园革命旧址》（451）
13. 革命圣地延安-枣园（136）
14. 【枣园知道】“百年辉煌看延安 坚定信念跟党走”——枣园革命旧址“五一”主题系列活动（一）（138）
15. 【学党史 颂党恩】27 延安枣园革命旧址（24）
16. 【枣园知道】“五一”小长假 延安枣园革命旧址游客接待达7.35万人次（96）
17. 沿着延西高速去旅行 ——带你一起走进圣地延安畅游枣园革命旧址（111）
18. 【红色堡垒·百年党史】红色地图 | 陕西革命旧址云上展：延安枣园革命旧址（116）
19. 【枣园知道】延安枣园革命旧址食品安全知识科普（59）
20. 【枣园知道】喜报：延安枣园革命旧址管理处荣获2021年度消防宣传工作先进集体和先进个人（341）
21. 【年终总结】“赓续红色血脉，传承红色基因”延安枣园革命旧址干部培训中心全年培训纪实（285）
22. 不忘初心跟党走，红色精神代代传 ——内蒙古志愿者走进延安革命圣地之二：枣园革命旧址（489）
23. 巾帼讲解员讲党史 | 星火燎原，走进延安枣园，听一架纺车背后的故事（646
24. 喜迎建党百年 云访红色基地|第十一站：延安枣园革命旧址（336）
25. 《理想照耀中国》系列融媒直播聚焦延安枣园革命旧址（362）
26. 宝塔山下讲党史|胡曲利：星火燎原，带你走进延安枣园革命旧址（2260）
27. 【枣园知道】延安枣园革命旧址管理处学习传达延安革命纪念地管理局党委（扩大）会议精神（218）
28. 延安市延安革命遗址之枣园革命旧址（105）
29. 追寻红色记忆——陕北枣园革命旧址（72）
30. 延安枣园革命旧址/大美地球村（13）
31. 枣园知道】延安枣园革命旧址管理处关于调整讲解收费标准的公示（2658）
32. 百篇陕西红色经典|第七十二篇 枣园革命旧址（127）
33. 【枣园知道】延安枣园革命旧址管理处严格落实疫情防控措施的公告（369）
34. 学党史系列活动之二：参观延安枣园革命旧址（31）
35. 【枣园知道】延安枣园革命旧址管理处关于疫情防控最新公告（588）
36. 【瞬间】延安枣园：见证中国革命走向高潮（65）
37. 延安枣园革命旧址——红色经典景区——陕西免费景点（115）
38. 今日延安 | 枣园革命旧址：党史教育成主题 红色文化润人心（253）
39. 【枣园知道】延安枣园革命旧址管理处开展“学习二十大 永远跟党走 奋进新征程”主题宣讲系列活动二（139）
40. 【枣园知道】延安枣园革命旧址管理处开展“学习二十大 永远跟党走 奋进新征程”主题宣讲系列活动一（184）
41. 红色之旅｜走进延安枣园革命旧址，感受革命初心力量（462）
42. 百年党史日日学 | 陕西革命旧址云上展：延安枣园革命旧址（116）
43. 延安枣园革命旧址管理处关于调整讲解收费标准的公示（163）
44. 美丽瞬间随手拍】走进延安枣园革命旧址 感受革命初心力量（127）
45. 【枣园知道】延安枣园革命旧址管理处党支部传达学习全市文物工作会议精神（232）
46. 【美丽瞬间随手拍】延安枣园革命旧址（43）
47. 游遍陕西——延安地区（408）：枣园革命旧址（72）
48. 党史宣讲 | 走进杨家岭革命旧址，感悟延安精神（18）
49. 【枣园知道】延安枣园革命旧址管理处党支部开展“学习贯彻二十大精神 赓续红色血脉 传承奋斗精神”主题党日活动（483）
50. 【公益云课】现场直播丨党史教育系列《红色圣地-延安枣园革命旧址》（523）
51. 云上微书||延安枣园革命旧址，云云来啦~（94）
52. 【枣园知道】延安枣园革命旧址管理处党支部举办学习贯彻党的二十大精神专题辅导讲座（245）
53. 红色故事寻访｜走进延安枣园革命旧址，感受革命初心力量（92）
54. 【枣园知道】延安枣园革命旧址管理处 五一“追寻红色记忆 勇攀时代高峰 喜迎二十大 健康脚下行”登山主题活动（138）
55. 【青讲】延安红色教育基地系列讲解(第二期) | 枣园革命旧址（133）
56. 【枣园知道】延安枣园革命旧址5.18国际博物馆日走进延安北关小学主题活动（193）
57. 我们一起学党史 | 延安枣园革命旧址的红色记忆（118）
58. 【枣园知道】延安枣园革命旧址管理处疫情防控紧急通知（1324）
59. 【枣园知道】压实责任防疫情 传导压力重实效——延安枣园革命旧址管理处召开全体干部职工大会（253）
60. 追寻红色记忆 传承红色基因--延安移动与延安枣园革命旧址管理处开展“党建和创”主题实践活动（111）
61. 【枣园知道】紧盯安全防线 夯实安全责任——延安枣园革命旧址管理处开展国庆节安全大检查（131）
62. “情满中秋红动延安，盛世国庆秀美枣园”——枣园革命旧址庆“双节”系列活动（11）
63. 枣园革命旧址------延安（192）
64. 学党史丨延安行之枣园革命旧址（201）
65. 【枣园知道】延安枣园革命旧址管理处召开疫情防控紧急会议（282）
66. 暑期实践|回顾延安岁月（三）：参观枣园革命旧址（28）
67. 【枣园知道】延安枣园革命旧址宣教部召开会议学习习近平总书记来陕考察重要讲话（154）
68. 【枣园知道】延安枣园革命旧址管理处党支部传达学习延安革命纪念地管理局党委（扩大）会议精神（152）
69. 【枣园知道】延安枣园革命旧址管理处召开安保会议安排部署近期工作（239）
70. 陕西延安：延安精神在身边（2428）
71. 延安市延安革命遗址之枣园革命旧址中共中央机构（59）
72. 传承红色精神 | 陕西延安驻防队伍参观枣园革命旧址（291）
73. 宁夏侨联与延安枣园革命旧址管理处开展党史联学共建活动（39）
74. “庆祝建党百年 传承红色基因 走进基层部队 赓续红色血脉”主题宣讲活动暨师乃博夫妇革命文物捐赠仪式在北京隆重举行（725）
75. 跟着全运火炬游陕西（一）（2862）
76. 延安市延安革命遗址之枣园革命旧址中央军委枣园作战室（80）
77. 汉院“三下乡” | 枣园红心，继往开来——汉口学院知行青蓝实践团于延安枣园革命旧址展开党史学习活动（262）
78. “融合红蓝使命、共筑‘陕亮’人心”：延安移动、枣园革命旧址管理处、延安宝塔消防大队开展主题党日活动（43）
79. 从延安枣园出发，1946年共青团迎来新生（2.7万）
80. 喜报！我市20家单位被命名为陕西省青少年教育基地！（1920）
81. 2021年11月18日 | 十九届六中全会，习近平总书记再谈“窑洞对”（1554）
82. 红旗渠精神｜我国的红色水利遗产——从红旗渠谈起（577）
83. 党史故事天天读 227期（38）
84. 《中国文化报》头版刊发《延安枣园：在情感共鸣中传承延安精神》（240）
85. 五一攻略4.0 | 开启红色文化之旅，重温陕西的红色记忆（1576）
86. 【弘扬红旗渠精神】我国的红色水利遗产——从红旗渠谈起（26）
87. 兰爱平调研延安北京知青博物馆 枣园革命旧址（392）
88. 坐着火车逛陕西！陕西旅游专列启动中，“东方红号”一马当先！（1万）
89. 聚焦革命文物保护，带你一览延安革命遗址（上）（605）
90. 五一假期即将开启，一大波美景已经安排...（1967）
91. “喜迎二十大 永远跟党走 奋进新征程”2022年全国大学生延安暑期社会实践专项活动合辑（二）（513）
92. 陕西扎实开展党史学习教育纪实（3347）
93. 宝塔山的见证——探寻中国共产党的延安岁月（1898）
94. 人民日报 | 发扬延安精神和红旗渠精神，全面推进乡村振兴——习近平总书记陕西延安和河南安阳考察重要讲话引发热烈反响（4692）
95. 【枣园知道】读书分享会丨第91期（47）
96. 陕西命名一批青少年教育基地，多数为文博场馆（1664）
97. 陕西命名一批青少年教育基地，榆林多个文博场馆上榜（471）
98. ​西安博物院等131个单位被命名为陕西省青少年教育基地（624）
99. 学党史 强信念 跟党走 || 北伐战争和革命力量的发展之北伐的胜利进军（140）
100. 陕西教育党员干部用延安精神滋养初心 把为民服务落在解决痛点难点上（2628）
101. 陕西命名一批青少年教育基地，商洛这些单位入选！（159）
102. 延安精神永放光芒（1185）
103. 壮观！延安的这些美景，切实体现了陕北的风土人情！值得一看~（41）
104. 写在十九届中央纪委六次全会召开之际（7.1万）
105. 自我革命：跳出历史周期率的第二个答案（4.1万）
106. 学党史 强信念 跟党走 || 北伐战争和革命力量的发展之工农革命运动的高涨（二）（151）
107. 奋进新时代”主题成就展图片荟萃（中央综合展区第五单元）（51）
108. 落实党史教育，践行延安精神：校史馆讲解员赴枣园旧址开展党史教育活动（1060）
109. 中国共产党人的精神谱系丨张思德精神（580）
110. 毛泽东曾言：“这是中国历史上从来未有的奇迹……”（5.2万）
111. 又见达州“身影”！中央纪委国家监委工作回顾来了（506）
112. 【枣园知道】百年党史大家学丨第180期《党的故事我来讲 特辑63——延安中学生活散记：把酱油和醋都吃光》（119）
113. 聚焦｜自我革命：跳出历史周期率的第二个答案（249）
114. 学党史 强信念 跟党走||延安青年讲师团线上开讲啦！（873）
115. 全国5A景区名单出炉，陕西占了11个，快来看看！（1万）
116. 学党史 强信念 跟党走 || 五卅运动和大革命高潮的兴起之五卅运动和大革命高潮的兴起之五党对中国革命基本问题的探索（二）（654）
117. 【学习卡片】全国爱国主义教育示范基地（陕西篇）：延安革命纪念地之枣园革命旧址（6）
118. 有理想 有血性 有本事——各地开展少年军校热血少年营活动（10万+）
119. 再见了妈妈，今天我就要去参加研学了（1361）
120. 延安的枣园革命旧址（194）
121. 全程高铁动车！今起，内江人去延安只需7.5小时——（7692）
122. 基层动态｜沈阳理工大学团员青年赴延安开展暑期社会实践（355）
123. 延安精神丨走进圣地延安：每年数百万人到访，在这里汲取信仰的力量（4943）
124. 红色专项实践展示⑤ | 缅怀革命先烈，践行雨花精神，南农青年这样做（401）
125. 弘扬延安精神 创新文旅发展（1044）
126. 青联之声 ｜ 省青联举办陕西省暨延安市青联委员学习党的二十大精神专题培训班（1750）
127. 这里是落脚点，也是出发点（1.3万）
128. 今起，成都⇌延安动车直达！（3535）
129. 【党史学习教育】坚定历史自信，走好新的赶考之路——论巩固拓展党史学习教育成果（309）
130. 中国纪检监察报头版报道了我省这项工作！（5600）
131. 【红都培训动态】炼化公司项目建设指挥部2022年度党务及宣传培训班圆满结束（99）
132. 《光明日报》头版头条刊登我校三位同学党史学习教育访谈录（3702）
133. 西延高铁进度条再刷新，赶早咥碗洋芋擦擦可好？（2903）
134. 记者节，让我们为自己喝彩（1.3万）
135. 宝鸡钢管公司团委开展沉浸式学习培训！（106）
136. “不忘跟党初心 牢记青春使命”-党的精神谱系研学专项实践活动侧记（148）
137. ​经历诸多人生第一次，20名大学生为何而来？（7982）
138. 延安——枣园革命旧址（162）
139. 延安精神：枣园革命旧址（521）
140. 延安枣园革命旧址游记（901）
141. ☀延安枣园革命旧址旅游攻略（129）
142. 陕西延安枣园革命旧址 全国革命传统教育的重要基地之一（36）
143. 延安枣园革命旧址 满园春色关不住（484）
144. 枣园革命旧址：秋高气爽景色好 游人如织看变化（728）
145. 延安枣园革命旧址开放公告（420）
146. 晋陕游记（11）：延安枣园革命旧址景区（264）
147. 【讲革命故事 忆峥嵘岁月】延安枣园革命旧址：感红色历史 承延安精神（1174）
148. 延安游记　之 枣园革命旧址（325）
149. 枣园革命旧址，延安绿化最好的园子了（38）
150. 各界延安【旅游】探访延安枣园革命旧址（9）
151. 延安红色调研活动 | 枣园革命旧址（236）
152. 延安枣园革命旧址，毛主席在延安住地，一个园林式的革命旧址（4806）
153. 瞻仰延安枣园革命旧址……回眸当年伟人足迹（217）
154. 枣园革命旧址：我要去延安 感受革命情怀（769）
155. 延安12345 | 延安枣园革命旧址闭园公告（212）
156. 园林式的革命纪念地延安枣园革命旧址（14）
157. 延安枣园革命旧址，一个园林式的革命纪念地，景色秀丽，风光迷人（图片）（126）
158. 延安红色革命教育——枣园革命旧址（29）
159. 延安之旅-枣园革命旧址（1047）
160. 红色延安之—枣园革命旧址（56）
161. “传承红色基因，缅怀革命先烈”——走进延安枣园革命旧址（123）
162. 社会实践｜【延安之旅】第一站——枣园革命旧址（41）
163. “延安枣园革命旧址——探寻红色记忆 践行延安精神”（182）
164. 枣园革命旧址：初雪素裹美如画（862）
165. 革命旧址---延安枣园（77）
166. 延安红色之行（五）：枣园革命旧址（59）
167. 枣园革命旧址献礼祖国快闪活动（21）
168. 延安枣园革命旧址管理处开展 “五一”节前安全大检查、环境卫生大扫除（73）
169. 红色延安|杨家岭革命旧址＆枣园革命旧址（210）
170. 延安12345 | 关于调整杨家岭、枣园革命旧址参观游览路线的公告（300）
171. 【党建培训】全省公安消防部队党建工作培训班系列报道之十三——组织参观延安枣园革命旧址（214）
172. 【暑期实践】参观延安枣园革命旧址和宝塔山，长征之路代代传，红色精神永发光（165）
173. 参观枣园革命旧址（213）
174. 延安12345 | 枣园革命旧址游客服务中心投用啦（160）
175. 【浪陕西】之壮丽山川延安（1925）
176. 延安枣园革命旧址管理处开展春季消防安全培训（23）
177. 瞻精神丰碑 走学习之路 | 延安分队参观枣园革命旧址（66）
178. 在延安新华广播电台、枣园革命旧址感悟峥嵘岁月（325）
179. 【游客看延安 】忆峥嵘岁月 看时代变迁（184）
180. 游枣园革命旧址 | 延安大学校史服务团成立十周年（459）
181. 延安枣园革命旧址管理处 开展健康教育专题培训（8）
182. 延安枣园革命旧址管理处开展消防安全知识培训与应急疏散演练（22）
183. 来一次延安之旅，追忆激情燃烧的岁月（2552
184. 以人民为中心！“洋记者”访革命圣地延安(（2.2万）
185. 延安枣园革命旧址管理处2018消防训练（61）
186. 【文联动态】“不忘初心、牢记使命”美丽延安主题摄影采风活动在枣园革命旧址成功举办（1210）
187. “不忘初心、牢记使命”美丽延安主题摄影采风活动在枣园革命旧址成功举办（123）
188. 工会主席学习之旅 | 第三站走进杨家岭和枣园革命旧址，实地学习延安精神（448）
189. 推荐 | 去延安，找寻理想之光（5100）
190. 观枣园革命旧址--市关工委全体学员走进延安学习培训（98）
191. 【纪实】来看这群青年记者眼中的延安与窑洞（1554）
192. 5.23，延安枣园旧址广场变成了红色的海洋！（5020）
193. 延安调查队党支部组织党员干部赴枣园革命旧址开展“讲初心”主题党课活动（37）
194. 延安枣园革命旧址闭园公告（244）
195. 这份延安市内两日游攻略送给你！带你周末玩转延安~（3169）
196. 军网记者延安行｜今天我们“遇见”张思德（1120）
197. 延安入选红色旅游全国十大热门目的地（2564）
198. 枣园革命旧址（6）
199. 2020.6.27 陕西省延安枣园革命旧址（180）
200. 快讯！赣州、遵义、延安在人民大会堂联合发布..（1.7万）
201. 【盛夏清音】在圣地延安，中央统战部、中央网信办组织网络大咖共话使命担当！（4731）
202. 延安 枣园（45）
203. 寒假去哪儿 | 清华师生开展“重走总书记初心之路”社会实践 陈旭参加梁家河支队实践并为师生讲党课（1万）
204. **视频：174**

**延安革命纪念馆**

1. 云访革命旧址丨一起打卡延安革命纪念馆（16）
2. 延安革命纪念馆：用革命文物传递延安精神（198）
3. 延安革命纪念馆走进洛川县老庙镇宣讲党的二十大精神（648）
4. 延安革命纪念馆举办学习贯彻党的二十大精神及习近平总书记来延考察重要讲话精神专题宣讲会（1215）
5. 云观革命旧址丨走进延安革命纪念馆（24）
6. 而今迈步从头越（1731）
7. 家乡党史我来说｜贺璐娜：延安革命纪念馆，追寻红色记忆（1551）
8. 红色革命之旅--延安革命纪念馆（102）
9. 【牢记嘱托，砥砺奋进】延安革命纪念馆走进老庙镇宣讲党的二十大精神（625）
10. 重走红色征程，赓续革命薪火——延安大学党员之家全体成员参观延安革命纪念馆（522）
11. 踏访延安革命纪念馆，重回令人难忘的火红岁月（135）
12. 云参观 | 智能制造学院学生党支部“云游”延安革命纪念馆（558）
13. 游记丨打卡红色中国——延安革命纪念馆（245）
14. 百个红色经典“云”参观（68）| 延安革命纪念馆（112）
15. 红色印迹·云参观 | 第十一站 • 延安革命纪念馆（129）
16. 喜报|延安革命纪念馆入围“首届全国博物馆志愿服务典型案例”和“2022年度全国文博社教百强案例（295）
17. 红色革命圣地--延安革命纪念馆（36）
18. 延安革命纪念馆参观（393）
19. 红色研学旅行——延安革命纪念馆（570）
20. 学习党的二十大丨华山监狱组织全狱党员民警线上参观延安革命纪念馆（249）
21. 延安革命纪念馆——国家5A景区（95）
22. 新疆艺术学院开展“追寻红色足迹 赓续红色血脉”线上参观延安革命纪念馆活动（580）
23. 光照千秋的延安精神——延安革命纪念馆（178）
24. 红色印记 | 革命圣地 红色灯塔——延安革命纪念馆（206）
25. 【建党百年 云游博览】延安革命纪念馆（53）
26. 革命圣地 红色灯塔——延安革命纪念馆（93）
27. 【展馆巡游】光照千秋的延安精神——延安革命纪念馆（581）
28. 烽火炼初心|走进延安革命纪念馆（146）
29. 春暖花开| 赴一场延安革命纪念馆之约（931）
30. 参观延安革命纪念馆）（146）
31. 延安革命纪念馆，回望我们的2021（1184）
32. 延安革命纪念馆参加第六届丝绸之路国际博览会（1105）
33. 【延安革命纪念馆国庆特辑】热潮涌动 家国情浓（1346）
34. 云参观︱延安革命纪念馆（61）
35. 铭记历史 珍爱和平｜延安革命纪念馆举行纪念抗日战争胜利77周年活动（928）
36. 延安革命纪念馆，我们来了！（176）
37. 蹄疾步稳以致远 勇毅笃行谱新篇——延安革命纪念馆召开2022年半年工作推进会（1289）
38. 延安革命纪念馆新馆设计（5652）
39. 打卡红色地标｜第十二站：延安革命纪念馆（73）
40. 红色建筑镌党史 | 延安革命纪念馆（188）
41. 2022，博物馆的力量（932）
42. 2022年陕西省革命文物融入高校思政教育培训班在延安革命纪念馆开班（1464）
43. 延安革命纪念馆参加第九届“中国博物馆及相关产品与技术博览会”（427）
44. 延安革命纪念馆传达学习全市文物工作会议精神（264）
45. 136. 延安革命纪念馆（124）
46. 巍巍宝塔 初心永照——走进延安革命纪念馆（48）
47. 思政 | 以建筑回忆党史——延安革命纪念馆（148）
48. 建党百年系列|延安革命纪念馆正式开馆！（1351）
49. 【百年党史】延安革命纪念馆（67）
50. 延安革命纪念馆全新亮相，展示光照千秋的延安精神（248）
51. 红色延安——参观延安革命纪念馆（354）
52. 延安革命纪念馆召开疫情防控与安全工作部署会（919）
53. 五月盛会（1876）
54. 延安革命纪念馆召开专题会议传达学习习近平总书记在瞻仰延安革命纪念地时发表的重要讲话精神（2462）
55. “伟大历程——中共一大至七大巡展（延安站）”在延安革命纪念馆隆重开幕（1715）
56. 时代共进 人民共享 | 延安革命纪念馆“红色故事”开启高校思想政治教育新模式（971）
57. 红色传记《史唯然》发行暨座谈会在延安革命纪念馆召开（2166）
58. 致敬革命圣地 光耀延安精神 ——延安革命纪念馆随笔（1432）
59. 青”听百年团史 | 第90期：延安革命纪念馆（13）
60. 延安革命纪念馆喜获第三届全国革命文物保护利用“十佳案例”（1238）
61. 陕西革命旧址云上展（三十五）| 延安革命纪念馆（73）
62. 筑梦忆党丨建筑经典学党史第6期一一延安革命纪念馆（14）
63. 延安革命纪念馆：在传承红色基因中提升青少年精神素养（463）
64. 【学习二十大】学习延安精神——线上参观延安革命纪念馆（169）
65. 延安革命纪念馆文物征集公告（1787）
66. 暑期实践｜回顾延安岁月（一）：参观延安革命纪念馆（35）
67. 党的旗帜高高飘扬 —— 组雕《旗帜》手稿落户延安革命纪念馆（2106）
68. 寻访红色建筑 | 第42期：延安革命纪念馆（71）
69. 学党史，筑信仰 | 延安革命纪念馆（182）
70. 活动总结 | 教育学部初级团校线上参观延安革命纪念馆活动圆满结束（308）
71. 一堂特殊的党史学习教育课——吴为山雕塑作品中的党史讲座在延安革命纪念馆举行（1321）
72. Day 1 延安革命纪念馆（26）
73. 抗疫一线绽芳华——延安革命纪念馆防疫抗疫纪实（1093）
74. 线上走访延安革命纪念馆——打卡革命圣地，解读延安精神（470）
75. 知文物学百年党史之见证（二十一）|延安革命纪念馆（39）
76. 展览数字化 居家也能看——延安革命纪念馆推出系列云端数字展览馆（1302）
77. 延安革命纪念馆举办学习贯彻习近平总书记重要讲话精神专题宣讲会（901）
78. 红色旅游大讲堂 | 延安革命纪念馆（93）
79. 纪念馆里学党史 共度三八妇女节（1137）
80. 红色文物系列 | 延安革命纪念馆（156）
81. “传承红色基因 赓续红色血脉——陕西省博物馆协会纪念馆专业委员会2021年年会”视频会议召开（933）
82. 踔厉奋发新时代 欢庆六一向未来：共读孩子们的来信（782）
83. 延安革命纪念馆展厅文字翻译（402）
84. 社会实践 | 生命科学学院暑期社会实践活动纪实——走进延安革命纪念馆数字博物馆（108）
85. 号外！延安革命纪念馆基本陈列荣获“全国博物馆十大陈列展览精品奖（1336）
86. 延安革命纪念馆如何走向“共享”（138）
87. 延安革命纪念馆关于闭馆的通知（4279）
88. (四) 延安革命纪念馆（136）
89. 延安革命纪念馆庆祝建党99周年暨建馆70周年系列活动之二（3125）
90. 延安旅记二 参观延安革命纪念馆（289）
91. 我到访过的纪念馆之二 | 延安革命纪念馆（135）
92. 延安革命纪念馆举行党的十九届六中全会精神进镇村宣讲活动（998）
93. 延安革命纪念馆（75）
94. 红色景点丨延安革命纪念馆（167）
95. 延安革命纪念馆召开会议 传达学习党的十九届六中全会精神（973）
96. 延安革命纪念馆宣教部荣获“全国巾帼文明岗”称（184）
97. 第一站 延安革命纪念馆（20）
98. 学党史系列活动之三：参观延安革命纪念馆（33）
99. 红色回响——延安革命纪念馆（180）
100. “华侨旗帜 民族光辉——陈嘉庚生平事迹展” 在延安革命纪念馆隆重开展（592）
101. 连环画里的中国优秀经典故事（486）
102. 西行漫记--延安革命纪念馆（62）
103. 喜迎建党百年 云游红色展馆 | 线上参观延安革命纪念馆（494）
104. 筑牢安全防线 确保节日安全——延安革命纪念馆开展“五一”假期安全大检查专项工作（757）
105. “学习用典——中国优秀经典故事全国连环作品展”开展仪式在延安革命纪念馆举行（1095）
106. 张锦秋院士作品：延安革命纪念馆（1486）
107. 赓续红色血脉 弘扬长征精神—— 延安革命纪念馆“长征故事我来讲”主题社教活动（1134）
108. 延安革命纪念馆 《伟大历程——中共中央在延安十三年历史陈列》开展公告（4653）
109. “特殊一课”——杜军民同志文物捐赠仪式在延安革命纪念馆举行（1946）
110. “延安·延安——吴为山革命主题雕塑作品展”开展仪式在延安革命纪念馆举行（1731）
111. 爱国强音 礼赞百年|延安革命纪念馆组织全体党员开展主题党日活动（1484）
112. 兰爱平指导延安革命纪念馆第一党支部 党史学习教育专题组织生活会（1321）
113. 走进延安革命纪念馆 追忆红色峥嵘岁月（28）
114. 延安革命纪念馆召开党史学习会（1215）
115. 景点巡礼丨延安革命纪念馆（23）
116. 暑期实践 | 走进延安革命纪念馆 重温党史 感悟延安精神（878）
117. 盘点陕西·延安的景点，你去过延安革命纪念馆吗（9）
118. 延安革命纪念馆|2020年，我们这样走过（81）
119. 延安精神：延安革命纪念馆（388）
120. 参观延安革命纪念馆心得体会（1）（199）
121. 五一国际劳动节延安革命纪念馆参观须知）（1726）
122. 延安精神淬灵魂 “双拥”花开谱新篇 ——延安革命纪念馆走进武警延安支队共庆“八一”（2075）
123. 习近平带领中共中央政治局常委瞻仰延安革命纪念地（5.7万）
124. 延安革命纪念馆恢复开放公告（3298）
125. 天平带你看延安｜延安革命纪念馆（191）
126. 创建国家5A级旅游景区(三）延安革命纪念馆（40）
127. 习近平带领中共中央政治局常委赴陕西延安瞻仰延安革命纪念地（10万+）
128. 延安旅游该看啥？追寻“红色”记忆：延安革命纪念馆（26）
129. 喜报|“首届全国博物馆志愿服务典型案例”和“2022年度全国文博社教百强案例”延安革命纪念馆入围（14）
130. 省委宣讲团来延安革命纪念馆宣讲党的二十大精神（1041）
131. 风景名胜：延安革命纪念馆（376）
132. 【打卡红色地标】走进延安革命纪念馆（227）
133. 【延安专辑】延安革命纪念馆（130）
134. 一群“特殊”的讲解员，走进了延安革命纪念馆！（716）
135. 不忘初心 牢记使命”主题教育基地 | 延安革命纪念馆（37）
136. 陕西红色地标（一）——延安革命纪念馆（26）
137. 延安革命纪念馆“五一”参观须知（218）
138. 延安革命纪念馆简介（36）
139. 文化旅游节|红色川陕：延安革命纪念馆（68）
140. 红色景点 | 延安革命纪念馆（155）
141. 一次延安行，一生延安情—延安革命纪念馆（23）
142. 红色文化大讲堂|延安革命纪念馆（122）
143. 【庆祝建团百年·100个团史故事87】延安革命纪念馆（14）
144. 长征纪念馆纪念地 ——延安革命纪念馆（28）
145. 延安革命纪念馆有哪些特殊物件？| 零时差Talk（1170）
146. 延安革命纪念馆开展消防安全宣传月主题活动（205）
147. 云学习·云参观 | VR全景·带您参观延安革命纪念馆（20）
148. 学习党的二十大，传承红色延安精神——城市交通运行保障党支部线上参观延安革命纪念馆（90）
149. 亲切的关怀 殷殷的嘱托——周恩来总理与延安革命纪念馆纪实（1750）
150. 一大拨老师，今天走进了延安革命纪念馆！（2133）
151. 参观延安革命纪念馆最新防疫须知（2340）
152. 延安美景之一 | 延安革命纪念馆（46）
153. 纪念抗战胜利75周年延安革命纪念馆馆藏精品艺术展盛大开启（1834）
154. 延安革命纪念馆：让文物“活”起来 讲述延安故事（1036）
155. 延安革命纪念馆献礼5.18国际博物馆日——聚力融合 包容多样（134）
156. 云游红色纪念馆——【陕西篇】延安革命纪念馆（62）
157. 党史展馆巡展I延安革命纪念馆（12）
158. 【延安之行】延安革命纪念馆（64）
159. 【鲁班有约】现代与传统的结合——延安革命纪念馆（126）
160. 红色故旅 | 革命圣地——延安革命纪念馆（166）
161. “云游”红色展馆-走进延安革命纪念馆（66）
162. 【学在路上】延安革命纪念馆概况（83）
163. 【走遍中国】延安革命纪念馆（27）
164. 【旅游资讯】延安革命纪念馆！（18）
165. 百年风华 再启新程 ——延安革命纪念馆干部职工收看庆祝中国共产党成立100周年大会直播（1186）
166. 延安的旅游景区（景点、演出）之三：延安革命纪念馆（33）
167. “五一”小长假 延安革命纪念馆游客接待创新高（190）
168. 延安革命纪念馆32件革命文物修复完成 将于6月中旬与市民见面（80）
169. 心系延安 （三）延安革命纪念馆（83）
170. “此生无憾为中华——马海德与他的革命导师宋庆龄”文物图片展在 延安革命纪念馆开展（2402）
171. 云旅行|延安革命纪念馆（172）
172. 百期党史学习（65）|延安革命纪念馆（12）
173. 延安革命纪念馆展陈概念初步设计方案征集公告（444）
174. 红色圣地——延安革命纪念馆（422）
175. 西北之行第5篇——延安革命纪念馆（261）
176. 国家文物局副局长关强赴延安革命纪念馆检查指导工作（1249）
177. 延安革命纪念馆春节期间开放指南（200）
178. 重磅！陕西唯一，全国15家，延安革命纪念馆入选！！！（532）
179. 延安革命纪念馆 红色建筑镌党史（1757）
180. 延安革命纪念馆召开2022年度工作会议（1055）
181. 【纪念馆巡礼】延安革命纪念馆（132）
182. 祖国华诞 | 这样的延安革命纪念馆 你见过吗？（330）
183. 《铸魂——延安时期的从严治党》大型专题展览隆重开幕（2260）
184. 三分钟云参观革命遗址(25)：延安革命纪念馆（59）
185. 走进延安 || 参观延安革命纪念馆（264）
186. 延安之旅-延安革命纪念馆（1875）
187. 喜报｜延安革命纪念馆在2020陕西省文博系统讲解比赛中喜获佳绩！（1726）
188. 薛耀军、兰爱平调研指导延安革命纪念馆陈列提升改造工作（812）
189. 喜报 | 延安革命纪念馆宣教部荣获“全国巾帼文明岗”称号（1307）
190. 讲好精品党课 献礼建党百年——延安革命纪念馆举办党史学习教育系列活动（1357）
191. 延安革命纪念馆全新亮相 对观众开放| 小青小美说新闻（886）
192. 薛耀军调研指导延安革命纪念馆陈列改造工作（793）
193. 延安革命纪念馆入选中华民族文化基因库（一期）红色基因库首批试点单位（56）
194. 延安革命纪念馆党委组织党员干部参观陕北三战三捷纪念馆（191）
195. ​“建党百年，我学党史，我来讲”第八期：延安革命纪念馆（55）
196. 新闻联播丨习近平带领中共中央政治局常委瞻仰延安革命纪念（10万+）
197. 献礼建党100周年｜“唱支山歌给党听”（1214）
198. 延安革命纪念馆里红歌汇成河，温馨而感动！（314）
199. 十四运会和残特奥会火炬传递延安市区站起跑仪式 在延安革命纪念馆举行（1938）
200. “革命文物保护修复工作站”挂牌仪式在延安革命纪念馆举行（167）
201. 延安革命纪念馆党委召开2020年度民主生活会（584）
202. 延安革命纪念馆——这是一个浓缩延安革命历史的地方（83）
203. 逐梦之旅第三日——参观延安革命纪念馆，重温延安革命光辉历史（170）
204. 薛耀军赴延安革命纪念馆检查指导工作（378）
205. 延安革命纪念馆邀您一起观看2021《赢在博物馆》（40）
206. 延安革命纪念馆党委组织全体党员干部开展主题党日活动（1842）
207. 延安革命纪念馆又得了一件宝贝！（514）
208. 延安革命纪念馆召开党委（扩大）会议学习习近平总书记来陕考察重要讲话重要指示（1102）
209. 延安革命纪念馆有哪些特殊物件？（305）
210. 习近平总书记带领中共中央政治局常委瞻仰延安革命纪念地（5018）
211. 赶紧到延安革命纪念馆来，有惊喜等着你哦！（484）
212. 延安革命纪念馆荣获中国建筑学会建筑创作大奖(2009-2019)（1108）
213. 2021年三下乡 | “不忘初心忆延安”之延安革命纪念馆（55）
214. 延安革命纪念地管理局对我馆2021年度目标责任完成情况进行考核（1078）
215. 红色延安之—革命纪念馆（34）
216. 从这里出发 ——延安革命纪念馆（191）
217. 铁木尔 • 达瓦买提爱国主义诗歌书画全国巡展（延安站） 在延安革命纪念馆隆重开幕（1949）
218. DAY.7 延安革命纪念馆，随着历史感受革命精神（71）
219. 寻访延安革命纪念馆：延安精神永放光芒（1121）
220. 延安革命纪念馆深入洛川县老庙镇过村开展乡村振兴调研工作（111）
221. “乡”约三秦 | 走进延安革命纪念馆（103）
222. 祝福祖国“最强音”，在延安革命纪念馆响起！（730）
223. 延安革命纪念馆入选红色基因库试点单位（1272）
224. 党史风华 | (二十六) 重要理论起源地——延安革命纪念馆（65）
225. 延安革命纪念馆 举办“纪念长征胜利八十周年”红歌联唱活动（1584）
226. 研究中心副科级以上干部参观延安革命纪念馆（131）
227. 铭记|国家级抗战纪念设施、遗址名录-延安革命纪念馆（74）
228. 不到延安革命纪念馆，就不知道啥叫游人如织！（1890）
229. 激情！一群萌宝延安革命纪念馆广场唱红歌！（4323）
230. 习近平在瞻仰延安革命纪念地时强调（12）
231. 延安市延安革命纪念馆之新民主主义的模范试验区（50）
232. 分享一篇文章（56）
233. 延安纪念馆（33）
234. 用好红色资源 凝聚奋进力量——陕西深入开展社会主义核心价值观宣传教育（29）
235. 【云游】云游红色教育基地 学习红色文化精神——新疆总队喀什支队各级开展“云参观”红色教育基地（168）
236. 【治国理政金句】习近平论延安精神的精髓（36）
237. 延安革命纪念馆全景）（87）
238. 【红色宣推】——延安革命纪念馆（42）
239. 走进延安革命纪念馆（15）
240. 南京大屠杀死难者国家公祭日悼念活动在延安革命纪念馆举行（1660）
241. 第6期 走进延安革命纪念馆（50）
242. 延安革命纪念馆参观游客剧增（91）
243. 【今日关注】奋斗共圆中国梦（10）
244. 【清沐市幼 声阅廉洁】——永济市市直幼儿园清廉学校建设推进活动之教师篇（三）（98）
245. 【每日学习】奋斗共圆中国梦（4）
246. 【理响大名----学习宣传贯彻党的二十大精神专栏】奋斗共圆中国梦（28）
247. 【博译党建】​李村璞教授应邀为我院师生作党的二十大精神宣讲（84）
248. 学习贯彻二十大 | 奋斗共圆中国梦（28）
249. 每日学习要点｜奋斗共圆中国梦等（15）
250. 分享一篇文章。（3）
251. **视频：153**
