# Supplementary material for: The network characteristics of classic red tourist attractions in Shaanxi province, China
Source: PLoS One. 2024 Mar 29;19(3):e0299286. doi: 10.1371/journal.pone.0299286 (PMC10980247; doi:10.1371/journal.pone.0299286)
Supplement: S6 File — (DOCX) [file pone.0299286.s008.docx]

**八路军西安办事处纪念馆**

1. 揽月人~旅游游记、八路军西安办事处纪念馆——红色教育基地（发布时间：2022-10-19）、（观看：660 点赞：14）
2. 小阿黑的日常、四合院里的红色纪念馆、（2022-1-10一样）、（点赞：18 喜欢：3 观看：1392）
3. Zhao030407、八路军西安办事处纪念馆、（2021-7-6、2021-5-18）、（点赞：4 喜欢：1 观看：678）
4. 一个老兵mc、s、（2022-9-5一样）、（点赞：5 观看：257）
5. 风清扬hwj、（2020-6-1、2020-5-31）、（点赞15 观看：1820）
6. 清风若溪、八路军西安办事处纪念馆、（2018-12-20、2018-12-16）、（点赞：36 喜欢：1 观看：1.1万、评论：2）
7. 月下独酌——并州君、八路军西安办事、（2022-1-8、2019-2-10）（点赞：4 喜欢：1 观看：431）
8. WH1962\抗战期间的记忆|八路军西安办事处、（2022-10-4、2022-10-3）（点赞：98 喜欢：6观看：1624、评论：56）
9. 阿萨隆、八路军西安办事处纪念馆、（2021-9-11、2019-6-11）（点赞：15 喜欢：1观看：2498、评论：6）
10. DQH长安、八路军西安办事处纪念馆（2020-4-17一样）（点赞：16 喜欢：2观看：4137评论：0）
11. 牛奶海、（2019-10-21、2019-9-28）、（点赞：36 评论：1 观看：5646）
12. 风清扬hwj、（2020-6-2、2020-5-31）、（点赞：17 观看：1687
13. 刘闲人、（2022-5-17、2022-5-13）、（点赞：3 观看：293）
14. 风清扬hwJ、（2020-6-2、2020-5-31）、（点赞：14 观看：1167）
15. WH1962、（2019-10-18一样）、（点赞：68 评论：2 喜欢：4 观看：7033）
16. 谁叫我是小、、、（2022-2-24一样）、（点赞：8 喜欢：2 观看：1082）
17. 风清扬hwJ、（2020-6-1、2020-5-31）、（点赞：15 观看：523）
18. 多拉爱梦67、（2019-12-16、2016-3-23）、（点赞：13 观看：701）
19. 魅力西安、**攻略**、（点赞：228 评论：4 喜欢：2 ）
20. 风清扬hwJ、（2020-6-2、2020-5-31）、（点赞：17 喜欢：1 ）
21. 左左xians、（2021-7-8、2021-6-20）、（点赞：29 评论：1 ）
22. Sengcar、（2019-3-10一样）
23. Chuntian、（2019-8-28一样）、（点赞：14 评论：2 ）
24. 驻村干部、（2019-11-20、2019-11-15）、（点赞：9）
25. M13\\\\798、（2021-10-8、2021-10-5）、（点赞：11 评论：1 喜欢：1）
26. 嘉祥春秋、（2017-11-7一样）、（点赞：2 ）
27. LMC2009、（2020-11-30、2017-3-26）、（点赞：31 喜欢：1 ）
28. 风清扬hwJ、（2020-6-29、2020-6-1）、（点赞：11、观看：1034）
29. M19\\\\832、（2019-8-29一样）、（点赞：17 评论：2 喜欢：1 观看：1611）
30. WECH、、15、（2016-11-27一样）、（点赞：3 观看：429）

**携程：“西安事变”纪念馆**

1. 王柒（三水）、（2022-9-22、2020-9-11）、（点赞：16 评论：2 喜欢：3 观看：1219）
2. 明洵、（2021-10-14一样）、（点赞：6 评论：3 观看：1153）
3. 大项、（2022-6-16一样）、（点赞：4 观看：411）
4. 热爱生活的雨桐、（2022-10-18一样）、（点赞：7 喜欢：1 观看：237）
5. 遇见茶小乖、（2019-9-28一样）、（点赞：85 评论：2 喜欢：7观看：1.4万）
6. Lmc2009、（2021-3-29一样）、（点赞：28 评论：1 喜欢：8观看：4748）
7. M34、、、986、（2022-5-7一样）、（点赞：4 观看：237）
8. M20\\277\、、（2021-10-15、2021-10-12一样）、（点赞：7 观看：284）
9. Karl2008、（2021-7-27、2021-5-11）、（点赞：7 观看：208）
10. 黎荷、（2021-1-15、2020-12-13）、（点赞：8 观看：421）
11. 无敌、、读、**攻略**、（点赞：23 喜欢：2观看：2150）
12. Karl2008、（2021-7-26、2021-5-11）、（点赞：8 观看：294）
13. 车晓刚、（2022-6-14、2022-5-19）、（点赞：5 观看“339）
14. 影客、（2021-5-11、2021-5-8）、（点赞：15 评论：3 喜欢：2观看：2406）
15. 大唐小狐狸、（2021-8-29一样）、（点赞：15 评论：3 喜欢：2 观看：1964）
16. 刘闲人、（2022-5-17、2022-5-13）、（点赞：3 观看：294）
17. 吴、珍、（2019-12-31一样）、（点赞：8 观看：364）
18. 一个北京人在、（点赞：4 观看：190）、
19. 一个北京人在、（2021-12-29、2021-11-20）、（点赞：4 观看：205）
20. 刘闲人、（2011-5-21、2022-5-20）、（点赞：2 观看：331）
21. 梦的光与影、（2020-8-4、2020-5-1）、（点赞：10、观看：1050）
22. 十年磨刀2229、（2019-7-19、2018-7-27）、（点赞：15、观看：3948）
23. 十年磨刀2229、（2019-2-6、2018-7-26）、（点赞：3 评论：1 观看：340）
24. 林隐隐、（2019-9-25、2019-5-24）、（点赞：16 喜欢：1 观看：1155
25. 翱翔2004803、（2021-11-27一样）、（点赞：9 观看：749）
26. LLHCY、（2019-8-21、2019-8-11）、（点赞：12 评论：1 观看：920）
27. MIXIGE、（2019-7-30、2019-4-5）、（点赞：12 评论：1 观看：766）
28. 寻找未知角度、（点赞：4 观看：242）
29. 李拾遗、、（点赞：4观看：236）
30. Im-、、（2022-10-10一样）、（点赞：3 ）

**2-汉中市川陕革命根据地纪念馆**

1. 赵赵呀.（点赞：37 喜欢：6 观看：2235）
2. AKA阿童木、（2020-4-20一样）、（点赞：39 评论：3 喜欢：4观看：4764）
3. 清风若溪、（2019-7-1、拍摄时间：2019-6-30）、（点赞：50 评论：6 喜欢：2 观看：1500）
4. Duang、（点赞：4 观看：220）

**延安革命纪念馆**

1. 习惯、、你、（2022-6-24一样、）、（点赞：6 观看：511）
2. Ch、、09、（2022-10-19一样）、（点赞：70 评论：1 喜欢：3 观看：1095）
3. 一个北京人、、（2021-12-27、2021-11-20）、（点赞：3 观看：243）
4. V 、、、vi、（2021-3-21一样）、（点赞：32 喜欢：3 观看：5841）
5. 务实、、（2022-4-11一样）、（点赞：6 观看：220）
6. 爱旅行的巴扎黑、（2022-6-21一样）、（点赞：102 评论：16 喜欢：16 观看：8191）
7. 唐公子、（2019-5-16、2019-5-3）、（点赞：43 评论：7 喜欢：3 观看：7099）
8. 森林迷、、（点赞：539 评论：18 喜欢：105 观看：9853）
9. 、、向导、李、、（当点赞：14 评论：1 观看：879）
10. 热心、、、（2022-7-28一样）、（点赞：4 观看：273）
11. 菲菲、、、攻略、、（点赞：4 观看：713）
12. M46、、、72、（点赞：519 评论：9 喜欢：168 观看：1.5万）
13. 小王带你、、、（2022-7-24一样）、（点赞：5 观看：383）
14. 快乐、、（2020-10-3、2020-9-28）、（点赞：11 观看：437）
15. 沙漠之舟、、、（2021-3-28一样）、（点赞：14 评论：1 观看：742）
16. 一个北京人在、、（点赞：3 观看：226）
17. 一个北京人在、、（点赞：4 观看：206）
18. 一个北京人在、、（点赞：3 观看：213）
19. 一个北京人在、、（点赞：3 观看：216）
20. 超人、、、（2022-3-30、2022-3-29）、（点赞：3 观看：499）
21. 一个北京人在、、（点赞：4 观看：236）
22. 一个北京人在、、（点赞：2 观看：219）
23. 一个北京人在、、（点赞：2 观看：196）
24. 一个北京人在、、（点赞：4 观看：225）
25. 一个北京人在、、（点赞：4 观看：208）
26. 妞妞Ma、（（2022-7-18）、（点赞：5 观看：290）
27. Wech、、、53、（点赞：9 观看：283）
28. M21、、、564、（2022-8-15）、（点赞：4 观看：292）
29. Jin、、、（2022-8-19）、（点赞：4 观看：509）
30. 张有、、、（2022-7-3）（点赞：4 观看：732）
31. 一个北京人在、、（点赞：2 观看：218）
32. 一个北京人在、、（点赞：4 观看：217）
33. 一个北京人在、、（点赞：2 观看：222）
34. 一个北京人在、、（点赞：2观看：194）
35. 一个北京人在、、（点赞：2 观看：193）
36. 一个北京人在、、（点赞：2 观看：183）
37. 一个北京人在、、（点赞：3 观看：201）
38. 一个北京人在、、（点赞：4 观看：191）
39. 国内旅游攻略、（2021-8-3）、（点赞：13 观看：2002）
40. 面向大海、、、、（点赞：9 喜欢：1 观看：304）
41. 一个北京人在、、（点赞：4 观看：218）
42. 一个北京人在、、（点赞：4 观看：235）
43. 可以、（2021-9-27）、（点赞：19 评论：2 喜欢：1 观看：737）
44. 一个老兵、、、（（2021-10-9）、（点赞：3 观看：232）
45. 蛙蛙、、、（点赞：18 喜欢：2 观看：983）
46. M50、、、13、（2022-4-27）、（点赞：2 观看：1000）
47. 一个北京人在、、（点赞：2 观看：185）
48. 一个北京人在、（2021-11-20）、（点赞：4 观看：260）
49. 百花、、（2022-3-9）、（点赞：5 观看：885）
50. 何尚且、（2022-9-29）、（点赞：6 观看：925）
51. 一个北京人在、、（点赞：4 观看：209）
52. 一个北京人在、（2021-11-20）、（点赞：4 观看：273）
53. 一个北京人在、（2021-11-20）、（点赞：3 观看：239
54. 蓝狐、（2021-7-21）、（点赞：7 观看：293）
55. 一个北京人在、2021-11-20、（点赞：4 观看：241）
56. 一个北京人在、2021-11-20、（点赞：4 观看：229）
57. 一个北京人在、2021-11-20、（点赞：4 观看：279）
58. 一个北京人在、2021-11-20、（点赞：2 观看：187）
59. 一个北京人在、（点赞：4 观看：198）
60. 一个北京人在、2021-11-20、（点赞：2 观看：243）
61. 一个北京人在、2021-11-20、（点赞：2 观看：224）
62. 多年以后（2021-12-15）（点赞：10 观看：1347）
63. 陕西、、张珊珊、（点赞：8 观看：1448）
64. 一个北京人在、2021-11-20、（点赞：2 观看：187）
65. 梦想旅店、（点赞：45 欧伦：5 观看：1683）
66. 眼镜的旅行、（2022-9-18）、（点赞：3 观看：612）
67. 一个北京人在、2021-11-20、（点赞：3 观看：246）
68. M50、、、27、（（2022-4-7）、（点赞11 喜欢：4 观看：2986）
69. 西安天马、、（点赞：9 喜欢：1 观看；1967）
70. 一个北京人在、2021-11-20、（点赞：3 观看：239）
71. 天马行空、（点赞：2 观看：182）
72. 陕西、、、（点赞：8 观看：881）
73. 牛奶海、（（2021-9-29）、（点赞：4 观看：242）
74. 毛毛旅行者、（2022-10-26）、（点赞：5 观看：277）
75. 达文西的旅行、（点赞：4 观看：184）
76. 边吃边看看、（点赞：157 评论：1 评论：29 观看：6396）
77. JK、、、（2019-9-9）、（点赞：51 评论：1 喜欢：2 观看：3288）
78. 陕西天美、、、（点赞：8 观看：1144）
79. 一个北京人在、2021-11-20、（点赞：3 观看：232）
80. 一个北京人在、2021-11-20、（点赞：2 观看：199）
81. 203、、、76、（2021-4-21）（点赞：4 观看：314）
82. 天若有情、、（2020-9-22）、（点赞：12 观看：460）
83. 一个北京人在、2021-11-20、（点赞：4 观看：245）
84. 一个北京人在、2021-11-20、（点赞：2 观看：221）
85. 张益达、、（2021-5-10）、（点赞：9 喜欢：1 观看：361）
86. M30、、、、65、（2021-4-23）、（点赞：17 观看：361）
87. Wech、、、（2022-6-10）、（点赞：4 观看：235）
88. 一个北京人在、2021-11-20、（点赞：4 观看：221）
89. 朱稀、、（2021-6-13）、（点赞：8 观看：381）
90. CF、、、90、（2020-9-25）、（点赞：5 观看：394）
91. 散步的猫、、（2021-6-29）、（点赞：32 评论：3 观看：2433）
92. Ch、、tian、（2021-7-30）、（点赞：8 观看：283）
93. 爱旅游的拾、（（2022-10-2）、（点赞：4 观看：601）
94. 小娅、、（2019-9-14）、（点赞：79 评论：4 喜欢：4 观看：5658）
95. 义薄云天、、（2021-7-29）、（点赞：8 观看：591）
96. M23、、、72、（2021-10-3）、（点赞：5 评论：3 观看：316）
97. 菜菜的后花园、（点赞：56 评论：17 喜欢：1观看：791）
98. 半罐小可乐、**攻略、**（点赞：47 评论：4 喜欢：7 观看：2460）
99. Zhao、、（2021-5-16）、（点赞：4 观看275）
100. 天道酬勤、、（2021-6-20）、（点赞：2 观看241）
101. 旅行者看风景、（点赞：56 评论：3 喜欢：5 观看1830）
102. 善待人生、、（2021-7-3）、（点赞：7 喜欢：1 观看328）
103. 雅、、521、（2021-7-26）、（点赞：7 观看：255）
104. 尼可、、**攻略**、（点赞：86 喜欢：22 观看：2626）
105. 木、、栗子、（2016-10-1）（点赞：11 喜欢：1 观看：377）
106. 环游世界的探讨、（2022-3-29）、（点赞：15 喜欢:1 gk :3111）
107. 单身游、（2020-6-28）、（点赞：8 观看：353）
108. IF2007、（2020-1-14）、（点赞：5 观看;188）
109. 风情云游、、（2022-7-17）、（点赞：5 观看：1435）
110. LY、、（2016-5-28）、（点赞：32 评论：4 观看：2635）
111. Tirp陕西、**攻略、**（点赞：26 评论：3 喜欢：4 观看：2424）
112. M30、、、65、（2020-4-23）、（点赞:15 gk :447）
113. Pei、、、hen、（2020-7-17）、（点赞：10 观看：475）
114. 王小王、（2021-9-20）、（点赞：2 评论：3 观看：307）
115. 自由旅游游人、（2019-10-9）、（点赞：21 观看：1观看：4101）
116. 、、范仲淹、（2019-7-20）、（点赞：12 评论：3 观看：519）
117. Wech、、、81、（2019-8-21）、（点赞：12 评论：3 观看：1028
118. 开心皓、、（2019-12-15）、（点赞：10 观看：756）
119. Sure1012、（2020-5-21）、（点赞：4 观看：237）
120. 圣托马斯、、（2021-10-10）、（点赞：3 评论：3 观看：335）
121. Cycat20、（2018-8-29）、（点赞：35 评论：4 观看：6125）
122. 晓斌、**攻略、**（点赞：65 评论：3 喜欢：13 观看1659）
123. 似水流、、、03、（2020-7-18）、（点赞：9 观看：602）
124. 看那天边的云、**攻略**、（点赞：494 评论：10 喜欢：162 观看：1.1万）
125. Lgoto、（2019-10-17）、（点赞：15 评论：1 观看：554）
126. Wang、（2019-10-4）、（点赞：17 喜欢：1 观看：589）
127. 祥云、、88、（2020-5-3）、（点赞：10 观看：434）
128. Oflair、（2019-10-12）、（点赞：15 观看：502）
129. Gzw001、（2020-1-7）、（点赞：73 评论：7 喜欢：3 观看：5996）
130. M32、、、74、（2021-7-6）、（点赞：29 评论：4 观看：610）
131. Linglizi、（2021-8-11）、（点赞：9观看1180）
132. 诺伊、、（2019-8-17）、（点赞：12 评论：1 观看3841）
133. 舞、、、光、（2018-7-9）、（点赞：12 评论：3 观看：660）
134. D37、、04、（2019-11-23）、（点赞：12 观看：486）
135. Wang 、（2019-10-4）、（点赞：20 观看：597）
136. 由我决定、（2019-7-9）、（点赞：13 评论：5 观看:499)
137. 芳芳外婆、（2021-12-12）、（点赞：3 观看269）
138. 王小王329、（2021-9-20）、（点赞:4 观看：245）
139. 七与多、（2019-8-8）、（中的：14 观看504）
140. WB2、、、46、（2021-10-22）、（点赞：5 观看：216）
141. tang yeye、（2019-7-7）、（点赞：12 评论：3 观看：585）
142. Les、、88、（2014-6-8）、（点赞：13 观看：3891）
143. Tuguang、、（2022-4-24）、（点赞：50 评论：6 喜欢：6 观看：1300）
144. 黔东南、、（2019-3-6）、（评论：1 观看：3824）
145. 流浪者旅拍、（2018-2-27）、（点赞：94 评论：7 喜欢：7 观看：1.3万）
146. 面向大海、、（2019-6-28）、（点赞：11 观看：341）
147. 芳芳外婆、（2021-12-29）、（点赞：3 观看：252）
148. M27、、、08、（2019-9-28）、（点赞：15 观看：476）
149. M23、、02、（2021-7-2）、（点赞：19 评论：3 喜欢：1 观看;1003）
150. Looo、、（2019-10-11）、（点赞：12 观看：484）
151. 臭co、（2019-5-23）、（点赞;6 观看：249）
152. 当地向导、、勇、（2019-9-24）、（点赞：15 观看：562）
153. 三三的歌、（2021-6-18）（点赞：11 观看：1180）
154. 北冥、、（2019-1-28）、（观看：198）
155. 明湖的心、（点赞：13 喜欢：1 观看：340）
156. Mech、、99、（2021-7-28）、（点赞：8 喜欢：1 观看）
157. 我是一只爱吃鱼的、（2019-5-19）、（点赞：6 观看：）
158. 东方、雪、（2012-9-7）、（点赞：12 观看：3923）
159. 默冬天、、（2019-8-29）、（点赞：15 评论：4 观看：939）
160. 一抹蔚蓝、（2021-10-13）、（点赞：4 观看：）
161. Chuntian、（2019-8-6）、（点赞：15 评论：4 观看：517）
162. Chuntian、（2021-7-30）、（点赞：8 观看：285）
163. M29、、026、（2019-1-31）、（点赞：3 观看：239
164. 非你不渴、（2020-6-20（点赞：14 评论：2 观看：1571）
165. 秋实的、、日记、（点赞：33 评论：1 喜欢：1 观看：3809）
166. 小荟带你、、攻略、（点赞：17 观看：4319）
167. 151、、、20（（2022-10-15）、（点赞：3 评论：1 观看：）
168. 呆妹的旅行日记（2021-8-3）、（点赞：26 评论：1 观看：3238）
169. 陈老师1982、（2020-10-9）、（点赞：6 观看：501）
170. 斯蒂芬、（2021-4-30）、（点赞：13 喜欢：1 观看：3277）
171. LMT、、02、（2019-8-31）、（点赞7 观看：1920）
172. M49、、（21020-10-21）、（点赞：8 喜欢：1 观看：361）
173. 小郝、、（点赞：7 喜欢：1 观看：238）
174. 带你旅行、（2021-7-18）、（点赞：51 评论：1喜欢：5 观看：3949）
175. 大熊乖乖、（2014-8-19）、（点赞：3 观看：405）
176. 小米1023、（2017-4-1）、（点赞：6 观看：140）
177. 咸菜、、（2017-4-2）、（点赞：观看：980）
178. 无疆、、（2017-4-18）、（点赞：4 观看：204）
179. 毛蛋、、、（2017-5-23）、（点赞：9 观看：268）
180. Meren、（2017-7-31）、（点赞：2 观看：1291）
181. 行天下、、（2017-12-26）、（点赞：10 观看：4039）
182. 202、、、258、（2018-3-3）、（点赞：10 观看：321）
183. M29、、026、（2019-2-1）、（点赞：21 观看：1148）
184. 更行更远的我、（2018-8-11）、（点赞：49 评论：1 喜欢：3 观看：840）
185. 喵陛下、（2019-5-2）、（点赞：7 观看：4879）
186. M36、、27、（2019-10-3）、（点赞：14 观看：512）
187. 瓦、西亚、、（2019-9-28）、（点赞：12 观看：436）
188. JXL、、、om、（2019-9-23）、（点赞：41 评论：1 喜欢：1 观看：2847）
189. 碎冰1314、（2019-10-5）、（点赞：16 喜欢：1 观看：512）
190. 莎莎、、（2019-9-20）、（当中：13 观看：4010）
191. ZY张张、（2019-6-25）、（点赞：14 观看;977）
192. 流浪的风景、（2017-7-11）、（点赞：11观看：526）
193. 小张很酷、（2019-11-26）、（点赞：10 观看：689）
194. 真的汉子、（2019-5-26）、（点赞：8 观看：768）
195. 刚哥、、（2019-12-14）、（点赞：14 评论：2 观看：4198）
196. Wang、（2019-10-4）、（点赞：17 喜欢：1 观看：591）
197. 风中、、（2011-8-23）、（点赞：5 观看:352）
198. 达帕、、（2019-11-5）、（点赞：53 评论：5 喜欢：1 观看；2745）
199. -CF、、、90、（2020-9-25）、（点赞：5 观看：396）
200. 金哥、、（2019-10-2）、（点赞：23 评论：1 观看：790）
201. Sugar、、（2019-10-31）、（点赞：11 观看：441）
202. M30、、65（2020-4-23）、（点赞：17 观看：521）
203. 风中、、（2021-4-20）、（点赞：11 观看：768）
204. 小肥羊、、（点赞：9 观看：4571）
205. 风之痕（2019-10-17）、（点赞：23 评论：1 观看：4867）
206. 一路游来、、（2020-10-13）、（点赞：10 观；438）
207. 奋斗、、（点赞：3 观看：324）
208. 峰行天下、（2019-3-27）、（点赞：7 观看;800）
209. 肉肉、、（2015-9-28）、（点赞：11 观看：1936）
210. 神奇在路上、（点赞：231 评论：6 喜欢：37 观看：1万）

**王家坪革命旧址**

1. IMC2009，王家坪革命旧址、（2021-5-31、2021-5-17）,、（点赞：43 喜欢：4 观看：4692）
2. 菜菜的后花园、（2021-5-18、2021-4-30）、（点赞：273 评论：28 喜欢：15 观看：1.4万
3. 余满的旅行、延安王、、肃然起敬、（2022-1-27一样）、（点赞：10、观看：1129）
4. Jinyu、、1、（（2022-8-18一样）、）、（点赞：1 观看：1044）
5. 热心市民、、（2022-7-29一样）、（点赞：4 观看：958）
6. 一个北京人、（2022-1-18、2021-11-20）、（点赞：2 观看：297）
7. 爱吃土豆的波妞、（2021-6-10一样）、（点赞：26 评论：3 喜欢：2 观看：2768）
8. 散步的猫胖胖、（2021-6-29一样）、（点赞：16 喜欢：2 观看：375）
9. 205、、、1、（2022-7-31一样）、（点赞：2 观看：152）
10. 面向大海、、（2021-7-27、2019-6-28）、（点赞：10 观看：1161）
11. Netcat、（2020-8-16一样）、（点赞：16 观看：475）
12. 游遍祖国、、、（2019-8-6、2019-7-10）、（点赞：13 评论：3 观看：944）
13. 李、波、（2011-11-2一样）、（点赞：4 观看：163）
14. 我的包包、、（2018-12-21一样）、（点赞：183 评论：22 喜欢：8 观看：1万）
15. 滇、、（2020-9-20、2020-9-18）、（点赞：10 评论：1 观看：3436）
16. 牛奶海、（2021-12-20、2021-9-20）、（点赞:3 观看：250）
17. 清风、、、（2020-1-31、2016-7-17）、（点赞：28 评论：2 观看：844）
18. Zhao、、、7、（2021-6-19、2021-5-16）、（点赞：3 观看：243）
19. Ling i、（2020-8-12、2020-8-11）、（点赞：13 评论：1 观看：2940）
20. 芳芳外婆、（2021-12-13、2021-12-12）、（点赞：2 观看：253）
21. 娴雅小溪、（2020-2-1、2016-7-17）、（点赞：18 观看：534）
22. 日月同辉、（2020-5-17、2020-5-2）、（点赞：18 观看：1115）
23. 金哥、、、（2019-10-9、2019-10-2）、（点赞：232 评论：30 喜欢：14 观看：1.4万）
24. 牛奶海、（2021-12-20、2021-9-20）、（点赞3 观看：187）
25. 牛奶海、（2021-12-20、2021-9-20）、（点赞：2 观看：199）
26. 一个北京、、（2022-1-17、2021-11-20）、（点赞：4 观看：173）
27. 牛奶海、2021-12-20、2021-9-20）、（点赞：2 观看：195）
28. 林、、斌、（2019-1-6、2018-12-30）、（点赞：8 观看：4309）
29. 牛奶海、2021-12-20、2021-9-20）、（点赞：4 观看：212）
30. 牛奶海、2021-12-20、2021-9-20）、（点赞：1 观看：197）
31. 仁者、、（2019-7-20一样）、（点赞：12 评论：5 观看：480）
32. 一个北京人、、（2022-1-17、2021-11-20）、（点赞：3 观看：180）
33. 多年以后、、（2021-12-22、2121-12-15）、（点赞：2 观看：256）
34. 一个北京人、、（2022-1-19、2021-11-20）、（点赞：4 观看：202）
35. 牛奶海、2021-12-20、2021-9-20）、（点赞：2 观看：189）
36. 牛奶海、2021-12-20、2021-9-20）、（点赞：2 观看：181）
37. 牛奶海、2021-12-20、2021-9-20）、（点赞：4 观看：208）
38. 牛奶海、2021-12-20、2021-9-20）、（点赞：3 观看：219）
39. 牛奶海、2021-12-20、2021-9-20）、（点赞：3 观看：253）
40. 牛奶海、2021-12-20、2021-9-20）、（点赞：4 观看：209）
41. 一个北京人、（2022-1-16、2021-11-20）、（点赞；4 观看：225）
42. 一个北京人、（点赞；3 观看：174）
43. 看IXDE \\\家、（2022-6-23一样）、（点赞：3 观看：850）
44. Wang、、（2019-10-6、2019-10-4）、（点赞：15 喜欢：1观看：493）
45. Wang、、（2019-10-6、2019-10-4）、（点赞：21 评论：1 1观看：3958）
46. 爱吃土豆的波妞、（点赞：15 观看：367）
47. 一个北京人、（点赞；2 观看：182）
48. 个北京人、、（2022-1-15、2021-11-20）、（点赞：2 观看：168）
49. 个北京人、、（2022-1-16、2021-11-20）、（点赞：2 观看：210）
50. 个北京人、、（2022-1-14、2021-11-20）、（点赞：3 观看：210）
51. 个北京人、、（2022-1-19、2021-11-20）、（点赞：3 观看：208）
52. 个北京人、、（2022-1-14、2021-11-20）、（点赞：4 观看：236）
53. 个北京人、、（2022-1-18、2021-11-20）、（点赞：4 观看：229）
54. 泽、5、（2018-7-4一样）、（点赞：6 观看：1473）
55. 面向大海、、（点赞：8 喜欢：1 观看：315）
56. 旅行家、、哥、（2019-12-21、2019-11-25）、（点赞：25 观看：613）
57. Wang、、（2019-10-6、2019-10-4）、（点赞：22 喜欢：1 观看：3955）
58. 喵陛下、（2019-5-2一样）、（点赞：7 观看：4876）
59. 一个北京人、（点赞；3 观看：183）
60. 雪中火、、、（（2021-8-9、2020-7-9）、（点赞：5 观看：242）
61. 好精致、（（2020-9-26、2020-9-9））、（点赞：7 观看：1875）
62. 一个北京人、、（2022-1-15、2021-11-20）、（点赞：4 观看：190）
63. 130、、、71、（2019-2-9一样）、（观看：262）
64. 一个北京人、（点赞：2 观看：171）
65. 一个北京人、（点赞：3 观看：196）
66. 一个北京人、（点赞：2 观看：195）
67. 一个北京人、（点赞：3 观看：194）
68. 巡、、、灯、（2019-5-30一样）、（点赞：10 观看：4100）
69. 陈老师、、2、（2020-10-9一样）、（点赞：5 观看：392）
70. 天、、博士、（2019-4-28一样）、（观看：231）
71. Gzw、、、1、（2020-1-25、2020-1-6）、（点赞：73 平凉：11 喜欢：4 观看：5787）
72. Jin、、、11、（2022-8-18一样）、（点赞：1 观看：1045）
73. 臭、、麻、（2019-5-23一样）、（点赞：6 观看：289）
74. LMT、、、2、（2021-9-28、2021-8-31）、（点赞：7 观看：1918）
75. 无、、行、（2017-4-18一样）、（点赞：3 观看：128）
76. 一个北京人、、（点赞：2 观看：184）
77. 兰、、步、（（2018-3-8一样））、（点赞：4 观看：3978）
78. 一个北京人、、（点赞：4 观看：206）
79. 陈大大、、（2019-10-26、2019-10-25）、（点赞：27 观看：4041）
80. 我是一只、、、猫、（2019-5-19一样）、（点赞：6 观看：192）
81. M10、、、1、（2018-12-10一样）、（点赞：1 观看：229）
82. 189、、、、（2019-5-29一样）、（点赞：6 观看:297）
83. 唐媛、（2019-12-4、2019-10-13）、（点赞：24 平凉：2 观看：4035）
84. 东、、树叶、（2019-8-3、2019-8-2）、（点赞：10 平凉：1 喜欢：1 观看：3916）
85. 一个北京人、、（当中：4 观看：201）
86. 东、、树叶、（2019-8-3、2019-8-2）、（点赞：11 评论：5 观看：479）
87. 一个北京人、、（点赞：3 观看：184）
88. 一个北京人、、（当中：4 观看：196）
89. 一个北京人、、点赞：2 观看：172）
90. 游古城、（2019-2-11一样）、（点赞：5 评论：1 观看：174）
91. 一个北京人、、（点赞：2 观看：183）
92. 晴、、0、（2019-10-7一样）、（点赞;11 观看：430）
93. Jan2019、（2019-5-8、2018-8-7）、（观看：3831）
94. 、、墨、（2019-9-14、2019-9-13）、（点赞：18 观看;630）
95. 唐公子、（2019-5-9、2019-5-4）、（点赞：32 评论：3 喜欢：2观看：6168）
96. 一个北京人、（点赞：3 观看：176）
97. 哈哈、、之、（2020-3-19一样）、（点赞：15 喜欢：1 观看：457）
98. 一个北京人、、（2022-1-16、2021-11-20）、（点赞：2 观看：211）
99. 旅行家、、（2019-11-20、2019-11-18）、（点赞：35 评论：1 喜欢：2 观看：4495）
100. Wang、、（2019-10-6、2019-10-4）、（点赞：15 喜欢：1 观看：494）
101. 一个北京人、、（2022-1-18、2021-11-20）、（点赞：4 观看：230）
102. 一个北京人、、（2022-1-14、2021-11-20）、（点赞：4 观看：237）

**枣园革命旧址**

1. 遇见美景、快乐欣赏、走进、、记忆、（发布：2020-6-29）、（点赞：101 评论：17 喜欢：7 观看：4587）
2. 猴子不候、（2022-7-25一样）、（点赞：5 观看：826）
3. Cherry2009、（2022-10-1）、（点赞：89 评论：5 喜欢：2 观看：1485）
4. Jinyu9711、延安枣园革命旧址、（2022-8-17一样、）（点赞：5 观看：751）
5. 菜菜的后花园、五一、、旧址、（2021-5-17）、（点赞：334 评论：21 喜欢：23 观看：2.1万 ）
6. 张北草原、、河南到陕西、、（点赞：12 喜欢：2 观看：881）
7. 开心帮胖嘟嘟大玩家、延安枣园、（2022-6-24、2022-6-23）、（点赞：2 观看：691）
8. M21...564、枣园革命旧址、（2022-8-17、2022-8-16）、（点赞：2 观看：1022）
9. 晨语、枣园革命旧址、（2022-2-21一样）、（点赞：12 观看：1361）
10. 奇乐旅行、北纬37、、见过么？攻略、（2022-8-17一样）、（点赞：26 喜欢：9 观看：2993）
11. M30...9465、（2022-8-7一样）、（点赞：9 喜欢：2 观看：340）
12. 古来豪杰、红色——两日游、（2021-8-2、2021-7-30）、（点赞：35 评论：3 喜欢：7 观看：4782）
13. LC春虹、（2021-6-15）、（点赞：2 观看：387）
14. 无语的、、288、（2022-6-28一样）、（点赞：3 观看：197）
15. New200th、走进、、旧居、（2021-6-8一样）、（点赞：20 喜欢：1 观看：1705）
16. 延安。。、李延峰、（点赞：7 观看：372）
17. 牛奶海、朱德旧居、（2021-12-17、2021-9-21）、（点赞：4 观看：246）
18. 牛奶海、毛泽东旧居、（2021-12-17、2021-9-21）、（点赞：4 观看：268）
19. 老鹰在飞翔、革命圣地延安、（2022-9-15）、（点赞：6 观看：541）
20. 爱旅游的喵喵阳、红色、、盎然、（2021-3-19）、（点赞：53 评论：8 喜欢：3 观看：3022）
21. 牛奶海：张闻天旧居、（2021-12-17、2021-9-21）、（点赞：3 观看：290）
22. 旺市杜甫、（2021-10-22、2021-10-21）、（点赞：3 观看：198）
23. 盐食计、（点赞：3 观看：178）
24. M475...362、中央、、变迁、（2022-1-3、2021-11-21）、（点赞：4 ）
25. 风之痕、（（2019-10-19）、（点赞：59 评论：3喜欢：3 观看：3145）
26. 旅行家、、（点赞：22 喜欢：1 观看：1246）
27. Imc、、（（2021-5-16））、（点赞：25 观看：1924）
28. 我就说俺家乡好、、（点赞：3 观看：221）
29. 我就说俺家乡好、、（点赞：4 观看：216）
30. 我就说俺家乡好、（2022-1-11、2021-11-21）、（点赞：2 观看：194）
31. 我就说俺家乡好、、（点赞：2 观看：169）
32. Imc、、（（2021-5-16））、（点赞：17 欧伦：喜欢：1 观看：2410）
33. Ye.、、、（2020-9-14）（点赞：13 喜欢：1 观看：620）
34. 提琴、、、（2021-2-5）、（点赞：9 观看：1480）
35. 海风、（2021-5-9）、（点赞：5 观看：394）
36. JJ77、、（2022-9-9）、（点赞：5 观看：393）
37. 梓、、（（2019-10-27））、（点赞：4 观看：519）
38. LMT、、、（2019-8-31）、（点赞：3 观看：787）
39. 老当益壮、、、（2021-5-25）、（点赞：6 观看：402）
40. 面向大海、、（2019-6-28）、（点赞：9 观看;1083）
41. 散步的猫胖胖、（2021-6-29）、（点赞：18 观看：454）
42. Zhao0304、、（2021-5-17）、（点赞：9 观看：254）
43. Linglizi 、（2020-8-11）、（点赞：10 评论：1 观看：2208）
44. 我也爱边牧、（2020-7-26）、（点赞：4 观看：355）
45. Yanyan19、、（2020-10-23）、（点赞：9 观看：457）
46. 网中的人、（2020-10-20）、（点赞：17 观看：1656）
47. 陈老师、、（2020-10-9）、（点赞：8 观看：384）
48. 韦、、索尼、（2020-9-25）、（点赞：10 观看：1040）
49. M93、、、（2020-9-1）、（点赞：11 观看：423）
50. 日月同辉、（2020-5-2）、（点赞：13 观看：1029）
51. 冯、勇、（（2021-10-13）、（点赞：6 观看：300）
52. 旅行达人、、（点赞：20 观看：1171）
53. 冯川老古、（2021-4-22）、（点赞：87 评论：9 喜欢：8 观看：3881）
54. 延安..李、、（点赞：10 观看：405）
55. 好精致、（2020-9-8）、（点赞：7 喜欢：1011）
56. 我就说俺家乡好、（2022-1-11、2021-11-21）、（点赞：4 观看：238）
57. 牛奶海、（2021-9-21）、（点赞：3 观看：316）
58. 爱旅游的喵喵（2021-3-19）、（点赞：53 评论：8 喜欢：3 观看：3022）
59. 娴雅小溪、（2016-7-17）、（点赞：12 观看：455）
60. 我就说俺家乡好、（2022-1-11、2021-11-21）、（点赞：4 观看：197）
61. 牛奶海、（2021-9-21）、（点赞：4 观看：246）
62. 牛奶、（2021-9-21）、（点赞：4 观看：268）
63. 牛奶海、（2021-9-21）、（点赞：3 观看：290）
64. Imc 2009、（2015-10-4）、（点赞：76 评论：6 喜欢：2 观看：5059）
65. 牛奶海、（2021-9-21）、（点赞：3 观看：318）
66. 我就说俺家乡好、（2022-1-11、2021-11-21）、（点赞：3 观看：160）
67. 我就说俺家乡好、（2022-1-11、2021-11-21）、（点赞：4 观看：228）
68. 我就说俺家乡好、（2022-1-11、2021-11-21）、（点赞：34 观看：196）
69. 我就说俺家乡好、（2022-1-11、2021-11-21）、（点赞：2 观看：186）
70. 何尚且、（2021-9-29）、（点赞：3 评论：1 观看：229）
71. 我就说俺家乡好、（2022-1-11、2021-11-21）、（点赞：4 观看：155）
72. 我就说俺家乡好、（2022-1-11、2021-11-21）、（点赞：3 观看：156）
73. 我就说俺家乡好、（2022-1-11、2021-11-21）、（点赞：2 观看：197）
74. 我就说俺家乡好、（2022-1-11、2021-11-21）、（点赞：4 观看：202）
75. 我就说俺家乡好、（2022-1-11、2021-11-21）、（点赞：4 观看：189）
76. 我就说俺家乡好、（2022-1-11、2021-11-21）、（点赞：4 观看：262）
77. 我就说俺家乡好、（2022-1-11、2021-11-21）、（点赞：4 观看：214）
78. 我就说俺家乡好、（2022-1-11、2021-11-21）、（点赞：3 观看：221）
79. 我就说俺家乡好、（2022-1-11、2021-11-21）、（点赞：2 观看：195）
80. 我就说俺家乡好、（2022-1-11、2021-11-21）、（点赞：2 观看：187）
81. 我就说俺家乡好、（2022-1-11、2021-11-21）、（点赞：2 观看：228）
82. 我就说俺家乡好、（2022-1-11、2021-11-21）、（点赞：3 观看：199）
83. 我就说俺家乡好、（2022-1-11、2021-11-21）、（点赞：4 观看：215）
84. 我就说俺家乡好、（2022-1-11、2021-11-21）、（点赞：3 观看：200）
85. 我就说俺家乡好、（2022-1-11、2021-11-21）、（点赞：3 观看：245）
86. 松、、皇帝、（2020-8-21）、（点赞：11 观看：513）
87. 我就说俺家乡好、（2022-1-11、2021-11-21）、（点赞：4 观看：228）
88. 牛奶海、（2021-9-21）、（点赞：2 观看：217）
89. 牛奶海、（2021-9-21）、（点赞：4 观看：236）
90. 牛奶海、（2021-9-21）、（点赞：3 观看：274）
91. 牛奶海、（2021-9-21）、（点赞：3 观看：246）
92. 牛奶海、（2021-9-21）、（点赞：3 观看：267）
93. 牛奶海、（2021-9-21）、（点赞：4 观看：228）
94. 牛奶海、（2021-9-21）、（点赞：3 观看：245）
95. 牛奶海、（2021-9-21）、（点赞：3 观看：201）
96. 爱吃土豆的波妞、（2021-6-10）、（点赞：31 评论：3 观看：1926）
97. 班超、、（2021-6-20）、（点赞：24 评论：3 喜欢：2 观看：1800）
98. 清风若、、（2016-7-7）、（点赞：24 观看：563）
99. IMC2009、（2015--10-4）、（点赞：76 评论：6 喜欢：2 观看：5059）
100. Cherry、、（2020-1-12）、（点赞：18 评论：1 观看：1799）
101. 云山、、（2021-10-1）、（点赞：10 评论：5 喜欢：1 观看：578）
102. 我就说俺家乡好、（2022-1-11、2021-11-21）、（点赞：4 观看：2235）
103. 我就说俺家乡好、（2022-1-11、2021-11-21）、（点赞：2 观看：177）
104. 我就说俺家乡好、（点赞：3 观看：201）
105. 牛奶海、（2021-9-21）、（点赞：4 观看：257）
106. 牛奶海、（2021-9-21）、（点赞：2 观看：178）
107. 牛奶海、（2021-9-21）、（点赞：3 观看：256）
108. 我就说俺家乡好、（点赞：2 观看：200）
109. 我就说俺家乡好、（点赞：4 观看：219）
110. 我就说俺家乡好、（点赞：6 观看：211）
111. 我就说俺家乡好、（点赞：3 观看：200）
112. 我就说俺家乡好、（点赞：2 观看：191）
113. 我就说俺家乡好、（点赞：3 观看：192）
114. 我就说俺家乡好、（点赞：2 观看：190）
115. 我就说俺家乡好、（点赞：2 观看：208）
116. 我就说俺家乡好、（点赞：4 观看：206）
117. 我就说俺家乡好、（点赞：4 观看：1906）
118. 我就说俺家乡好、（点赞：5 观看：218）
119. 我就说俺家乡好、（点赞：5 观看：214）
120. 我就说俺家乡好、（点赞：2 观看：193）
121. 我就说俺家乡好、（点赞：3 观看：196）
122. 我就说俺家乡好、（点赞：3 观看：222）
123. 我就说俺家乡好、（点赞：3 观看：212）
124. 我就说俺家乡好、（点赞：2观看：213）
125. 我就说俺家乡好、（点赞：2观看：189）
126. 我就说俺家乡好、（点赞：3 观看：192）
127. 我就说俺家乡好、（点赞：3 观看：218）
128. 我就说俺家乡好、（点赞：4 观看：241）
129. 我就说俺家乡好、（点赞：2 观看：193）
130. 我就说俺家乡好、（点赞：3 观看：249）
131. 我就说俺家乡好、（点赞：2 观看：200）
132. 我就说俺家乡好、（点赞：2 观看：204）
133. 我就说俺家乡好、（点赞：2 观看：191）
134. 我就说俺家乡好、（点赞：3 观看：173）
135. 我就说俺家乡好、（点赞：4 观看：236）
136. 我就说俺家乡好、（点赞：4 观看：246）
137. 唐媛、、（2019-10-13）、（点赞：14 评论：1 观看:1873）
138. 旺市杜甫（2021-10-21）、（点赞：3 观看：207）
139. 我就说俺家乡好、（点赞：3 观看：205）
140. 蜥爸、（2021-9-10）、（点赞：8 喜欢：1 观看：262）
141. 开心皓、、（2019-12-15）、（点赞：7 观看：3969）
142. 向导、、张勇、**攻略**、（点赞：9 评论：1 观看：2545）
143. 地域男爵、（2019-12-6）、（点赞：7 观看：1427）
144. 远方戎马、、（2021-4-9）（点赞：10 观看：420）
145. 蕙质兰心、、（2019-12-5）、（点赞：9 观看：606）
146. 面向大海、、（点赞：10 观看：289）
147. 西溪老翁、（2005-12-7）、（点赞：10 观看：1033）
148. 叽里咕噜、、（2021-2-21）、（点赞：7 观看：288）
149. M49、、、76（2021-11-2）、（点赞：6 喜欢：1 观看：309）
150. M48、、、16（2021-7-25）、（点赞：11 喜欢：1 观看：305）
151. 天涯海角、、（2021-7-5）、（点赞：7 观看：250）
152. 东方逸、、（2017-9-5）、（点赞：12 观看：562）
153. 203、、、76（2021-4-21）、（观看：123）
154. 维多利、、（2021-2-19）、（点赞：5 喜欢：1 观看：345）
155. 200、、29、（2020-10-23）（点赞：9 评论：1 观看：512）
156. 老鹰在飞翔、、（2020-3-11）、（点赞：10 观看：428）
157. 赫本、、（2020-12-29）、（点赞：6 观看：396）
158. M41、、86（2019-11-7）、（点赞：15 观看：1516）
159. 旅行家、、（2020-10-5）、（点赞：25 喜欢：1观看：541）
160. 一蓑烟雨、、（2020-11-26）、（点赞：7 观看：1160）
161. 马、、博（2020-11-26）、（点赞：6 观看：468）、
162. 陈世、、（点赞：9观看：422）
163. 滇国、、、（2020-9-20）、（点赞：78 评论：1 喜欢：4 观看：2.1万）
164. 臻观天下、（2020-10-28）、（点赞：6 观看：434）
165. 150、、92、（2019-10-7）、（点赞：15 评论：2 观看：726）
166. 自家江湖、（点赞：8 观看：848）
167. 大海、、27、（2020-10-4）、（点赞：18 评论：1 喜欢：1 观看：2422）
168. 我是二、、（2020-9-27）、（点赞：9 观看：422）
169. 爱在此、、（2020-8-12）、（点赞：7 观看：5192）
170. 从不犹豫、（2019-9-9）、（点赞：19 观看;623）
171. 似水流年、、（2020-7-17）、（点赞：9 观看：679）
172. Wang 、、（2019-10-4）、（点赞：16 观看：500）
173. Wang 、、（2019-10-4）、（点赞：16 观看：529）
174. Pei、、（2020-7-18）、（点赞：7 观看：496）
175. 一生旅行、、（2020-7-17）、（点赞：12 观看：495）
176. M36、、50（2019-9-9）、（点赞：16 喜欢：1观看：1900）
177. Les、、88（2014-6-8）、（点赞：11 观看：536）
178. 东方红、（2020-5-3）、（点赞：7 观看：380）
179. 王朝背影、（2020-4-13）、（点赞：10 观看：453）
180. 方寸山人、（2019-7-13）、（点赞：11 观看：416）
181. 向导张文茹、攻略、（点赞12 喜欢：2 观看：1065）
182. 向导、、张勇、（2020-2-20）、（点赞：9 观看：400）
183. 没有昵称、、（2019-10-20）、（点赞：16 评论：2 观看：437）
184. 钟爱白、、（2019-4-30）、（点赞：30 评论：5 喜欢：1 观看：4454）
185. Gzw001、（2020-1-6）、（点赞：13 评论：1 喜欢：1 观看：2785）
186. 流浪的0、（2018-8-14）、（点赞：6 观看：974）
187. Yeqiang、、（2019-10-11）、（点赞：34 评论：2 观看：5503）
188. 驴-驴-友、（2019-12-6）、（点赞:16 观看：1010）
189. 达帕、、、（2019-11-5）、（点赞：24 喜欢：1 观看：1690）
190. M22、、96、（2019-11-18）、（点赞：10 观看：379）、
191. 旅行家、、（2019-11-18）、（点赞：32 喜欢：2 观看：4148）
192. 我是苏小康、（2019-5-22）、（点赞：453 评论：49 喜欢：26 观看：3,。9万）、
193. Fg3309、（2019-10-28）、（点赞：13 观看：3874）
194. 听泉品茶、（2019-9-20）、（点赞：25 评论：1 喜欢：1 观看：3611）
195. 黑利伯格、、（2019-10-6）、（点赞：18 评论：2 观看：4781）
196. Wech、、（2019-10-23）、（点赞：15 观看：433）
197. 陈大大、、（2019-10-25）、（点赞：15 观看：499）
198. 旅游、、的出厨子、（2019-10-24）、（点赞：19 观看：3884）
199. 哈拉哈河、（2019-10-13）、（点赞：29 喜欢：1 观看：658）
200. 嘉祥春秋、（2019-10-17）、（点赞：12 观看：406）
201. Yangdg、、8（2017-4-19）、（点赞：51 评论：1 喜欢：4 观看：1484）
202. tour大鹏、（2019-6-15）、（点赞：253 评论：43 喜欢：18 观看：1.5万）
203. 醒目、（2019-9-9）、（点赞：15 观看：4026）
204. 当地向导回延安、（2019---8-29）、（点赞：81 评论：9 喜欢：6 观看：5609）
205. 问愁、（2014-9-6）、（点赞：34 评论：2 喜欢：1 观看：1611）
206. 平遥海海、（2017-5-29）、（点赞：3 观看：160）
207. 行天下、、（2017-12-25）、（点赞：9观看：417）
208. Wech、、（2018-6-19）、（点赞：11 评论：3 观看：794）
209. M10、、71、（2018-12-8）、（点赞：1评论：2 观看：338）
210. M10、、71、（2018-12-8）、（点赞：1 观看：334）
211. Vick、、（2018-12-20）、（点赞：181 评论：37 喜欢：10 观看：1,。2万）
212. M30、、58、（2019-1-6）、（gk 337）
213. 春天、、36、（2019-1-9）、（点赞：10 观看：4416）
214. 华-88 （2019-1-10）、（点赞：23 喜欢：1 观看：2717）
215. 北冥、、（2019-1-27）、（评论：1 观看：167）
216. M11、71、（2019-1-28）、（点赞：5 观看：323）
217. 剑胆、、E、（2019-1-31）、（点赞：1 观看：223）
218. 跟着老公、、（2019-2-6）、（观看252）
219. yan雨、、（点赞：4 观看：3924）
220. 一梦的旅行、、（2018-12-17）、（观看：2522）
221. 游古城、（2019-2-11）、（点赞：8 看：288）
222. 境界、哥（2019-2-22）、（点赞：11 评论4 喜欢：2 观看;2373）
223. 林晓斌、（2019-1-6）、（点赞：2 观看;544）
224. 我的包包、、（2018-12-21）、（点赞：186 评论：26 喜欢：15 观看：8921）
225. 大庆、、（2019-3-9）、（点赞：28 评论：4 观看;470）
226. 你好、（2019-3-12）、（点赞：1 观看：210）
227. 君临天下、（2019-3-13）、（点赞：4 观看;3888）
228. 太空飞、、（2019-2-10）、（点赞：4 评论：1 观看：3867）
229. 往钱、、、（2019-3-22）、（点赞：5 观看280）
230. 大老虎的、、（2019-3-24）、（观看：3824）
231. Wech、、（2019-4-2）、（观看：317）
232. Hijk、、（2019-4-9）、（观看：3832）、
233. Hijk、、（2019-4-9）、（点赞：4观看：262）
234. Hijk、、（2019-4-9）、（点赞：1观看：282）
235. 我是一只爱吃鱼、、（2019-5-19）、（点赞：6 观看：216）
236. 臭co、、（2019-5-23）、（点赞：6 观看：229）
237. 唐公子（2019-5-3）、（点赞：24 评论：1 喜欢：2 观看：2406）
238. 知足常乐、（2017-10-22）、（点赞：16 评论1 xih :3 gk :5328）
239. Hijk、、（2019-4-9）、（点赞：5 喜欢：1观看：3886）
240. LY、、（2016-5-28）、（点赞：7 评论：1 观看：343）
241. 我是一只爱吃鱼的、（2019-5-19）、（点赞：6 观看：290）
242. 与人为善、、（2019-6-20）、（点赞：7 评论：5 观看：290）
243. 踏遍、、（2019-4-9）、（点赞：12 评论：4 喜欢：1 观看：1605）
244. 由我决定(（2019-7-9）、（点赞：10 评论：6 观看：541）
245. Dabao（2013-7-9）、（点赞：16 评论：4 喜欢：1 观看：1214）
246. 孤独的、、（2019-7-10）、（点赞：17 评论：7 观看;460）
247. 萨维、、（2019-7-20）、（点赞：13 评论：2 观看：535）
248. 舞-月-（（2018-7-9）、（点赞：13 评论：2 观看：3907）
249. 武夷山、（2019-7-23）（点赞：12 评论：5 观看：682）
250. 两刷子、（2019-7-27）、（点赞：13 评论：3 观看：494）
251. 深蓝、、（2019-7-26）、（点赞16 plun :6 gk :636）
252. 风花雪月、（2019-8-1）、（点赞：13 评论：2 观看：3957）
253. 爱钱如命的、、（2019-8-4）、（点赞：20 评论：8 观看：626）
254. Yunbo、、（2019-7-7）|（点赞：17 评论：5 观看：625）
255. 星光、、（2019-5-10）、（点赞：5 观看：1289）
256. 长河落日、、（2019-7-29）、（点赞：26 评论：7 观看：1539）
257. Chuntian、（2019-8-5）、（点赞：12 评论：5 观看：535）
258. 呼呼、、（2019-5-23）、（点赞：4 评论：1 观看：2010）
259. 爱格、、（2019-7-9）、（点赞：24 、pun：3 观看：3969）
260. 博特、、（2019-6-12）、（点赞：8 观看：1362）
261. 输不起的人（2017-9-20）、（点赞：13 观看：475）
262. 喵陛下（2019-5-4）、（点赞：6 观看：2072）

**杨家岭革命旧址**

1. 我就是山野村夫、（2022-1-20、2021-11-21）、（点赞：2 观看：247）
2. 我就是山野村夫、（2022-1-18、2021-11-21）、（点赞：2 观看：188）
3. 我就是山野村夫、（2022-1-19、2021-11-21）、（点赞：3 观看：206）
4. 我就是山野村夫、（2022-1-16、2021-11-21）、（点赞：2 观看：109）
5. 牛奶海、（2021-12-16、2021-9-22）、（点赞：2 观看：215）
6. 我就是山野村夫、（2022-1-11、2021-11-21）、（点赞：4 观看：252）
7. 我就是山野村夫、（2022-1-10、2021-11-21）、（点赞：4 观看：226）
8. 我就是山野村夫、（2022-1-9、2021-11-21）、（点赞：2 观看：221）
9. 牛奶海、（2021-12-16、2021-9-22）、（点赞：2 观看199）
10. 牛奶海、（2021-12-16、2021-9-22）、（点赞：2 观看：213）
11. 我就是山野村夫、（点赞：3 观看：195）
12. 我就是山野村夫、（点赞：2 观看：213）
13. 我就是山野村夫、（2022-1-10、2021-11-21）、（点赞：3 观看：205）
14. 我就是山野村夫、（2022-1-4、2021-11-21）、（点赞：6 观看：199）
15. 我就是山野村夫、（2022-1-13、2021-11-21）、（点赞：4 观看：239）
16. 我就是山野村夫、（2022-1-15、2021-11-21）、（点赞：2 观看：216）
17. 牛奶海、（2021-12-16、2021-9-22）、（点赞：2 观看：193）
18. 牛奶海、（2021-12-16、2021-9-22）、（点赞：4 观看：201）
19. 牛奶海、（2021-12-16、2021-9-22）、（点赞：3 观看：193）
20. 牛奶海、（2021-12-16、2021-9-22）、（点赞：4 观看：210）
21. 我就是山野村夫、（2022-1-9、2021-11-21）、（点赞：4 观看：243）
22. 我就是山野村夫、（2022-1-15、2021-11-21）、（点赞：6 观看：234）
23. 我就是山野村夫、（2022-1-18、2021-11-21）、（点赞：3 观看：207）
24. 我就是山野村夫、（2022-1-13、2021-11-21）、（点赞：2 观看：192）
25. 牛奶海、（2021-12-16、2021-9-22）、（点赞：3 观看：229）
26. 牛奶海、（2021-12-16、2021-9-22）、（点赞：4 观看：203）
27. 我就是山野村夫、（2022-1-17、2021-11-21）、（点赞：6 观看：224）
28. 我就是山野村夫、（2022-1-17、2021-11-21）、（点赞：4 观看：272）
29. 牛奶海、（2021-12-16、2021-9-22）、（点赞：2 观看：233）
30. 我就是山野村夫、（2022-1-12、2021-11-21）、（点赞：4 观看：219）
31. 我就是山野村夫、（2022-1-12、2021-11-21）、（点赞：4 观看：239）
32. 我就是山野村夫、（2022-1-11、2021-11-21）、（点赞：3 观看：314）
33. 牛奶海、（2021-12-16、2021-9-22）、（点赞：2 观看：186）
34. 我就是山野村夫、（点赞：3 观看：196）
35. 我就是山野村夫、（点赞：2 观看：231）
36. 牛奶海、（2021-12-16、2021-9-22）、（点赞：2 观看：196）
37. 我就是山野村夫、（点赞：2 观看：150）
38. 我就是山野村夫、（点赞：2 观看：158）
39. 牛奶海、（2021-12-16、2021-9-22）、（点赞：2 观看：195）
40. 我就是山野村夫、（点赞：2 观看：191）
41. 我就是山野村夫、（点赞：2 观看：200）
42. 我就是山野村夫、（点赞：3 观看：196）
43. 我就是山野村夫、（点赞：6 观看：225）
44. 我就是山野村夫、（点赞：2 观看：232）
45. 我就是山野村夫、（点赞：4 观看：231）
46. 我就是山野村夫、（点赞：3 观看：170）
47. 我就是山野村夫、（点赞：4 观看：219
48. 李延、、（点赞：18 评论：3 喜欢：4 观看;）
49. 无忧、、（2016-8-18）、（点赞：6 观看：180）
50. 、、向导大同、、、（2017-1-19）、（点赞：9 评论：3 观看：601）
51. 无疆行、（2017-4-18）、（点赞：11 观看：242）
52. Setree、（2017-6-15）、（点赞：2 观看：118）
53. 邯郸、、（2018-9-7）、（点赞：3 观看：555）
54. M10、、71、（2018-12-9）（点赞：1 观看：283）
55. M10、、71、（2018-12-9）（点赞：1 观看：261）
56. 夫君君、、（2018-12-23）、（观看：177）
57. 虫出江湖、（2018-12-23）、（评论：1 观看：176）
58. 我的包包、、（2018-12-21）、（点赞：191 评论：31 喜欢:11 gk :7829）
59. 筱莲、（2018-12-23）、（点赞：325 评论：45 喜欢：18 观看：1.2万）
60. Wech、、、（2019-1-15）、（观看：224）
61. M25、、17、（2019-2-10）（评论：1 观看：139）
62. 雪宝走天涯、（2019-2-9）、（评论：1 观看：207）
63. 你好、（2019-3-12）、（点赞：1 观看：256）
64. Where、、（2019-1-8）、（点赞：5 观看：3929）
65. Yush、、（2019-3-20）、（点赞：1 评论：1 观看：213）
66. 银川、、（2019-2-17）、（点赞：141 评论：6 喜欢：6 观看：5337）
67. 候红、、、（2019-5-2）、（点赞：1 观看：131）
68. 金、、穆工、（2019-5-3）、（点赞：1 观看：233）
69. 唐公子、（2019-5-3）、（点赞：2 观看：427）
70. 知足常乐、（2017-10-22）、（点赞：17 评论：2 观看：2553）
71. 138、、、26、（2019-5-14）、（点赞：5 观看：194）
72. **北**京大胖、（2019-3-30）、（观看：745）
73. 我是一只爱吃的、、（2019-5-19）、（点赞：6 观看：177）
74. M31、、02、（2019-4-20）、（观看：199）
75. 崔小满、（2019-5-23）、（点赞：4 评论：1 观看：1183）
76. 鼎天立地（2019-6-6）、（点赞：11观看：3862）
77. 踏遍、、（2019-4-9）、（点赞：12 评论：2 观看：436）
78. M33、、78、（2019-7-6）、（点赞：10 评论：5 观看：542）
79. 荒野、、（2017-10-3）、（点赞：1 观看：178）
80. LY、、（2016-5-28）、（点赞：7 评论：2 观看：377）
81. 三晋沙龙、（2019-7-17）、（点赞：15 评论：2 观看：489）
82. 舞-月-光（2018-7-9）、（2018-7-9）、（点赞：13 评论：2 观看：638）
83. M38、、40、（2018-6-17）、（点赞：18 评论：3 喜欢：1 观看：646）
84. 长河落日、（2019-7-26）、（点赞：19 评论：3 观看：3959）
85. 婉兮、、（2019-4-25）、（点赞：3 观看：268）
86. 仙女、、、（2019-8-25）、（点赞：15 评论：2观看：1379）
87. HM、、（2019-8-11）、（点赞：11 评论：5 观看：446）
88. 大甲、、（2019-7-15）、（点赞：6 评论：3 观看：1715）
89. 薇儿、（2019-8-1）、（点赞：12 评论：3 观看：660）
90. 陕西路人、（2019-7-31）、（点赞：18 评论：4 观看：4093）
91. 魔都、、（2019-9-28）、（点赞：11 观看：375）
92. 默、、皇帝、（2019-8-30）、（点赞：46 评论：11 喜欢：4 观看：5046）
93. 爱格、、（2019-7-9）、（点赞：24 评论：2 观看：3971）
94. 由我决定、（2019-7-12）、（点赞：16 评论：6 观看：854）
95. 寻找--、（2019-8-27）、（点赞：15 评论：2 观看：856）
96. M59、、（2019-10-2）、（点赞：15 观看：493）
97. Les、、（2014-6-8）、（点赞：14 观看：404）
98. 134、、（20119-10-2）、（点赞：11 评论：2 观看：505）
99. 我是苏小康、（2019-5-23）、（点赞：37 评论：6 喜欢：1 观看：3352）
100. 武夷山、（2019-7-24）、（点赞：11 观看：478）
101. Gic、、59、（2019-10-30）、（点赞：13 观看：1208）
102. 胡杨呼呼、（2019-10-31）、（点赞：12 观看：426）
103. 老哥、、（2019-10-27）、（点赞：11 观看：495）
104. 问愁、（2015-10-1）、（点赞：49 评论：5 观看：2273）
105. 老君（2009-10-21）、（点赞：12 评论：614）
106. Tang 、、（2019-10-4）、（点赞：12 观看：514）
107. 雷雨、（2019-10-14）、（点赞：17 观看：4159）
108. 老吴1、（2019-11-11）、（点赞：12观看：3867）
109. 周舟、、（2019-9-18）、（点赞：98 评论：2 喜欢：3 观看：1万）
110. 雨路、、（2019-10-18）、（点赞：9 评论：1 观看：490）
111. Wech 、、（2019-8-24）、（点赞：11 观看：418）
112. 一如136、（2018-9-8）、（点赞：12 观看：412）
113. 开心皓、、（2019-12-15）、（点赞：8 观看：505）
114. Cherry2009、（2020-1-12）、（点赞：18 评论：1 观看：1269）
115. Dabao、、（2013-7-19）、（点赞：10 评论：2 观看：1777）
116. Wech 、、（201910-5）、（点赞：9评论：2 观看：973）
117. Wang、（2019-10-4）、（点赞：20 喜欢：1 观看：3975）
118. Wang、（2019-10-4）、（点赞：10 观看：491）
119. M14、、\76\（2019-11-2）、（点赞：7 观看：1319）
120. 、、张勇、（2020-2-20）、（点赞：11 观看：424）
121. 东方红、、（2020-5-3）、（点赞：7 观看;381）
122. Wang、（2019-8-21）、（点赞：12 评论：3 观看：3934）
123. 娟子、、（2020-6-16）（点赞：14 喜欢：1 观看：1726）
124. Hijk、、（2020-4-23）、（点赞12 观看：447）
125. 态然、（2020-6-27）、（点赞：10 观看：463）
126. Cost（2020-6-27）、（点赞：13 观看：559）
127. 似水流年、（2020-7-18）、（点赞：8 观看：527）
128. 薇、、的风、（2020-5-31）、（点赞：14 观看：1291）
129. 行、、九州、（2020-5-4）、（点赞：22 评论：3 喜欢;1 观看：1580）
130. M30、、（2020-4-23）、（点赞：13 观看：445）
131. Home 、、（2020-10-9）、（点赞：10 喜欢：1 观看：2163）
132. Sawe、、（2020-10-9）、（点赞：5 观看：379）
133. 一蓑烟雨、、（2020-11-25）、（点赞：13 喜欢：1 观看：4414）
134. 旅行家、、（2020-10-5）、（点赞：21 评论：2 喜欢：2 观看：603）
135. Yuli、、（2019-11-17）、（点赞：15 观看：395）
136. 滇国、、（2020-10-19）、（点赞：23 喜欢：1 观看：3461）
137. Han、、（点赞：8 观看：880）
138. 涛涛、、（2020-7-26）、（点赞：9 观看：1781）
139. 延安大东、、（点赞：16 评论：2 观看：1166）
140. 宁夏旅游、、（2021-5-4）、（点赞：8 观看：333）
141. 婉兮、、（2021-3-223）、（点赞：5 观看：274）
142. Han、、（2021-5-9）、（点赞：7 观看：383）
143. 唐媛、（2019-10-13）、（点赞：23 评论：2 观看：578）
144. 浪漫、、（2021-7-2）、（点赞：6 观看：826）
145. 风之痕（2019-10-19）、（点赞：8 观看：1333）
146. 狼图腾、（2020-10-1）、（点赞：4 观看：258）
147. 远方啊远方啊、、（2021-10-17）、（点赞：4 观看：232）
148. 义薄云天、、（2021-7-29）、（点赞：6 观看：310）
149. 程程、、（点赞：26 评论：1 喜欢：3 观看;1561）
150. Wangjian、、（2021-5-7）、（点赞：7观看：1450）
151. 、、张勇、（2021-8-12）、（点赞：12 观看：715）
152. Gzw001、（2020-1-8）（点赞：20 评论：1 喜欢：1 观看：1746）
153. 爱旅游的喵喵、、（2021-3-18）、（点赞：27 评论：3 喜欢：1 观看：2000）
154. 一点咨询（点赞：21 喜欢：1 观看：2568）
155. 清风若、、（2016-7-17）（点赞：24 喜欢：1 观看：1074）
156. Amyo、、（2021-10-27）、（点赞：6 喜欢：1 观看;296）
157. 达帕、、、（2019-11-5）、（点赞：53 评论：5 喜欢：1 观看：2749）
158. 瓦瓦旅行、、（点赞：2 观看：287）
159. 一只百灵鸟、、（2022-8-2）、（点赞：5 观看：418）
160. Qtrqq、、（2022-3-7）、（点赞：4 观看;226）
161. 娴雅小溪、（2016-7-17）、（点赞：13 喜欢：1 观看:442）
162. San、、、62、（2021-6-20）、（点赞：17 评论：3 观看：1430）
163. 班超、、（2021-6-18）、（点赞：31 评论：6 观看;1500）
164. 梦想旅店、（点赞{46、评论：5 喜欢：2 观看：2089）
165. 扭扭、、（点赞：4 喜欢：1 观看：440）
166. 云朵家、、（2021-6-25）、（点赞：142 评论：2 喜欢：18 观看:5770）
167. 、、李延、、（点赞：15 评论：2 喜欢：1 观看：1133）
168. Jinyu、（2022-8-21）（点赞：5 观看：530）
169. Sawe、、（2020-10-9）、（点赞;14 观看：724）
170. M46、、（点赞：519 评论：9 喜欢：168 观看：1.5万）
171. 爱吃土豆的波妞、（2021-6-10）、（点赞：20 评论：3 喜欢：1 观看：2056）
172. Xxjm、、（2022-8-31）、（点赞：3观看：244）
173. 边吃边看看、（点赞：157 评论：1 喜欢：29 观看：6420）
174. 、李延、、（点赞：18 评论：3 喜欢：4 观看：1070）
175. 金哥、、（2019-10-2）、（点赞：662 评论：41 喜欢：79 观看：5.1万
176. 陕西虎牙、（2021-11-26）、（点赞4 观看：496）
177. 、、张勇、（2022-3-1）、（点赞：7 评论：3 喜欢：1 观看：2008）
178. 可爱的二娃、（2022-9-20）、（点赞：4 观看：207）
179. 日月同辉、（2020-5-2）、（点赞：15 观看：1190）
180. 不乐不归、（2021-4-21）、（点赞：10 观看：412）
181. 、、李延、、（点赞：19 观看：1704）
182. 文艺蜀黍、、（点赞：32 评论：1 喜欢：1观看：1490）
183. Zhao0304、（2021-5-16）、（点赞：7 观看;300）
184. Linglizi、（2020-8-11）、（点赞：7 评论：1 观看：1735）
185. 游侠半仙、（2020-10-7）、（点赞：6 观看：745）
186. 毛毛旅行、（2022-10-26）、（点赞：5 观看：286）
187. 网中的人、（2020-10-20）、（点赞：22 观看：2149）
188. 我的四叶、、（2022-8-28）、（点赞：3 观看：715）
189. 菜菜的后花园、（点赞：56 评论：17 喜欢：1 观看：792）
190. Mechan、、（2021-7-28）、（点赞：8 喜欢：1 观看：412）
191. 我也爱边牧、（2020-7-26）、（点赞：7 观看：298）
192. 散步的猫胖胖、（2021-6-29）、（点赞：17 观看：452）
193. 风之痕（2019-10-19）、（点赞：7 观看：1267）
194. 好精致、（2020-9-8）、（点赞：19 观看：1188）
195. 冯川老、、（2021-4-22）、（点赞：72 评论:5 喜欢：2 观看：8036）
196. 面向大海、、（2019-6-29）、（点赞：10 观看：354）
197. 梓峰、、、、（2019-10-27）、（点赞：5 观看：258）
198. 蛙蛙旅行、（点赞：18 喜欢：2 观看：986）
199. 超级v奶爸、、（2021-3-18）、（点赞：10 喜欢：1 观看：1170）
200. 一个老兵、、（2022-8-18）、（点赞：4 观看：203）
201. 遇见美景、、（2020-6-29）、（点赞：47 评论：13 喜欢：4 观看：877）
202. 多年以后、（2021-12-15）、（点赞：3 观看：271）
203. 老当益壮、、（2021-5-25）、（点赞：6 喜欢：1 观看：445）
204. 多忘、、（2021-7-26）、（点赞：19 评论：2 观看：1323）
205. JJ77（2022-9-14）（点赞：4 观看：312）
206. Ca、、、y、（2021-5-21）、（点赞：20 喜欢：1 观看：1514）
207. 我的世外桃源、（2022-5-2）、（点赞：4 观看：395）
208. 菜菜的、、（2021-5-16）（点赞：274 评论：31 喜欢：23 观看：1.2万）
209. 达文西、、（点赞：4 观看：186）
210. M21、、、64、（2022-8-15）、（点赞：4 观看：472）
211. 眼镜的旅行、、（2022-9-19）、（点赞：3 观看：704）
212. 我也爱边牧|（2020-7-26）、（点赞：5 观看;1413）
213. Jinyu、、11（2022-8-16）、（点赞：9 观看：1271）
214. 爱旅行的巴扎黑、（2022-6-23）、（点赞：79 评论：9 喜欢：15 观看：7642）

**凤凰山革命旧址**

1. jinyu9711、延安凤凰山革命旧址、（2022-8-15一样）、（点赞：6 观看：1052）
2. 谁说我不是善男信女、（2022-1-3、2021-11-20）、（点赞：6 观看：246）
3. 谁说我不是善男信女、（2022-1-11、2021-11-20）、（点赞：4 观看：268）
4. 谁说我不是善男信女、（2022-1-14、2021-11-20）、（点赞：5 观看：189）
5. 散步的猫胖胖、（2021-7-1一样）、（点赞：12 喜欢：1 观看：1159）
6. 谁说我不是善男信女、（点赞：4 ）
7. 谢、英雄、（2022-1-20一样）、（点赞：14 喜欢：1 观看：）
8. 大雅、、旧事、（2022-5-29一样）、（点赞：5 观看）
9. 谁说我不是善男信女、（（2022-1-15、2021-11-20）（点赞：9 观看）
10. 谁说我不是善男信女、（（2022-1-5、2021-11-20）（点赞：5 观看）
11. 谁说我不是善男信女、（点赞：8观看：238）
12. 说我不是善男信女、（2022-1-4、2021-11-20）、（点赞5 观看：206）
13. 上面、（2022-1-4、2021-11-20）、（点赞：5 喜欢：1 观看：232）
14. 上面、（2022-1-4、2021-11-20）、（点赞：7平凉：1 喜欢：1 观看：230）
15. 芳芳外婆、（2021-1-11一样）、（点赞：4、观看：273）
16. Gzw001、（2020-1-21、2020-1-8）、（点赞：25 平凉：3 喜欢：1 观看：3465）
17. 说我不是善男信女、（2022-1-8、2021-11-20）、（点赞8 观看：275）
18. 说我不是善男信女、、（点赞6 观看：231）
19. 说我不是善男信女、（2022-1-13、2021-11-20）、（点赞4 观看：211）
20. 说我不是善男信女、（2022-1-7、2021-11-20）、（点赞7 平凉：1 观看：267）
21. 说我不是善男信女、（2022-1-9、2021-11-20）、（点赞8 观看：259）
22. 说我不是善男信女、（2022-1-5、2021-11-20）、（点赞6评论：1 观看：217）
23. 说我不是善男信女、（2022-1-12、2021-11-20）、（点赞3 观看：209）
24. 说我不是善男信女、（2022-1-7、2021-11-20）、（点赞4 观看：234）
25. 芳芳外婆、（2021-1-12一样）、（点赞：4、观看：335）
26. 说我不是善男信女、（2022-1-20、2021-11-20）、（点赞7评论：1喜欢：1 观看：240）
27. 说我不是善男信女、（2022-1-15、2021-11-28）、（点赞5 观看：300）
28. 说我不是善男信女、（2022-1-6、2021-11-20）、（点赞3 观看：221）
29. 说我不是善男信女、（2022-1-12、2021-11-20）、（点赞6 观看：254）
30. 说我不是善男信女、（2022-1-9、2021-11-20）、（点赞6喜欢：1 观看：244）
31. 说我不是善男信女、（2022-1-8、2021-11-20）、（点赞10 评论：1喜欢：1 观看：290）
32. 说我不是善男信女、（2022-1-10、2021-11-20）、（点赞6 喜欢：1 观看：254）
33. 说我不是善男信女、（点赞6 观看：195）
34. 说我不是善男信女、（2022-1-11、2021-11-20）、（点赞3观看：199）
35. 说我不是善男信女、（点赞3 观看：207）
36. 说我不是善男信女、（2022-1-7、2021-11-20）、（点赞4 观看：234）
37. 说我不是善男信女、（2022-1-6、2021-11-20）、（点赞2 观看：235）
38. 说我不是善男信女、、（点赞3 观看：191）
39. 说我不是善男信女、、（点赞6 观看：258）
40. 说我不是善男信女、（点赞6 观看：185）
41. 说我不是善男信女、（点赞5观看：266）
42. 说我不是善男信女、、（点赞4 观看：190）
43. 说我不是善男信女、（点赞2 观看：223）
44. 说我不是善男信女、、（点赞3 观看：233）
45. 说我不是善男信女、（点赞5喜欢：1 观看：216）
46. 、、天下、（2019-6-23、2019-5-21）（点赞：35 评论：3 喜欢：3 观看：4319）
47. 说我不是善男信女、（点赞2 观看：207）
48. 说我不是善男信女、（点赞6观看：200）
49. 说我不是善男信女、（点赞4 观看：208）
50. 说我不是善男信女、（点赞4 观看：235）
51. 说我不是善男信女、（点赞6 观看：197）
52. 说我不是善男信女、（点赞2 观看：189）
53. 说我不是善男信女、（点赞4 观看：234）
54. 说我不是善男信女、（点赞6 观看：234）
55. 说我不是善男信女、（点赞6 观看：224）
56. 说我不是善男信女、（点赞8 喜欢：1 观看：280）
57. 说我不是善男信女、（点赞6 观看：279）
58. LMT、、0、2、（2021-9-27、2021-8-31）、（点赞：3 观看：1604）
59. 说我不是善男信女、（点赞2 观看：190）
60. 说我不是善男信女、（点赞6 观看：215）
61. 风、痕、（（2019-10-24、2019-10-19）、（点赞：23 评论：1 观看：2003）
62. 芳芳外婆、（2022-2-8一样）、（点赞4 观看：244）
63. 好精致、（2020-9-16、2020-9-9）、（点赞：16 观看：2161）

**“四八”烈士陵园**

1. 喵陛下、（2019-5-3一样）、（点赞：14 评论：1 观看：5025）

**洛川县洛川会议纪念馆**

1. M37...145、（点赞：17 观看：4284）
2. 当地向导杜延岗、洛川会议旧址、（2021-3-15、2021-3-14）、（点赞：6 观看：468）
3. Q天涯、洛川会议旧址、（2019-10-4一样）、（点赞：12 观看：720）
4. Lmc2009、洛川会议旧址、（2019-11-7、2019-11-6）、（点赞：71 评论：1 喜欢：5 观看：6584）
5. M23...575、洛川会议旧址、（2019-10-7、2019-10-6）、（点赞：58 评论：5 喜欢：3 观看：6280）
6. 陕西微旅游、洛川会议旧址、（2020-1-10一样）、（点赞：23 评论：1 喜欢：1 观看：3302）
7. 牛牛魔王88、洛川会议旧址、（2020-11-20一样）、（点赞：5 观看：517）
8. 云中有我月、洛川会议旧址、（2019-8-7、2019-8-3）、（点赞：14 评论：4 观看：1373）

**子长县瓦窑堡会议旧址**

1. 水晶罐头、瓦窑堡会议旧址、（202-7-27、2021-7-9）、（点赞：6 观看：567）
2. 蛙儿子、瓦窑堡会议旧址、（2019-9-14一样）、（点赞：8 观看：1635）

**宝塔山景区**

1. 爱旅行的巴扎黑、宝塔山**攻略**、（2022-6-22）、（点赞:55 评论：4 喜欢：8 观看：4882）
2. 吃喝玩乐的颓废生活、宝塔山、（2021-5-8、2021-5-3）、（点赞：26 评论：4 喜欢：1 观看：4692）
3. 爱吃的小鱼、**攻略**、（点赞：182 评论：2 喜欢：45 观看：6819）
4. 天马行空y、宝塔山风景区、（点赞：2 观看：181）
5. 星、、者、**攻略**、（点赞：110 评论：1 喜欢：21 观看：6849）
6. CC颜夕、攻略、（点赞：506 评论：5 喜欢：69、观看：1.4万）
7. 小荟带你看中国、攻略|（点赞：893、 评论：34 喜欢：235 观看：11万）
8. 眼镜的旅行、攻略、（点赞：2 观看：579）
9. 青圆爱旅行、攻略、（点赞：4 观看：274）
10. 吃喝玩乐的颓废生活、宝塔山、（2021-5-2、2021-5-1）、（点赞：70 评论：3 喜欢：1 观看：215）
11. 小陈爱旅游、攻略、（点赞：3 观看：336）
12. 甜得、（点赞：4 观看：203）
13. 旅游知识点、（点赞：10 喜欢：1 观看：408）
14. 我也爱边牧、（2021-6-7、2021-5-25）、（点赞：8 观看：489）
15. 秋实的城市日记、攻略、（点赞：33 评论：1 喜欢：1 观看：3808）
16. Linglizi、宝塔山风景区、（2020-9-1、2020-8-11）（点赞：18 评论：1 观看：2089）
17. 大雅宝旧事、（2020-5-28一样）（点赞：3 观看：711）
18. 再平凡、、（2021-7-30一样）（点赞：33 评论：3观看：2247）
19. 冯、勇、（2021-10-13一样）\（点赞3 观看：398）
20. Linglizi、宝塔山风景区、（点赞6 评论：3 观看：776）
21. 向导包车、攻略、（点赞：95 评论：10 喜欢：15 观看;8201）\
22. Lyn0919、（2020-9-16、2017-10-5）、（点赞：15 喜欢：1 观看：1621）
23. 长风vision、（2021-7-21一样）、（点赞：63 评论：2 喜欢：4 观看：5119）
24. 天行天下、宝塔山、（2022-9-11一样）、（点赞：9 观看：215）
25. 杨洪8825、（2020-8-22一样）、（点赞：12 观看：535）
26. 渺渺看世界、（点赞：80 评论：3 喜欢：4 观看：2863）
27. 喝剩的摩卡、（2021-11-16一样）、（点赞：13 评论：3 喜欢：3 观看:1391）
28. 龙志带你去旅行、（点赞：3 评论：2 观看：191 ）
29. 台军虎、（2019-8-20、2019-8-8）、（点赞：16 评论：2 观看：508）
30. dwj、（2021-2-22、2020-10-31）、（点赞：53 评论：7 喜欢：1观看：1565）
31. 博物馆的胖胖、（2021-8-15、2020-7-5）、（点赞：40 评论：1 喜欢：2 观看3604）
32. 天上的蓝精灵017、（2022-4-21一样）、（点赞：4 观看：203）
33. 种草机、（2022-8-16）、（点赞：3 喜欢：1 观看：873）
34. 爱旅游的拾柒、（2022-10-2 、2022-10-1）、（点赞：2 观看：631）
35. 小程爱旅游、（2022-10-23一样）、（点赞：3 观看：303）
36. 芳芳外婆、（2022-2-2一样）、（点赞：4 观看：333）
37. Cycat20、（2019-7-4、2018-8-28）、（点赞：18 评论：5 观看：2528）
38. 璞石右棋、（2021-10-30一样）、（点赞：15 评论：6 喜欢：3 观看：1118）
39. M24...864、（2020-6-15一样）、（点赞：5 观看：257）
40. 还在路上的人、（2020-11-5、2020-11-3）、（点赞：8 观看：732）
41. 华-888、（2019-1-11一样）、（评论：2 观看：229）
42. （陕北）、、、（点赞：13 观看：1950）
43. 拉风的小白兔、（2014-11-30一样）、（点赞：3 观看：202）
44. Myyohayoha、（2021-7-28一样）、（点赞：7 观看：565）
45. 宝塔山景区、（2018-1-14一样）、（点赞：1 观看：218）
46. M42..0968、（2020-12-2一样）、（点赞：50 喜欢：3 观看：3482）
47. 当地向导.张勇、攻略、（点赞：17 评论：1 喜欢：1 观看:4729）

**桥儿沟革命旧址**

1. 谁说我不是善男信女、（2022-1-18、2021-11-20）、（点赞：3 观看：198）
2. 谁说我不是善男信女、（点赞：2 观看：207）
3. 谁说我不是善男信女、（点赞：2 观看：176）
4. 谁说我不是善男信女、（点赞：2 防控、、观看：209）
5. 芳芳外婆、（2021-12-12一样）、（点赞：4 观看：234）
6. 谁说我不是善男信女、（2022-1-19、2021-11-20）、（点赞：2 ）
7. 谁说我不是善男信女（点赞：3）
8. 谁说我不是善男信女、（2022-1-19、2021-11-20）、（点赞：2 ）
9. 谁说我不是善男信女、（2022-1-18、2021-11-20）、（点赞：4 ）
10. 漫天飘雪、（2021-4-20、2021-4-12）、（点赞：7 喜欢：1 ）
11. 谁说我不是善男信女、（2011-1-18、2021-11-20）、（点赞：4）
12. Zp..g、（点赞：4
13. M26...42、（2019-7-22一样）、（点赞：14 评论：4 观看：1018）

**南泥湾革命旧址**

1. 孔梦杰、（2021-7-27一样）、（点赞11 观看）
2. LINGLIZI、（2020-8-19、2020-8-12）、（点赞：7）
3. Liukeit、（2021-7-7、2021-7-2）、（点赞：3））
4. Liukeit、（2021-7-8、2021-7-2）、（点赞：2）
5. 西安向导孙、娟、（2021-10-15、2021-10-14）、（点赞：8 ）
6. 一蓑烟雨75、（2020-11-19一样）、（点赞：6 喜欢：1 ）
7. Liukeit、（2021-7-6、2021-7-2）、（点赞：7）
8. 小蜗牛、（2021-8-3一样）、（点赞：12）
9. Liukeit、（2021-7-5、2021-7-2）、（点赞：4 观看：229）
10. Liukeit、（2021-7-22、2021-7-2）（点赞：9 观看：289）
11. 渝人yuren、（2020-4-21一样）、（点赞：15 评论：1 观看：1852）
12. Misliu621、（2019-11-10、2021-11-7）、（点赞：10 观看：491）
13. Liukeit、（2021-7-24、2021-7-2）、（点赞：5 观看：195）
14. 东方驴客、（2019-7-7、2017-4-30）、（点赞：16 评论：6 观看：822）
15. 风之痕123123、（2019-10-22、2019-10-18）、（点赞：30 评论：2 观看：6159）
16. 由我决定、（2019-7-11、2019-7-10）、（点赞：12 评论：3 观看：584）
17. 梦想旅店、（点赞：38 评论：3 喜欢：3 观看：2142）
18. 没有美丽、、风景、（点赞：10 观看：395）
19. 饺子、（22020-1-29一样）、（点赞：28 观看：6636）
20. 牧T风、（点赞：7 观看：247）
21. 非你不渴、（2020-6-12、2020-6-9）、（点赞：14 评论：2 观看：1205）
22. 金陵、、（2019-11-12一样）、（点赞：4 观看;3389）
23. LMT\\02、（点赞：2 观看：1051）

**中共中央西北局革命旧址**

1. 云锦（繁体）、攻略、（点赞：211 评论：3 喜欢：91 观看：1.5万）
2. 锦瑟、123、（2021-4-22一样）、（点赞：7 观看：2181）
3. 阿木阿土、（2021-6-12、2021-6-5）、（点赞：34 评论：5 喜欢：1 观看：3304）
4. 脚步、天涯、（2021-7-6、2020--3-30）、（点赞：12 评论：1 观看：538）

**陕甘宁边区政府旧址**

1. 开挂的猫咪：、、不要门票、（2021-7-9）、（点赞：12 观看：2749）
2. zhangan55 、红色、、、旧址、（2020-2-10、2019-9-22）、（点赞：28 评论：1 观看：2107）
3. 牛奶海、（2021-12-15、2021-9-22）、（点赞：2 观看：232）
4. M93..30、（2020-9-3一样）、（点赞：2 观看：572）

**吴起镇革命旧址**

1. 大宋长歌、（2019-3-23一样）、（评论：1 观看：819）
2. 皓月东辉、（2020-1-7、2017-8-21）、（点赞：11 观看：1017）
3. WHCH...7、（2019-8-15、2019-7-13）、（点赞：20 评论：6 观看：4464）
4. 没有美丽的爱情、（2020-11-17、2020-11-16）、（喜欢：1 观看：797）
5. M37....7、（2019-12-8一样）、（点赞：14 评论：2 观看：816）
6. 阿平0001、（2019-8-20、2019-8-17）、（点赞：17 评论：4 喜欢：1 观看：1820）

**中国人民抗日军政大学纪念馆**

1. 芳芳外婆、（2021-11-21一样）、（点赞：4 观看：322）
2. 从不犹豫、（点赞：5观看：882）
3. 面向大海、（2021-7-27、2019-6-28）、（点赞：11 观看：620）
4. 芳芳外婆、（2021-11-3一样）、（点赞：4 观看：277）
5. 一头可爱的小毛驴、（2021-9-25、2021-7-11）、（点赞：8 喜欢：1 观看：381）
6. 我就说、、好、（2022-1-15、2021-11-20）、（点赞：4 观看：232）
7. 我就说、、好（2022-1-15、2021-11-20）、（点赞：4 观看：307）

**清凉山革命旧址**

1. 吃喝玩乐的颓废生活、清凉山（2021-5-8、2021-5-3）、（点赞：26 评论：4 喜欢：1 观看：4688）
2. 吃喝玩乐的颓废生活、（2021-5-5、2021-5-3）、（点赞：7 观看：1267）
3. 我就说山野村夫、清凉山上的万佛洞、（2021-12-27、2021-11-21）、（点赞：4 观看：638）
4. 吃喝玩乐的颓废生活、新闻纪念馆、（2021-5-5、2021-5-3）、（点赞：13 评论：1 观看：505）
5. 苏、、I、（2022-7-19、2022-7-27）、（点赞：10 喜欢：1观看：1073）
6. 还在路上、（2021-12-19一样）、（点赞：3 观看:203）
7. 、标、（2021-6-6、2021-5-27）、（点赞：30 喜欢：4 观看：3434）
8. 芳芳外婆、（2021-12-10一样）、（点赞：3 观看：282）
9. WH、、2、（2020-12-30一样）、（点赞：275 评论：48 喜欢：5 观看：8088）
10. 一人、、Y\、（点赞：6 评论：3 观看：908）
11. 东来、、（2020-5-23、2013-8-27）、（点赞：9 喜欢：1 观看：2046）

**咸阳市旬邑县马栏革命旧址**

1、清风若溪、马栏革命旧居、（2021-5-11、2021-5-1）（点赞：62 评论：12 喜欢：2 观看：2357）

2、悍生无解、（2020-1-15）、（点赞：244 评论：34 喜欢：41、观看：1.1万）

3、祥云88、（2021-7-13、2021-7-1）、（点赞：3 观看：303）

4、娴雅小溪、（2021-5-4、2021-5-1）、（点赞：19 评论：1 观看：686）

5、口是心非、（2020-6-27一样）、（点赞：13 观看：883）

6、幽人、（2022-3-6一样）、（点赞：4 观看：493）

7、口是心非、（2020-7-2、2020-6-27）、（点赞：12 评论：1 观看：732）

8、自在远方1、（2022-3-15一样）、（点赞：4 评论：2 观看：512）

9、一个老兵、、（2022-4-3一样）、（点赞：3 评论：433）

10、、、航、（2019-11-1、2019-9-24）、（点赞：评论：1 观看：1023）

11、祥云8、（2021-8-22 、2021-7-1）、（点赞：10 观看：400

**铜川市陕甘边照金革命根据地旧址**

1、开挂的猫咪、陕、、票、（2021-7-9一样）、（点赞：12 观看：2766）

2、你在焦点在、照金纪念馆、（2021-11-24一样）（点赞：7 观看：2115）

3、熊出没的地方、红色——照金、（2022-2-25一样）、（点赞：6 观看：1118）

4、乐游陕西、、、、拍、（2022-5-13一样）、（点赞：117 评论：2 喜欢：5 观看：6662）

5、涅槃虚静、陕西照金、（2021-7-5、2011-10-28）、（点赞：17 评论：3 观看：3548）

6、方圆四百里、（2020-9-10、2020-9-1）、（点赞：12 评论：1 观看：1546）

7、流浪者旅拍、陕西、、地、（2021-1-29、2018-2-18）、（点赞：18 喜欢：1 观看：3847）

8、篱上风、红色、游、（2021-5-13一样）、（点赞：13 观看：1284）

9、化工广哥、照金、、基地、（2021-6-20、2021-6-19）、（点赞：20 评论：3 喜欢：2 观看：2634）

10、M21..563\红色照金、（2019-7-3一样）、（点赞：22 评论：2 观看：6494）

11、Pma-zyh、（2019-9-13、2019-6-29）、（点赞：22 观看：5938）

12、娴雅小溪、（2021-8-24、2021-8-21）、（点赞：7 观看：512）

13、s 烟、（2021-8-8、2021-8-4）、（点赞：10观看：416）

14、蓝曦蓝曦、（2021-6-6一样）、（点赞：3 观看：321）

15、口是心非、（2021-7-21、2021-7-9）、（点赞：8 观看：253）

16、春暖花开、（2019-5-3一样）、（点赞：1 观看：638）

17、清风若曦、（2019-6-16、2019-6-15）、（点赞：58 评论：8 喜欢：1 观看：3493）

18、张新卫、（2021-7-24、2021-6-10）、（点赞：8 观看：298）

19、无欲无求、（2021-10-6、2021-10-4）、（点赞：7 评论：3 观看：589）

20、口是心非、（2021-7-22、2021-7-9）、（点赞：7 观看：241）

21、M24、、、32、（2019-8-20、2019-8-3）、（点赞：13 评论：3 观看：870）

22、独自在旅行、（2020-9-10、2020-6-26）、（点赞：11 喜欢：1 观看：550）

23、M21、、4、（2019-7-3一样）、（点赞：22 评论：2 观看：6496）

24、长木、、（点赞：39 评论：3观看1812）

25、萌、夏、（2019-9-24、2019-9-23）、（点赞：14 观看1008）

26\ICE\\、、5、（2019-8-20、2019-8-17）、（点赞：11 评论：3观看694 ）

1. 关、、n、（2020-1-5、2016-10-9）、（点赞：23 评论：2观看797）
2. JU8、（2019-10-30一样）、（点赞：21 喜欢：1 观看：1102）
3. M24、、7、（2019-12-2、2019-11-21）、（点赞：20 评论：2观看750）
4. 口是心非、（2019-7-7一样）、（点赞：16 评论：2 喜欢：1观看694）
5. 警、、主、（2019-8-3、2019-8-2）、（点赞：18 评论:4 观看1089）
6. 琉璃、拍、（2020-4-10、2020-4-4）、（点赞：178 评论：6 喜欢：9 观看9.4万）
7. 小可、（（2017-6-15一样））、（点赞：10 观看：1025）

米**脂县杨家沟革命旧址**

1. 陕北土疙瘩、（点赞：6 观看：866）
2. 紫色雪狐、杨家沟、、（2022-9-14一样）、（点赞：4 观看：247）
3. 320.....921、（2019-12-8、2019-11-29）、（点赞：16 观看：776）
4. 风吟天下v、（2021-7-26、2021-7-4）、（点赞：223 评论：3 喜欢：2 观看：5953）
5. 超喜欢油爆虾、杨家沟、、（2021-5-3一样）、（点赞：7 观看：740）
6. 好景致、（点赞：19 评论：3 观看：3017）
7. 好景致、（点赞：25 评论：2观看：2126）
8. 好景致、周恩来旧居、（点赞：11 观看：1424）
9. 娴雅小溪、杨家沟革命纪念馆、（2020-2-22、2016-7-24）、（点赞：14 观看：941）
10. WH1962、杨家沟革命纪念馆、（（2020-12-21一样）、（点赞：227 评论：39 喜欢：8 观看：1万）
11. M90...30.、杨家沟革命旧址、（2020-9-4一样）、（点赞：9 观看：870）
12. M27\、、1491、（2019-9-23一样）、（点赞：49 评论：7 喜欢：4 观看：5890）
13. 清风若曦、（2020-2-19、2016-7-24）、（点赞：27 观看：979 ）
14. 故圆、、（2020-9-29、2019-10-5）、（点赞：29 喜欢：2观看：7111）

**佳县神泉堡革命纪念馆,**

1. 咸阳天圆地方、（2020-3-25一样）、（点赞：10 观看：1101）
2. WCCH、、、20、（2020-7-20、2020-7-19）、（点赞：7 观看：1021）
3. 清风若曦、（2020-2-20、2016-7-25）、（点赞：24 评论：2 观看：953）

**绥德县革命历史纪念馆**

1、136、、、（2021-10-24、2021-9-25）、（点赞：13 喜欢：1 观看：1784）

1. 京、、3、（2020-5-12、2018-5-17）、（点赞：24 评论：1 观看：1605）
2. 在观景的路上、（2021-10-26、2021-10-10）、（点赞：59 评论：9 喜欢：3 观看：2504）

**眉县扶眉战役纪念馆**

1. 清风若、、(2020-6-23、2020-6-14）、（点赞：26 ）
2. 风\\hwj、（2019-11-6一样）、（点赞:23 喜欢：2 ）
3. 娴雅、、（2020-6-24、2020-6-14）、（点赞：24 评论：2 ）
4. 小西北狼、（2020-8-8、2020-6-23）、（点赞：8 ）
5. 1830、、、（2020-6-27、2020-6-20）、（点赞：17）
6. 悍、解、（2021-6-13、2021-6-11）、（点赞：116 评论;10 喜欢:15观看 :6444）

**汉中市洋县华阳红二十五军司令部旧址**

1. 金子小哥、（2019-8-10一样）、（点赞：27 评论：5 喜欢：3 观看：6146）
2. 金秋旅游笔记、（2022-10-20一样）、（点赞：4 观看：223）
3. 追风的耿、（2022-1-7一样）、（点赞：245 评论：20 喜欢：55 观看：1.6万）
4. 生活反馈、（2022-6-23一样）、（点赞：27 喜欢：4 观看：3357）

**西乡县红二十九军军部旧址及红四方面军总后医院旧址**

1. 承诺太假、、（点赞：7 喜欢：2 观看：2920）
2. 逐e、（2022-4-16一样）、（点赞：9 观看：1824）
3. 春天2月、、（2021-6-7、2021-6-2）、（点赞：8 观看：345）
4. 春天2月、、（2021-6-2、2021-6-2）、（点赞：9 观看：421）

**咸阳市泾阳县安吴青训班革命旧址**

1. 悍生无解、（2019-12-30、2019-6-28）、（点赞：159 评论：13喜欢：31 观看：6531）
2. QD杨、（2020-5-27一样）、（点赞：8 喜欢：1 观看：1157）
3. 凌姐看世界、（2019-11-2、2019-11-1）、（点赞：87 欧伦：6 喜欢：9 观看：6261
4. **黄陵县陕甘边小石崖革命旧址**

1、独自旅行2020、（2020-8-18一样）、（点赞：11 观看：4153）

1. **靖边县小河会议旧址**
2. 铁蛋他大爷、（2020-10-21、2020-10-4）、（点赞：14 观看：1084）
3. 6@浏若清风、（2019-4-22、2019-4-19）、（点赞：7 喜欢：1 观看：1194）
4. 云风1972、（2019-11-12、2019-10-27）、（点赞：22 评论：2 观看：4171）
5. Xoomei、（2019-8-7一样）、（点赞：11 观看：933）

**富平县青少年教育基地**

1. 下一站8541、（2022-4-5一样）、（点赞：3 观看：417）
2. 逸游长安旅行、（2021-8-23一样）、（点赞：53 评论：2 喜欢：8 观看：3153）
