# Supplementary material for: The network characteristics of classic red tourist attractions in Shaanxi province, China
Source: PLoS One. 2024 Mar 29;19(3):e0299286. doi: 10.1371/journal.pone.0299286 (PMC10980247; doi:10.1371/journal.pone.0299286)
Supplement: Attachment — Submitted filename: Response to Reviewers.docx [file pone.0299286.s010.docx]

**Modification instructions**

**Dear editors and reviewers of PLOS ONE：**

Hello!Thank you very much for your valuable suggestions for the paper! Your rigorous academic attitude and scientific research spirit have greatly inspired and benefited me. In the process of revising the paper, I have gained a profound understanding of the pertinence and guidance of the suggestions provided. The reviewer's comments are of great significance for further improvement and enhancement of the paper. In response to your comments and suggestions, the following comprehensive modifications have been made to the paper:

**Reviewer #1**：

(1) Scientific issues still need to be concise, such as focusing on coupling network attention and tourism flow network structure, so as to provide theoretical support for evaluating actual destination activities. Is there any probability to extend the conclusions and suggestions to other red tourism places in China.

**Revision note**: Firstly, I would like to express our gratitude to the expert's suggestions. In response to the aforementioned recommendations, this study has made revisions to the "suggestions" section.

**The revised content is as follows**(**For details, please refer to paper P26-P29**)：

In summary, the network attention towards the classic red tourism scenic spots in Shaanxi province is relatively low, and there are significant differences in network attention between different levels and types of attractions (Table 2). Based on this, this study proposes the following suggestions from macro and micro perspectives. From a macro perspective, on one hand, it is necessary to continue investing in and developing high-level attractions, and continuously play the key role of red heritage sites. On the other hand, attention should be given to the development of medium and small-sized attractions, with increased promotion to enhance their visibility and influence. From a micro perspective, suitable development strategies should be formulated based on tourism flow attributes and characteristics such as node weighted degree and comprehensive ranking value, focusing on four types of attractions: high-high, high-low, low-high, and low-low. The specific strategies are as follows:

(1)High-high type: Leveraging synergies to create tourism growth poles. Such tourist attractions have strong market influence and future development should be further enhanced while leveraging their synergistic effects. On the one hand, based on the advantage of tourist resources, offline tourism products should continue to be innovated while combining online advantages to expand market scale. On the other hand, the driving role of such tourist attractions should be leveraged to create characteristic and high-quality tourism routes, such as those based on revolutionary sites like the Zaoyuan Revolutionary Site and Yan’an Revolutionary Memorial Hall, combined with red tourist attractions (Fumei Battle Memorial Hall, Malan Revolutionary Site) and non-red tourist attractions (Yellow Emperor’s Mausoleum, Huangtu National Geopark, Zhongshan Grottoes).(2)High-low type:Optimizing product supply and improving service quality. This type of scenic spot already has a considerable number of potential tourists, and in the future, it should improve the basic tourism infrastructure and strengthen the standardization of tourism services. By combining the online influence advantages of historical sites such as Wangjiaping Revolutionary Site and Nanniwan Revolutionary Site, interactive and experiential activities such as red knowledge competition and agricultural experience could be organized based on the unique characteristics of these sites. This would further enhance the attractiveness of tourism and continue to improve the reputation and visibility of the scenic area.(3)Low-high type: Innovative marketing and promotional methods to enhance tourism attractiveness. This type of tourist attraction has relatively significant characteristics in the tourism flow, but it has low online attention. In the future, the marketing and promotional methods of this type of tourist attraction should be improved, and online tourism promotion should be strengthened to increase online attention. For example, through platforms such as Douyin, Xiaohongshu, and Weibo, adopting O2O integrated marketing and live streaming, innovative marketing and promotional methods should be implemented for attractions such as the ‘Xi’an Incident’ Memorial Hall and Baotashan to enhance the online influence of the attractions and cultivate potential tourists.(4)Low-low type: To enhance the online visibility of tourist attractions and improve the overall level of tourism products and services. The online visibility and development level of such attractions are relatively low, and it is necessary to comprehensively and multidimensionally enhance the development level of these attractions in the future. This can be achieved by increasing traditional media marketing such as television, radio, newspapers, magazines, while actively engaging in new media marketing. Additionally, it is important to further improve the level of attraction services, enhance reception capacity, promote the integration of culture and tourism, and expand the scope of revolutionary education, red-themed festivals, educational tourism, and other content, in order to create diversified tourism products.

Given the era value and practical demands of the development of red tourism, this study takes a perspective that combines ‘virtual’ and ‘reality’ and selects domestically representative and typical red tourism classic scenic spots with relatively abundant red tourism resources as the research objects. It attempts to explore the mutual relationship between the network attention of the research area and the structure of tourism flows from a spatial perspective. Certainly, due to the constraints of data availability and completeness, the author only obtained network data from six platforms. There are still many aspects of this study that are worth further discussion, such as expanding the selection of data sources, conducting a more systematic and in-depth exploration of the network attention and formation mechanism of tourism flow displacement in the research area. This will be the focus of subsequent research.

(2) There was problem of data resource overlaps for the data collection section. Both tourist flow and network attention data sources have Mafengwo and Qunar, therefore the reliability analysis and logical analysis of the data source should be supplemented. Please revise or give some explanations.

**Revision note**: Firstly, I would like to express my gratitude to the experts and teachers for their valuable suggestions. In response to the aforementioned recommendations, this study provides the following detailed explanations.

**The revised content is as follows(For details, please refer to paper P9 and P11)**：

Based on considering the objectivity, validity, and availability of the data, this study selected five social platforms, including Ctrip, WeChat, Baidu, 360, and Mafengwo, as the indicators for network attention (P9). Mafengwo and Qunar platforms were chosen as indicators for tourism flow data. Although there is some overlap between network attention and tourism flow data platforms, the selected indicators are different. Specifically, the indicators for network attention include the number of netizens’ comments, likes, comments, and views on Mafengwo platform, as well as the number of views on travelogues (Table 2). On the other hand, the indicators for tourism flow data are the travelogues (texts describing tourists’ movements between attractions) on both Mafengwo and Qunar platforms (P11). Therefore, the data from these two sources are not overlapping.

(3)I suggest you refine data screening. You selected travel notes published by tourism websites as data sources, which is innovative. Big data have great advantages in recording, but data quality need to be ensured.

**Revision note**: Firstly, I would like to express my gratitude to the expert for providing valuable feedback. In response to the aforementioned suggestions, this study has further refined and focused on the issues, and provided a detailed explanation of the data processing methods used in this research.

**The revised content and explanation are as follows(For details, please refer to paper P9)**：

Based on the references to previous studies and considering the comprehensiveness, accuracy, and availability of data, this paper selected five social media platforms, namely Ctrip, WeChat, Baidu, 360, and Mafengwo, as the sources for network attention data. The selection was made based on the evaluation of the abundance and completeness of data from each platform.The specific steps are as follows: Firstly, data collection is conducted using scope retrieval method (Table 1). This involves utilizing the Octopus collection tool to gather data from various platforms such as Mafengwo, Ctrip, Baidu, and 360 (The data collection period extends until January 1, 2023.). Data from WeChat platform is acquired through manual collection method (Data samples can be found in Table 2.). Next, the collected data is processed according to the indices of online reviews, online travelogues, social media, and search engines to obtain data on network attention (Table 2). Additionally, tourism flow data is sourced from Mafengwo and Qunar platforms (The data collection period extends until January 1, 2023.). In this study, travelogue texts from tourists are used as data, and a total of 1413 data points are acquired through the Octopus collection tool. Each of the 1413 travelogues is read individually, and the travel route data in each travelogue is counted. Eventually, 146 valid travel routes are obtained.To ensure the integrity of the travel flow network structure, non-red attractions are retained in the travel routes. The travel route ‘Nanniwan→Wangjiaping→Zaoyuan’ is split into ‘Nanniwan→Wangjiaping’ and ‘Wangjiaping→Zaoyuan’, resulting in 468 valid O-D data.

I have the following minor problems regarding to the expressions or figure and table formats:(1) In abstract and discussion section, data processing is more noteworthy than software, such as social network theory.

**Revision note**: First of all, I would like to express my gratitude to the expert for providing valuable insights. In response to the aforementioned suggestions, this study has made revisions to the "abstract" and “ discussion”section.

**The revised content is as follows(For details, please refer to paper P1 and P25)**：

This study, based on the perspective of combining ‘virtual’ and ‘reality’, takes 13 classic red tourism scenic areas in Shaanxi province as examples. It constructs a multi-source data network attention evaluation index and adopts social network analysis method to explore the network attention and tourism flow of the study case, and further investigates the relationship between the two. (P1)

This study employs social network analysis theory and multiple data sources such as network attention evaluation indicators to examine the relationship between the network attention and tourism flow characteristics of the classic red tourism scenic areas in Shaanxi province. The results reveal that:(P25)

(2) The database establishment should not be combined with red tourist attractions’ spatial layout in 3.1. They were two different things.

**Revision note**: First and foremost, I would like to express my gratitude to the expert for providing valuable insights. In response to the aforementioned suggestions, this study has removed Figure 2 and transformed the relevant data into a table. The specific modifications are as follows.

**The revised content is as follows(For details, please refer to paper P15):**

**Table 3. Network attention of scenic spots**

| **Network attention** | **Number of scenic spots** | **Percentage (%)** | **Representative scenic spots** |
| --- | --- | --- | --- |
| **0.000000-0.100000** | 2 | 5 | Wuqi Town Revolutionary Site ,Wei Hua Uprising Memorial Hall |
| **0.100001-0.200000** | 12 | 30 | The Memorial Hall of the Anti-Japanese Military and Political University of the Chinese People , the former site of the Shaanxi-Gansu-Ningxia Border Region’s Revolutionary Base in Zhaoguan |
| **0.200001-0.300000** | 5 | 12.5 | Niutiling Battle Site ,Qingliangshan Revolutionary Site |
| **0.300001-0.400000** | 9 | 22.5 | Fengghuangshan Revolutionary Site, Qianpoling Battlefield Remains |
| **0.400001-0.500000** | 4 | 10 | ‘Xi’an Incident’ Memorial Hall, Malan Revolutionary Site |
| **0.500001-0.600000** | 5 | 12.5 | Xiahe Conference Site, Zaoyuan Revolutionary Site |
| **0.600001-1.000000** | 3 | 7.5 | Nanjiniwan Revolutionary Site, Yan’an Revolutionary Memorial Hall |

(3)Figure1lacks elevation legend, and coordinates should be added.

**Revision note**: Firstly, I would like to express my gratitude to the expert for providing their valuable insights. In response to the aforementioned suggestions, this study has made revisions to "Figure 1".

**The modified version is as follows(For details, please refer to paper P7)：**

**Fig 1. Distribution map of classic red tourist attractions in Shaanxi province**

**Note: The map is drawn based on the standard map of the National Natural Resources Department of China (Surveying and mapping approval No. GS(2019)1822), and the base map remains unaltered.**

(4)Horizontal lines should be added to Table 1.

**Revision note**: Firstly, I would like to express my gratitude to the expert for providing their valuable insights. At the same time, modifications have been made to Table 1.

**The modified version is as follows(For details, please refer to paper P10):**

**Table 1. Examples of classic red tourist attractions in Shaanxi province**

| **Red tourism classic scenic areas** | **Number of attractions** | **Search example** |
| --- | --- | --- |
| **Xi’an red tourism series attractions** | 2 | Eighth Route Army Xi’an Office Memorial Hall, ‘Xi’an Incident’ Memorial Hall |
| **Yulin city red tourism series attractions** | 3 | Yangjiagou Revolutionary Site, Shenquanbao Revolutionary Memorial Hall, Suide County Revolutionary History Memorial Hall |
| **Fuping county red tourism series attractions** | 4 | Fuping County Youth Education Base,  the Memorial Site of the 120th Division of the Eighth Route Army’s Anti-Japanese War,  the Former Site of the Traffic Liaison Station of Weibei Revolutionary Base Area,  Kangzhuang Battle Martyrs Cemetery |
| **Yan’an Revolutionary**  **Memorial Site series attractions** | 17 | Yan’an Revolutionary Memorial Hall, Zaoyuan Revolutionary Site, Yangjialing Revolutionary Site, Wangjiaping Revolutionary Site, Fengghuangshan Revolutionary Site, Qingliangshan Revolutionary Site, “48” Martyrs Cemetery, Luochuan Conference Memorial Hall, Wayaobao Conference Site, Baotashan Scenic Area, Qiaoergou Revolutionary Site, Nanniwan Revolutionary Site, the Revolutionary Site of the Northwest Bureau of the Central Committee of the Communist Party of China, the Former site of the Shaanxi-Gansu-Ningxia Border Region Government, Baoan Revolutionary Site, Wuqi Town Revolutionary Site, the Memorial Hall of Chinese People’s Anti-Japanese Military and Political University |

(5) Network attention index selection. The weights of the four selected indexes with strong collinearity are close, so it is better to employ all.

**Revision note**:Firstly, I would like to express my gratitude to the expert for their valuable feedback. In response to the aforementioned suggestions, this study has further refined and focused on the issues, which will be elaborated in detail.

**The explanation are as follows（****For details, please refer to paper P11**)：

This study has utilized all four indicators of network attention and assigned weights to them.

(6) Figure 2 also had format problems. Table 3 could be deleted or be placed in supplement.

**Revision Note:** Firstly, I would like to express our gratitude to the expert for providing valuable feedback. In response to the suggestions, we have removed "Figure 2" and presented the data in table format. Table 3 has been deleted. (**For details, please refer to paper P15**)

(7)Maybe there are other better expressions for current Figure 4.

**Revision note**: Firstly, I would like to express my gratitude to the expert for their valuable suggestions. In response to the aforementioned recommendations, this study has re-drawn "Figure 4" with the following modifications.

**The revised content is as follows（For details, please refer to paper P22):**

**Fig 3. Diagram of** **network attention,** **weighting degree, and comprehensive ranking value (S3 Table)**

(8) Check the format of references.

**Revision note**:Firstly, I would like to express my gratitude to the experts and teachers for their valuable suggestions. In response to the aforementioned recommendations, this study has made modifications to the format of the "References" section. For further details, please refer to pages 29 to 32 of the paper.

**Reviewer #2：**

Dear Author:The manuscript entitled Research on the Network Attention and Its Characteristics of Tourism Flow Network Structure of Red Tourist Attraction in Shaanxi, China requires major revision.

(1)The things that need to be done to improve the quality of this scientific paper are: please complete the problem statement, clarify the research problem, and mention the research method in connection with statistical analysis in the abstract section.

**Revision note**：Firstly, I would like to express my gratitude to the expert for providing valuable suggestions. In response to the aforementioned recommendations, this study has further refined and focused on the issues, and made detailed revisions to the "abstract" section.

**The revised content is as follows(For details, please refer to paper P1)**:

Tourism flow is a significant tourism phenomenon and a hot topic of tourism geography research. This study, based on the perspective of combining ‘virtual’ and ‘reality’, takes 13 classic red tourism scenic areas in Shaanxi province as examples. It constructs a multi-source data network attention evaluation index and adopts social network analysis method to explore the network attention and tourism flow of the study case, and further investigates the relationship between the two.

（2）The problem formulation, research objectives, and research problems in the introduction need to be added.

**Revision note**：First, I would like to express my gratitude to the expert for providing valuable insights. In response to the aforementioned suggestions, this research has made thorough revisions to the "Introduction" section.

**The revised content is as follow(For details, please refer to paper P3)**:

The spatial pattern of tourism flows represents tourists’ movement and reflects the diversity of tourism resources, making it a focal point of research in tourism geography. The level of network attention reflects the overall perception of tourists towards a destination from a spatial perspective ^[2]^. It serves as a mapping of public attention and potential demand, and also has a predictive effect on actual tourist flows ^[3]^. Therefore, there exists a close relationship between network attention and tourism flows ^[4]^, providing a new perspective for studying tourism activities. In recent years, with the development of the digital economy, the ‘internet +’ model has played an important role in promoting rural revitalization and regional tourism development in the context of red tourism. It has provided new momentum for the upgrading and transformation of the tourism industry. Therefore, this study intends to select classic red tourism scenic areas in Shaanxi province as the case study area. Based on a combination of ‘virtual’ and ‘reality’ perspectives, it aims to explore the characteristics of network attention and the structure of tourism flows in red tourism. Through empirical research, it seeks to deepen the understanding of the mutual relationship between tourism flows and network attention, which has significant academic value for enhancing the theoretical understanding of their relationship.

(3)Make sure the font size and font type are the same for each paragraph.

**Revision note**：Firstly, I would like to express my gratitude to the expert for providing valuable suggestions. In response to the aforementioned recommendations, this study has made revisions to the manuscript in accordance with the requirements of the journal. For specific details, please refer to the revised content of the document.

(4)Theoretical analysis with the help of secondary data on research results needs to be added to research results, especially the analysis of spatial layout characteristics. Include numerical information in the conclusion.

**Revision note**：Firstly, we would like to express our gratitude to the expert for providing valuable suggestions. In response to the aforementioned recommendations, we have made detailed revisions.

**The revised content is as follow（For details, please refer to paper P17, P25 and P26)**

Overall, the case-based network structure exhibits a spatial characteristic of ‘one-level, multiple cores’ (Fig 2). The ‘one-level’ refers to the aggregation of core nodes in Yan’an City, represented by sites such as Zaoyuan Revolutionary Site and Yan’an Revolutionary Memorial Hall. These nodes are located at the core of the network and possess strong aggregation and diffusion capabilities. The ‘multiple cores’ indicate the formation of multiple important core nodes. In terms of regional distribution, apart from Yan’an City, Xi’an City has also formed important nodes represented by the ‘Xi’an Incident’ Memorial Hall and the Eighth Route Army Xi’an Office Memorial Hall. Additionally, in the network structure, red nodes often achieve their own development through collaboration with non-red nodes. Non-red nodes such as Hukou Waterfall, Yan’an 1938, and Drum Tower have close connections with other nodes and play important roles in the network structure.(P17)

(1)The spatial layout of ‘north dense and south sparse’ has been formed in the case study. Specifically, it is manifested by the highest total number of tourist attractions in northern Shaanxi (23) and their concentration in Yan’an city (17), while southern Shaanxi has the lowest total number of tourist attractions (6) and they are relatively dispersed in distribution.(2) The overall network attention of the case study is relatively low (with a mean of 0.327752) and there are differences in network attention among different scenic spots. In terms of regions, the scenic spots in Yan’an have higher network attention, while the network attention of scenic spots in other regions is relatively low. In terms of scenic spot types, scenic spots with higher levels of tourism and historical sites have higher network attention, while other types of scenic spots have lower network attention.(3) The overall tourism flow network structure of the case study exhibits spatial characteristics of ‘one level and multicore’ and a significant small-world phenomenon. The core nodes are mostly located in Yan’an City, and together with the core nodes in Xi’an City, they form a ‘multicore’ network structure. The nodes in the small-world network are closely connected, with a larger number of medium and small-sized nodes.(P25-P26)

(5) Include numerical information in the conclusion.

**Revision note**：Firstly, we would like to express our gratitude to the expert for providing valuable suggestions. In response to the aforementioned recommendations, we have made detailed revisions.

**The revised content is as follow（For details, please refer to paper P25 )**

(1)The spatial layout of ‘north dense and south sparse’ has been formed in the case study. Specifically, it is manifested by the highest total number of tourist attractions in northern Shaanxi (23) and their concentration in Yan’an city (17), while southern Shaanxi has the lowest total number of tourist attractions (6) and they are relatively dispersed in distribution.(2) The overall network attention of the case study is relatively low (with a mean of 0.327752) and there are differences in network attention among different scenic spots. In terms of regions, the scenic spots in Yan’an have higher network attention, while the network attention of scenic spots in other regions is relatively low. In terms of scenic spot types, scenic spots with higher levels of tourism and historical sites have higher network attention, while other types of scenic spots have lower network attention.

(6)Clarify the source of the photo data again.

**Revision note**：Firstly, I would like to express my gratitude to the expert and teacher for providing their valuable insights. In response to the aforementioned suggestions, this study has made the following revisions: "Figure 1" has been redrawn and the image source has been indicated.

**The modified content is as follows(For details, please refer to paper P7):**

**Fig 1. Distribution map of classic red tourist attractions in Shaanxi province**

**Note: The map is drawn based on the standard map of the National Natural Resources Department of China (Surveying and mapping approval No. GS(2019)1822), and the base map remains unaltered.**

(7)In the Methods chapter, it is necessary to mention the type of statistical analysis, clarify the population size and sample size, clarify the primary data collection techniques and data collection tools, and clarify the secondary data collection techniques. These are the suggestions that can be conveyed; hopefully it will be useful.

**Revision note**: First of all, I would like to express my gratitude to the expert for the suggestions provided. In response to the aforementioned recommendations, this research has made detailed explanations and modifications to the "research methods" section, as well as the data sample processing techniques.

**The revised content and explanation are as follows(For details, please refer to paper P9)**：

There are two main methods for evaluating network attention: one is based on Baidu Index, and the other is to construct a network attention evaluation index using multiple data sources. However, when the research area includes multiple tourism resources, it is difficult to obtain comprehensive and reliable data using Baidu Index as the indicator . As this study involves a large number of tourism resources, the reliability and accuracy of using a single data source are poor. Therefore, it is necessary to construct a network attention evaluation model to study the case area. Based on the references to previous studies and considering the comprehensiveness, accuracy, and availability of data, this paper selected five social media platforms, namely Ctrip, WeChat, Baidu, 360, and Mafengwo, as the sources for network attention data. The selection was made based on the evaluation of the abundance and completeness of data from each platform.The specific steps are as follows: Firstly, data collection is conducted using scope retrieval method (Table 1). This involves utilizing the Octopus collection tool to gather data from various platforms such as Mafengwo, Ctrip, Baidu, and 360(The data collection period extends until January 1, 2023.). Data from WeChat platform is acquired through manual collection method (Data samples can be found in Table 2.). Next, the collected data is processed according to the indices of online reviews, online travelogues, social media, and search engines to obtain data on network attention (Table 2). Additionally, tourism flow data is sourced from Mafengwo and Qunar platforms(The data collection period extends until January 1, 2023.). In this study, travelogue texts from tourists are used as data, and a total of 1413 data points are acquired through the Octopus collection tool. Each of the 1413 travelogues is read individually, and the travel route data in each travelogue is counted. Eventually, 146 valid travel routes are obtained. To ensure the integrity of the travel flow network structure, non-red attractions are retained in the travel routes. The travel route ‘Nanniwan→Wangjiaping→Zaoyuan’ is split into ‘Nanniwan→Wangjiaping’ and ‘Wangjiaping→Zaoyuan’, resulting in 468 valid O-D data.

**Reviewer #3：**

（1）Data Sources and Platform Selection:The paper would benefit from a more comprehensive approach to data collection, particularly with regard to network attention and tourism flow. It is important to diversify data sources and choose representative platforms. Consider including supplementary data from platforms like Weibo and railway 12306.

**Revision note**: Firstly, I would like to express my gratitude to the expert for providing valuable insights. In response to the aforementioned suggestions, this study offers the following modifications and explanations.

**The revised content and explanation are as follows(For details, please refer to paper P9 and P28)：**

Based on the consideration of comprehensiveness, accuracy, and availability of data ^[8]^, this study obtained data on network attention and tourist flow from six Chinese social platforms including Ctrip, WeChat, Baidu, 360, Mafengwo, and Qunar(P9). Initially, data from the Weibo platform was also collected, but the quantity and validity of the data were low, so it was excluded from the study. In addition, the data acquisition channels for the Railway 12306 platform were limited and difficult, so this platform's data was also excluded from the study based on the consideration of data availability. The authors would like to thank the reviewers for their valuable suggestions.

Certainly, due to the constraints of data availability and completeness, the author only obtained network data from six platforms. There are still many aspects of this study that are worth further discussion, such as expanding the selection of data sources, conducting a more systematic and in-depth exploration of the network attention and formation mechanism of tourism flow displacement in the research area. This will be the focus of subsequent research.（P28）

（2）Aesthetic Improvement of Figures: Figures 1 and 2 require aesthetic adjustments to enhance their visual appeal and effectiveness in conveying the intended information. Please revise them accordingly for better clarity and presentation.

**Revision note**: Firstly, I would like to express my gratitude to the expert for providing valuable feedback. In response to the aforementioned suggestions, this study has made the following modifications: the images have been redrawn and the source has been indicated.

**The revised content is as follow(For details, please refer to paper P7 and P15)：**

**Fig 1. Distribution map of classic red tourist attractions in Shaanxi province**

**Note: The map is drawn based on the standard map of the National Natural Resources Department of China (Surveying and mapping approval No. GS(2019)1822), and the base map remains unaltered.**

**Table 3. Network attention of scenic spots**

| **Network attention** | **Number of scenic spots** | **Percentage (%)** | **Representative scenic spots** |
| --- | --- | --- | --- |
| **0.000000-0.100000** | 2 | 5 | Wuqi Town Revolutionary Site ,Wei Hua Uprising Memorial Hall |
| **0.100001-0.200000** | 12 | 30 | The Memorial Hall of the Anti-Japanese Military and Political University of the Chinese People , the former site of the Shaanxi-Gansu-Ningxia Border Region’s Revolutionary Base in Zhaoguan |
| **0.200001-0.300000** | 5 | 12.5 | Niutiling Battle Site ,Qingliangshan Revolutionary Site |
| **0.300001-0.400000** | 9 | 22.5 | Fengghuangshan Revolutionary Site, Qianpoling Battlefield Remains |
| **0.400001-0.500000** | 4 | 10 | ‘Xi’an Incident’ Memorial Hall, Malan Revolutionary Site |
| **0.500001-0.600000** | 5 | 12.5 | Xiahe Conference Site, Zaoyuan Revolutionary Site |
| **0.600001-1.000000** | 3 | 7.5 | Nanjiniwan Revolutionary Site, Yan’an Revolutionary Memorial Hall |

（3）Analysis of Discrepancy between Network Attention and Tourism Flow:While the research method is appropriate, it is essential to delve deeper into analyzing the reasons for the observed differences between network attention and tourism flow. This addition will provide a more insightful and holistic view of the study.

**Revision note**:First of all, I would like to express my gratitude to the expert for providing valuable insights. In response to the aforementioned suggestions, this study presents the following modifications and explanations.

**The revised content and explanation are as follows(For details, please refer to paper P24)：**

The network attention reflects the level of interest that tourists have in red tourism resources in the virtual online world, while the tourism flow network reflects the movement of tourists in the real world. Research has shown that, on one hand, network attention can depict the evolution process of tourism flow and has a certain ‘precursor effect’, indicating a significant correlation between the ‘virtual’ and the ‘real’. On the other hand, network attention behavior occurs after the visit and has a ‘lag effect’, thus there is a correlation as well as differences between the two. In addition, from the perspective of research objects, the analysis of textual data reveals that tourists’ red tourism behavior mostly occurs during party building and study tours, which to some extent reflects the characteristic differences between tourism flow and network attention. Therefore, when formulating tourism strategies, tourism destinations should not only consider the construction of the scenic area’s online promotion platform, but also pay attention to the attributes and characteristics of their own tourism resources.

（4）Conclusion and Correlation:The conclusion rightly highlights that the correlation between network attention and tourism flow appears to be inconspicuous, suggesting that the influence of network attention on tourism flow might not be substantial. This observation is noteworthy and should be emphasized in the conclusion.

**Revision note**: Firstly, I would like to express my gratitude towards the expert's suggestions. In response to the aforementioned recommendations, this study has already made revisions and explanations to the "Conclusion" section.

**The revised content and explanation are as follows(For details, please refer to paper P26)**：

(4) There is a connection as well as differences between the network attention and tourist flow of case-based destinations. Based on these differences, attractions can be categorized into four types: high-high, high-low, low-high, and low-low. Each type has a different level of development, requiring differentiated measures to promote the development of various types of attractions.In terms of its causes, the network attention has certain characteristics of ‘precursor effect’ and ‘lag effect’, which are somewhat misaligned with the actual tourist flow. Additionally, the attributes of destination tourism resources in specific cases will also to some extent affect the fluctuations in online attention and tourist flow.

（5）Language and Grammar Corrections:The following word and grammar corrections should be made for improved readability and accuracy.

**Revision note:**Firstly, I would like to express my gratitude to the expert for providing valuable suggestions. In response to the aforementioned recommendations, this study has made modifications to the grammar of the paper. For further details, please refer to the main body of the paper.

Finally, I would like to express my gratitude to the reviewing experts and editing teachers for their instructive comments and constructive suggestions provided for this paper. I wish you all the best in your work and personal life. Meanwhile, I hope that PLOS ONE will continue to thrive and prosper.
