# Supplementary material for: The network characteristics of classic red tourist attractions in Shaanxi province, China
Source: PLoS One. 2024 Mar 29;19(3):e0299286. doi: 10.1371/journal.pone.0299286 (PMC10980247; doi:10.1371/journal.pone.0299286)
Supplement: Attachment — Submitted filename: Response to Reviewers.docx [file pone.0299286.s011.docx]

**Modification instructions**

Dear editors and reviewers of PLOS ONE:

Thank you for giving us the opportunity to submit a revised draft of the manuscript ‘Research on the network attention and its characteristics of tourism flow network structure of classic red tourism scenic areas in Shaanxi, China’ for publication in the Journal of PLOS ONE. We appreciate the time and effort that you and the reviewers dedicated to providing feedback on our manuscript and are grateful for the insightful comments on and valuable improvements to our paper. We have incorporated most of the suggestions made by the reviewers. Those changes are highlighted in the manuscript. Please see below, in blue, for a point-by-point response to the reviewers’ comments and concerns. All page numbers refer to the revised manuscript file with tracked changes.

Reviewer #4

**1.The title is of Chinese style and not so good. It is suggested to change the title to "The network characteristics of classic red tourist attractions in Shaanxi province, China".**

**Revision note**: Thank you for the reviewer's comments. In response to the suggestions provided, this study has made modifications to the title. For further details, please refer to the revised manuscript.

**2. In abstract, the description of virtual and reality is misleading and inaccurate.**

**Revision note:**Thank you for the comments provided by the reviewer. In response to the suggestions, this study has made revisions to the abstract. The modified content is as follows (for details, please refer to manuscript pages 1-2).

**Abstract**

This study takes the classic red tourism attractions in Shaanxi province, China as an example and constructs a multi-source data network attention evaluation index. Additionally, it employs social network theory to explore the network attention and tourist flow characteristics of the case study area.Research shows that: (1) Overall, the network attention to case-based destinations is relatively low, and there are significant differences in network attention among different attractions. Spatially, the distribution of network attention is uneven. This is manifested by higher network attention to attractions in Yan’an city and lower network attention to attractions in other regions.(2) There are differences in the network attention of different types of attractions. High-level attractions receive higher network attention, while low-level attractions receive lower network attention. Additionally, archaeological sites tend to receive higher network attention.(3) The network density of tourist flow is low, and the tourism connections between nodes are not closely linked. The linkage between core nodes and edge nodes in tourism is poor. Developed tourism routes only exist in core nodes.(4) Nodes such as Zaoyuan revolution site, Yangjialing revolution site, and Wangjiaping revolution site have a significant influence in the network structure. In addition, non-red nodes play an important role in the network structure, and red tourism is often combined with non-red tourism resources.(5) There is a correlation between network attention and tourist flow, as well as a ‘misplacement’ feature. Based on the characteristics of attractions, they can be divided into four types:bright-star attractions, cash-cow attractions, thin-dog attractions, and question attractions. Based on these conclusions, this study proposes targeted development suggestions for the four types of attractions based on the principle of ‘precise identification and classification strategies’.

**3. The concept of red tourism needs professional explanation and literature support. Why China attaches great importance to the development of red tourism? How is it different from other types of tourism?**

**Revision note:**Thank you for the comments provided by the reviewers. In response to the aforementioned suggestions, this study has made revisions to the content related to red tourism. The modifications made are as follows. (For further details, please refer to pages 2 to 3 of the paper.)

Red tourism is a specialized form of tourism in China that revolves around revolutionary memorial sites, landmarks, and other related elements. It focuses on the revolutionary history, achievements, and spirit ^[1]^. Unlike other types of tourism, red tourism is unique to China and serves as an important means to promote Chinese revolutionary culture. The Chinese government attaches great importance to the development of red tourism, resulting in a strong market demand and promising future prospects for red tourism in China. In recent years, red tourism has experienced rapid growth under the government’s promotion.On March 23, 2021, People’s Daily pointed out that from 2004 to 2019, China’s red tourism resources have been continuously expanding, and the red tourism market has become increasingly active. The ‘Report on the development of China’s red tourism (2022)’ states that in 2022, the cumulative number of tourists received in China’s red tourism reached 3.478 billion, with a comprehensive income of 929.5 billion yuan. Red tourism has gradually become an important driving force for China’s tourism development.

**4. In introduction, the first paragraph introduces the red tourism, and the second paragraph introduces the tourist flow, but there is no transition between the two paragraphs. It is suggested that the introduction part focus on the development of the red tourism scenic spots, and the tourism flow should be moved to the literature review part.**

**Revision note:**Thank you for the comments provided by the reviewer. In response to the aforementioned suggestions, this study has made revisions to the introduction section. The modified content is as follows. (Please refer to pages 2-4 of the paper for more details.)

Red tourism is a specialized form of tourism in China that revolves around revolutionary memorial sites, landmarks, and other related elements. It focuses on the revolutionary history, achievements, and spirit ^[1]^. Unlike other types of tourism, red tourism is unique to China and serves as an important means to promote Chinese revolutionary culture. The Chinese government attaches great importance to the development of red tourism, resulting in a strong market demand and promising future prospects for red tourism in China. In recent years, red tourism has experienced rapid growth under the government’s promotion.On March 23, 2021, People’s Daily pointed out that from 2004 to 2019, China’s red tourism resources have been continuously expanding, and the red tourism market has become increasingly active. The ‘Report on the development of China’s red tourism (2022)’ states that in 2022, the cumulative number of tourists received in China’s red tourism reached 3.478 billion, with a comprehensive income of 929.5 billion yuan. Red tourism has gradually become an important driving force for China’s tourism development.

Red tourism is an important component of China’s tourism, which has been extensively studied by scholars. Currently, both domestic and international research on red tourism focuses on its value, spatial characteristics, and influencing factors.

In terms of the value of red tourism, scholars believe that its development not only brings enormous economic benefits, but also serves as an important form of spreading advanced culture and conducting ideological education ^[2]^. In the context of the new era, red tourism carries the historical mission of disseminating red culture and enhancing national identity ^[3]^, which holds profound significance for China. Furthermore, red tourism possesses profound cultural connotations and contemporary values, serving as an important driving force for enhancing cultural self-confidence and meeting the cultural consumption needs of the people ^[4]^.In terms of the spatial characteristics of red tourism, scholars often use social network theory and spatial structure theory to study the spatial features of red tourism. For example, Cong Li et al. (2021) studied the network structure of red tourist flow in China based on network data ^[5]^. Subsequently, Hong Xiafang and Huang Lingguang (2022) analyzed the spatial distribution of revolutionary cultural relics in Jiangxi province, China ^[6]^. Building on the aforementioned studies, Zou Jianqin et al. (2021) conducted research on the influencing factors of red tourism ^[7]^. Scholars have pointed out that factors such as economic development, information technology, transportation conditions, and geographical distance are important factors influencing red tourism ^[8]^. Wang Qin’an et al. (2022) additionally emphasized that per capita disposable income, tourism revenue, and population size are also key influencing factors ^[9]^. Furthermore, climate conditions and holiday systems have a significant impact on the spatial distribution of red tourism ^[10]^.

Due to the fact that red resources and red culture are the foundation of red tourism, the Chinese government attaches great importance to the utilization of red resources and the inheritance of red culture. Promoting the integration and development of red culture and tourism is an important measure to promote the development of the Chinese tourism industry ^[11]^. The development of red resources and red culture not only helps to promote national spirit, but also enhances the cultural cultivation of the people. Scenic areas should increase the development strategies for red resources and red culture ^[12]^, so as to maximize their practical value.

**5. I am not sure why the classic red scenic spot in Shaanxi Province were selected instead of other provinces in China.**

**Revision note:**Thank you for the comments provided by the reviewers. In response to the suggestions, this study offers the following explanations: Firstly, Shaanxi province possesses abundant red tourism resources and exerts significant influence in China. Therefore, it was chosen as the case study location. Additionally, the influential nature of classic red tourism scenic spots makes them the most representative tourism resources among numerous red tourism destinations. They attract high public attention and serve as important drivers for promoting the high-quality development of red tourism in China. Moreover, they serve as significant platforms and carriers for telling red stories, inheriting red genes, conducting patriotic education, and promoting national spirit. Lastly, the data validity of the classic red tourism scenic spots in Shaanxi Province is satisfactory, thus making them suitable for analysis. Therefore, this study selected representative and typical classic red tourism scenic spots in Shaanxi province as the case study site.

**6. The manuscript mentioned that "In terms of network attention and tourism flow adaptation relationship, there are fewer studies on the integration and development of the two". At present, there are a lot of researches on the adoption of big data to analyze tourism flow, so it is suggested to remove the sentence.**

**Revision note:**Thank you for the reviewer's comments. In response to the aforementioned suggestions, this study has removed the mentioned paragraph. For more details, please refer to the main body of the paper.

**7. Why has the network structure of tourism flow become one of the main directions of tourism flow research, and what is the research purpose of the network structure?**

**Revision note:**Thank you for the reviewer's comments. In response to the suggestions provided, this study has made revisions to the tourism flow analysis. The modifications made are as follows (for further details, please refer to pages 5-6 of the paper).

The spatial structure of tourism refers to the spatial scale and degree of aggregation formed by the interaction of tourism objects ^[15]^. This spatial scale and degree of aggregation can reflect the spatial attributes and interrelationships of tourism activities, providing guidance for the spatial planning of tourist attractions.Based on this, scholars have conducted extensive research on the spatial characteristics of tourist flow. In terms of research methods, most studies use data from tourism digital footprints such as online texts and images, and analyze the spatial characteristics of tourist flow using spatial analysis methods and social network analysis methods. The scales of the research objects include attractions ^[16]^, cities ^[17]^, provinces ^[18]^, urban agglomerations ^[19]^, regions ^[20]^, and the whole country ^[21]^, among others.Based on this, scholars have further analyzed the influencing factors of tourist flow, pointing out that the spatial structure of tourist flow is closely related to factors such as tourism resource endowment, transportation level, and distance ^[21]^. The number of private car ownership, total social consumer goods retail sales, and internet broadband user access also have a strong impact on tourist flow ^[23]^. In addition, Yang Li et al. (2023) found that the total volume of telecommunications services, the number of tourism industry employees, the number of accommodation enterprises, the number of corporate legal entities, and the total government investment in the tourism industry are important factors affecting tourism information flow ^[24]^.In terms of the correlation of tourist flow, most scholars have studied the coupling relationship between tourist flow and transportation based on a coupling perspective ^[25-26]^. Some scholars have also focused on other correlations of tourist flow, such as the correlation between tourist flow and the tourism environment ^[27]^, and the degree of coupling between tourism and the economy ^[28]^. A few scholars have also studied the impact of haze ^[29]^, transportation ^[30]^, policies ^[31]^, language ^[32]^, natural disasters, and trade openness ^[33]^ on tourist flow.

**8. The literature review on the evolution of spatial structure of tourism flows and its influencing factors is too simple and lacks in-depth discussion.**

**Revision note:**Thank you for the comments provided by the reviewer. In response to the aforementioned suggestions, this study has made revisions to the tourism flow analysis. The modifications made are as follows (for further details, please refer to pages 4 to 6 of the paper).

Tourist flow connect the origin and destination and serve as the foundation for the development of the tourism industry ^[13]^. Its spatial pattern not only represents the movement of tourists but also reveals the diversity of tourism resources, making it an important tool for studying tourism phenomena. Tourist flow can be broadly classified into general and narrow categories, and this study focuses on narrow tourist flow^[14]^. In recent years, with the rise of tourism big data, the academic community has conducted an increasing number of studies on tourist flow, with most of them focusing on the spatial characteristics of tourist flow, their influencing factors, and their correlations.

The spatial structure of tourism refers to the spatial scale and degree of aggregation formed by the interaction of tourism objects ^[15]^. This spatial scale and degree of aggregation can reflect the spatial attributes and interrelationships of tourism activities, providing guidance for the spatial planning of tourist attractions.Based on this, scholars have conducted extensive research on the spatial characteristics of tourist flow. In terms of research methods, most studies use data from tourism digital footprints such as online texts and images, and analyze the spatial characteristics of tourist flow using spatial analysis methods and social network analysis methods. The scales of the research objects include attractions ^[16]^, cities ^[17]^, provinces ^[18]^, urban agglomerations ^[19]^, regions ^[20]^, and the whole country ^[21]^, among others.Based on this, scholars have further analyzed the influencing factors of tourist flow, pointing out that the spatial structure of tourist flow is closely related to factors such as tourism resource endowment, transportation level, and distance ^[21]^. The number of private car ownership, total social consumer goods retail sales, and internet broadband user access also have a strong impact on tourist flow ^[23]^. In addition, Yang Li et al. (2023) found that the total volume of telecommunications services, the number of tourism industry employees, the number of accommodation enterprises, the number of corporate legal entities, and the total government investment in the tourism industry are important factors affecting tourism information flow ^[24]^.In terms of the correlation of tourist flow, most scholars have studied the coupling relationship between tourist flow and transportation based on a coupling perspective ^[25-26]^. Some scholars have also focused on other correlations of tourist flow, such as the correlation between tourist flow and the tourism environment ^[27]^, and the degree of coupling between tourism and the economy ^[28]^. A few scholars have also studied the impact of haze ^[29]^, transportation ^[30]^, policies ^[31]^, language ^[32]^, natural disasters, and trade openness ^[33]^ on tourist flow.

**9. It is necessary to add the current research methods, the network attention and the transformation methods of tourism flow in international research.**

**Revision note:**Thank you for the comments provided by the reviewer. In response to the aforementioned suggestions, this study has been revised accordingly. The modifications made are as follows (please refer to pages 6 to 8 of the paper for further details).

The internet provides an important channel for tourists to obtain travel information. When tourists collect and browse travel information on the internet, they leave behind their browsing history, which is referred to as tourism network attention. Tourism network attention can reflect the level of public interest in tourist destinations, as well as reveal the characteristics of public needs and behavioral intentions ^[34]^. Therefore, scholars have focused on studying network attention in the field of tourism.

Currently, there are two main methods for measuring network attention: one is based on Baidu index, and the other is based on constructing a network attention index using multiple sources of data. Reviewing relevant literature reveals that most studies use Baidu index as the platform and obtain raw data on tourism network attention through keyword searches. Additionally, many studies utilize spatial analysis methods to explore the spatiotemporal characteristics of different research subjects.Yang Li et al. (2017) investigated the network attention of national park-like attractions in the Yangtze River Delta in China based on Baidu index data ^[35]^. Subsequently, Sun Xiaobei et al. (2018) conducted a study on the network attention of cave attractions in China ^[36]^. Furthermore, Shu Li et al. (2020) explored the characteristics of network attention towards sports tourism in China using Baidu index data ^[37]^. Subsequently, there has been an increasing number of studies on network attention.However, when the research area includes multiple tourism resources, it is difficult to obtain comprehensive and effective data using Baidu index as an indicator ^[38]^. Therefore, it is necessary to construct a network attention using multiple sources of data. For example, some scholars have used the network attention of websites such as Qunar, Ctrip, and Dianping as data to study the coupling between network attention and scenic attraction ^[39]^.

At the same time, scholars have also paid attention to the factors influencing the level of network attention to tourism. Research has found that factors such as economic level, population size, distance, climate, and transportation are important factors influencing network attention ^[40-41]^. Additionally, the number of traffic accidents has a significant impact on the level of network attention towards tourism safety ^[42]^, and the degree of trade openness and international tourism openness can affect the level of international tourism network attention ^[43]^. Apart from the aforementioned factors, special events ^[44]^, negative information ^[45]^, and policies ^[46]^ also have a significant impact on network attention. Some scholars have also studied the correlation between network attention and tourist volume ^[47-48]^, abundance of tourism resources ^[49]^, and tourism attractiveness ^[50]^. These studies have greatly enriched the research on network attention and provided valuable references for this study.

In addition, scholars have proposed methods for converting network attention into tourist flow from the perspective of measures and suggestions. As tourism network attention has predictive significance, most scholars believe that, on one hand, scenic areas should promptly pay attention to network attention information (such as online searches, browsing, ticket purchases, etc.) and prepare for tourism reception in advance. On the other hand, scenic areas should attach importance to the dissemination and marketing role of the internet in order to attract tourists. Furthermore, Lan Xue et al. (2023) constructed an index for the conversion of network attention into tourist flow, and proposed a quantitative measurement method , further deepening the study of the relationship between network attention and tourist flow.

**10. This is an international journal, so the research area in Figure 1 needs to supplement the location of Shaanxi Province in China, and it needs to be added the basic information including specific names and types of red tourist attractions.**

**Revision note:**Thank you for the comments provided by the reviewer. In response to the suggestions raised, this study has made revisions to Figure 1. The modifications are as follows, and for further details, please refer to page 10 of the paper.

**Fig.1 Distribution map of classic red tourism attractions in Shaanxi province**

**Note: This map is drawn based on the standard map of the ministry of natural resources of China (surveying and mapping approval No. GS(2019)1822), and the base map remains unaltered. The same applies to Fig.2.**

**11. There are over 150 red tourism areas in Shanxi province, why only 40 classic red tourism areas were selected?**

**Revision note:**Thank you for the reviewer's comments. In response to the suggestions, we would like to provide the following explanation: Initially, this study aimed to investigate all the red tourism attractions in Shaanxi Province. However, during the data collection process, it was discovered that some of the tourism sites had missing data and lacked data validity. Therefore, we decided to select representative red tourism classic scenic areas with complete and reliable data as the case study site.

**12. It is recommended to provide additional explanations on technologies such as Baidu coordinate system picking tool for international readers.**

**Revision note:**Thank you for the reviewer's comments. In response to the suggestions provided, the present study has made the following modifications:

Using Baidu coordinate picking tool, the longitude and latitude of the case study site were obtained to create a spatial distribution map of the red tourism classic attractions in Shaanxi province (Fig 1).(The Baidu coordinate system picking tool (http://api.map.baidu.com/lbsapi/getpoint/index.html) is a tool used for obtaining geographical location information of tourist destinations, including the latitude and longitude coordinates of scenic spots.)（Please refer to page 10 of the paper for further details.）

**13. There are many professional terms of tourist attractions in Table 1. It is recommended to check the English expression.**

**Revision note:**Thank you for the reviewer's comments. In response to the aforementioned suggestions, this study has been revised accordingly. Please refer to the main body of the manuscript for further details.

**14. The figure of spatial distribution of Internet attention of tourist attractions needs to be added.**

**Revision note:**Thank you for the comments provided by the reviewers. In response to the suggestions, this study has incorporated a spatial distribution map of online attention. The modifications made are detailed in the revised version, please refer to page 17 of the paper for further information.

**Fig.2 Distribution of network attention**

**15. In Figure 2, it is difficult to clearly distinguish the relationship between 40 tourist attractions, especially the nodes level and position in tourist attraction network.**

**Revision note:**Thank you for the comments provided by the reviewers. In response to the suggestions, this study has made revisions to Figure 2 as follows. For further details, please refer to page 19 of the paper.

**Fig.3 The network structure of tourism flow (S2 Table)**

**(Note: Larger nodes in the graph indicate greater importance of the corresponding nodes. Thicker lines represent denser tourism flows between nodes.)**

**16. For the comparison of core and edge nodes in the network structure, it is recommended to add core-edge analysis.**

**Revision note:**Thank you for the reviewer's comments. In response to the suggestions, this study has incorporated core-periphery analysis, and the revised content is as follows (please refer to pages 19-20 of the paper for details).

**Core-edge**

According to Table 4, Zaoyuan revolution site, Yan’an revolution memorial hall, Wangjiaping revolution site, Yangjialing revolution site, and Baota mount are located at the core positions of the network. This indicates that these nodes have strong aggregation and diffusion capabilities, making them core nodes. The density of the core nodes is 0.095, which is relatively small, indicating that the tourist flow between core nodes is not closely connected (Table 5). There are a large number of edge nodes in the network, scattered throughout Shaanxi province. Representative nodes include Anwuqing training revolution site. The density between edge nodes is 0.004, indicating a significant hierarchical differentiation in the structure of the tourist flow network. The density between the core and edge regions is 0.008, and the density between the edge and core regions is 0.005. This indicates that the core nodes hold an advantageous position in the exchange relationship with edge nodes, and the tourism development capacity of edge nodes needs to be improved.

**Table 4. Example of core-edge results (N=93)**

|  | **Core nodes** | **Edge nodes** |
| --- | --- | --- |
| **Core nodes** | Zaoyuan revolution site,Yan’an revolution memorial hall,Wangjiaping revolution site,Yangjialing revolution site,Baota mount,Xi’an incident memorial hall | Chenghuang temple  ,Yuanjia village  ,Zhi Yuan |
| **Edge nodes** | Qin terracotta army,  Daming palace national park | Anwuqing training revolution site,Yonglezhai |

**Table 5. Density of core-edge**

|  | **Density of core regions** | **Density of edge regions** |
| --- | --- | --- |
| **Density of core regions** | 0.095 | 0.008 |
| **Density of edge regions** | 0.005 | 0.004 |

**17. Why are 96 nodes (divided into 8 groups) composed of small-world phenomenon?**

**Revision note:**Thank you for the reviewer's comments. In response to the suggestions provided, this study would like to address the following points: The results were obtained through software analysis. Additionally, upon re-examining the data, it was found that the characteristics of small-world phenomena align with the core-edge features. As this constitutes redundant content, the analysis on small-world properties has been removed. For further details, please refer to the main text of the manuscript.

**18. What is the basis for excluding small worlds with a total number of nodes less than 5?**

**Revision note:**Thank you for the comments provided by the reviewer. In response to the suggestions, this study provides the following reply: If the scale of the small-world network is too small, its structure becomes unstable. After conducting repeated experiments, small-world networks with a total of five nodes or less were removed, resulting in a final set of five small-world networks ^[38]^. Furthermore, upon re-examining the data, it was found that the characteristics of the small-world network align with the core-edge structure, which is redundant information. Therefore, the analysis of the small-world network has been removed. For more details, please refer to the main text of the manuscript.

**19. Table 4 has shown the characteristics of the small world, so it is suggested to be deleted: " According to Table 4, it can be seen that the core nodes are mostly located in Yan’an City. Among them, ..., Additionally, it can be observed that non-red nodes (Yan’an 1938, Hukou Waterfall, etc.) and red nodes jointly construct the overall network structure, exerting a significant influence within the network structure."**

**Revision note:**Thank you for the comments provided by the reviewer. In response to the aforementioned suggestions, this study has removed the mentioned paragraph. For more information, please refer to the main body of the paper.

**20. The correlation needs to be verified by continuous data, rather than by observing only three data.**

**Revision note:**Thank you for the comments provided by the reviewer. In response to this suggestion, we would like to address the following points: Firstly, currently there is no available data on the interconnections between network attention, node weighted degree, and comprehensive ranking value, thus making it impossible to conduct a correlation analysis. Additionally, upon revising the manuscript, we found that both node weighted degree and comprehensive ranking value fall under the analysis of the importance of travel flow nodes, which is repetitive content (see reference 38). Moreover, there is limited literature on the research of comprehensive ranking value. Therefore, the content related to comprehensive ranking value has been removed in the revised version. Furthermore, building upon existing research, this study investigates the characteristics of online attention and node weighted degree based on the Boston matrix analysis method. The specific details are presented in the following section (for more information, please refer to pages 22 to 24 of the paper).

The Boston matrix, also known as the market growth rate-relative market share matrix ^[56]^, suggests that under the interaction of market growth rate and relative market share, there are four different types of products: bright-star products (high sales growth rate, high market share), thin-dog products (low sales growth rate, low market share), question products (high sales growth rate, low market share), and cash-cow products (low sales growth rate, high market share).Based on the Boston matrix, Li Jingyi et al. (2002) creatively constructed a model that reflects the tourism market ^[57]^, which has been widely applied in tourism research. Currently, this method has been extensively adopted by scholars in the field of tourism research.Based on the analysis in the previous section, it can be concluded that there is a correlation between network attention and tourist flow. Therefore, this study draws on the division method of the Boston matrix and relevant tourism research ^[58-59]^ to determine the criteria for dividing tourist attractions based on network attention and node weighted degree. Similarly, tourist attractions are divided into bright-star attractions, cash-cow attractions, thin-dog attractions, and question attractions. The division of each type of attraction is shown in Table 7.

**Table 7. Features of network attention and node weighted degree (example)**

| **Bright-star attractions** | **Cash-cow attractions** | **Thin-dog attractions** | **Question attractions** |
| --- | --- | --- | --- |
| Zaoyuan revolution site,  Wangjiaping revolution site,  Yan’an revolution memorial hall | Yangjialing revolution site,  Baota mount | Xi’an incident memorial hall,  Nanniwan revolution site | Qingliang mountain revolution site,  Revolution site of the northwest bureau ,Phoenix mountain revolution site,Qiaoergou revolution site |

According to Table 7, it can be observed that there is a correlation between tourist flow and network attention, but they also exhibit a ‘misplacement’ characteristic.Specifically, in terms of bright-star attractions: this type of attraction has a high level of network attention and node weighted degree. The main representative attractions are Zaoyuan revolution site, Wangjiaping revolution site, and Yan’an revolution memorial hall. This category already possesses significant online influence and should be further leveraged to drive synergistic effects among these attractions in the future. Cash-cow attractions: this type of attraction is characterized by high network attention and low node weighted degree. The representative attractions are Yangjialing revolution site and Baota mount. This category attracts a considerable number of potential tourists, and therefore, it is necessary to enhance the tourism reception capacity of such attractions in the future. Thin-dog attractions: this type of attraction is characterized by low network attention and high node weighted degree. Representative attractions include Xi’an incident memorial hall and Nanniwan revolution site. In the future, innovative marketing and promotional methods need to be implemented to enhance their tourism influence. Question attractions: this type of attraction has both low network attention and node weighted degree, encompassing most of the attractions in the case study area. Representative attractions include Qingliang mountain revolution site, and Qiaoergou revolution site. It is urgent to develop multidimensional tourism development measures for these attractions.

**21. What is the basis of the group division in Table 6?**

**Revision note:**Thank you for the reviewer's comments. In response to the suggestions provided, the present study has made the following modifications (for details, please refer to pages 22-24 of the paper).

The Boston matrix, also known as the market growth rate-relative market share matrix ^[56]^, suggests that under the interaction of market growth rate and relative market share, there are four different types of products: bright-star products (high sales growth rate, high market share), thin-dog products (low sales growth rate, low market share), question products (high sales growth rate, low market share), and cash-cow products (low sales growth rate, high market share).Based on the Boston matrix, Li Jingyi et al. (2002) creatively constructed a model that reflects the tourism market ^[57]^, which has been widely applied in tourism research. Currently, this method has been extensively adopted by scholars in the field of tourism research.Based on the analysis in the previous section, it can be concluded that there is a correlation between network attention and tourist flow. Therefore, this study draws on the division method of the Boston matrix and relevant tourism research ^[58-59]^ to determine the criteria for dividing tourist attractions based on network attention and node weighted degree. Similarly, tourist attractions are divided into bright-star attractions, cash-cow attractions, thin-dog attractions, and question attractions. The division of each type of attraction is shown in Table 7.

**Table 7. Features of network attention and node weighted degree (example)**

| **Bright-star attractions** | **Cash-cow attractions** | **Thin-dog attractions** | **Question attractions** |
| --- | --- | --- | --- |
| Zaoyuan revolution site,  Wangjiaping revolution site,  Yan’an revolution memorial hall | Yangjialing revolution site,  Baota mount | Xi’an incident memorial hall,  Nanniwan revolution site | Qingliang mountain revolution site,  Revolution site of the northwest bureau ,Phoenix mountain revolution site,Qiaoergou revolution site |

According to Table 7, it can be observed that there is a correlation between tourist flow and network attention, but they also exhibit a ‘misplacement’ characteristic.Specifically, in terms of bright-star attractions: this type of attraction has a high level of network attention and node weighted degree. The main representative attractions are Zaoyuan revolution site, Wangjiaping revolution site, and Yan’an revolution memorial hall. This category already possesses significant online influence and should be further leveraged to drive synergistic effects among these attractions in the future. Cash-cow attractions: this type of attraction is characterized by high network attention and low node weighted degree. The representative attractions are Yangjialing revolution site and Baota mount. This category attracts a considerable number of potential tourists, and therefore, it is necessary to enhance the tourism reception capacity of such attractions in the future. Thin-dog attractions: this type of attraction is characterized by low network attention and high node weighted degree. Representative attractions include Xi’an incident memorial hall and Nanniwan revolution site. In the future, innovative marketing and promotional methods need to be implemented to enhance their tourism influence. Question attractions: this type of attraction has both low network attention and node weighted degree, encompassing most of the attractions in the case study area. Representative attractions include Qingliang mountain revolution site, and Qiaoergou revolution site. It is urgent to develop multidimensional tourism development measures for these attractions.

**22. The discussion on the four types of scenic spots is not supported by literature, and the relationship between discussion and the conclusion is not very close, and the reasons for the formation of the four types were not be analyzed.**

**Revision note:**Thank you for the comments provided by the reviewers. In response to the aforementioned suggestions, this study has made the following modifications. For more details, please refer to pages 22 to 29 of the paper.

The Boston matrix, also known as the market growth rate-relative market share matrix ^[56]^, suggests that under the interaction of market growth rate and relative market share, there are four different types of products: bright-star products (high sales growth rate, high market share), thin-dog products (low sales growth rate, low market share), question products (high sales growth rate, low market share), and cash-cow products (low sales growth rate, high market share).Based on the Boston matrix, Li Jingyi et al. (2002) creatively constructed a model that reflects the tourism market ^[57]^, which has been widely applied in tourism research. Currently, this method has been extensively adopted by scholars in the field of tourism research.Based on the analysis in the previous section, it can be concluded that there is a correlation between network attention and tourist flow. Therefore, this study draws on the division method of the Boston matrix and relevant tourism research ^[58-59]^ to determine the criteria for dividing tourist attractions based on network attention and node weighted degree. Similarly, tourist attractions are divided into bright-star attractions, cash-cow attractions, thin-dog attractions, and question attractions. The division of each type of attraction is shown in Table 7.

**Table 7. Features of network attention and node weighted degree (example)**

| **Bright-star attractions** | **Cash-cow attractions** | **Thin-dog attractions** | **Question attractions** |
| --- | --- | --- | --- |
| Zaoyuan revolution site,  Wangjiaping revolution site,  Yan’an revolution memorial hall | Yangjialing revolution site,  Baota mount | Xi’an incident memorial hall,  Nanniwan revolution site | Qingliang mountain revolution site,  Revolution site of the northwest bureau ,Phoenix mountain revolution site,Qiaoergou revolution site |

According to Table 7, it can be observed that there is a correlation between tourist flow and network attention, but they also exhibit a ‘misplacement’ characteristic.Specifically, in terms of bright-star attractions: this type of attraction has a high level of network attention and node weighted degree. The main representative attractions are Zaoyuan revolution site, Wangjiaping revolution site, and Yan’an revolution memorial hall. This category already possesses significant online influence and should be further leveraged to drive synergistic effects among these attractions in the future. Cash-cow attractions: this type of attraction is characterized by high network attention and low node weighted degree. The representative attractions are Yangjialing revolution site and Baota mount. This category attracts a considerable number of potential tourists, and therefore, it is necessary to enhance the tourism reception capacity of such attractions in the future. Thin-dog attractions: this type of attraction is characterized by low network attention and high node weighted degree. Representative attractions include Xi’an incident memorial hall and Nanniwan revolution site. In the future, innovative marketing and promotional methods need to be implemented to enhance their tourism influence. Question attractions: this type of attraction has both low network attention and node weighted degree, encompassing most of the attractions in the case study area. Representative attractions include Qingliang mountain revolution site, and Qiaoergou revolution site. It is urgent to develop multidimensional tourism development measures for these attractions.

**Conclusion**

This study investigates the characteristics of network attention and tourist flow in the classic red tourism attractions in Shaanxi province, China, using social network theory and other methods. The findings are as follows:

(1) The network attention to the case study sites is relatively low (with a mean value of 0.340238677), and there is a significant difference in network attention among different attractions. The promotional and advertising capabilities of attractions on the internet need to be improved urgently. In terms of spatial distribution, the network attention is unevenly distributed. This is mainly reflected in the higher network attention of attractions in Yan’an city, while the network attention of attractions in other regions is relatively low. The abundance of tourism resources, level, and popularity of scenic areas are closely related to network attention.(2) There are variations in the network attention received by different types of attractions. Attractions with higher rankings tend to receive higher network attention, while those with lower rankings tend to receive lower network attention. Additionally, archaeological sites exhibit higher network attention, indicating that tourists’ preferences have a certain influence on the network attention received.(3) The network density of tourist flow in the case study area is low (0.023), indicating a lack of close tourism connections between nodes. The interconnectivity between core and edge nodes is poor. Developed tourist routes are only present among the core tourism nodes. Edge nodes are in a disadvantaged position within the network structure, and their tourism capacity needs improvement.(4) Zaoyuan revolution site, Yangjialing revolution site, Wangjiaping revolution site, and other nodes have a significant influence in the network structure. The connection between non-red nodes and red nodes is relatively close, playing an important role in the network structure.(5) There is a correlation between network attention and tourist flow, as well as a ‘misplacement’ feature. Based on the Boston matrix, attractions can be divided into bright-star attractions, cash-cow attractions, thin-dog attractions, and question attractions.

**Discussion**

In the context of the internet, various social platforms have provided new channels for tourists to obtain travel information, which has also brought new opportunities and challenges to the development of red tourism scenic spots. Network attention, as an important indicator of the promotion and publicity capability of tourist attractions on the internet, should be given due attention. Currently, there are various channels for promoting tourist attractions, such as websites and software, which provide convenience for this purpose. The measurement of network attention should be conducted from multiple perspectives. Therefore, this study has constructed a dataset of network attention from multiple sources. Based on this background, this study analyzes the network attention and tourist flow characteristics of red tourism classic attractions in Shaanxi province, China, in order to promote the high-quality development of red tourism under the ‘internet+’ background.

Research has found that the network attention to red attractions develops unevenly, and relevant scenic areas should pay attention to this situation. Additionally, there is a close connection between non-red attractions and red attractions in terms of tourist flow, which brings new opportunities for the development of attractions. The study also reveals a certain relationship between network attention and tourist flow.High-level, high-quality, and well-known attractions have a higher level of network attention. However, there is also a ‘misplacement’ feature between network attention and tourist flow. In other words, a scenic spot with high network attention does not necessarily have a large number of tourists. There are many factors that influence network attention, such as tourists’ preferences and policies, which can affect the level of network attention for tourism. In addition, red tourism has its own uniqueness. Red tourism is an important way to promote revolutionary culture and ideological education. Due to the attention given by government departments, red tourism has a large scale of online promotion, which also has a certain impact on the network attention of attractions. Therefore, under the background of ‘internet +’, scenic spots should not only focus on the effects of online promotion but also pay attention to the attributes of tourism resources, in order to promote the transformation

Please refer to the relevant literature for support, see 56-59.

Reviewer #5:

**(1) This paper is a typical case study, and the research conclusions have certain practical value for the development of red tourism in Shaanxi Province. However, the article does not seem to present any lessons and conclusions that can be generalized, which makes the actual contribution of the article very limited.**

**Revision note:**Thank you for the comments provided by the reviewers. In response to the aforementioned suggestions, the present study has made the following modifications (please refer to pages 27-29 of the paper for further details).

**Discussion**

In the context of the internet, various social platforms have provided new channels for tourists to obtain travel information, which has also brought new opportunities and challenges to the development of red tourism scenic spots. Network attention, as an important indicator of the promotion and publicity capability of tourist attractions on the internet, should be given due attention. Currently, there are various channels for promoting tourist attractions, such as websites and software, which provide convenience for this purpose. The measurement of network attention should be conducted from multiple perspectives. Therefore, this study has constructed a dataset of network attention from multiple sources. Based on this background, this study analyzes the network attention and tourist flow characteristics of red tourism classic attractions in Shaanxi province, China, in order to promote the high-quality development of red tourism under the ‘internet+’ background.

Research has found that the network attention to red attractions develops unevenly, and relevant scenic areas should pay attention to this situation. Additionally, there is a close connection between non-red attractions and red attractions in terms of tourist flow, which brings new opportunities for the development of attractions. The study also reveals a certain relationship between network attention and tourist flow.High-level, high-quality, and well-known attractions have a higher level of network attention. However, there is also a ‘misplacement’ feature between network attention and tourist flow. In other words, a scenic spot with high network attention does not necessarily have a large number of tourists. There are many factors that influence network attention, such as tourists’ preferences and policies, which can affect the level of network attention for tourism. In addition, red tourism has its own uniqueness. Red tourism is an important way to promote revolutionary culture and ideological education. Due to the attention given by government departments, red tourism has a large scale of online promotion, which also has a certain impact on the network attention of attractions. Therefore, under the background of ‘internet +’, scenic spots should not only focus on the effects of online promotion but also pay attention to the attributes of tourism resources, in order to promote the transformation and upgrading of tourist attractions comprehensively and from multiple perspectives.

**(2) Some of the specific contents of the article are not clear, which makes people very confused.**

For example: In the page 9, the author said:“ To ensure the integrity of the travel flow network structure, non-red attractions are retained in the travel routes. The travel route ‘Nanniwan→Wangjiaping→Zaoyuan’ is split into‘Nanniwan→Wangjiaping’ and ‘Wangjiaping→Zaoyuan’, resulting in 468 valid O-D data. ”

Here, the explanation of the data processing method is very sloppy, and it is impossible to understand the true intention of the author, and it cannot be assumed that all readers understand the research methods used by the author.

**Revision note:**Thank you for the comments provided by the reviewer. In response to the aforementioned suggestions, this study has made the following revisions. For more details, please refer to page 11 of the paper

Building upon existing research ^[38]^, to ensure the integrity of the tourist flow network structure, non-red attractions in the tourist flow routes were retained, and the aforementioned data was transformed into directed flow data between attractions. Ultimately, 468 valid origin-destination (O-D) data were obtained.

**Why is it that there are only 13 red tourist attractions and 40 red tourist attractions, but a matrix of 92 attractions can be constructed (Figure 2). Where does the extra part come from? What is their relationship with red tourist attractions?**

**Revision note:**Thank you for the reviewer's comments. In response to the suggestions provided, this study has made the following modifications. Please refer to page 11 of the paper for further details

Building upon existing research ^[38]^, to ensure the integrity of the tourist flow network structure, non-red attractions in the tourist flow routes were retained, and the aforementioned data was transformed into directed flow data between attractions. Ultimately, 468 valid origin-destination (O-D) data were obtained.

**(3) The author lacks a discussion of the limitations of the article.**

**Revision note:**Thank you for the reviewer's comments. In response to the aforementioned suggestions, this study has made the following modifications. For details, please refer to page 29 of the paper.

Due to objective constraints such as data availability and completeness, the author only obtained network data from six platforms. In the future, a quantitative model can be constructed to select comprehensive indicators. Additionally, the formation mechanism of network attention and the characteristics of tourist flow misplacement are also important research directions of concern.

**(4) The content, structure and presentation of all images in the article need to be further optimized.**

**Revision note:**Thank you for the reviewer's comments. In response to the aforementioned suggestions, we have made improvements and revisions to the manuscript. Please refer to the full text of the manuscript for details.

Reviewer #6：

**a) The theoretical framework has only been mentioned without any additional explanations (e.g. why and for which reasons the Authors used the theory of social network analysis**

**Revision note:**Thank you for the reviewer's comments. In response to the suggestions provided, the present study has made the following modifications. For further details, please refer to pages 14-15 of the paper.

Social network theory posits that social groups are interconnected networks in which actors (nodes) mutually influence each other. This theory assesses the importance of nodes in the network from a relational perspective and analyzes the significance of both individual nodes and the overall network based on the characteristics of the network structure ^[55]^. It has been widely applied in tourism research. This study adopt indicators such as size and density, core-edge, and node weighted degree to analyze the characteristics of tourist flow, drawing on this theory. Size and density represent the connections between nodes in the network. A larger value indicates closer connections between nodes, and vice versa. Node weighted degree reflects the diffusion and aggregation capacity of nodes. A higher value indicates greater capacity of the node. Core-edge is used to analyze the importance of individual nodes in the network.

**b) Please give the definition of the “red tourism.”**

**Revision note:**Thank you for the comments provided by the reviewer. In response to the aforementioned suggestions, this study has made the following modifications. For further details, please refer to pages 1 to 2 of the paper.

Red tourism is a specialized form of tourism in China that revolves around revolutionary memorial sites, landmarks, and other related elements. It focuses on the revolutionary history, achievements, and spirit ^[1]^. Unlike other types of tourism, red tourism is unique to China and serves as an important means to promote Chinese revolutionary culture. The Chinese government attaches great importance to the development of red tourism, resulting in a strong market demand and promising future prospects for red tourism in China.

**c) I’m not sure if the data of tourist traffic are correct: I do not understand “the annual number of participants in red tourism has increased from 140 million to 1.41 billion”**

**Revision note:**Thank you for the reviewer's comments. In response to the aforementioned suggestion, this study has removed the sentence in question. Please refer to the main body of the manuscript for further details.

**d) In many parts of the article, we can find some repetitions. The text should be cut.**

**Revision note:**Thank you for the reviewer's comments. In response to the aforementioned suggestions, this study has been revised. Please refer to the revised manuscript for details.

**e) Probably the most important value of the paper is their methodological aspect(s). Please try to develop this part and give some conclusions in the final subchapter. The Authors can use some other paper on the problems with traffic (flow) research published in last years, e.g. https://www.geographiapolonica.pl/article/item/13268.html**

**e) transportation conditions [31-32], and accessibility which is one of the most important part of relationships between tourism and transport (please see https://sciendo.com/pl/article/10.1515/mgr-2015-0002 or other papers of the same Authors)**

**Revision note:**Thank you very much for the comments provided by the reviewer. In response to the aforementioned suggestions, this study has been revised. The modifications made are as follows, for further details please refer to page 29 of the paper.

In the context of the internet, various social platforms have provided new channels for tourists to obtain travel information, which has also brought new opportunities and challenges to the development of red tourism scenic spots. Network attention, as an important indicator of the promotion and publicity capability of tourist attractions on the internet, should be given due attention. Currently, there are various channels for promoting tourist attractions, such as websites and software, which provide convenience for this purpose. The measurement of network attention should be conducted from multiple perspectives. Therefore, this study has constructed a dataset of network attention from multiple sources. Based on this background, this study analyzes the network attention and tourist flow characteristics of red tourism classic attractions in Shaanxi province, China, in order to promote the high-quality development of red tourism under the ‘internet+’ background.

Due to objective constraints such as data availability and completeness, the author only obtained network data from six platforms. In the future, a quantitative model can be constructed to select comprehensive indicators. Additionally, the formation mechanism of network attention and the characteristics of tourist flow misplacement are also important research directions of concern.

**d) Some figures are not readable, especially fig. 3**

**Revision note:**Thank you very much for the comments provided by the reviewer. In response to the aforementioned suggestions, this study has been revised accordingly. Please refer to the manuscript for further details.

**e) Conclusions are very narrow and related only to the case study. There is no any discussion to other research, results etc. Some results are very basic such as “The case sites have formed a spatial layout of the ‘dense in the north and sparse in the south’.”**

**Revision note:**Thank you for the reviewer's comments. In response to the aforementioned suggestions, this study has made the following modifications. For details, please refer to the paper on pages 1, and 24 to 29.

**Abstract**

This study takes the classic red tourism attractions in Shaanxi province, China as an example and constructs a multi-source data network attention evaluation index. Additionally, it employs social network theory to explore the network attention and tourist flow characteristics of the case study area.Research shows that: (1) Overall, the network attention to case-based destinations is relatively low, and there are significant differences in network attention among different attractions. Spatially, the distribution of network attention is uneven. This is manifested by higher network attention to attractions in Yan’an city and lower network attention to attractions in other regions.(2) There are differences in the network attention of different types of attractions. High-level attractions receive higher network attention, while low-level attractions receive lower network attention. Additionally, archaeological sites tend to receive higher network attention.(3) The network density of tourist flow is low, and the tourism connections between nodes are not closely linked. The linkage between core nodes and edge nodes in tourism is poor. Developed tourism routes only exist in core nodes.(4) Nodes such as Zaoyuan revolution site, Yangjialing revolution site, and Wangjiaping revolution site have a significant influence in the network structure. In addition, non-red nodes play an important role in the network structure, and red tourism is often combined with non-red tourism resources.(5) There is a correlation between network attention and tourist flow, as well as a ‘misplacement’ feature. Based on the characteristics of attractions, they can be divided into four types:bright-star attractions, cash-cow attractions, thin-dog attractions, and question attractions. Based on these conclusions, this study proposes targeted development suggestions for the four types of attractions based on the principle of ‘precise identification and classification strategies’.

**Conclusion**

This study investigates the characteristics of network attention and tourist flow in the classic red tourism attractions in Shaanxi province, China, using social network theory and other methods. The findings are as follows:

(1) The network attention to the case study sites is relatively low (with a mean value of 0.340238677), and there is a significant difference in network attention among different attractions. The promotional and advertising capabilities of attractions on the internet need to be improved urgently. In terms of spatial distribution, the network attention is unevenly distributed. This is mainly reflected in the higher network attention of attractions in Yan’an city, while the network attention of attractions in other regions is relatively low. The abundance of tourism resources, level, and popularity of scenic areas are closely related to network attention.(2) There are variations in the network attention received by different types of attractions. Attractions with higher rankings tend to receive higher network attention, while those with lower rankings tend to receive lower network attention. Additionally, archaeological sites exhibit higher network attention, indicating that tourists’ preferences have a certain influence on the network attention received.(3) The network density of tourist flow in the case study area is low (0.023), indicating a lack of close tourism connections between nodes. The interconnectivity between core and edge nodes is poor. Developed tourist routes are only present among the core tourism nodes. Edge nodes are in a disadvantaged position within the network structure, and their tourism capacity needs improvement.(4) Zaoyuan revolution site, Yangjialing revolution site, Wangjiaping revolution site, and other nodes have a significant influence in the network structure. The connection between non-red nodes and red nodes is relatively close, playing an important role in the network structure.(5) There is a correlation between network attention and tourist flow, as well as a ‘misplacement’ feature. Based on the Boston matrix, attractions can be divided into bright-star attractions, cash-cow attractions, thin-dog attractions, and question attractions.

**Discussion**

In the context of the internet, various social platforms have provided new channels for tourists to obtain travel information, which has also brought new opportunities and challenges to the development of red tourism scenic spots. Network attention, as an important indicator of the promotion and publicity capability of tourist attractions on the internet, should be given due attention. Currently, there are various channels for promoting tourist attractions, such as websites and software, which provide convenience for this purpose. The measurement of network attention should be conducted from multiple perspectives. Therefore, this study has constructed a dataset of network attention from multiple sources. Based on this background, this study analyzes the network attention and tourist flow characteristics of red tourism classic attractions in Shaanxi province, China, in order to promote the high-quality development of red tourism under the ‘internet+’ background.

Research has found that the network attention to red attractions develops unevenly, and relevant scenic areas should pay attention to this situation. Additionally, there is a close connection between non-red attractions and red attractions in terms of tourist flow, which brings new opportunities for the development of attractions. The study also reveals a certain relationship between network attention and tourist flow.High-level, high-quality, and well-known attractions have a higher level of network attention. However, there is also a ‘misplacement’ feature between network attention and tourist flow. In other words, a scenic spot with high network attention does not necessarily have a large number of tourists. There are many factors that influence network attention, such as tourists’ preferences and policies, which can affect the level of network attention for tourism. In addition, red tourism has its own uniqueness. Red tourism is an important way to promote revolutionary culture and ideological education. Due to the attention given by government departments, red tourism has a large scale of online promotion, which also has a certain impact on the network attention of attractions. Therefore, under the background of ‘internet +’, scenic spots should not only focus on the effects of online promotion but also pay attention to the attributes of tourism resources, in order to promote the transformation and upgrading of tourist attractions comprehensively and from multiple perspectives.

Due to objective constraints such as data availability and completeness, the author only obtained network data from six platforms. In the future, a quantitative model can be constructed to select comprehensive indicators. Additionally, the formation mechanism of network attention and the characteristics of tourist flow misplacement are also important research directions of concern.

**7. PLOS authors have the option to publish the peer review history of their article (what does this mean?). If published, this will include your full peer review and any attached files.**

**Revision note:**Authors may choose not to disclose the peer review history of their articles.

Lastly, we would like to express our sincere gratitude to the reviewers and editors for their constructive feedback and guidance on this paper. I wish you all the best in your work and hope that PLOS ONE will continue to thrive and prosper.
