# Supplementary material for: The network characteristics of classic red tourist attractions in Shaanxi province, China
Source: PLoS One. 2024 Mar 29;19(3):e0299286. doi: 10.1371/journal.pone.0299286 (PMC10980247; doi:10.1371/journal.pone.0299286)
Supplement: Attachment — Submitted filename: Response to Reviewers.docx [file pone.0299286.s012.docx]

**Modification instructions**

Dear editors and reviewers of PLOS ONE:

Thank you for giving us the opportunity to submit a revised draft of the manuscript ‘The network characteristics of classic red tourist attractions in Shaanxi province, China ’ for publication in the Journal of PLOS ONE. We appreciate the time and effort that you and the reviewers dedicated to providing feedback on our manuscript and are grateful for the insightful comments on and valuable improvements to our paper. We have incorporated most of the suggestions made by the reviewers. Those changes are highlighted in the manuscript. Please see below, in blue, for a point-by-point response to the reviewers’ comments and concerns. All page numbers refer to the revised manuscript file with tracked changes.

Reviewer #4

**1. Although this study modified the abstract, the abstract lacks research background, framework, significance and innovative conclusions, and some sentences are unclear. For example, non-red nodes play an important role in the network structure. What role does it play?**

**Revision note**: Thank you for the reviewer's comments. We have made modifications to the abstract section, and the revised version is as follows. For more details, please refer to pages 1-2 of the paper.

Red tourism is a distinctive form of tourism in China. Its network attention serves as a typical indicator to measure the level of promotion and publicity for red tourism, as well as an important reflection of its influence.Understanding the network structure of red tourism is of significant importance for optimizing the spatial pattern of tourism and promoting the development of the tourism industry. Based on this,this study takes the classic red tourism attractions in Shaanxi province, China as an example and constructs a multi-source data network attention evaluation index. Additionally, it employs social network theory to explore the network attention and tourist flow characteristics of the case study area.Research shows that: (1) Overall, the network attention to case-based destinations is relatively low, and there are significant differences in network attention among different attractions. Spatially, the distribution of network attention is uneven. This is manifested by higher network attention to attractions in Yan’an city and lower network attention to attractions in other regions.(2) There are differences in the network attention of different types of attractions. High-level attractions have a higher level of online attention, while low-level attractions have a lower level of network attention. Additionally, archaeological sites tend to receive a higher level of online attention.(3) The network density of tourist flow is low, and the tourism connections between nodes are not closely linked. The linkage between core nodes and edge nodes in tourism is poor. Developed tourism routes only exist in core nodes.(4) Nodes such as Zaoyuan revolution site, Yangjialing revolution site, and Wangjiaping revolution site have a significant influence in the network structure. In addition, the integration and development between red nodes and non-red nodes have been achieved.(5) There is a correlation between network attention and tourist flow, as well as a ‘misplacement’ feature. Based on the characteristics of attractions, they can be divided into four types:bright-star attractions, cash-cow attractions, thin-dog attractions, and question attractions.Based on the above conclusions, this study proposes targeted development recommendations.

**2. Due to the lack of data in some tourist sites and the lack of data validity, I doubt that this will affect your research conclusions**.

**Revision note:**Thank you for the reviewer's comments.We made the following response: Due to the large number of subjects in this study, we chose to use multiple sources as data platforms. Initially, we collected data from eight platforms that showed good data validity. After applying entropy value processing, we eliminated data from two platforms and ultimately retained data from six platforms, all of which demonstrated good validity. The data from different platforms exhibit variations, but they all effectively represent the characteristics of network attention.

**3. The reason why the 96 nodes are divided into 8 groups needs to be explained in detail, Why is the software analysis can divide nodes into 8 groups by default？**

**Revision note:**Thank you for the reviewer's comments.By further revising the article, this paper has made modifications to this particular section. For detailed information, please refer to the main body of the paper.

**4. A ternary closure forms a minimal stable structure: a Loop containing three nodes.**

**Revision note:**Thank you for the reviewer's comments.This content is not mentioned in the present article. For further details, please refer to the main body of the paper.

**5. The group classification can be well explained by the Boston matrix. However, the limitations of this method should also be noted, and it is difficult to balance two or more conditions at the same time.**

**Revision note:**Thank you for the reviewer's comments.We respond as follows: In this article, we have conducted a further review of the relevant literature on the Boston matrix and found that there are numerous applications concerning the relationship between the two. Therefore, we have applied this matrix to analyze the relationship between network attention and tourist flow. In our subsequent studies, we will also intensify our learning on the application of this matrix in order to gain a comprehensive understanding of its applications.

**6. The manuscript has been revised a lot. It is suggested to readjust the logic between the paragraphs and check the English editing.**

**Revision note:**Thank you for the reviewer's comments.We have conducted a thorough reading and revision of the entire document. Please refer to the main body of the paper for further details.

Reviewer #5:

**(1) In the data part, the author only introduces the data of network attention, and does not introduce the data of tourism flow, which is obviously inappropriate. Therefore, I suggest that the author add a description of the tourist flow data.**

**Revision note:**Thank you for the reviewer's comments.We have made revisions to the relevant content. The modified content is as follows. For further details, please refer to page 10 of the paper.

Building upon existing research ^[39]^, to ensure the integrity of the tourist flow network structure, non-red attractions in the tourist flow routes were retained, and the aforementioned data was transformed into directed flow data between attractions. Ultimately, 468 valid origin-destination (O-D) data were obtained.

**(2) The references of the article need to be adjusted somewhat. At present, most of the references in the article are from Chinese journals, and there is no good academic dialogue with English journals. In fact, there are quite a lot of international studies on red tourism, tourism flow, and tourism network attention, and the authors should make appropriate adjustments to the literature.**

**Revision note：**Thank you for the reviewer's comments,we have made modifications to the references. Please refer to the main body of the paper for details.

Reviewer #6:

**1.Some of the changes may still be questionable. Overall, the article is China-centric and, in my opinion, the lack of reference to global literature on the subject is a mistake.**

**Revision note：**Thank you for the reviewer's comments,we have made modifications to the references. Please refer to the main body of the paper for details.

**2.In my opinion, the discussion still has some limitations and is poorly anchored in international circulation and literature. This can significantly reduce the scope of the article's importance.**

**Revision note：**Thank you for the reviewer's comments,we have made modifications to the references. Please refer to the main body of the paper for details.

Research has revealed significant variations in the development of network attention for red tourism destinations. Non-red tourism destinations exhibit a closer tourism connection with red tourism destinations, which brings forth new insights for the development of these destinations.The study also found that there is a certain relationship between network attention and tourist flow. This relationship is mainly manifested in scenic spots that are of high level, good quality, and high popularity, as they tend to attract more network attention. Although previous studies have shown a significant positive correlation between network attention and visitor traffic ^[57]^, there is also a distinct ‘mismatch’ characteristic between network attention and tourist flow.This study found that high network attention does not necessarily result in high tourist flow in scenic areas.Specifically, there are numerous factors influencing network attention. Red tourism receives significant attention from Chinese government departments, leading to extensive online promotion and a substantial impact on the network attention of scenic areas. On the other hand, tourist flow is influenced by various factors such as resource endowment, transportation level, distance, and preferences ^[21-23]^.Therefore, there is both correlation and dislocation between network attention and tourist flow. In the context of ‘Internet+’, scenic spots should not only focus on the effect of online promotion but also take into account the ‘mismatch’ of network attention, promoting the transformation and upgrading of tourist attractions comprehensively and from multiple perspectives.

**3.You need to be careful with the definitions, for example the revised one regarding red tourism. This is not a concept that only applies to this type of tourism in China. It is more a term for communist or socialist elements that occur in many countries.**

**Revision note：**Thank you for the reviewer's comments.We have made revisions to the discussion section. The revised content is as follows. For details, please refer to pages 27-28 of the paper.

Under the guidance of the government, red tourism has rapidly developed, which has sparked scholarly research on red tourism.In China, red tourism generally refers to themed tourism activities that revolve around revolutionary commemorative sites and landmarks, with a focus on the history, events, and spirit of the revolution ^[1]^.Red tourism, unlike other types of tourism, serves as a significant avenue for promoting revolutionary culture. The Chinese government attaches great importance to the development of red tourism, resulting in a strong market demand and promising future prospects for red tourism in China. In recent years, red tourism has experienced rapid growth under the government’s promotion.On March 23, 2021, People’s Daily pointed out that from 2004 to 2019, China’s red tourism resources have been continuously expanding, and the red tourism market has become increasingly active. The ‘Report on the development of China’s red tourism (2022)’ states that in 2022, the cumulative number of tourists received in China’s red tourism reached 3.478 billion, with a comprehensive income of 929.5 billion yuan. Red tourism has gradually become an important driving force for China’s tourism development.

Lastly, we would like to express our sincere gratitude to the reviewers and editors for their constructive feedback and guidance on this paper. I wish you all the best in your work and hope that PLOS ONE will continue to thrive and prosper.
